# Genome-wide identification, evolution, and transcript profiling of Aldehyde dehydrogenase superfamily in potato during development stages and stress conditions

**DOI:** 10.1038/s41598-021-97691-9

**Published:** 2021-09-14

**Authors:** Md. Sifatul Islam, Md. Soyib Hasan, Md. Nazmul Hasan, Shamsul H. Prodhan, Tahmina Islam, Ajit Ghosh

**Affiliations:** 1 Department of Biochemistry and Molecular Biology, Shahjalal University of Science and Technology, Sylhet, 3114 Bangladesh; 2 Department of Genetic Engineering and Biotechnology, Shahjalal University of Science and Technology, Sylhet, 3114 Bangladesh; 3 Department of Botany, University of Dhaka, Dhaka, 3114 Bangladesh

**Keywords:** Plant genetics, Plant stress responses, Plant sciences

## Abstract

The Aldehyde dehydrogenase (ALDH) superfamily comprises a group of enzymes involved in the scavenging of toxic aldehyde molecules by converting them into their corresponding non-toxic carboxylic acids. A genome-wide study in potato identified a total of 22 *ALDH* genes grouped into ten families that are presented unevenly throughout all the 12 chromosomes. Based on the evolutionary analysis of ALDH proteins from different plant species, ALDH2 and ALDH3 were found to be the most abundant families in the plant, while ALDH18 was found to be the most distantly related one. Gene expression analysis revealed that the expression of *StALDH* genes is highly tissue-specific and divergent in various abiotic, biotic, and hormonal treatments. Structural modelling and functional analysis of selected StALDH members revealed conservancy in their secondary structures and cofactor binding sites. Taken together, our findings provide comprehensive information on the *ALDH* gene family in potato that will help in developing a framework for further functional studies.

## Introduction

Plants are exposing to different types of abiotic and biotic stresses either alone or in combination throughout their entire lifespan, which has a detrimental impact on their overall growth and ultimately yield^1,2^. Under the influence of unfavourable environmental conditions, there is an increased amassment of the cellular toxic compounds as well as reactive oxygen species (ROS), which results in enhanced aldehyde production^3^. Aldehyde molecules are intermediate of various cellular and metabolic processes but accumulate in response to stress conditions, such as drought, salinity, desiccation, cold, and heat^4^. Excess aldehyde production has a destructive effect on plant metabolism that leads to cell injury^5^. Hence, the exclusion/neutralization of this excess aldehyde is obligatory for the plant for its survivability and functionality.

A family of detoxifying enzymes named aldehyde dehydrogenases (ALDHs; enzyme class EC: 1.2.1.3) play a vital role in scavenging active aldehyde molecules and thus, provide stress tolerance to plant^6^. The NAD(P)^+^ dependent ALDH superfamily enzymes convert endogenous and exogenous aromatic/aliphatic aldehydes to their corresponding non-toxic carboxylic acids by irreversible oxidation^6^ and maintain aldehyde homeostasis to get accustomed to environmental fluctuations. Expression of *ALDH* transcripts was found to be high in a variety of tissues of *Oryza sativa*^7^, *Brassica rapa*^8^, and *Glycine max*^9^. In most of the analyzed plant species, these genes are found to be upregulated under salinity, drought, and heat conditions, as well as responsive to different hormones indicating their role in such stress adaptation pathways^4,8,10^. ALDH proteins have been classified into 24 families identified from both prokaryotes and eukaryotes, according to the ALDH Gene Nomenclature Committee (AGNC)^11^. Among the total of 24 families, 14 ALDH families (ALDH- 2, 3, 5, 6, 7, 10, 11, 12, 18, 19, 21, 22, 23, and 24) have been reported in plant species and out of them seven ALDH families (ALDH- 11, 12, 19, 21, 22, 23, and 24) are exclusively found in plant species^12^. However, the presence of ALDH family- 23 and 24 has been only reported in *Physcomitrella patens*, *Chlamydomonas reinhardtii*^13^, and *Selaginella moellendorffii*^12^. ALDH protein superfamily has a conserved domain (PF00171) with unique catalytic, cofactor-binding, and oligomerization sites that could function alone or conjointly^10,14^. According to the crystal structure, the majority of the ALDHs has distinctive active site residues of cysteine (PS00070) and glutamic acid (PS00687) with unique Rossmann fold in their amino acid sequences. However, the foreseen position of the conserved cysteine and glutamic acid residues are diverse in the primary structure^10^. In addition to the conserved cysteine and glutamic acid, few other residues interact with NAD(P)^+^ cofactor, which is essential for the catalytic activity^10,15^.

Previous studies showed that AtALDH3I1 and AtALDH7B4 considerably minimized lipid peroxidation and provided salinity and drought tolerance to transgenic *Arabidopsis* plants^4^. In rice, *OsALDH*2-4, *OsALDH*3-4, *OsALDH*7, *OsALDH*18-2*,* and *OsALDH*12 genes had been reported to express more than two folds in response to drought stress^7^. The involvement of the maize *ALDH22A1* gene had been found in the tolerance of salinity, dehydration, and ABA treatment^15^. To date, *ALDH* genes have been discovered and analyzed in various plant species, such as *Arabidopsis*^16^, rice^7^, grape^17^, soybean^10^, maize^15^, tomato^14^, Populus^18^, *Brassica rapa*^8^. However, the genome-wide analysis and functional characterization of *ALDH* genes have not been elucidated in potato yet.

Potato (*Solanum tuberosum* L.) is globally the third most significant food crop after rice and wheat and is considered as the most extensively grown tuber food crop^19^. Potato belongs to the Solanaceae family, which has 12 chromosomes with proximately 840 Mb of medium size genome^20^. The primary obstruction of potato yielding is drought, salinity, and high temperature, which significantly cause loss of production worldwide^21^. The availability of the complete genome sequence of potato^22^ creates an opportunity to explore stress-responsive gene families that could provide tolerance against environmental stresses. Therefore, a detailed comparative genome-wide analysis of the ALDH gene family has been conducted in potato in the current study. We have identified a total of 22 ALDH members in potato, distributed in 10 families. Detailed of each member including their physicochemical properties, genomic organizations, presence of conserved motifs and domains, structural organization, sub-cellular localization, evolutionary relationship, expression pattern, and 3D structure were investigated. Further, the expression of all the identified *StALDH* members was analyzed in thirteen anatomical tissues and response to various abiotic, biotic, and hormonal treatments using publicly available mRNA seq data. Transcript profiling of few selected *StALDH* members was validated by quantitative RT-PCR in response to three devastating abiotic stress conditions. Additionally, structure-functional features of the newly identified StALDH family 2 members were analyzed using molecular docking study. These investigations and expression profiling will help to understand the role of *StALDH* genes and create the basis of further functional analysis in other plant species.

## Results

### Genome-wide analysis of potato identifies 22 putative ALDH members

**Figure 1:**
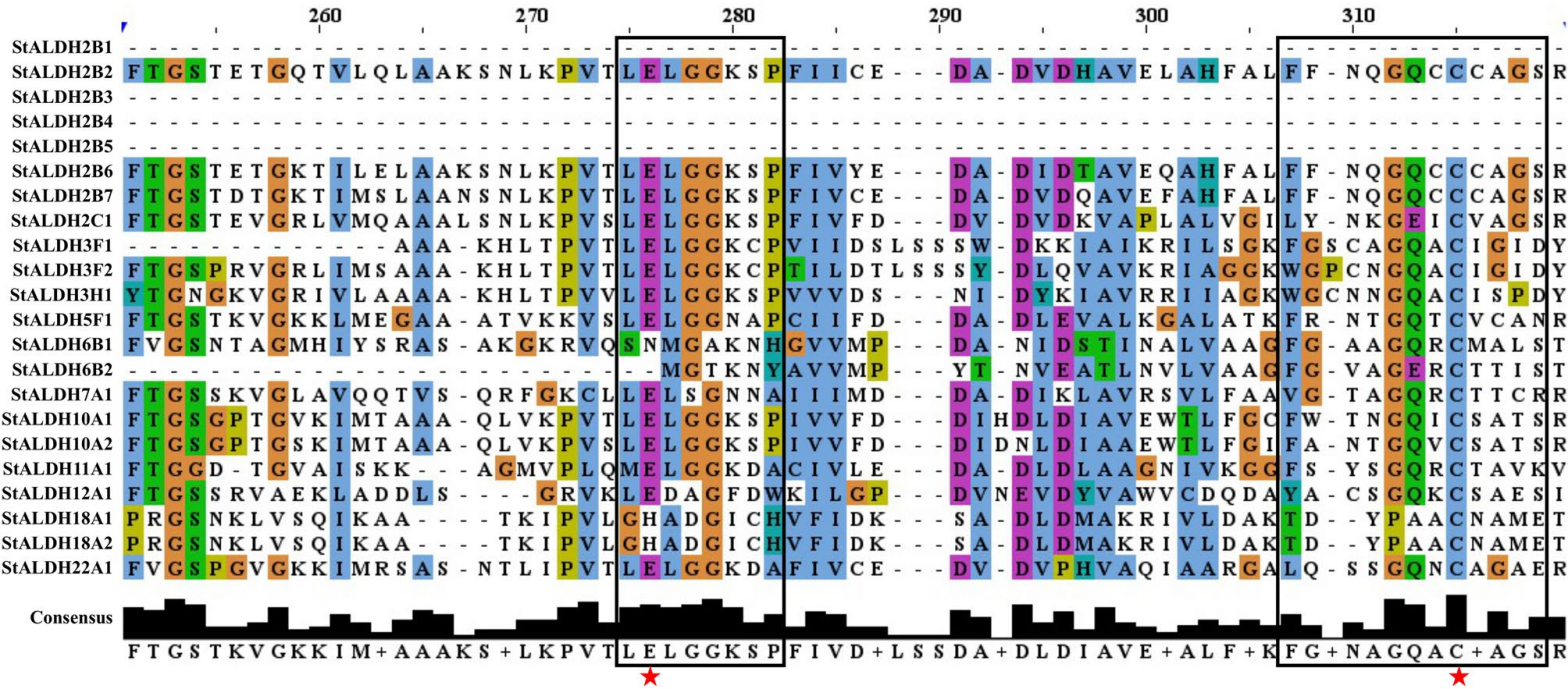
Sequence alignment of the ALDH domain of all the identified StALDH proteins. The ALDH conserved domain (PF00171) of all the putative StALDH proteins were analyzed to identify the conserved active site residues. The boxes represent the conserve motifs for ALDH proteins, and the star indicates the conserved active site glutamate and cysteine residues.

A total of 22 putative ALDH proteins were identified in *S. tuberosum* based on homology search in the Solanaceae Genomics Network (https://solgenomics.net/) (Appendix 1 and 2). NCBI Conserved Domain Database and Pfam analyses confirmed the presence of conserved ALDH domain (PF00171) in all the identified candidates, which is the fundamental property of the ALDH superfamily (Fig. S1). Analysis with PROSITE and multiple sequence alignment (Fig. 1) confirmed the appearance of conserved cysteine active site (PS00070) and glutamic active site (PS00687) in most of the StALDH proteins. 14 out of the total 22 (StALDH2B2, StALDH2B6, StALDH2B7, StALDH2C1, StALDH3F1, StALDH3F2, StALDH3H1, StALDH5F1, StALDH7A1, STALDH10A1, StALDH10A2, StALDH11A1, StALDH12A1, and StALDH22A1) proteins have both the cysteine and glutamic acid active site residues; while StALDH6B1, StALDH6B2, StALDH18A1, and StALDH18A2 proteins have only cysteine active site: and the remaining four (StALDH2B1, StALDH2B3, StALDH2B4, and StALDH2B5) proteins contain no conserved active site in the domain structure (Fig. 1 and Table S1). The catalytic glutamic acid residue functions as a general base in the hydrolytic ALDHs^23^, thus the absence of glutamic acid as well as cysteine residues in the four hydrolytic ALDHs analyzed sequence might be being an incomplete sequence. Similarly, ALDH6 and ALDH18 enzymes do not possess the catalytic glutamate residue because they contain Coenzyme A (CoA) dependent acylating and Δ-1-pyrroline-5-carboxylate synthetases activity, respectively^12^. All these identified StALDH proteins were grouped into ten families (ALDH2, ALDH3, ALDH5, ALDH6, ALDH7, ALDH10, ALDH11, ALDH12, ALDH18, and ALDH22) and nomenclature based on the established criteria designed by AGNC^11^. Among all the 10 families of StALDH, ALDH2 has the largest number of 8 members, while ALDH5, ALDH7, ALDH11, ALDH12, and ALDH22 has only one member per family (Table S1). ALDH3 has 3 members, while the rest families have 2 members each. The biggest protein, StALDH18A1 is 717 aa in length with a molecular weight of 77.47 kDa, while the smallest protein one, StALDH6B2 is 88 aa in length and 9.35 kDa in size (Table S1). The gene length of *StALDH* varied from a range of 2005 nt (*StALDH2B3*) to 9423 nt (*StALDH18A1*). The predicted isoelectric point (pI) of all the putative StALDH proteins varied from 5.10 (StALDH2C1) to 10.00 (StALDH2B5). Subcellular localization of most of the StALDH proteins was mainly predicted to cytosol and chloroplast, followed by mitochondria, plasma membrane, nucleus, and extracellular space (Table S1).

### All the identified *StALDH* members distributed unevenly in ten different chromosomes

**Figure 2:**
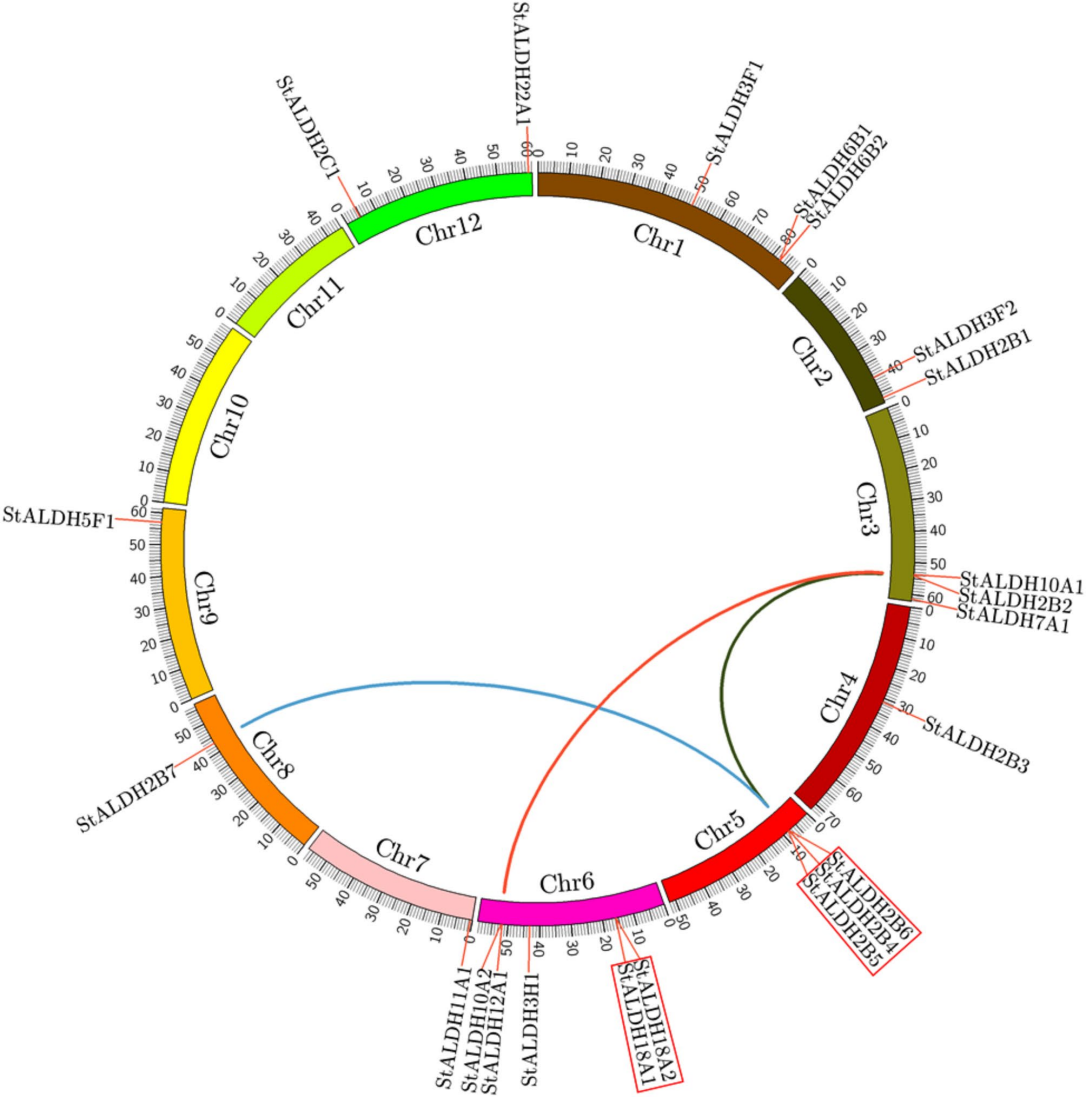
Chromosomal localization and gene duplication of *StALDH* genes. All the identified putative 22 *StALDH* genes were indicated on 12 different chromosomes of potato by red labels. Potato chromosomes (Chr1-Chr12) were depicted with different colour bars generated by Circos software (http://circos.ca/). The scale along with each chromosome indicates their respective genomic size. Three WGD/segmentally duplication gene pairs were connected by red, dark green, and blue lines. Tandem duplication events were indicated by a red box outside the gene names.

Illustration of the chromosomal distribution indicated that all the putative 22 *StALDH* genes are located unequally on 10 out of 12 chromosomes of potato (Fig. 2). Chromosome 6 possesses the maximum number of 5 genes, followed by chromosomes 1, 3, and 5 with three members each. Chromosome 12 contains two *StALDH* genes; followed by chromosomes 2, 4, 8, and 9 contain a single *ALDH* gene per chromosome. There was no *StALDH* gene in Chromosome 10 and 11. The expansion of *StALDH* gene families could be justified through gene duplication analysis^24^ (Fig. 2 and Table S2). Three tandem duplication gene pairs (*StALDH2B4*|*StALDH2B5*, *StALDH2B5*|*StALDH2B6*, and *StALDH18A1*|*StALDH18A2*) and three whole genome duplication (WGD)/segmental duplication events (*StALDH2B2*| *StALDH2B6*, *StALDH2B6*|*StALDH2B7*, and *StALDH10A1*|*StALDH10A2*) were identified (Table S2). All the duplicated gene pairs were under negative purifying selection pressure with a d_N_/d_S_ value of less than 1. Based on the value of d_S_, the duplication event of *StALDH18A1*|*StALDH18A2* was more recent of 0.4 Mya and the duplication event between StALDH18A1 and StALDH18A2 might relate to the most ancient genome duplication (61.7 Mya) as compared with the other events of 10.8, 16, 18.8, 25.8 Mya.

### Exon–intron structure, conserved domain, and motif analysis of StALDHs

**Figure 3:**
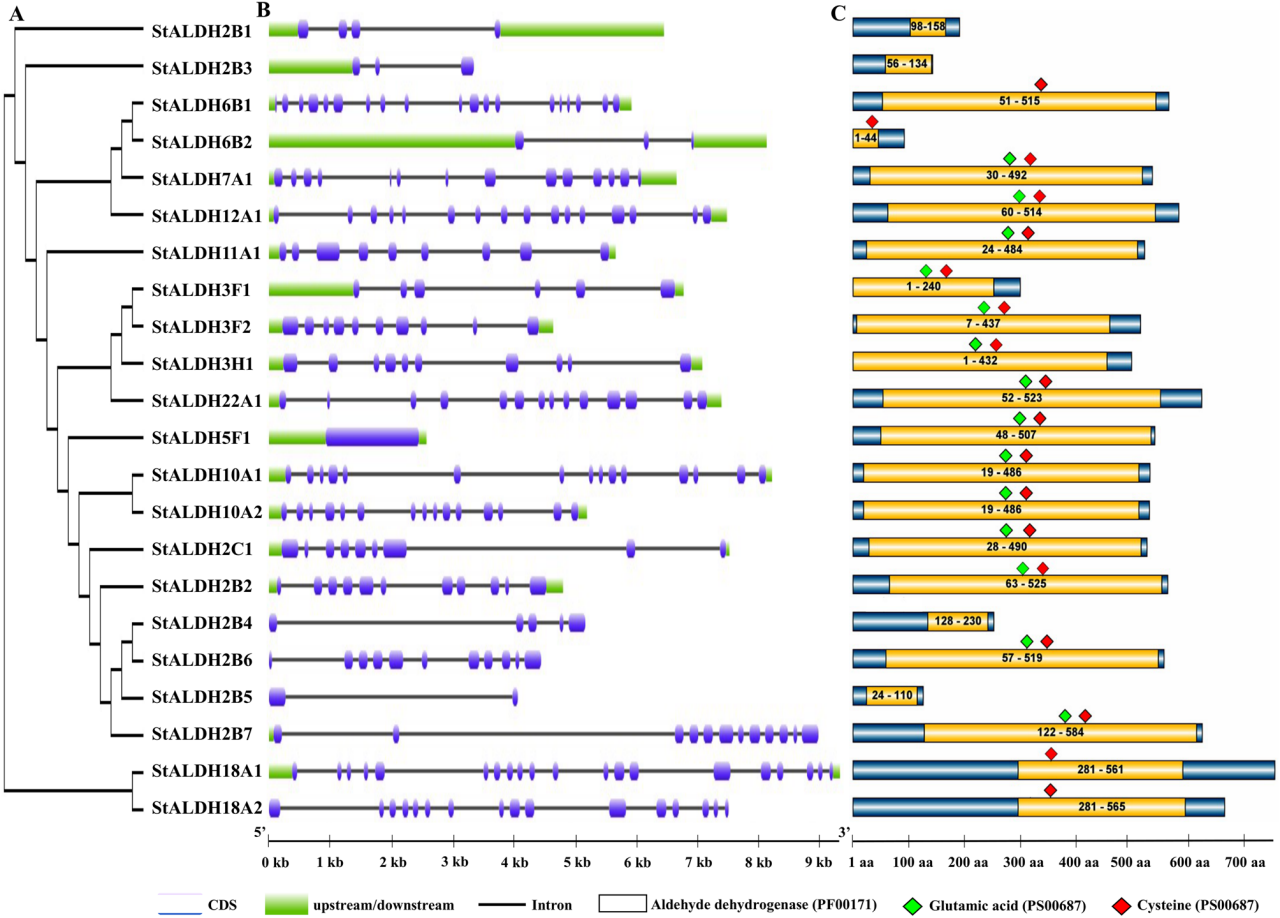
Structural organization of potato ALDH members. (**A**) The phylogenetic tree was generated using MEGA X. (**B**) Exon–intron structures of the putative *StALDH* genes. The graphic representation of the identified *StALDH* gene models generated using GSDS where the blue-colour rounded rectangle, green rectangle, and grey line represented exon, upstream/downstream, and intron, respectively. (**C**) The distribution of conserved aldehyde dehydrogenase domain (PF00171) and active site residues in potato ALDH proteins. The relative positions of each domain within each protein were shown in red boxes. The presence of glutamic acid and cysteine active sites were indicated by lime green and red diamond, respectively. Note: Figures were generated by Microsoft PowerPoint 2010 (https://www.microsoft.com/en-us/download/office.aspx).

The expansion of ALDH family members in potato was further explored by generating an unrooted phylogenetic tree (Fig. 3A). Each class of StALDH members are clustered together to form a separate clade except StALDH2B1 and StALDH2B3 due to their partial sequence (Fig. 3A). This indicates the separation of individual ALDH classes took place before the species-specific expansion. The transcript structure of each *StALDH* member was illustrated by comparing their coding DNA sequences with the respective genomic DNA sequences using the Gene Structure Display Server (http://gsds.cbi.pku.edu.cn/index.php). Enormous dissimilarities had been observed among the gene structures of 22 *StALDH* transcripts (Fig. 3B). The length of genomic DNA sequences differed from 2005 bp (*StALDH2B3*) to 9423 bp (*StALDH18A1*). We found that the number of exons differed from 1 to 19 in *StALDH* transcripts. The size of the exon also differed in different *StALDHs*; the largest exon was found in *StALDH5F1.* Some genes possessed the same amount of exon, although almost all the families showed gain or loss of exons within their members. Among the *StALDH* genes, the highest number of exons was identified in *StALDH6B1* (19 exons) followed by *StALDH12A1* and *StALDH18A2* (16 exons), while only a single exon was identified in *StALDH2B1*, *StALDH3F1*, *StALDH3H1*, and *StALDH5F1.* Consequently, the intron number varied from 0 to 18 in *StALDHs*. There was no intron found in *StALDH2B1*, *StALDH3F1*, *StALDH3H1*, and *StALDH5F1*, and the highest number of introns was found in *StALDH6B1* (18 introns). Transcript structure also revealed that almost all the *StALDHs* have upstream/downstream regions, excluding *StALDH2B4*, *StALDH2B5*, *StALDH2B6, StALDH18A2*.

All the 22 StALDH proteins were investigated using Pfam to identify the existence of conserved ALDH domain—PF00171 in them (Figs. 1 and 3C). Our analyses revealed that all the 22 *StALDHs* have a single ALDH domain (PF00171). Domain analyses revealed that StALDH22A1 contained the largest size ALDH domain (472 aa) followed by StALDH18A1 and StALDH18A2 (468 aa), while StALDH6B2 contained the smallest domain of 44 aa long (Fig. 3C). Ten highly conserved motifs of more than 10 amino acids in length were identified among the 22 StALDH proteins using the online MEME motif search tool (Fig. S2 and Table S3). Almost all the *StALDH* genes contain at least one conserved motif except StALDH2B3 and StALDH6B2. StALDH2B2, StALDH2B6, StALDH2B7, StALDH2C1, StALDH10A1, and StALDH10A2 contain all the 10 conserved motifs (Fig. S2). Among the identified motifs, motif-1 and -9 were found in the maximum of 15 sites, followed by motif-3 and -5 in the 14 sites, while motif-7 and -8 were found only in 8 sites (Table S3). Several of the identified genes/proteins were appeared to be incomplete/truncated with very low protein length (less than 300 aa), molecular mass of less than 32 kDa and shorter conserved ALDH domain. This suggests that either these proteins might be non-functional or the product of pseudo-genes. Although few of them showed significant tissue-specific expression (Fig. S3), six out of the identified 22 members (StALDH2B1, StALDH2B3, StALDH2B4, StALDH2B5, StALDH3F1, and StALDH6B2) were not included for the expression analysis.

### StALDHs share a common core of plant ALDH family

**Figure 4:**
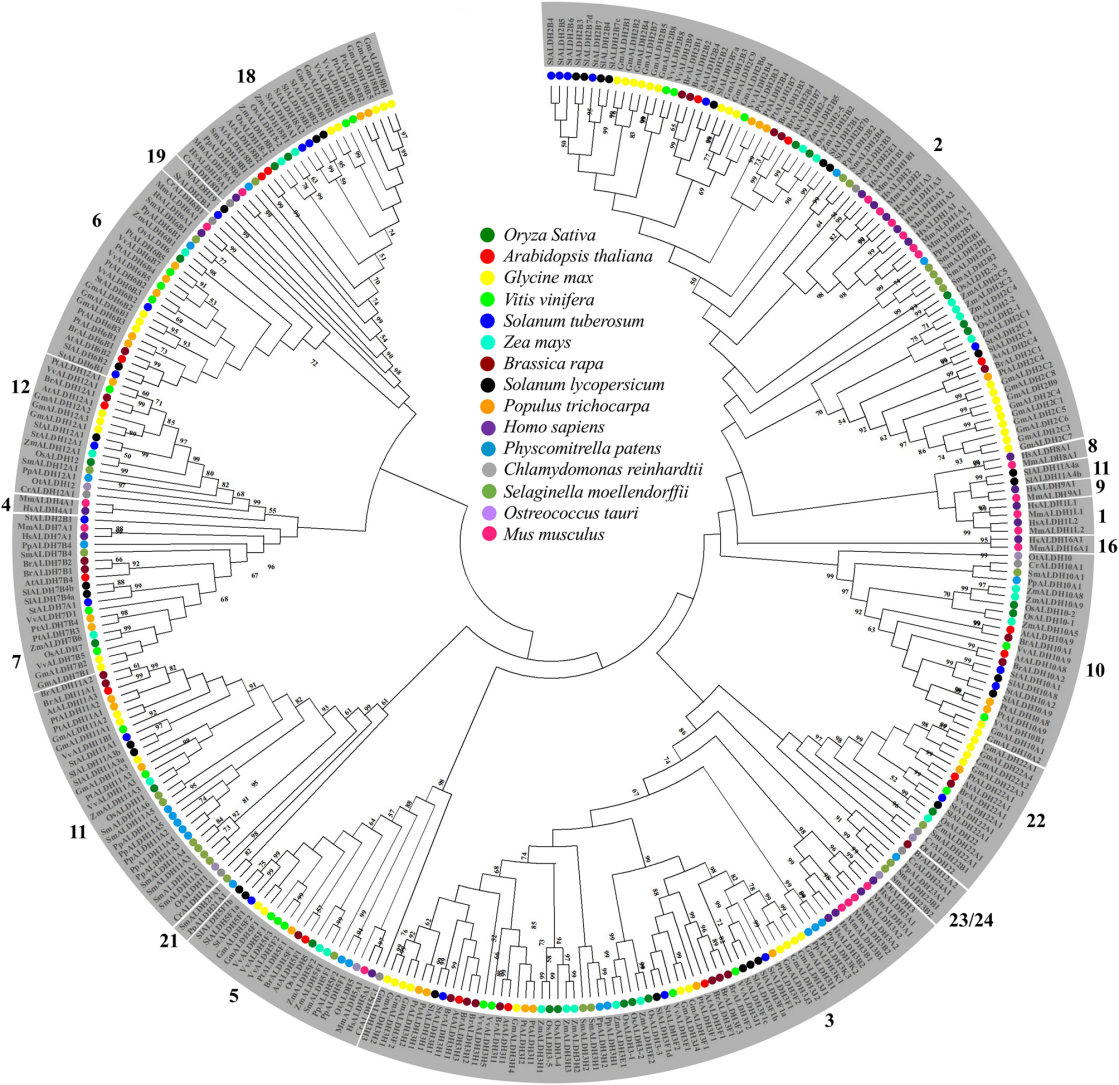
Phylogenetic analysis of potato ALDH members. ALDH proteins from various species including *Arabidopsis*, rice, soybean, maize, field mustard, grape, potato, tomato, black cottonwood, moss, green algae, mouse, and human were collected from databases. A total of 335 protein sequences from 12 different species were aligned by ClustalW followed by the construction of a maximum-likelihood tree using MEGA X (https://www.megasoftware.net/) with 1000 bootstrap replicates. Bootstrap values greater than 50 were shown in the different branching points of the tree indicating significant clustering. ALDH members from different species were indicated by a different colour and summarized in the middle of the tree. The tree was divided into 11 families based on their clustering pattern and individual ALDH family number was mentioned.

To investigate the sequence resemblances and evolutionary relationship among ALDHs, a phylogenetic relationship was established among the well-studied dicots like *Arabidopsis*, *Solanum lycopersicum, Glycine max, Vitis vinifera*, *Brassica rapa*, *Populus trichocarpa*; and monocots including *Oryza sativa* and *Zea mays*; two mosses (*Physcomitrella patens* and *Selaginella moellendorffii*); two algae (*Chlamydomonas reinhardtii* and *Ostreococcus tauri*); and two mammals (*Homo sapiens* and *Mus musculus*) ALDH members. A maximum-likelihood phylogenetic tree was constructed using 335 protein sequences from all the above-mentioned species (Fig. 4). Our investigation revealed that all these ALDH proteins from various species grouped in 19 families (ALDH-1, 2, 3, 4, 5, 6, 7, 8, 9, 10, 11, 12, 16, 18, 19, 21, 22, 23 and 24). Interestingly, most of the proteins from the same family were clustered together irrespective of the source/type of organism. Most of the StALDH proteins shared a common core of plant ALDH families and mainly were distributed in 10 major plant specific families and one minor (SlALDH19) subgroup only present in tomato. Three ALDH subgroups (21, 23 and 24) were observed only from the sequences of lower plant Algae and Mosses. However, non-pant animals including human and mouse form five distinguished subfamilies such as ALDH-1, 4, 8, 9 and 16. Truncated / pseudo StALDH members (StALDH2B1, StALDH2B3, StALDH2B4, StALDH2B5, StALDH3F1, and StALDH6B2) showed inconsistency in forming the clade due to their short sequences. Distinct clades comprised of members from family- 2, 3, 5, 6, 18 and 22- were observed across the evolutionary diverse species (Fig. 4 and Table 1). ALDH family—2 and 3 formed the biggest clusters indicating their abundance in all the studied species, while family-5, 12 and 22 had the lowest number of members (Fig. 4). Overall, our observation revealed that the evolution of plant *ALDH* genes happened before the separation of monocot—rice, maize; and dicot—*Arabidopsis*, soybean, tomato, mustard, grape, and potato as all the ALDH subfamily members were found to be clustered together from both monocot and dicot plants in the phylogenetic tree (Fig. 4).

**Table 1:** Comparative identification of the *ALDH* gene families in various organisms.

Species	Common Name	Genome size (Mbp)	ALDH family																			Total ALDH		References	
			1	2	3	4	5	6	7	8	9	10	11	12	16	18	19	21	22	23	24				
*Arabidopsis thaliana*	Thale cress	135	–	3	3	–	1	1	1	–	–	2	1	1	–	2	–	–	1	–	–	16		^4^	
*Solanum tuberosum*	Potato	840	–	8	3	–	1	2	1	–	–	2	1	1	–	2	–	–	1	–	–	22		Current study	
*Brassica rapa*	Field mustard	485	–	5	7	–	1	1	2	–	–	2	2	2	–	–	–	–	1	–	–	23		^8^	
*Vitis vinifera*	Grape	300	–	3	4	–	3	3	2	–	–	2	2	1	–	2	–	–	1	–	–	23		^17^	
*Oryza Sativa*	Rice	372	–	5	5	–	1	1	1	–	–	2	1	1	–	2	–	–	1	–	–	20		^7^	
*Zea mays*	Maize	3,000	–	6	5	–	2	1	1	–	–	2	1	1	–	2	–	–	1	–	–	22		^15^	
*Glycine max*	Soybean	1,150	–	18	11	–	2	3	2	–	–	2	3	4	–	5	–	–	4	–	–	53		^9^	
*Gossypium raimondi*	Cotton	880	–	8	6	–	1	3	1	–	–	2	3	1	–	4	–	–	1	–	–	30		^60^	
*Solanum lycopersicum*	Tomato	950	–	8	5	–	2	1	2	–	–	2	4	1	–	2	1	–	1	–	–	29		^14^	
*Sorghum bicolor*	Sorghum	660	–	5	4	–	1	1	1	–	–	2	1	1	–	2	–	–	1	–	–	19		^12^	
*Senna italica*	Foxtail millet	515	–	6	4		1	1	1			2	1	1		2			1			20		^17^	
*Populus trichocarpa*	Black cottonwood	403	–	4	6	–	1	4	2	–	–	2	3	1	–	2	–	–	1	–	–	26		^12^	
*Malus domestica*	Apple	750	–	13	7	–	2	2	2	–	–	2	3	2	–	4	–	–	2	–	–	39		^34^	
*Chlamydomonas reinhardtii*	Unicellular green algae	112	–	1	–	–	1	1	–	–	–	1	1	1	–	1	–	–	1	–	1	9		^13^	
*Ostreococcus tauri*	Marine green algae	12	–	–	1	–	1	–	–	–	–	1	1	1	–	–	–	–	1	–	–	6		^13^	
*Physcomitrium patens*	Moss	480	–	2	5	–	2	1	1	–	–	1	5	1	–	1	–	1		1	–	21		^13^	
*Selaginella moellendorffii*	Gemmiferous Spikemoss	100	–	6	2	–	1	1	1	–	–	1	6	1	–	1	–	1	1	2	–	24		^12^	
*Homo Sapiens*	Human	3,000	6	1	4	1	1	1	1	1	1	–	–	–	1	1	–	–	–	–	–	19		^12^	
*Mus musculus*	House mouse	2,600	7	1	4	1	1	1	1	1	1	–	–	–	1	1	–	–	–	–	–	20		^17^	

### Expression of *StALDH* genes is abundant in fruits

**Figure 5:**
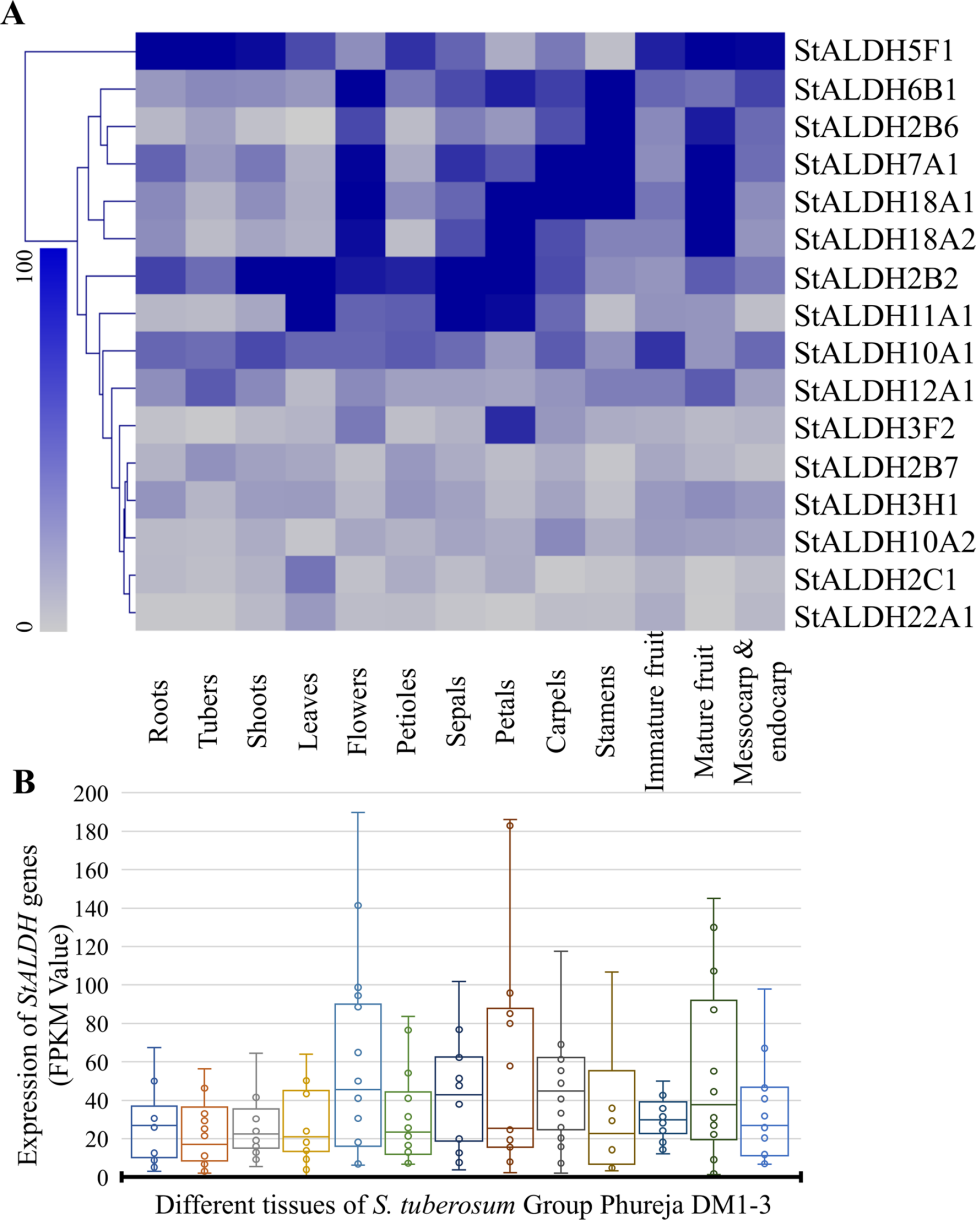
Expression profiles of *StALDH*s at different tissues. Transcript abundance of 16 *StALDH* genes in thirteen different tissues (roots, tubers, shoots, leaves, flowers, petioles, sepals, petals, carpels, stamens, immature fruits, mature fruit and inside of fruit) was analyzed. (**A**) The RNA-seq data was retrieved from Potato Genome Sequencing Consortium (PGSC). The Fragments Per Kilobase of transcript per Million mapped reads (FPKM) values were normalized and the heatmap was generated with a hierarchical clustering of Manhattan distance correlation using MeV 4.9 software (http://mev.tm4.org/). The colour bar represents relative expression values, where the increase in the intensities of blue colour represents the level of expression. (**B**) The box and whisker scatter plot illustrate the FPKM value of each gene at each tissue. The tissue name in 4Acorrespondsd to the boxes in 4B.

The expression profile of all the identified *StALDH* genes was analyzed in thirteen different tissues including roots, tubers, shoots, leaves, flowers, petioles, sepals, petals, carpels, stamens, immature fruits, mature fruit and inside of fruit (mesocarp & endocarp). Various *StALDH* genes exhibited differential tissue-specific expression patterns. Amidst all the 16 *StALDH* genes, *StALDH*2B2 exhibited the maximum level of expression in almost all the considered tissues except tuber, carpel, stamen, and fruits where *StALDH*5F1 exhibited maximum expression (Fig. 5). Some of the members of the *StALDH* gene family display highly tissue-specific expression; for example, the expression of *StALDH3F*2 is petal specific, and *StALDH2C*1 is leaf-specific (Fig. 5A). All these tissue-specific genes do not show a high level of expression in other tissues or organs. Some of the *StALDH* genes exhibited high expression in multiple tissues, such as *StALDH5F1* showed high expression in root, tuber, and shoot; *StALDH6B1* showed high expression in flower, petal, and stamen; *StALDH7A1* showed high expression in root, flower, carpel, and stamen; and *StALDH11A1* found to be highly expressed in leaf, sepal, and petal. Surprisingly, some of the genes showed a very low level of expression in all the tissues, such as *StALDH3H1*, *StALDH10A2*, *StALDH3F1*, and *StALDH22A1*. Global gene expression analysis in various tissues revealed that *StALDH* genes were abundant in fruit (immature, mature, and mesocarp & endocarp), flowers, and carpels with a median FPKM more than 25, two-fold higher than that in the root (Fig. 5B). Moreover, *StALDH5F*1 showed a remarkably higher level of expression in most of the tissues with a median FPKM of 76, followed by *StALDH2B*2 with a median FPKM of 67 in different tissues.

### *StALDH* transcripts showed differential expression pattern in response to various abiotic and biotic stress elicitors, and hormonal treatments

**Figure 6:**
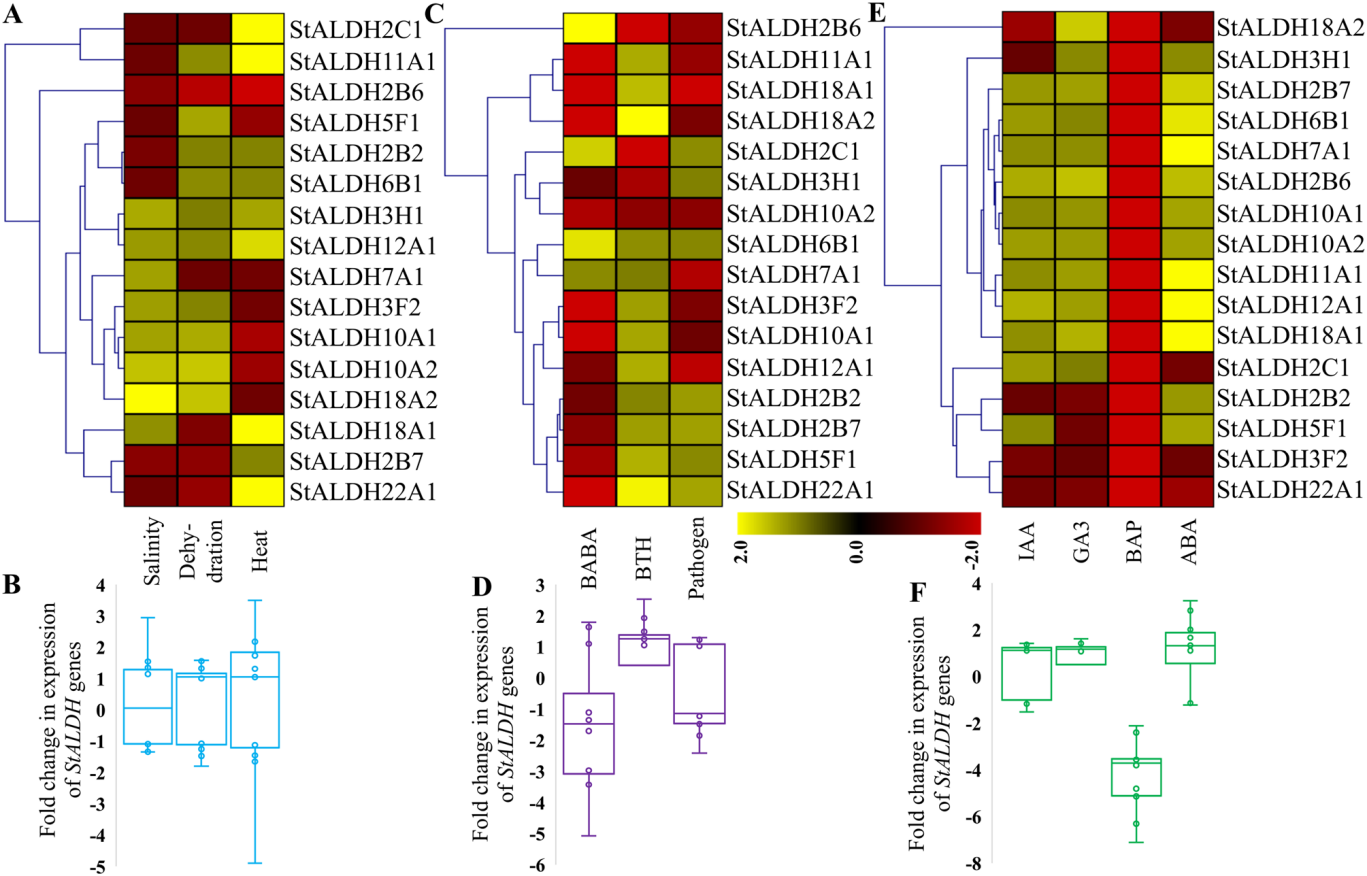
Expression analysis of *StALDH* genes upon environmental unfavorable conditions. The expression pattern of the 16 *StALDH* genes was investigated under various (**A**,**B**) Abiotic stresses, (**C**,**D**) Biotic elicitors, and (**E**,**F**) Hormonal treatments. Expression data were obtained from PGSC for three abiotic stresses such as salinity, dehydration, and heat; three biotic elicitors such as BABA, BTH, and pathogen; four hormonal treatments such as IAA, GA3, BAP, and ABA; and compared with their respective control samples to calculate the fold change in expression. Heatmap was created by hierarchical clustering of Manhattan distance correlation using MeV 4.9 software (http://mev.tm4.org/). Colour scale represents fold changes, where yellow colour indicated upregulation and red colour indicated downregulation of individual gene. The box and whisker scatter plots below heatmaps showed the median fold change values of each gene.

To have a better understanding of the function of *StALDH* genes under abiotic stresses, we analyzed their transcript profiling in response to three abiotic stress conditions viz. salinity, dehydration, and heat (Fig. 6A). Among the 16 *StALDH* genes, *StALDH3H1* and *StALDH12A*1 were found to be highly upregulated in all three abiotic stress conditions. A cluster of genes *StALDH3F*2, *StALDH10A*1, *StALDH10A*2, and *StALDH18A*2 showed a medium to a high level of upregulation in response to salinity and dehydration stresses. Besides this cluster, *StALDH11A*1 showed the highest level of upregulation in response to salt, dehydration, and heat, respectively. Transcripts of *StALDH11A*1 showed an upregulation of more than 10 folds, respectively in response to salt, dehydration, and heat. Based on the median fold change in expression of all *StALDH* genes, the total transcript abundance of *StALDH* genes was mostly upregulated in response to dehydration stress, while mostly downregulated in response to heat stress (Fig. 6B).

For the biotic stress responsiveness of *StALDHs*, the expression pattern was observed responding to β-aminobutyric acid (BABA) and benzothiadiazole (BTH) and pathogen treatment (Fig. 6C). *StALDH6B*1 is the only member showing upregulation in all three biotic stress conditions. A clade of *StALDH2B*2, *StALDH2B*7, *StALDH5F*1, and *StALDH22A*1 were upregulated in both BTH and pathogen treatment. Some genes show upregulation, in particular, one type of stress treatment; *StALDH2B*6 and *StALDH2C*1 showed upregulation only in response to BABA treatment; *StALDH11A*1, *StALDH18A*1, *StALDH18A*2, *StALDH3F*2, *StALDH10A*1, and *StALDH12A*1 showed upregulation only in response to BTH treatment. Transcript of *StALDH3H1* showing upregulation only in response to pathogen treatment. In response to BABA treatment, *StALDH2B6* showed the highest upregulation of more than 4 folds changes. *StALDH10A2* showed downregulation in all three biotic stress elicitors. However, the median fold change in expression of all *StALDH* genes showed mostly upregulation for BTH induction, while downregulation in response to BABA and pathogens (Fig. 6D).

Phytohormones play crucial roles in coordinating regulatory networks and the signal transduction pathways associated with external stimuli^25^. The expression pattern was analyzed in response to different hormonal treatments, such as 6-benzyl amino purine (BAP), indole-3-acetic acid (IAA), abscisic acid (ABA), gibberellic acid (GA3) (Fig. 6E). Surprisingly, all the 22 *StALDH* genes showed downregulation in response to BAP treatment. A clade of *StALDH2B*7, *StALDH6B*1, *StALDH7A*1, *StALDH2B*6, *StALDH10A*1, *StALDH10A*2, *StALDH11A*1, *StALDH12A*1, and *StALDH18A*1 were found to be upregulated in response to other three (IAA, ABA, and GA3) hormone treatments. A cluster of two genes *StALDH3F*2 and *StALDH22A*1) showed downregulation in response to all four hormonal treatments. The total transcript abundance of *StALDH* genes was extremely low in BAP treatment as observed previously in the case of *StTPS*s^26^, while the rest of the three hormone treatments bought upregulation (Fig. 6F).

### Abiotic stress-responsiveness of selected *StALDH* genes were verified using qRT-PCR

**Figure 7:**
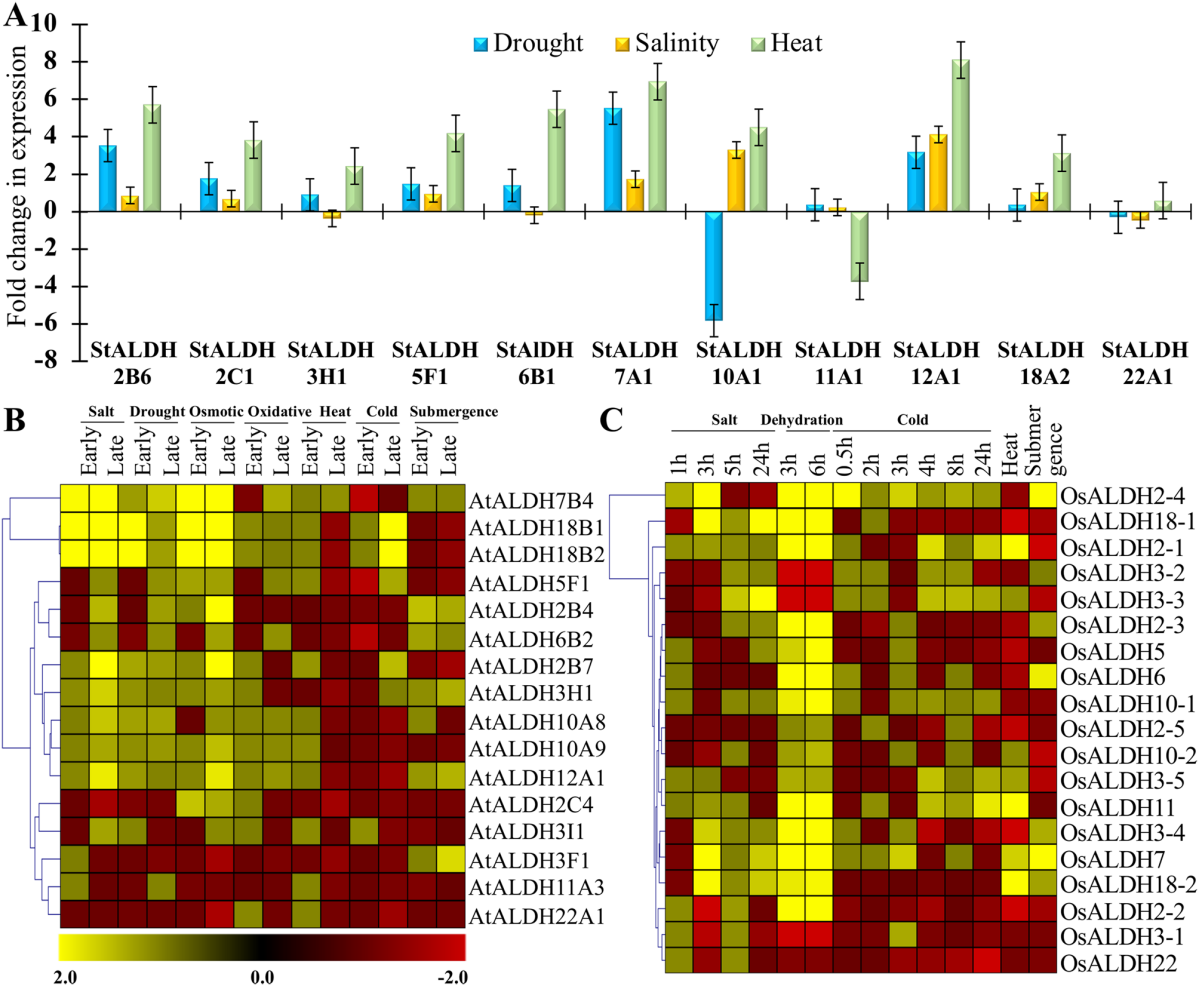
Expression analysis of *ALDH* superfamily members in response to abiotic stress. (**A**) The expression pattern of 11 selected *StALDH* genes was investigated in response to three abiotic stresses such as salinity, dehydration, and heat using qRT-PCR. The average fold change in expression as compared to their respective control was represented in the bar diagram (n = 3). Heatmaps with hierarchical clustering of Manhattan distance correlation of the transcript alteration of (**B**) *AtALDH* and (**C**) *OsALDH* superfamily members were generated using MeV 4.9 software (http://mev.tm4.org/). The Colour scale represents the fold changes, where yellow colour indicated upregulation and red colour indicated downregulation of individual gene.

Global gene expression analysis of *StALDH* in response to various stress conditions revealed that *StALDH* transcripts are regulated/altered differently depending on the type of environmental stimuli. The differential expression of genes was verified in response to salt (NaCl), drought (Mannitol) and heat stress. Quantitative RT-PCR was performed for highly stress-inducible 11 selected *StALDH* genes (*StALDH*-2B6, 2C1, 3H1, 5F1, 6B1, 7A1, 10A1, 11A1, 12A1, 18A2 and 22A1). Data analysis revealed that most of the genes showed upregulation in response to all three treatments (Fig. 7A). Only *StALDH*10A1 and *StALDH*11A1, showed significant down-regulation in response to drought and heat treatments, respectively. Transcript of *StALDH*12A1 was highly in all three conditions followed by *StALDH*7A1 and *StALDH*2B6. The results suggested that *StALDH* members showed frequent stress-induced upregulation in their transcript level.

To verify this finding, we have analyzed the expression pattern of ALDH members from two widely studied model plants- *Arabidopsis* and rice. Most of the *AtALDH* members showed upregulation in response to salinity, drought, and osmotic stresses, while fluctuation of temperature (either cold or heat) mostly resulted in downregulation (Fig. 7B). Similarly, the expression of *OsALDH* transcripts was highly upregulated in response to different degree of dehydration stress (Fig. 7C), followed by salinity and cold stresses. Overall, the abiotic stress-induced transcript alteration of *ALDH* superfamily members was found to be evolutionarily conserved in both monocot and dicotyledons plant species.

### Promoters of *StALDH* genes contain various abiotic and hormone-responsive *cis-elements*

*Cis*-regulatory elements are crucial factors to influence gene expression and regulation^27^. In total, 22 *cis*-regulatory elements including 16 abiotic stress and 6 phytohormone responsive elements were identified in the putative promoter region of *StALDH* genes (Fig. S4). The appearance of various hormonal responsive elements (ABRE, ARE, GARE motif, TC rich elements, TCT motifs, and TGA elements) on *StALDH* promoters revealed the feasible impact of different hormones, for instance, abscisic acid, gibberellin, auxin, jasmonic acid, and salicylic acid on the expression of *StALDH* genes. Our analysis revealed that promoter of *StALDH2B4* and *StALDH3H1* comprised the maximum number of 5 phytohormone responsive elements. Among the hormone-responsive *cis*-elements, ABRE is present in the promoter region of 14 out of 22 *StALDH* genes that indicate potato ALDH genes associated with ABRE elements have a significant role in drought stress. Furthermore, abiotic stress-responsive elements such as AE-box, ACE, Box 4, ERE, Gap-box, GATA-motif, GATT-motif, G-box, LTR, MBS, MRE, O_2_-site, P-box, Sp1, TCA-element, and I-box were also found to be present on *StALDH* promoters. Among the abiotic stress-responsive elements, light-responsive Box-4 and G-box were identified in most of the promoters. Overall, the promoter of *StALDH2B4* comprises the maximum number of 11 *cis*-regulatory elements, followed by *StALDH2B3* and *StALDH22A1* with 10 *cis*-regulatory elements, while *StALDH2B7* and *StALDH6B2* promoters comprised the lowest number of only three elements.

### Homology modelling of representative StALDH proteins

**Figure 8:**
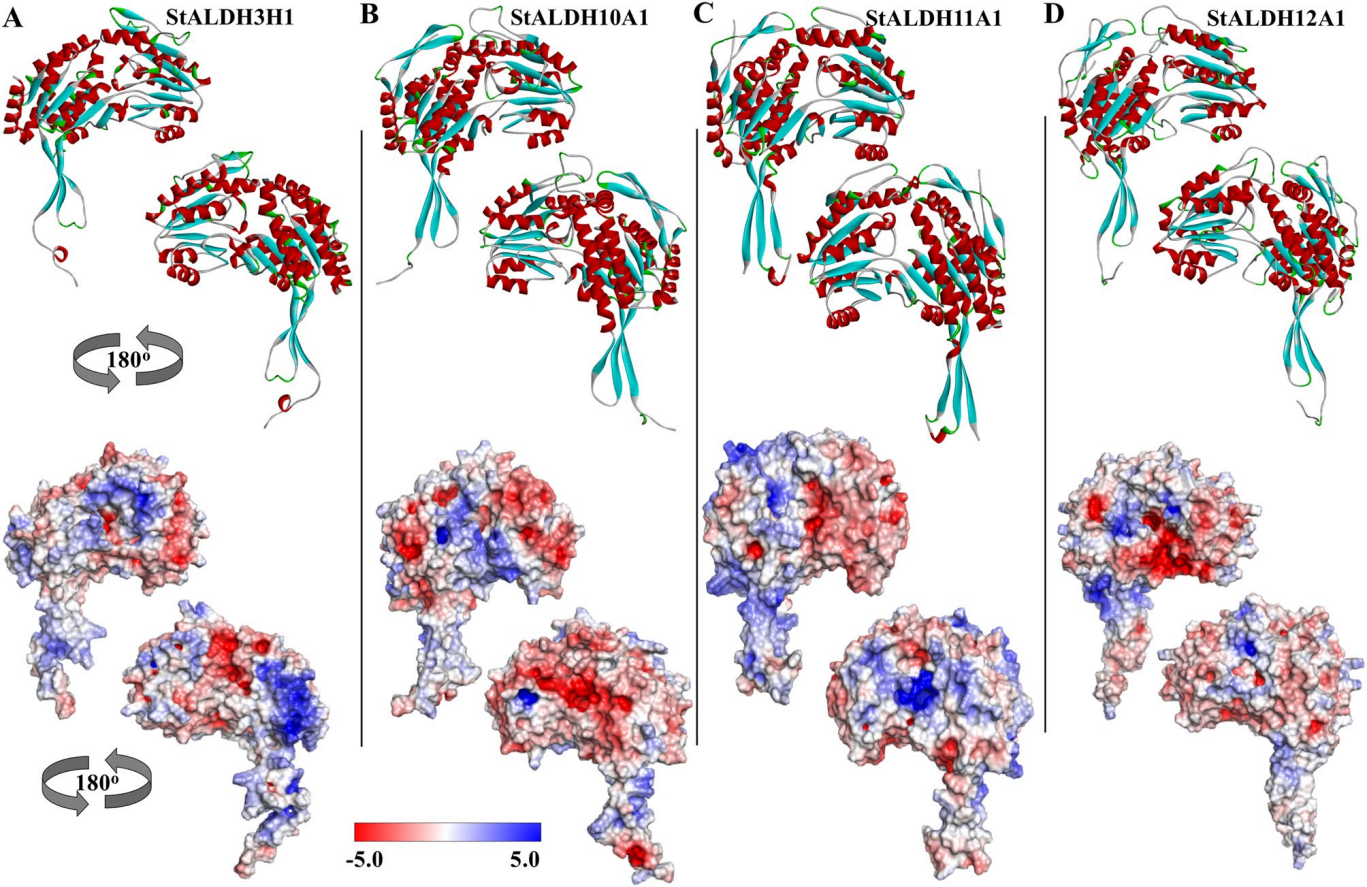
Three-dimensional structure analysis of the selected StALDH proteins. Four highly abiotic stress-specific upregulated proteins (**A**) StALDH3H1, (**B**) StALDH10A1, (**C**) StALDH11A1, and (**D**) StALDH12A1 were selected for homology modelling. All the structures were depicted as cartoon diagram for the structure analysis of each protein in two different views (rotated 180°). The electrostatic surface potential of each selected proteins was illustrated to show charge distribution in the surface of the proteins. The surface colours were clamped at red (− 5) and blue (+ 5). Red and blue colour indicated negatively and positively charged amino acids, respectively. All the structures were visualized and generated using Discovery Studio Visualizer 2016 software (https://discover.3ds.com/discovery-studio-visualizer-download).

Self-optimized prediction method with alignment (SOPMA) predicted the presence of alternate ratio of alpha helices, extended strands, beta turns, and coils in the different StALDH protein structures (Table S4). The presence of alpha-helix ranging from 29.12 to 56.67% dominates the other form in the secondary structure prediction, followed by random coil (30.00–43.68%), extended strand (9.17–20.88%) and finally beta-turn (4.15–11.36%). Protein glycosylation is another important aspect of protein structure that regulates a wide range of biological processes such as protein folding, signalling, stability, conformation, and cell–cell interactions^28^. Glycosylation analysis predicted that 12 out of 16 analyzed StALDH proteins have potential N-glycosylation sites, among them StALDH12A1, StALDH18A1, and StALDH18A2 have the highest number 3N-glycosylation sites (Table S5). To know the structural arrangement and 3-D coordination, four abiotic stress-responsive proteins- StALDH3H1, StALDH10A1, StALDH11A1, and StALDH12A1 were selected for homology modelling using the template of *Rattus norvegicus* ALDH (PDB: 1AD3), *Solanum lycopersicum* ALDH (PDB: 4I9B), *Streptococcus mutans* ALDH (PDB: 1EUH), and *Zea mays* ALDH (PDB: 6D97), respectively (Fig. 8). The generated homology models were validated using MolProbity Ramachandran plot analysis (Fig. S5). Results confirmed the accuracy of 3D modeling as most of the residues of StALDH3H1 (Fig. 8A), StALDH10A1 (Fig. 8B), StALDH11A1 (Fig. 8C), and StALDH12A1 (Fig. 8D) were placed in the favored region of 95.1%, 97.8%, 96.0% and 97.7%, respectively (Fig. S5). The homology model revealed that the overall structure of the four selected proteins was very similar in terms of common strands and helices in the Rossmann folding type (Fig. 8). However, few remarkable dissimilarities were noticed in the length and conformation of the oligomerization site, angle of alpha-helices and beta-sheets, and tail of the N-terminal. StALDH11A1 showed a longer loop in the oligomerization domain and more curvature coil in the catalytic and coenzyme binding site (Fig. 8C) than the other selected proteins. The surface charge distribution of the selected proteins was generated through the Adaptive Poisson-Boltzmann Solver (APBS) package as shown in two surface views rotated 180° (Fig. 8A–D). Different colours depicted in these models indicated different surface properties, where blue representing positive charge, red negative charge, and white neutral charge. Significant dissimilarities have been observed in the positively, and negatively charged amino acid distribution in the surface of those selected proteins.

## Discussion

Potato is a good source of dietary fiber with other essential nutrients and serves as the main food to more than a billion people in over 100 countries^29,30^. Consequently, increasing the yield of potato has a significant role in satisfying the nutritional demands for global population growth^31^. As potato is a stress-sensitive crop, its capability to deter various abiotic and biotic stress is essential for producing as a major food source in near future. The complete genome sequence of potato was made available in 2011^22^. In this study, we have performed a comprehensive investigation of ALDH members in potato to reveal its functional correlation with various abiotic and biotic stress conditions. *ALDH* genes have been identified in both prokaryotic and eukaryotic organisms and specified within almost all plant species^12^. Previously 16, 20, 22, 53, 23, 23, and 29 *ALDH* genes have been identified in *Arabidopsis*^4^, rice^7^, maize^15^, soybean^9^, grape^17^, mustard^8^, and tomato^14^, respectively (Table 1). We have identified 22 *ALDH* genes in the potato (genome size of 840 Mb), which is greater than the number of previously identified smaller genome sized *Arabidopsis* (16) and rice (20), but lower than mustard and grape (23 each). Thus, the number of total ALDH could be directly correlated with their respective genome size (Table 1). Scatter plot with regression analysis showed a significant correlation (R^2^ = 0.6128) between the total identified ALDH gene numbers with their respective genome size on the selected organisms except for *Z. mays*, *H. Sapiens*, and *M. musculus* (Fig. S6).

Plant *ALDH* genes are mainly grouped into 14 families, while only ten ALDH families (Family-2, 3, 5, 6, 7, 10, 11, 12, 18, and 22) have been found in potato consistent with the previously identified *Arabidopsis*, rice, maize, soybean, and grape ALDH family. Interestingly, the presence of ALDH19 has been only reported from tomato plants^14^ and family ALDH-21, 23 and 24 identified only in mosses and algae, to date (Table 1 and Fig. 4). Expansion of a gene family evolved from the process of whole-genome duplication, tandem duplication, or segmental duplication^32^. We have observed both WGD/segmental duplication and tandem duplication events that are involved in the expansion of the potato ALDH gene family. Previously, two tandem duplication events had been recorded in *O. Sativa* (*OsALDH2-1*|*2-2* and *OsALDH3-1*|*3-2*)^7^, *V. vinifera* (*VvALDH5F1*|*5F2*|*5F3* and *VvALDH6B3*|*6B5*)^17^. In our current study, we also found two tandem duplication events (*StALDH2B4*|*StALDH2B5*|*StALDH2B6*, and *StALDH18A1*|*StALDH18A2*). Besides, we have also found three WGD/segmental duplication events that took place approximately 61.7, 25.8, and 18.8 Mya ago. Though 9 out of 22 *StALDH* genes have emerged from the duplication events, it was not always possible to determine their function and expression relying on their common ancestors. Six out of these 22 *StALDH* genes (*StALDH2B1*, *StALDH2B3*, *StALDH2B4*, *StALDH2B5*, *StALDH3F1*, and *StALDH6B2*) have partial/truncated sequence and thus, do not possess the conserved active site residues/ALDH domain (Table S1, and Fig. 1). However, three of them (*StALDH2B3*, *StALDH2B4*, and *StALDH2B5*) showed significant transcript abundance in stamen and mature fruit (Fig. S3). Thus, these genes could be pseudogene, or neo-/sub-functionalized, which need further experiment to confirm. Phylogenetic analysis of *S. tuberosum* ALDH members with other identified plant and non-plant species revealed that members from the same family clustered together. This indicates the fact of the evolution of plant *ALDH* genes took place before the detachment of dicotyledon—*Arabidopsis* grape, soybean, tomato, potato and mustard and monocotyledon—rice, and maize. Moreover, our phylogenetic investigation unveiled that ALDH family-2, 5, 7, and 10 are closely related, whereas family-18 is the most distantly related one (Fig. 4). In addition, ALDH-2 and ALDH-3 are the two most extended families, while ALDH-12 and ALDH-18 are the smallest families in the twelve analyzed species.

Plant *ALDH* genes have a significant role in environmental adaptability and alteration in expression patterns when exposed to a variety of stressors such as dehydration, extreme salinity, heat, oxidative stress, and many others^12,33^. Therefore, expression profiling of different *StALDH* genes reveals their function in different stress conditions. Different members of plant *ALDH* genes have been found to express in different tissues and developmental stages. In an earlier study, *MdALDH3F*1 and *MdALDH10A*8 were found to be highly expressed in the fruit development of apple^34^. The expression level of *VvALDH2B8*, *VvALDH3H5,* and *VvALDH18B1* significantly increased during grape development and ripening^17^. *GmALDH3H2* and *GmALDH3H4* showed a high expression level in the flower of soybean^9^. In *Solanum tuberosum*, a high expression level was observed in fruit (immature, mature, and mesocarp & endocarp), flower, and carpel with a median FPKM of more than 25 (Fig. 5). However, *StALDH2B2* and *StALDH5F1* showed remarkably higher expression in almost all tissues. Thus, our investigation has consistency with the fact that different *ALDH* genes have different expression patterns in a tissue-specific manner. Previously *ALDH* genes were found to be upregulated in drought, salinity, and heat stresses in various organisms. Transcripts of *OsALDH*2-4 and *GmALDH2B*2 genes were highly up-regulated in response to drought stress in rice and soybean, respectively^7,9^; that of *VvALDH2B4* and *VvALDH2B8* have shown up-regulation in response to drought and salinity stress in grape^17^ and *PtALDH3H*4 and *PtALDH6B*4 in black cottonwood were found to be upregulated in response to heat stress^18^. In our study, we have observed an upregulation of 50% (8/16) *StALDHs* in response to salinity stress among them *StALDH18A2* showed the highest upregulation of almost three-fold change. In response to dehydration stress, 62.5% (10/16) genes were upregulated, among them *StALDH10A2 and StALDH18A2* showed the maximum upregulation of 1.5 folds (Fig. 6A). Moreover, the abiotic stress-specific transcript upregulation of *StALDH*12A1, *StALDH*7A1, and *StALDH*2B6 was further confirmed by qRT-PCR analysis in one of the Bangladeshi potato variety (Fig. 7A) and the abiotic stress-specific transcript upregulation found to be evolutionary conserved in Arabidopsis and rice (Fig. 7B,C). Similarly, 56.25% (9/16) *StALDH* genes showed upregulation in response to heat. To the best of our knowledge, the role of ALDH in biotic stress has not been investigated thoroughly. Most of the *StALDH* genes showed upregulation in response to different biotic stress elicitors (Fig. 6C). Among the 16 members, *StALDH6B*1 showed universal upregulation in response to all three biotic stress conditions. Phytohormones played important roles in the ability to respond to various stress condition. Formerly, *AtALDH3I*1 and *AtALDH7B*4 from *Arabidopsis* and *BrALDH12A*1 from *Brassica rapa* have shown significant upregulation in response to ABA treatment. We have observed a similar pattern of upregulation for most of the *StALDH* transcripts in response to ABA, GA3, and ABA treatments. Interestingly, BAP treatment resulted in complete downregulation of all *StALDHs* (Fig. 6E)*.* This result indicates that BAP might be a key negative regulator for ALDH gene transcription, similar to *TPS* genes^26^. Moreover, the presence of various phytohormone and stress-responsive *cis*-acting regulatory elements in the putative *StALDH* promoter regions could be directly correlated with the observed expression profile. The promoter of *ALDH7* genes of different Brassicaceae family contained conserved ACGT-containing motif, dehydration-responsive element (DRE) and C-reactive low temperature-responsive element (CRT) that is induction by salt, dehydration, and ABA in leaves^35^. From the *cis*-regulatory elements analysis, we found that *StALDH*2B2, *StALDH*3H1, *StALDH*5F1, *StALDH*10A1, *StALDH*11A1, *StALDH*12A1, and *StALDH*18A2 contained cis-element which has a critical role in response to drought stress^36^. This result is compatible with our abiotic stress expression findings as these genes are upregulated in response to drought stress condition.

**Figure 9:**
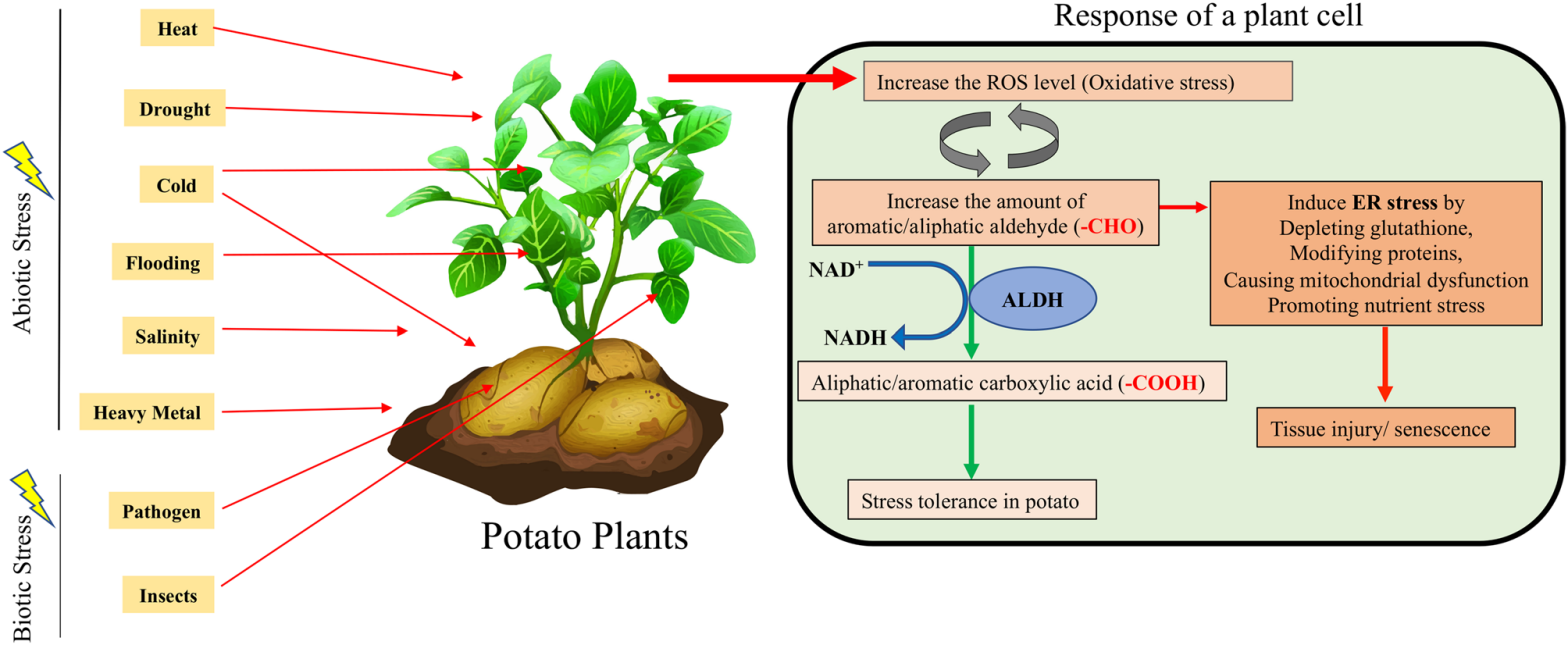
Illustration of the possible role of StALDH in aldehyde detoxification under stress conditions. Different abiotic (heat, cold, salinity, drought, flood, heavy metal) and biotic (pathogen and insects) stresses affect various part of potato plants and induce intercellular reactive oxygen species (ROS) accumulation. ROS in turn forms a vicious cycle by forming reactive aldehydes that could be either detoxified by ALDH or resulted in tissue degeneration. The figure was generated using Adobe Illustrator (https://www.adobe.com/products/illustrator.html).

Cellular functions of a protein are accomplished by 3D folded protein structure and protein–ligand interactions^15^. To gain an insight into its function homology-based modelling of ALDH protein was done previously in rice^10^, maize^15^ and tomato^14^. In the present study, we have analyzed the structure of four abiotic stress-specific proteins for their structural variation mainly in the oligomerization sites and charge distribution in the outer surface. Having identified the putative StALDH proteins along with their transcript profile and subcellular compartments, a cellular model for stress-resistant via aldehyde dehydrogenase has been proposed for potato (Fig. 9). Abiotic and biotic stresses arise a disproportion enhancement of ROS production and induce oxidative stress in general^37^. Oxidative stress, in turn, triggers lipid peroxidation to produces aldehydes as by-products. Thus, ROS induced aldehyde by-products form a vicious circle to further amplify the destructive function of reactive species^38^. Excessive ROS induced aldehydes cause several downstream modifications including depletion of reduced glutathione, protein oxidation/modification, mitochondrial dysfunction, and nutrient stress, which ultimately lead towards endoplasmic reticulum (ER) stress. ER stress promotes the unfolding of proteins, leading to metabolic remodelling, inflammatory responses, cytotoxicity, and even DNA abandonment, hence threatening cellular viability^37^. To counteract the deleterious effects of reactive aldehydes in the cell, StALDH proteins may catalyze the conversion of aldehyde to acid. However, these aldehydes could be reduced by aldose reductase^39^, or neutralized by glutathione conjugation facilitated by glutathione S-transferases^40^.

## Materials and methods

### Identification, characterization, and nomenclature of *ALDH* genes in potato

For the identification of *Solanum tuberosum* ALDH proteins, a BLASTp search was conducted with a stringent E-value cut-off (≤ e − 3) using previously identified ALDH protein sequences of *Arabidopsis*^4^, rice^7^, and tomato^14^ as query sequences; and InterPro ID (IPR015590) search in the Solanaceae Genomics Network database (https://solgenomics.net/). NCBI conserved domain database (https://www.ncbi.nlm.nih.gov/Structure/cdd/wrpsb.cgi) and Pfam server (http://pfam.xfam.org/) were used to confirm the presence of conserved ALDH domain (PF00171) in the resulting protein sequences. The presence of ALDH cysteine active site (PS00070) and glutamic acid active site (PS00687) were confirmed using PROSITE (http://prosite.expasy.org/) and multiple sequence alignment by Clustal Omega (https://www.ebi.ac.uk/Tools/msa/clustalo/). All the confirmed potato ALDH members from *S. tuberosum* were named according to the protocol of the ALDH Gene Nomenclature Committee (AGNC)^11^ and specified as StALDH. According to the AGNC criteria, protein labels (ALDH) were accompanied by a family designation number (1, 2, 3, etc.), a subfamily designation letter (A, B, C, etc.), and a gene description number accordant with chromosomal order. Amino acid sequences greater than 40% identical to the previously identified ALDH sequences were grouped in the same family, and sequences greater than 60% similarity were grouped as protein subfamily. Protein sequences of less than 40% similarity were grouped as novel ALDH protein family. Physical properties of protein such as polypeptide length, pI, and molecular weight were predicted using ExPASy ProtParam (https://web.expasy.org/protparam/) tool. Chromosomal location, gene length, and CDS coordinate (5’ to 3’) were retrieved from the Spud DB database. Subcellular localization of each StALDH proteins was predicted using CELLO v.2.5 (http://cello.life.nctu.edu.tw/)^41^; WoLF PSORT (https://www.genscript.com/wolf-psort.html)^42^, and chloroplast localization was confirmed by ChloroP (http://www.cbs.dtu.dk/services/ChloroP/)^43^.

### Genomic organization and duplication analysis of *StALDH* genes

The genomic position of all the 22 *StALDH* genes was illustrated by CIRCOS software^44^ based on the positional information available in Table S1. For synteny analysis synteny block within the *StALDH* genes were retrieved from the plant genome duplication database (http://chibba.agtec.uga.edu/duplication/index/downloads)^34^. Duplication events were predicted by considering ≥ 80% sequence similarity among the ALDH proteins^40,45^. Tandem duplication events were predicted by finding adjacent homologous *StALDH* genes on the identical chromosome with no more than one gene that separate them^17,34^. Duplicated *StALDH* gene pairs falling in the recognized syntenic blocks were defined as whole-genome duplication or segmental duplication^9,46^. Synonymous rate (d_S_), non-synonymous rate (d_N_), and evolutionary constraint (d_N_/d_S_) were calculated using the PAL2NAL program (http://www.bork.embl.de/pal2nal/)^47^. For the evaluation of duplication time, d_S_ values were calculated as d_S_/(2 × 6.1 × 10^–9^) × 10^–6^ Mya^48^.

### Sequence alignment and phylogenetic analysis of ALDH members

Multiple sequence alignment of 335 ALDH protein sequences (Appendix 3) derived from *Solanum tuberosum* (22 proteins)*, Arabidopsis thaliana* (16 proteins)*, Oryza sativa* (20 proteins)*, Glycine max* (53 proteins)*, Brassica rapa* (23 proteins)*, Vitis vinifera* (23 proteins), *Solanum lycopersicum* (29 proteins), *Zea mays* (23 proteins), *Populus trichocarpa* (27 proteins), *Homo sapiens* (19 sequences), *Chlamydomonas reinhardtii* (9 proteins), *Physcomitrella patens* (21 proteins), *Selaginella moellendorffii* (24 proteins), *Ostreococcus tauri* (6 proteins), and *Mus musculus* (20 proteins) were performed using ClustalW with default parameters^49^. The alignment result was used for the evolutionary genetic analysis and construction of a phylogenetic tree based using the Maximum-likelihood algorithm of MEGA X (https://www.megasoftware.net/) with 1000 bootstrap replicates^50^. Partial deletion with 95% site coverage cut-off and Jones-Taylor-Thornton (JTT) model was taken for the analysis.

### Gene structure analysis, domain assessment, and motif identification

The illustration of *StALDH* gene structures was analyzed using the Gene Structure Display Server 2.0 (GSDS; http://gsds.cbi.pku.edu.cn/)^51^. The presence of conserved ALDH domains in all the 22 potato ALDH proteins was recognized using Pfam (http://www.pfam.xfam.org/). The presence of ALDH active sites was identified using PROSITE (http://prosite.expasy.org/). Afterwards, the domain architecture was drawn manually and combined with the figure of gene structure. Conserved motifs in the putative ALDH protein family were predicted using the Multiple Expectation Maximization for Motif Elicitation (MEME) program (http://meme-suite.org/)^52^ with the default parameters and the maximum number of motifs was set as 10.

### Putative promoter sequence analysis for *cis*-regulatory elements

The 1000 bp of 5’ upstream DNA sequences of all the *StALDH* genes were retrieved from the Spud DB database (http://solanaceae.plantbiology.msu.edu/) and analyzed using the PlantCARE database (http://bioinformatics.psb.ugent.be/webtools/plantcare/html/) for the prediction of putative hormone or stress-responsive *cis*-regulatory elements^53^.

### Gene expression analysis of potato *ALDH*s

The mRNA seq data of the 16 *StALDH* genes (*StALDH2B1*, *StALDH2B3*, *StALDH2B4*, *StALDH2B5*, *StALDH3F1*, and *StALDH6B2* were excluded from the analysis as they had partial domain sequence) in different developmental tissues and responses to abiotic, hormonal, and biotic stress conditions was obtained from Spud DB: Potato Genomics Resource database (http://solanaceae.plantbiology.msu.edu/) using the locus information from *S. tuberosum* Group Phureja DM 1–3 genome. The fragment per kilobase per million reads (FPKM) values were retrieved for thirteen different tissues/developmental stages, including root, tuber, shoot, petiole, leaf, sepal, flower, petal, carpel, stamen, immature fruits, mature fruit and inside of fruit (Messocarp & endocarp). Different abiotic stresses including 150 mM NaCl (Salinity), 260 µM mannitol (Dehydration), incubation at 35^0^C (Heat); and hormonal treatment of 10 µM BAP (6-benzyl amino purine), 10 µM IAA (indole-3-acetic acid), 50 µM ABA (abscisic acid), and 50 µM GA3 (gibberellic acid) were given at the whole plant for 24 h, and the fold change in expression (FPKM value under stress condition / FPKM value at control condition) was calculated as compared with respective control/untreated samples. Biotic stress treatments included BABA (β-aminobutyric acid), BTH (benzothiadiazole), and pathogen challenge were imposed on the leaf for 72 h. The samples were pooled at 24, 48, and 72 h; and the fold change in expression was calculated as compared with the respective untreated 24, 48, and 72 h pooled samples. The expression data was used to create the heat map by hierarchical clustering with the Manhattan correlation coefficient distance measurement method using MeV 4.9 software (http://mev.tm4.org)^54^.

### Plant materials and stress treatment

The expression profiles of *StALDH* genes were evaluated in one of the cultivated potato variety of Bangladesh, Diamant (BARI Alu-7). Seeds were purchased from the Bangladesh agricultural development corporation (BADC), Srimangol, Bangladesh. All the experimental research on plants were conducted according to the proper guidelines and legislation of national and international regulations. Potato seedlings were grown in a culture room (16 h light/8 h dark and 24–26 °C temperature) at the Plant Genetic Engineering Laboratory, Department of Genetic Engineering and Biotechnology, Shahjalal University of Science and Technology University, Sylhet-3114, Bangladesh according to Qin et al.^55^. Seedlings (15 days old) were subjected to 150 mM NaCl for salinity or 260 mM mannitol for drought or kept at 37 ± 1 °C for heat treatment. Samples of each treatment with triplicates were harvested after 24 h and stored at − 80 °C after frozen in liquid nitrogen until RNA isolation.

### RNA isolation, cDNA synthesis and qRT-PCR

Total RNA was extracted from the stored samples using TRIzol reagent (Invitrogen, USA) following the manufacturer’s instructions. GoScript Reverse Transcription System (Promega, USA) was used for cDNA synthesis following RNase-free DNase I (Invitrogen) treatment to the isolated RNA. Primer-BLAST program (http://www.ncbi.nlm.nih.gov/tools/primer-blast/) was used to design the gene-specific primers for the selected *StALDH* genes and *StActin* (Accession number: X55749) gene was used as a housekeeping control^55^. All these primers were synthesized from Macrogen (http://dna.macrogen.com/eng/) and listed in Table S6. SYBR Green PCR Master Mix (Thermo Fisher Scientific, USA) and Applied Biosystems StepOne Real-Time PCR System were used to perform real-time PCR assay with a thermal cycling of 94 °C for 10 min, 45 cycles of 94 °C for 15 s, 60 °C for 30 s and 72 °C for 45 s. The relative quantification for the fold change in expression of each gene was calculated based on the 2^(- Delta Delta CT) method^56^ as described previously.

### Structural features analysis and homology modelling of StALDH proteins

The secondary structure of potato ALDH proteins was predicted using the SOPMA (Self-Optimized Prediction Method with Alignment; https://npsa-prabi.ibcp.fr/cgi-bin/npsa_automat.pl?page=/NPSA/npsa_sopma.html) tool^57^. N-glycosylation sites of StALDHs were recognized using NetNGlyc 1.0 server (http://www.cbs.dtu.dk/services/NetNGlyc/)^58^. Four abiotic stress-responsive proteins StALDH3H1, StALDH10A1, StALDH11A1, and StALDH12A1 were selected for the homology modelling using suitable homologous templates from the PDB database (http://ncbi.nlm.nih.gov/). ALDH protein models were built by the top PDB closed template via the target-template input using SWISS-MODEL of the ExPASy web server (https://swissmodel.expasy.org/). Discovery Studio Visualizer 2016 software (https://discover.3ds.com/discovery-studio-visualizer-download)^59^ was used to visualize the predicted structures and verified with MolProbity Ramachandran analysis using PSVS (https://montelionelab.chem.rpi.edu/PSVS/).

## Conclusion

In conclusion, we have identified a total of 22 putative ALDH members, that were grouped into ten families. Detailed investigation of these genes was carried out regarding their classification, genomic organization, sub-cellular localization, structure, evolution, promotor analysis, and protein modelling. Moreover, analyses of their expression profiles at various potato tissues and under biotic and abiotic stress treatments widen our understanding of this multidimensional protein. Collectively, this study led to the functional characterization of potato *ALDH* genes. Unlike other previous reports, the current study covered a wider perspective of the detoxification process of reactive aldehydes that will pave way for many more future studies for a better understanding of stress alleviation pathways in plants.

## Supplementary Information

Supplementary Information.

## Data Availability

The authors declare that all the data and plant materials will be available without restrictions.
