# Supplementary material for: Genome-wide identification, evolution, and transcript profiling of Aldehyde dehydrogenase superfamily in potato during development stages and stress conditions
Source: Sci Rep. 2021 Sep 14;11:18284. doi: 10.1038/s41598-021-97691-9 (PMC8440639; doi:10.1038/s41598-021-97691-9)
Supplement: Supplementary file 1 — Supplementary Information. [file 41598_2021_97691_MOESM1_ESM.pdf]

Genome-wide identification, evolution, and transcript profiling of Aldehyde dehydrogenase superfamily in potato during development stages and stress conditions

Short title: Identification and transcript profiling of StALDH superfamily

Md. Sifatul Islam[1], Md. Soyib Hasan[1], Md. Nazmul Hasan[2], Shamsul H. Prodhan[2], Tahmina Islam[3], Ajit Ghosh[1,*]

1Department of Biochemistry and Molecular Biology, Shahjalal University of Science and Technology, Sylhet-3114, Bangladesh;
2Department of Genetic Engineering and Biotechnology, Shahjalal University of Science and Technology, Sylhet-3114, Bangladesh;
3Department of Botany, University of Dhaka, Dhaka-3114, Bangladesh.

*To whom correspondence should be addressed:
Ajit Ghosh Ph.D.,
E-mail: ajitghoshbd@gmail.com, aghosh-bmb@sust.edu

Supplementary Table S1. Detailed information of the identified StALDH members including their subfamilies, size variation, structural arrangements, and subcellular localization

Family	Gene Name	*Solanum tuberosum* group Phureja DM1-3 - PGSC loci	Chr	CDS coordinate (5' to 3')	Strand	Length		Conserved domain/sites			MW (kDa)	pI	Localization
						Gene (nt)	Protein (aa)	PF00171 start-stop	PS00687 yes/no	PS00070 yes/no			
Family 2	StALDH2B1	PGSC0003DMG402000528	2	46920438-46926955	+	6518	182	98-158	No	No	20.43	7.63	Cp[1,2]
	StALDH2B2	PGSC0003DMG400024490	3	54102305-54107157	+	4853	535	63-525	Yes	Yes	58.33	8.02	Mt[1,2], Cp[2,3]
	StALDH2B3	PGSC0003DMG400002213	4	31398921-31400925	+	2005	136	56-134	No	No	15.57	9.85	Nu[1], Cp[2]
	StALDH2B4	PGSC0003DMG402004125	5	7774270-7779490	+	5221	240	128-230	No	No	26.54	5.70	Cy[1], Cp[2]
	StALDH2B5	PGSC0003DMG400017005	5	8018695-8022801	-	4107	120	24-110	No	No	13.71	10.0	Mt[1], Nu[2]
	StALDH2B6	PGSC0003DMG401004125	5	7767911-7772402	+	4492	529	57-519	Yes	Yes	57.92	8.04	Mt[1,2]
	StALDH2B7	PGSC0003DMG400014496	8	43082891-43091958	+	9068	594	122-584	Yes	Yes	65.60	9.25	Mt[1], Cp[2,3]
	StALDH2C1	PGSC0003DMG400000321	12	4625886-4633484	+	7599	500	28-490	Yes	Yes	54.68	5.10	Cy[1,2]
Family 3	StALDH3F1	PGSC0003DMG400019991	1	49970671-49977513	-	6843	285	1-240	Yes	Yes	31.89	8.93	Pm[1], Cy[2]
	StALDH3F2	PGSC0003DMG400003619	2	39881712-39886402	+	4691	489	7-437	Yes	Yes	54.39	6.30	Pm[1], Cy[1,2]
	StALDH3H1	PGSC0003DMG400017568	6	43110843-43117994	-	7152	474	1-432	Yes	Yes	51.98	8.70	Cy[1,2]
Family 5	StALDH5F1	PGSC0003DMG400017144	9	57005229-57007827	-	2599	513	48-507	Yes	Yes	54.46	6.92	Cp[1], Mt[2]
Family 6	StALDH6B1	PGSC0003DMG402010673	1	82766951-82772932	-	5982	537	51-515	No	Yes	57.77	6.53	Mt[1,2], Cp[1,2]
	StALDH6B2	PGSC0003DMG401010673	1	82773700-82781913	-	8214	88	1-44	No	Yes	9.35	9.45	Mt[1], Cp[2]
Family 7	StALDH7A1	PGSC0003DMG400022885	3	61505990-61512715	-	6726	509	30-492	Yes	Yes	54.64	5.64	Cy[1,2]
Family 10	StALDH10A1	PGSC0003DMG400024582	3	53757940-53766241	-	8302	505	19-486	Yes	Yes	55.90	5.27	Cy[1], Po[2]
	StALDH10A2	PGSC0003DMG400033028	6	52130166-52135413	-	5248	504	19-486	Yes	Yes	54.91	6.14	Cy[1], Mt[2]
Family 11	StALDH11A1	PGSC0003DMG400011132	7	583375-589095	+	5721	496	24-484	Yes	Yes	53.13	6.76	Cy[1,2]
Family 12	StALDH12A1	PGSC0003DMG400033072	6	51835999-51843555	+	7557	554	60-514	Yes	Yes	61.45	6.79	Mt[1,2], Cp[2,3]
Family 18	StALDH18A1	PGSC0003DMG402026767	6	15776837-15786259	+	9423	717	281-561	No	Yes	77.47	5.64	Cy1, ER[2]
	StALDH18A2	PGSC0003DMG401026767	6	15776950-15784535	+	7586	632	281-565	No	Yes	68.44	5.76	Cy1, ER[2]
Family 22	StALDH22A1	PGSC0003DMG400004698	12	60024228-60031694	-	7467	593	52-523	Yes	Yes	65.59	7.86	Pm[1], Ec[2]

Abbreviations: Chr, chromosome number; MW, Molecular Weight; pI, Isoelectric point; nt, nucleotide; aa, amino acid; kDa, kilodalton; Cp, Chloroplast; Cy, Cytosol; Ec, Extracellular; ER, Endoplasmic reticulum; Mt, Mitochondria; Nu, Nucleus; Pm, Plasma-membrane; Po, Peroxisome.

Sub-cellular localization was prediction by

1. CELLO v.2.5 (http://cello.life.nctu.edu.tw/)
2. WoLF PSORT (https://www.genscript.com/wolf-psort.html)
3. ChloroP (http://www.cbs.dtu.dk/services/ChloroP/)

Supplementary Table S2. Gene duplication analysis of potato *ALDH* genes

**No.**	**Locus 1**	**Locus 2**	**Identity (%)**	**d_N**	**d_S**	**d_N/d_S**	**Duplication time (Mya)**	**Duplication type**
1	StALDH2B5	StALDH2B4	85	0.2688	0.4812	0.5585	16.0	Tandem
2	StALDH2B5	StALDH2B6	89	0.2824	0.3247	0.8697	10.8	Tandem
3	StALDH2B6	StALDH2B2	80	0.1337	1.8532	0.0721	61.7	WGD/Segmental
4	StALDH2B6	StALDH2B7	82	0.1169	0.7766	0.1505	25.8	WGD/Segmental
5	StALDH10A1	StALDH10A2	82	0.1061	0.5656	0.1876	18.8	WGD/Segmental
6	StALDH18A1	StALDH18A2	99	0.0098	0.0120	0.8184	0.4	Tandem

Supplementary Table S3. Detailed information of putative conserved motifs in the StALDH proteins

No.	Motif	Width	Site	E-value
1	PTILSBVKDDMLIAKEEIFGPVLPILKFKTVEEVIERANSTKYGLAAGVF	50	15	1.2e-246
2	PFGGYKMSGFGREKGEYGLDNYLQVKAVVTPLKN	34	13	4.5e-149
3	LKPVTLELGGKSPFIVFEDAD	21	14	2.4e-100
4	AVELALFAJFYNQGQCCCAGSRLYVHESIYDEF	33	11	1.2e-095
5	HVYTLREPLGVVGVITPWNFPLLMFLWK	28	14	3.5e-103
6	GZFVDSASGKTFPTJBPRTGEVIAHIPEGDAEDVNRAVSAARKA	44	10	3.8e-107
7	ALYVAELLREAGLPPGVLNIVTGFGPTAGAALCSHPDVDKJAFTGSTETG	50	8	3.1e-104
8	SKBIDTANTLTRALRVGTVWINCFDTFDA	29	8	1.7e-074
9	PALAAGNTIVLKPSEQAPLS	20	15	1.6e-058
10	PKMSAYERSKILLKJADLIEEKNDEIATLETWDTG	35	10	3.4e-043

Supplementary Table S4. Prediction of secondary structure of the identified StALDH proteins

No	Protein	Alpha helix (Hh)	Extended Strand (Ee)	Beta Turn (Tt)	Random Coil (Cc)
1	StALDH2B2	43.18%	16.64%	7.85%	31.78%
2	StALDH2B6	44.23%	16.45%	7.75%	31.57%
3	StALDH2B7	40.40%	16.84%	7.74%	35.02%
4	StALDH2C1	40.20%	17.80%	8.60%	33.40%
5	StALDH3F2	35.79%	18.60%	9.82%	35.79%
6	StALDH3H1	43.04%	17.51%	7.38%	32.07%
7	StALDH5F1	46.00%	15.98%	7.60%	30.41%
8	StALDH6B1	36.50%	19.37%	8.38%	35.75%
9	StALDH7A1	42.44%	17.29%	4.52%	35.76%
10	StALDH10A1	43.76%	15.84%	6.93%	33.47%
11	StALDH10A2	41.67%	17.06%	7.34%	33.93%
12	StALDH11A1	41.53%	18.35%	6.85%	33.27%
13	StALDH12A1	38.45%	13.72%	4.15%	43.68%
14	StALDH18A1	49.93%	17.43%	5.86%	26.78%
15	StALDH18A2	52.06%	15.66%	6.33%	25.95%
16	StALDH22A1	44.01%	13.49%	5.56%	36.93%

Supplementary Table S5. Prediction of N-glycosylation sites of all the identified StALDH proteins

No.	Name	No of site	Position	Region	Score
1	StALDH2B2	2	53	NHTK	0.6519
			455	NNTR	0.5466
2	StALDH2B6	0	--	--	--
3	StALDH2B7	2	13	NKTH	0.7808
			48	NWSV	0.6192
4	StALDH2C1	1	420	NCTN	0.6468
5	StALDH3F2	0	--	--	--
6	StALDH3H1	0	--	--	--
7	StALDH5F1	0	--	--	--
8	StALDH6B1	2	125	NVTT	0.7639
			165	NVSN	0.5713
9	StALDH7A1	2	27	NGTW	0.5869
			136	NGSV	0.6988
10	StALDH10A1	2	416	NDTK	0.5182
			449	NCSQ	0.5372
11	StALDH10A2	2	416	NDTK	0.5221
			449	NCSQ	0.5451
12	StALDH11A1	1	37	NPTT	0.5382
13	StALDH12A1	3	26	NSTR	0.5224
			31	NHTL	0.6194
			346	NWSK	0.5358
14	StALDH18A1	3	77	NSSF	0.5776
			138	NETV	0.7144
			176	NDSL	0.6357
15	StALDH18A2	3	77	NSSF	0.5768
			138	NETV	0.7123
			176	NDSL	0.6319
16	StALDH22A1	1	126	NISS	0.7024

Table S6. List of primers *StALDH* gene-specific and housekeeping primers used for the qRT-PCR analysis.

No	Primer Name	Forward Primer (5′→3′)	Reverse Primer (5′→3′)	Product size
1	StALDH2B6	ACAGAGCAGGGTCCTCAGAT	TACCAAATTGCTCACCGCCA	112
2	StALDH2C1	CATGGGTGGTTGGTGATCCT	GCAATTTTGCACCCTCCCTC	120
3	StALDH3H1	GCCAGAGAAAAGCCACTTGC	ATGGAAGAGTGGAAAGCGCA	136
4	StALDH5F1	GCTTGGCGTGTTACTGAAGC	TGATTGTTTCACGCCTCCGA	99
5	StALDH6B1	GCAAGCAGGCAAAAGAACGA	CCAGACAGGATTGTGGGACC	139
6	StALDH7A1	TGGTCAGCGCTGTACAACAT	TGCAATGGCCCAAGTAAGGT	141
7	StALDH10A1	AGACTGCAAGCTTGGTCCTG	TTAAGTGCTCAGGACGGTCG	122
8	StALDH11A1	GGTCATGGACGCAAAGGAGA	GCGATTCTCATGTCTGGCCT	111
9	StALDH12A1	CAACAGGGGCTCCACAGAAT	CATCCTAGTGGCATGGGACC	142
10	StALDH18A2	GCTTGCACCGTGGAAATTGT	TGAGAAATGTAACTTGCCTTCCA	82
11	StALDH22A1	ACCGGTGGTGGCCATTTATT	TCACCAATGCCCGTAAACGA	141
12	StActin	GCTTCCCGATGGTCAAGTCA	GGATTCCAGCTGCTTCCATTC	111

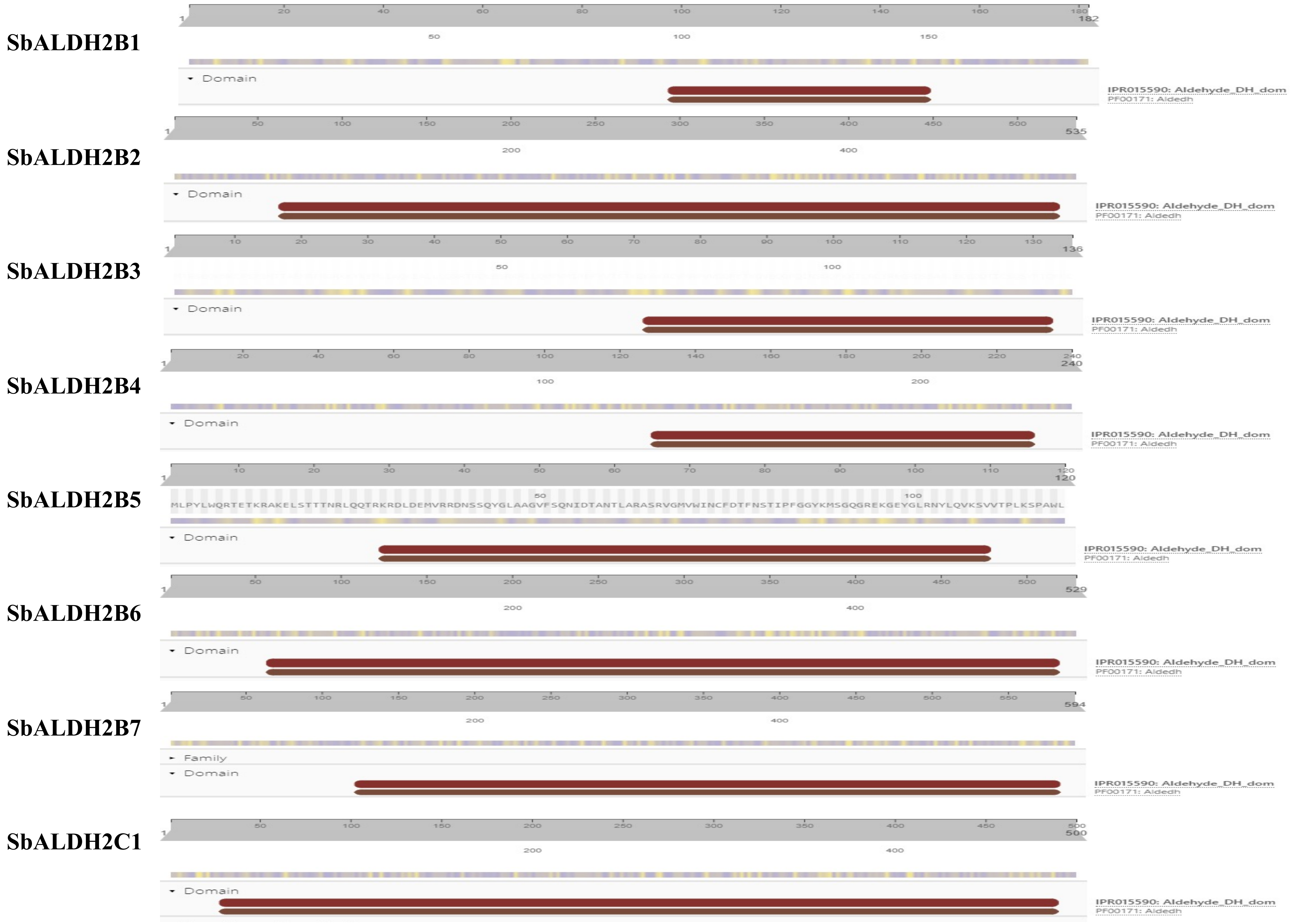

Fig. S1 (A). Distribution of the conserved aldehyde dehydrogenase domain (IPR015590/PF00171) in the identified StALDH proteins.

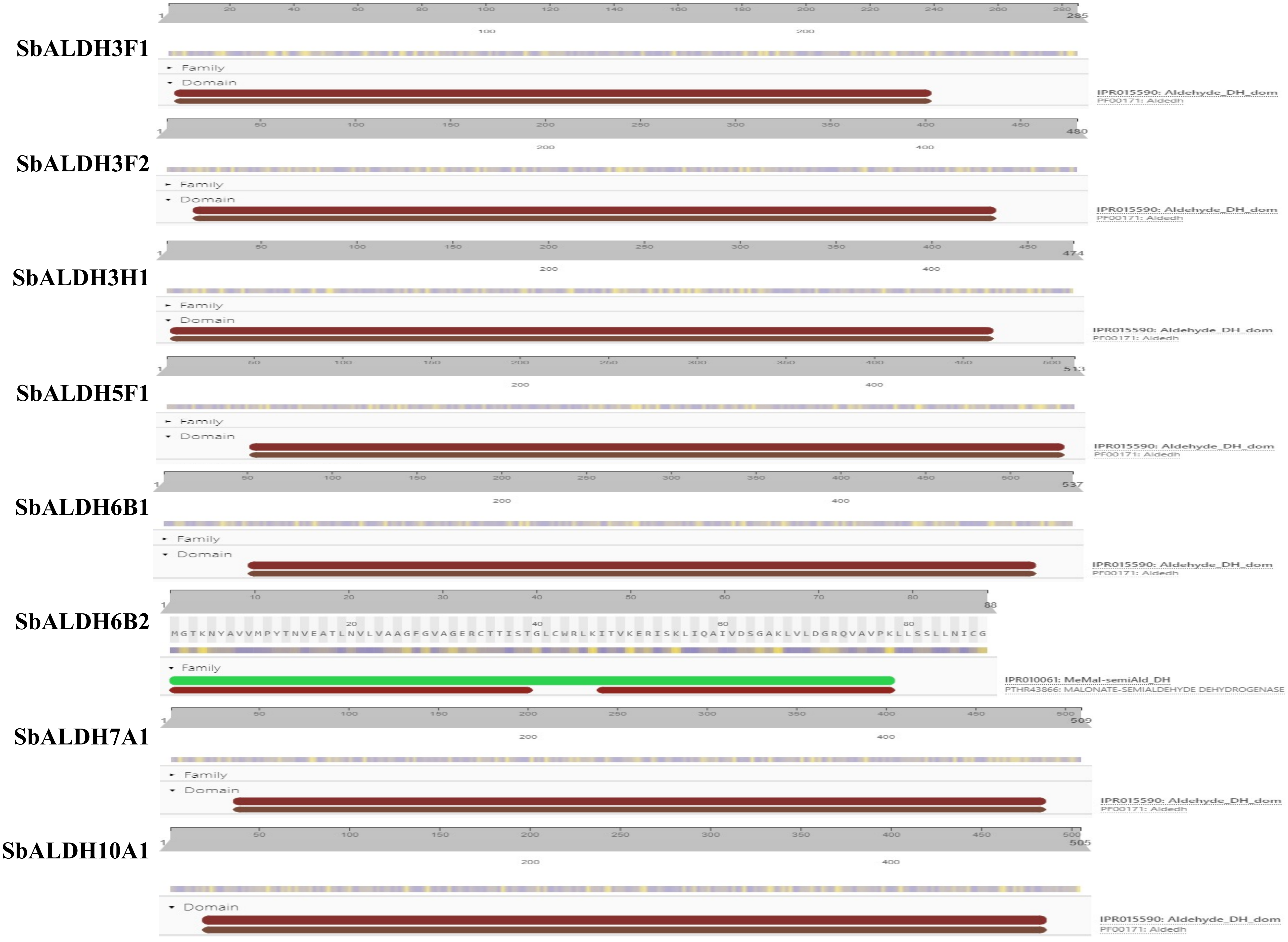

Fig. S1 (B). Distribution of the conserved aldehyde dehydrogenase domain (IPR015590/PF00171) in the identified StALDH proteins.

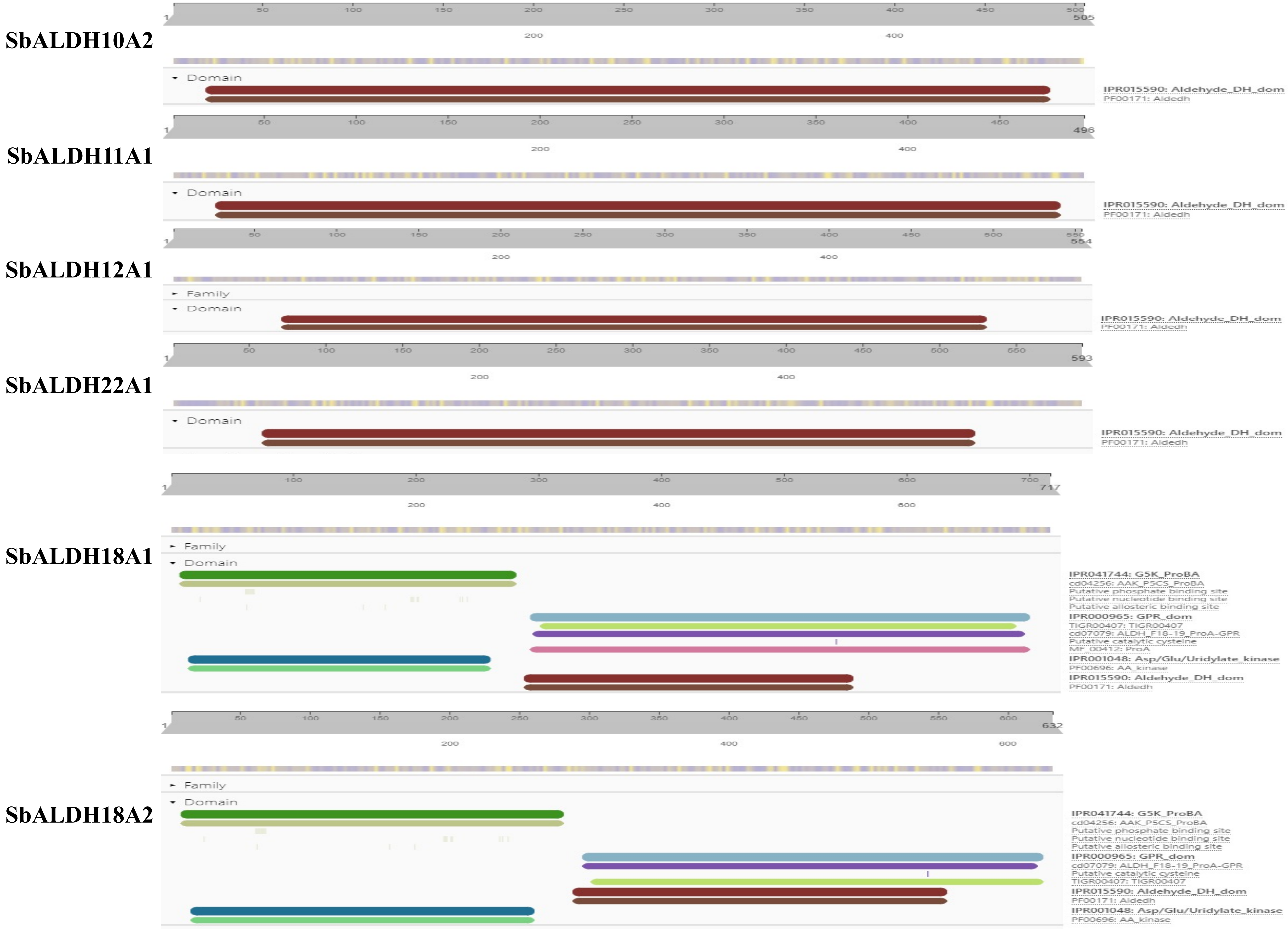

Fig. S1 (C). Distribution of the conserved aldehyde dehydrogenase domain (IPR015590/PF00171) in the identified StALDH proteins.

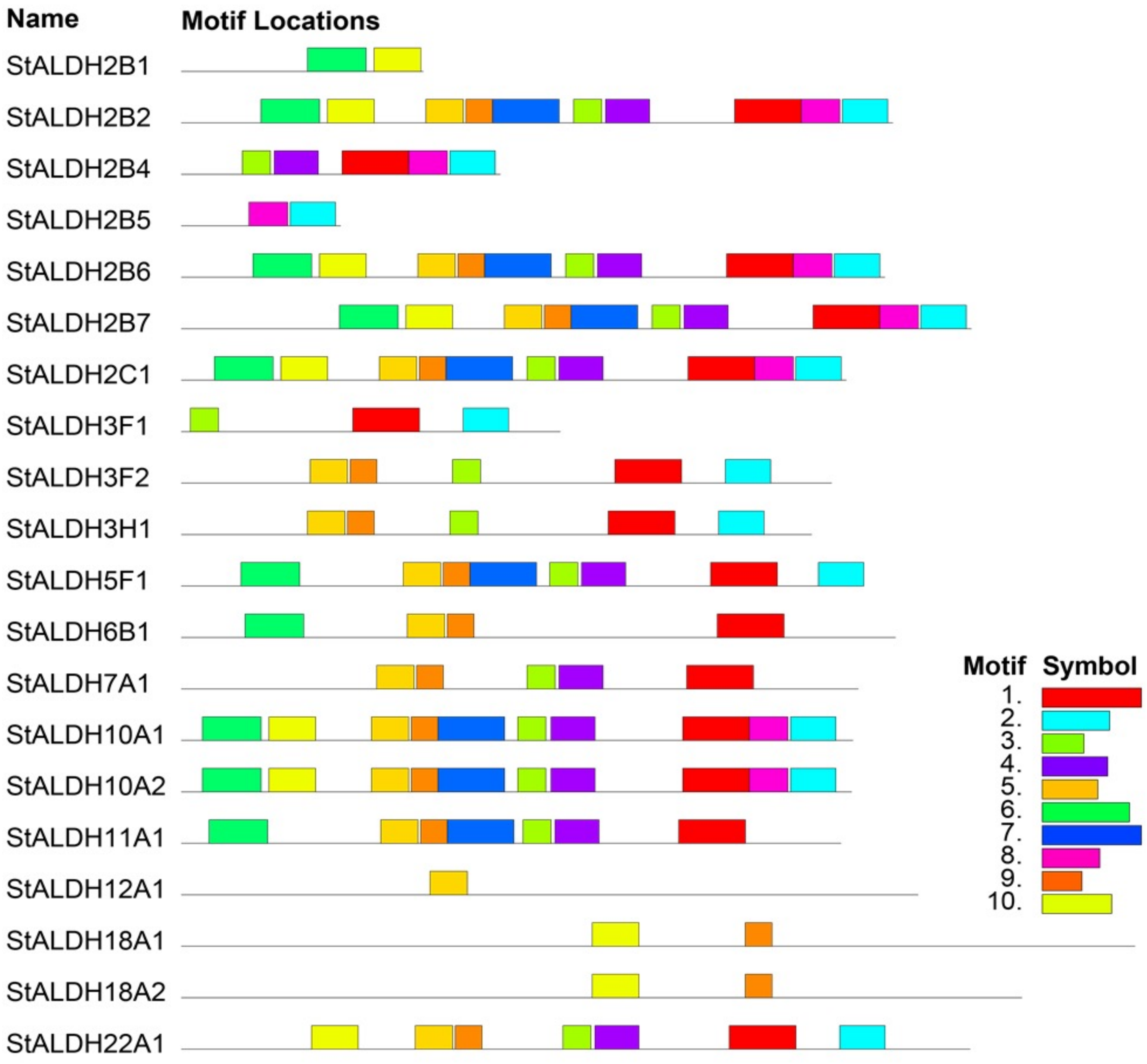

Fig. S2. The conserved motif in potato ALDH superfamily. The different-colored boxes represent different motifs and their position in each ALDH protein sequence. Figures were generated using the Multiple Expectation Maximization for Motif Elicitation (MEME) program (http://meme-suite.org/) with the default parameters and the maximum number of motifs was set as 10.

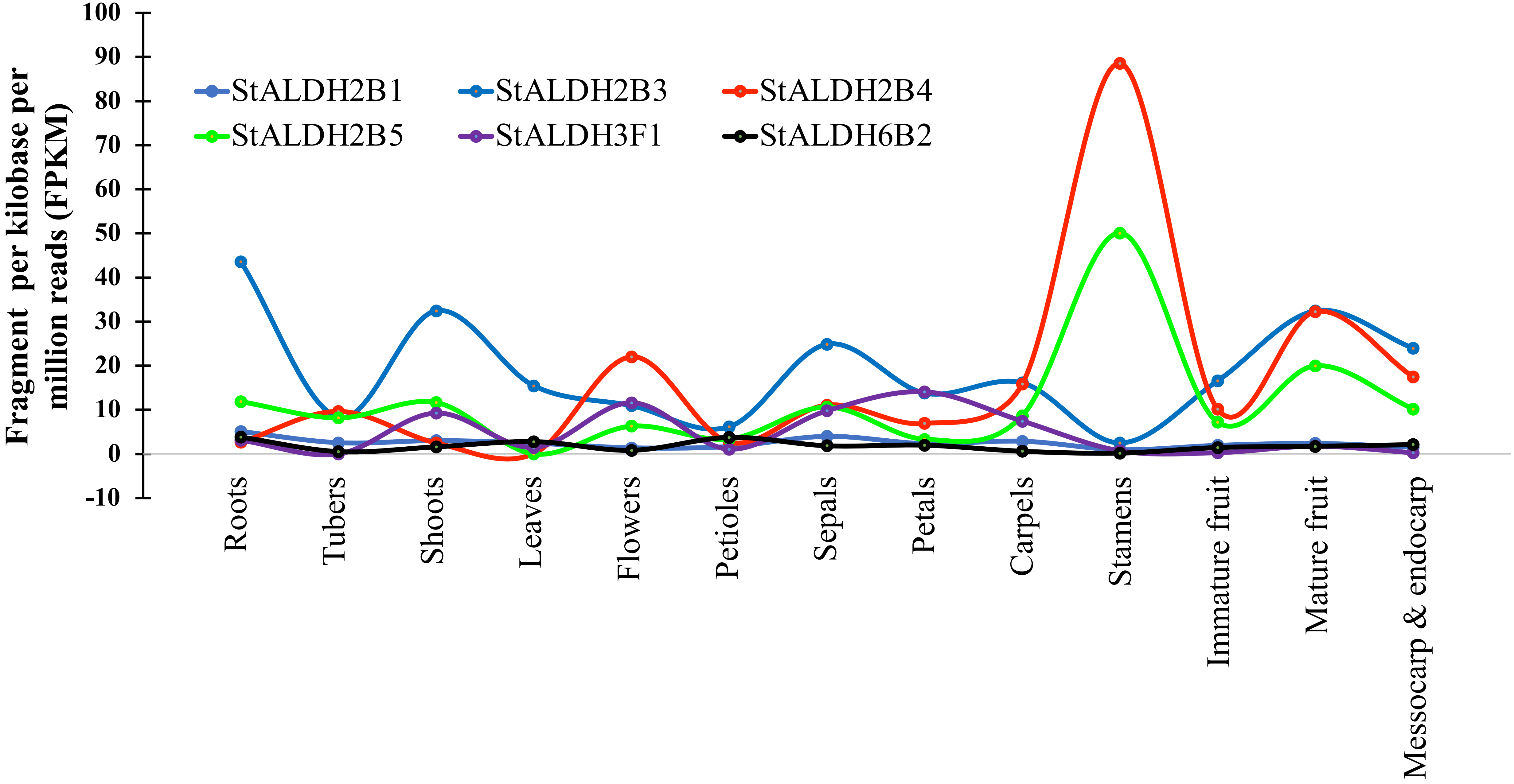

Fig. S3. Expression profiles of six putative psedo-*StALDH* genes at different tissues. Transcript abundance of 6 *StALDH* genes in thirteen different tissues (roots, tubers, shoots, leaves, flowers, petioles, sepals, petals, carpels, stamens, immature fruits, mature fruit and inside of fruit) were analyzed. The Fragments Per Kilobase of transcript per Million mapped reads (FPKM) values were normalized and used for the generation of bar diagram. The color line represents one individual gene.

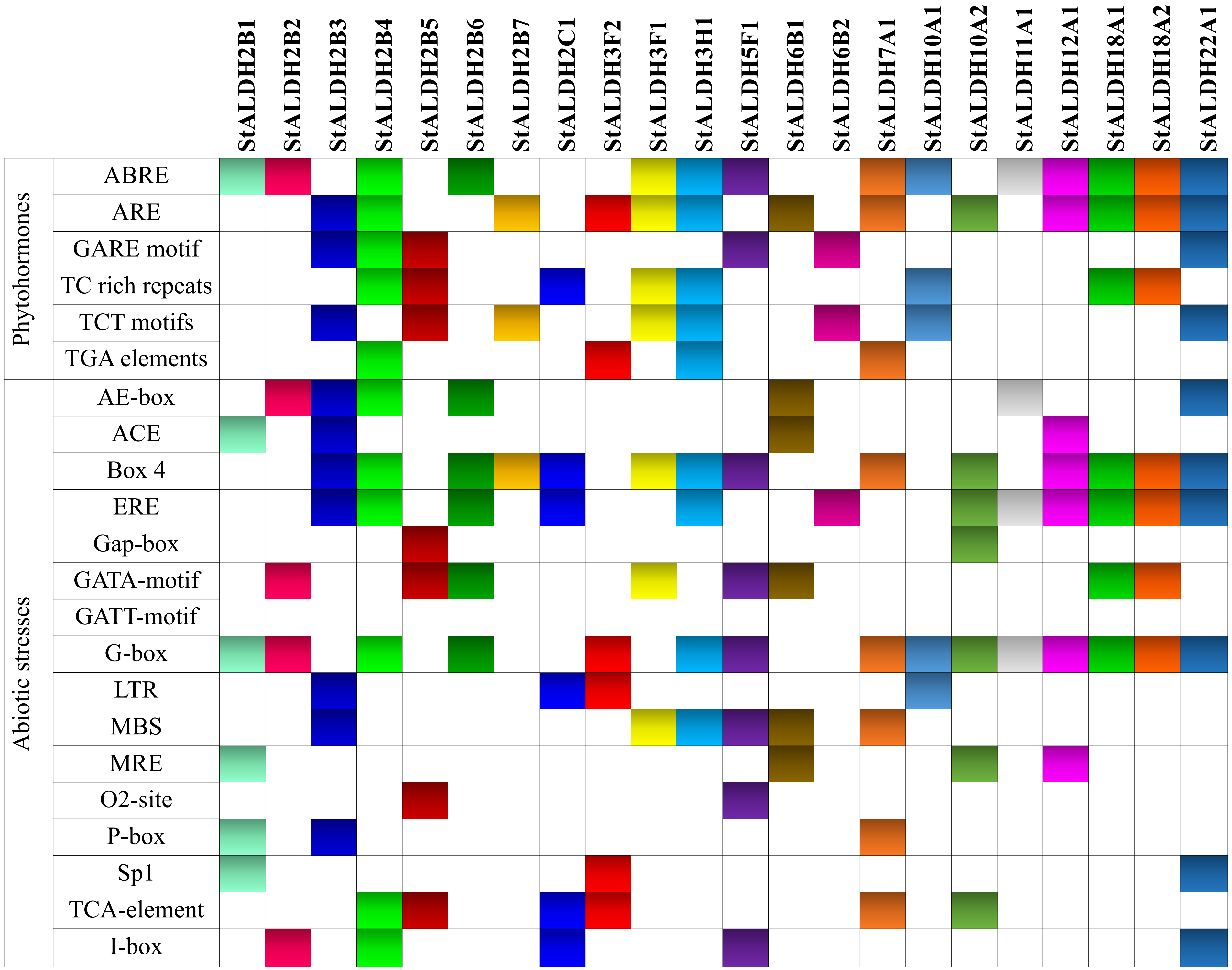

Fig. S4. *Cis*-regulatory elements identified in potato ALDH promoters. The 1kb sequence from the translation start codon of the genes were used for analyzing the presence of cis-regulatory elements. The graph was plotted based on the presence of cis-regulatory elements that was categorized into two segment: phytohormones and abiotic stresses. Note: Figure was generated by Microsoft Excel 2010 (https://www.microsoft.com/en-us/download/office.aspx).

StALDH3H1

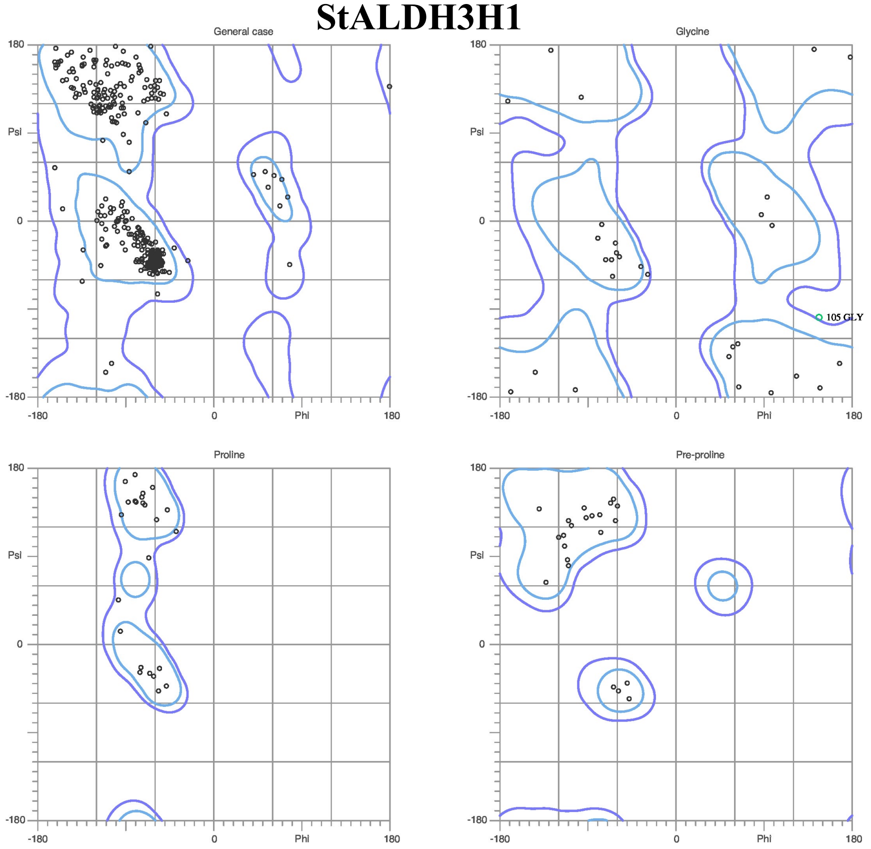

95.1% (425/447) of all residues were in favored (98%) regions.
99.8% (446/447) of all residues were in allowed (>99.8%) regions.

There were 1 outliers (phi, psi):
105 GLY (146.2, -98.1)

StALDH10A1

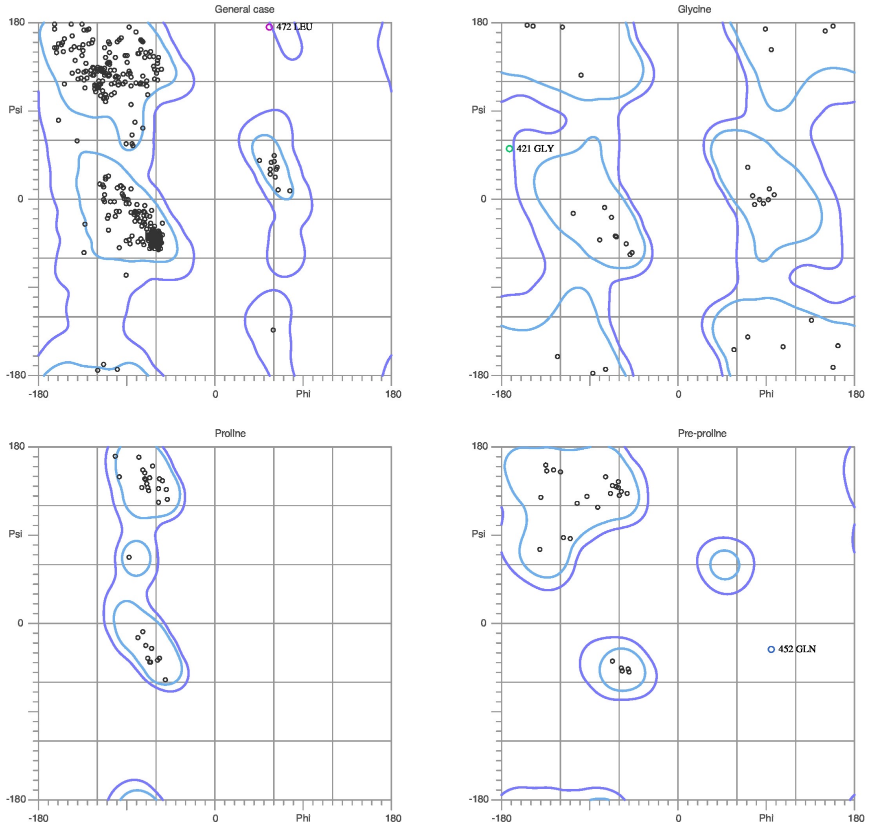

97.8% (484/495) of all residues were in favored (98%) regions.
99.4% (492/495) of all residues were in allowed (>99.8%) regions.

There were 3 outliers (phi, psi):
421 GLY (-172.3, 52.6)
452 GLN (95.6, -26.2)
472 LEU (55.2, 176.3)

StALDH11A1

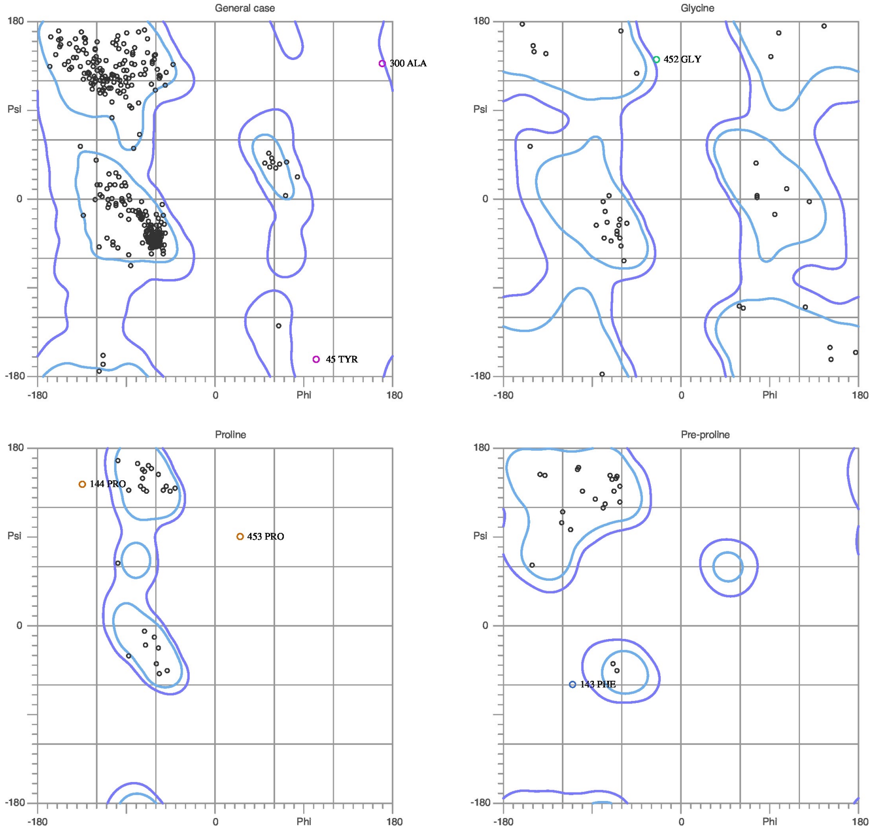

96.0% (456/475) of all residues were in favored (98%) regions.
98.7% (469/475) of all residues were in allowed (>99.8%) regions.

There were 6 outliers (phi, psi):
45 TYR (102.8, -162.2)
143 PHE (-110.5, -59.0)
144 PRO (-135.3, 144.8)
300 ALA (169.3, 138.4)
452 GLY (-25.1, 142.7)
453 PRO (26.0, 91.5)

StALDH12A1

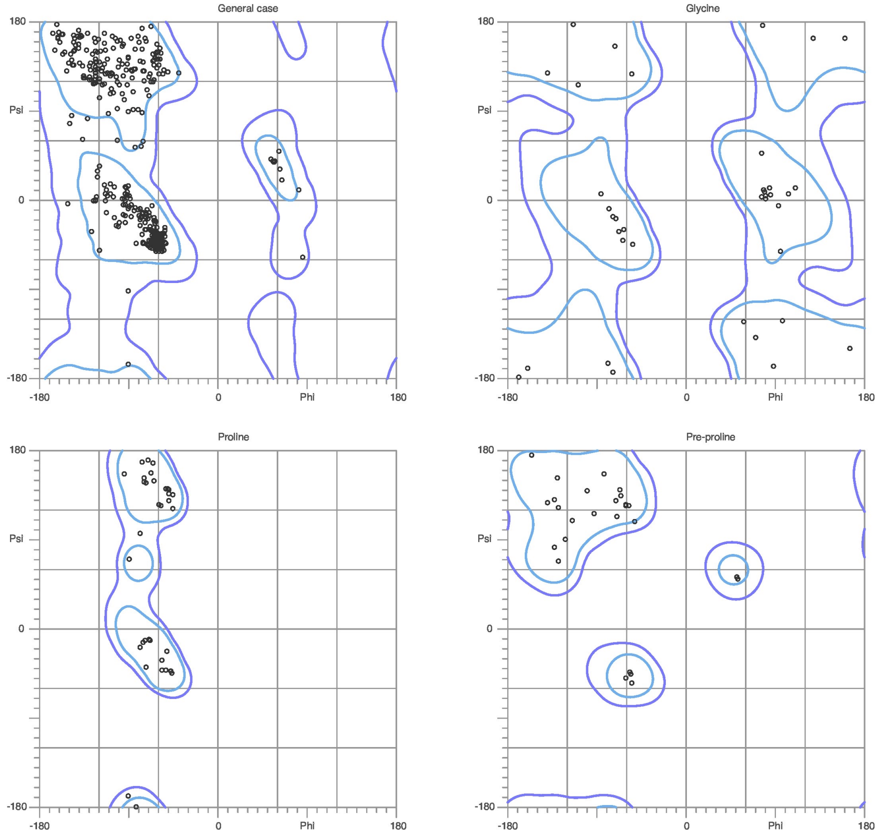

97.7% (508/520) of all residues were in favored (98%) regions.
100.0% (520/520) of all residues were in allowed (>99.8%) regions.

There were no outliers.

Fig. S5. MolProbity Ramachandran analysis. Ramachandran plot analysis for the validation of 3D homology model of four selected StALDH proteins. Favorable and allowed regions of different cases were shown in the figure. Discovery Studio Visualizer 2016 software was used to visualize the predicted structures and verified with MolProbity Ramachandran analysis using PSVS (http://psvs-1_5-dev.nesg.org/).

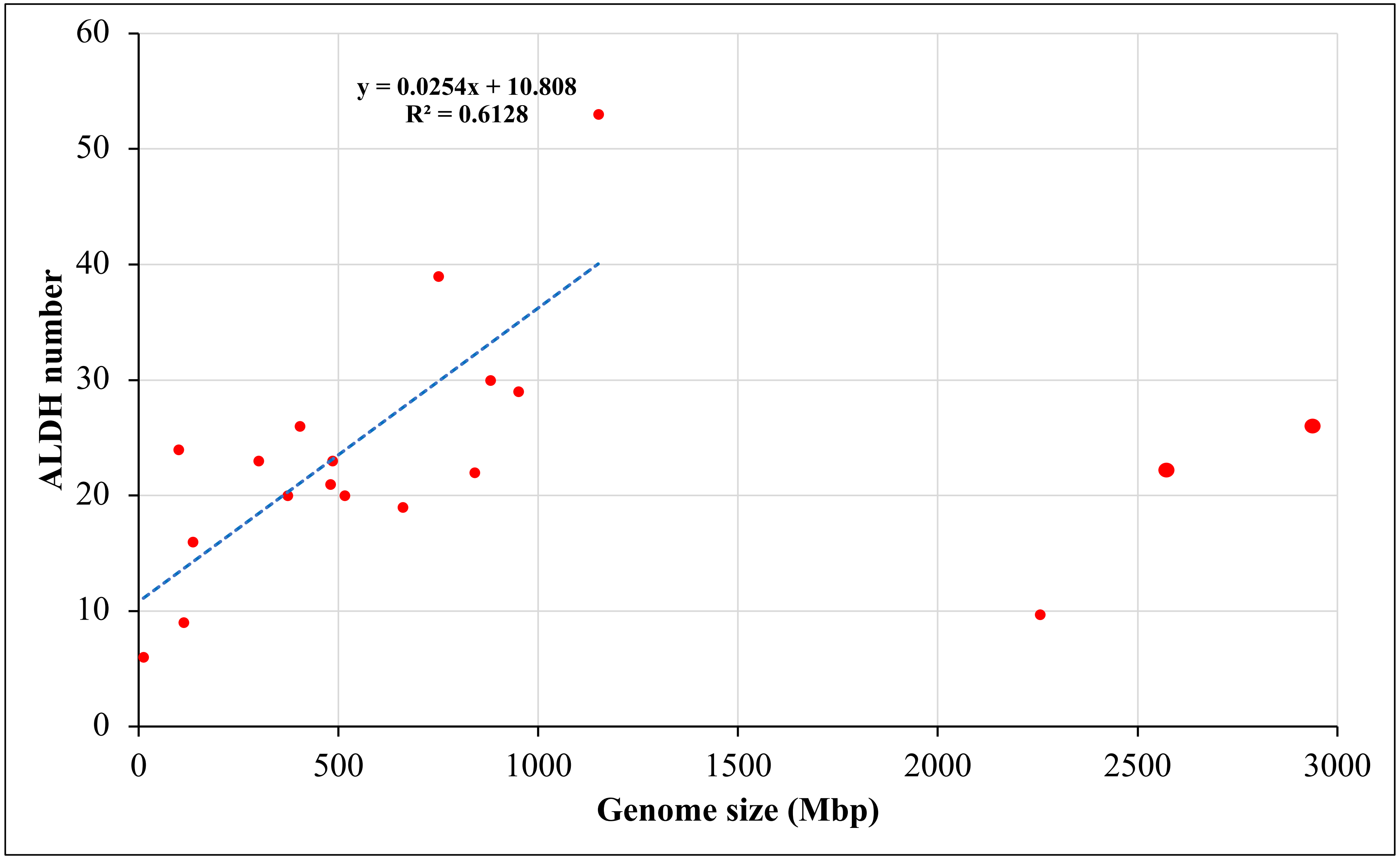

Fig. S6. Scatter plot to corelate the ALDH gene numbers with the respective genome size of different species. Note: Figure was generated by Microsoft Excel 2010 (https://www.microsoft.com/en-us/download/office.aspx).

Appendix 1. Unique identification ID of the StALDH members

SL no	Gene Name	Genome ID	Protein ID	Transcript ID	CDS ID
1	StALDH2B1	PGSC0003DMG402000528	PGSC0003DMP400001061	PGSC0003DMT400001429	PGSC0003DMC400001061
2	StALDH2B2	PGSC0003DMG400024490	PGSC0003DMP400042353	PGSC0003DMT400062930	PGSC0003DMC400042353
3	StALDH2B3	PGSC0003DMG400002213	PGSC0003DMP400003944	PGSC0003DMT400005645	PGSC0003DMC400003944
4	StALDH2B4	PGSC0003DMG402004125	PGSC0003DMP400007373	PGSC0003DMT400010556	PGSC0003DMC400007373
5	StALDH2B5	PGSC0003DMG400017005	PGSC0003DMP400029726	PGSC0003DMT400043817	PGSC0003DMC400029726
6	StALDH2B6	PGSC0003DMG401004125	PGSC0003DMP400007372	PGSC0003DMT400010555	PGSC0003DMC400007372
7	StALDH2B7	PGSC0003DMG400014496	PGSC0003DMP400025542	PGSC0003DMT400037582	PGSC0003DMC400025542
8	StALDH2C1	PGSC0003DMG400000321	PGSC0003DMP400000631	PGSC0003DMT400000852	PGSC0003DMC400000631
9	StALDH3F1	PGSC0003DMG400019991	PGSC0003DMP400034695	PGSC0003DMT400051470	PGSC0003DMC400034695
10	StALDH3F2	PGSC0003DMG400003619	PGSC0003DMP400006467	PGSC0003DMT400009314	PGSC0003DMC400006467
11	StALDH3H1	PGSC0003DMG400017568	PGSC0003DMP400030691	PGSC0003DMT400045297	PGSC0003DMC400030691
12	StALDH5F1	PGSC0003DMG400017144	PGSC0003DMP400029942	PGSC0003DMT400044163	PGSC0003DMC400029942
13	StALDH6B1	PGSC0003DMG402010673	PGSC0003DMP400018869	PGSC0003DMT400027691	PGSC0003DMC400018869
14	StALDH6B2	PGSC0003DMG401010673	PGSC0003DMP400018867	PGSC0003DMT400027687	PGSC0003DMC400018867
15	StALDH7A1	PGSC0003DMG400022885	PGSC0003DMP400039674	PGSC0003DMT400058896	PGSC0003DMC400039674
16	StALDH10A1	PGSC0003DMG400024582	PGSC0003DMP400042549	PGSC0003DMT400063205	PGSC0003DMC400042549
17	StALDH10A2	PGSC0003DMG400033028	PGSC0003DMP400055759	PGSC0003DMT400083025	PGSC0003DMC400055759
18	StALDH11A1	PGSC0003DMG400011132	PGSC0003DMP400019654	PGSC0003DMT400028915	PGSC0003DMC400019654
19	StALDH12A1	PGSC0003DMG400033072	PGSC0003DMP400055842	PGSC0003DMT400083137	PGSC0003DMC400055842
20	StALDH18A1	PGSC0003DMG402026767	PGSC0003DMP400046503	PGSC0003DMT400068829	PGSC0003DMC400046503
21	StALDH18A2	PGSC0003DMG401026767	PGSC0003DMP400046501	PGSC0003DMT400068827	PGSC0003DMC400046501
22	StALDH22A1	PGSC0003DMG400004698	PGSC0003DMP400008325	PGSC0003DMT400011970	PGSC0003DMC400008325

Appendix 2. All the identified StALDH protein sequences

>StALDH2B1

MNPTTKAETNVNRPKSSTFLYCNSNHIASNCRKPSNLVMFEEDGEIDSFLLGGQTHQEYSNYWKKGIRRFCSSAVVHEEPITPPVEVKYNQLLIN
GQFVDAASGKTFPTFDPRTGEAITTVAEANTEDVNRAVSAARKAFDEGPWPKMTAYVGRIKFMVLQLLLTVCTMFRPCMSQLVLPDR

>StALDH2B2

MAARRLSSLLSRSLHLPSASASLGRSHGVARHINRFSTAAAVEELITPPVQVNHTKLLINGQFVDSASGKTFPTLDPRTGEVIANVAEGDLEDVN
RAVAAARKAFDEGPWPKMSAYERSRIMLKFADLVEKHNDEIAALETWDNGKPYLQAAQAEVPSFVRLFRYYAGWADKIHGLTVPADGPYHVQTLH
EPIGVAGQIIPWNFPLLMMAWKVGPALACGNTIVLKTAEQTPLTALYVANLFHEAGLPPGVLNIVSGFGPTAGAALASHMDVDKLAFTGSTETGQ
TVLQLAAKSNLKPVTLELGGKSPFIICEDADVDHAVELAHFALFFNQGQCCCAGSRTYVHERVYDEFVEKAKARAMRRVVGDPFKKGVEQGPQID
SEQFKKILRYIREGRDSSATLECGGDRIGSKGYFIQPTVFSNVKEDMSIAQDEIFGPVQCVFKFKDIGEVIKRANNTRYGLAAGVFTKNIDTANT
LTRGLRAGTVWINCYDIFDAGIPFGGYKMSGTGREKGIYSLNNYLQVKAVVTPLKNPAWI

>StALDH2B3

MTRSEQKNKCPSFSNTTAEMRFNGRKKYKEMLLAQKEALLLQRRTRDLESRRRLLQVPVMIREPYVTSTKGKAKACVMRPVVGDPFTKGVEQGPQ
INSGQFKKTLRCIREGRDSSAALECGDDTICSQSYFIQFKC

>StALDH2B4

MILVCFPSTTLSRKLLLSSPPSEIAPPKPHNNIVNDEDNNPKEKSNLKPVTLELGGKSPFIVCEDADIDTAVEQAHLALFFNQGQCCCAGSQTYV
HEKVYDEFLEKAKARTLKRVVGDPFKSGTEQGPQDDMLIAQDEIFGPVQSILKFKDLDEVVRRANSSRYGLAAGVFSQNIDTANTLARALRVGMV
WINCFDTFDATIPFGGYKMSGQGREKGEYDLRNYLQVKAVATALKNPTWL

>StALDH2B5

MLPYLWQRTETKRAKELSTTTNRLQQTRKRDLDEMVRRDNSSQYGLAAGVFSQNIDTANTLARASRVGMVWINCFDTFNSTIPFGGYKMSGQGRE
KGEYGLRNYLQVKSVVTPLKSPAWL

>StALDH2B6

MAARVFLSRSVHLLSKGKRSHLGRIAAYKYSTAAALEEPIKPTVNVDHTKLFINGQFVDSASGKTFPTLDPRTGEVIAHIAEGDAEDINRAVAAA
RKAFDEGPWPRMTAYERSKILLRLADLIEKHNDQIATLETWDTGKPYAQAAKIEVPMVVRLLRYYAGWADKIHGMTIPADGPYHVQTLHEPIGVA
GQIIPWNFPLLMFSWKIGPALACGNTIVLKTAEQTPLSALYVANLLQEAGLPEGVLNIISGFGATAGASLCSHMDVDKLAFTGSTETGKTILELA
AKSNLKPVTLELGGKSPFIVYEDADIDTAVEQAHFALFFNQGQCCCAGSRTYVHEKVYDEFLEKAKARALKRVVGDPFKSGTEQGPQIDSKQFDK
IMKYIRSGVDSGATLETGGEQFGKKGYYIKPTVFSNVKDDMLIAQDEIFGPVQSILKFKDLDEVVRRANSSRYGLAAGVFSQNIDTANTLARALR
VGTVWINCFDTFDATIPFGGYKMSGQGREKGEYGLRNYLQVKAVVTPLKNPAWL

>StALDH2B7

MGKGTGSFGKRRNKTHTLCVRCGRRSFHIQKSRCSACAYPAARLRKYNWSVKALRRKTTGTGRMRYLRNVPRRFKTNFREGRNSRVAATAALRYT
TAAPIAQDPIKPSVNVEYTKLFINGQFVDSTSGKTFPTLDPRTGEVIAHVAEGDVEDINRAVVAARNAFDEGPWPKMSAYERSKVLFRIADLIEK
HNDEIATLETWDSGKLYQQVATIEIPMIVRILRYYAGWADKIHGMTVPADGPYHVQTLHEPIGVVGQIIPWNFPLLMFAWKIGPALACGNTVVLK
TAEQTPLSALYVSKLLQEAGLPEGVVNVISGFGPTAGAALCSHMDVDKLAFTGSTDTGKTIMSLAANSNLKPVTLELGGKSPFIVCEDADVDQAV
EFAHFALFFNQGQCCCAGSRTYVHESIYDEFVEKAKARALKRTVGDPFESGNEQGPQISSEQFEKVLKYIRSGIESGATLETGGDRLGTRGYYIK
PTVFSNVKDDMLIATDEIFGPVQSILKFKDHDEVIRRANATKYGLAAGVFTKNIDTANTFMRALRVGTIWINCFDIFDAAIPFGGYKMSGQGREK
GEYSLKQYLQVKAVVTSLKNPAWL

>StALDH2C1

MAESNGNSETQFQIPKIKFTKLFINGEFVDSVSGNTFETIDPRNEEVIARISEGDKEDVDLAVKAAREAFDDGPWPRLSPSERRRIMLKFADLIV
ENAEEIAALDAMDAGKLFAPVKNMDIPAAAEVIRYYAGAADKIHGTTLKMSCELQGYTLLEPIGVVGHIIPWNFPTQMFVMKVGPALAAGCTMIV
KPAEQTPLSALYYAQLAKQAGVPDGVINVVTGFGSTAGAALCSHMDVDKISFTGSTEVGRLVMQAAALSNLKPVSLELGGKSPFIVFDDVDVDKV
APLALVGILYNKGEICVAGSRLFIQEGIYDKFVKKLEEMAKTWVVGDPFDPNSHQGPQVDKKQYERVLSYIEHGKREGAKLLTGGNALDRKGYFI
EPTIFIDVEDDMTIAKEEIFGPVLAVMKFKTVEEVIKRANCTNYGLAAGVMTNDLNIANTVSRSIRAGVIWINCYFAFDPDCPYGGYKCSGFERD
LGMEGLHKYLQVKSVATPIYNSPWL

>StALDH3F1

MGAAAKHLTPVTLELGGKCPVIIDSLSSSWDKKIAIKRILSGKFGSCAGQACIGIDYILVDKTFVNELVKLIKLGIPKMFGENPKESHSISRIVN
RNHFLRLKNLLDEPMVKKSVIYGGSSDEDNLYIEPTVLLDPPMQSTIMTEEIFGPLLPIITLDKIEDSIEFINARPKPLTIYAFTKNEEFKRKIT
KGTSSGSLVFNDTIIQYAADTLPFGGVGQSGFGRYHGKFSFDTFSHEKAIARRSFLTDIWFRYPPWSDHTLQLFKSAFIYDYLSIVLITLGLKKA

>StALDH3F2

MNGVEEDVLGELRTTFRSGRTRSVAWRKAQLQAILKLLDENEEEIFEALKQDLGKHPVESYRDEVGVVRKSATNALRCVEKWMAPQKAPIPLVLF
PARGAVVSEPLGVVLLFVSWNFPISLTLDPAIGAISAGNTIVLKPSELAPKCSSVLANTIPRYLDPEAIKVVEGGQDVSEQLLQLKWDKIFFTGS
PRVGRLIMSAAAKHLTPVTLELGGKCPTILDTLSSSYDLQVAVKRIAGGKWGPCNGQACIGIDYVLVETQFAPVLIELLEKIIKTFYGENLKTLG
NLARIVNKHHFDRVHNLLKDPKVAASVVYGGSVDEENMVIEPTILLNPPLDADIMTEEIFGPLLPIITLNNIEESIQFINSRPKPLAIYAFTKND
SLKEKILQETSSGSLTFNDAMIQFICDTLPFGGVGQSGYGRYHGKFSFDTFSHEKAVLHRSLLIELESRYPPWNNFKLEFVRLAYDYDYLGLILL
LLGLRGIFRTNRRQ

>StALDH3H1

MDAEAIVKELRGTYGTGKTKSYEWRVSQLKALFKIAENHEKEITDALYSDLSKPELEAFIHEISMMKTACKLALKELKRWMKPEKVKTSLTSFPS
SAEIVPEPLGVVLVISAWNYPFLLSLDPVIGAIAAGNAVVLKPSEIAPATSSVLAKLLGQYMDVSAIRVVEGAVPETTALLEQKWDKIFYTGNGK
VGRIVLAAAAKHLTPVVLELGGKSPVVVDSNIDYKIAVRRIIAGKWGCNNGQACISPDYIITTKENVPKLLDAMKQELEKFYGKDPLKSGDLSRI
VNANHFQRLSKLLDDKKVVDKVVHGGQRDEDNLKISPTILLDVPEDSLIMKEEIFGPLLPIITVNKVEDSIQFINAREKPLAAYLFTSNKKLEEE
FVMNISAGGLLINDTTLQVALSTLPFGGVGESGMGSCHGKFSFDSFSHKKAVLRRSFAGDVPARYPPYTTGKARFLKALLNGDILGLIRALIGW

>StALDH5F1

MALSACAMLHRSSISGPVRLMTTATQSIAAKLSSSGLLRSQALIGGKWVDAYDGKTIKVHNPATGEVITDVPCMGGRETNDAISSAYDAFSSWSK
LTAAERSKYLRKWYDLIMAHKEELGQLMTLEQGKPLKEAIGEVSYGAGFIEFSAEEGKRIYGDIIPSPLADRRLFVLKQPVGVVGAITPWNFPLA
MITRKVGPALACGCTVVIKPSELTPLTALAAAELSIQAGIPPGVVNVVMGNAPAIGDALLASPQVRKITFTGSTKVGKKLMEGAAATVKKVSLEL
GGNAPCIIFDDADLEVALKGALATKFRNTGQTCVCANRILVQEGIYDKFANAFAKAVQNMKVGDGFTEGVEQGPLINEAAVQKVESFVEEATSKG
AKVLVGGKRHSLGMTFYEPTVVTGVNSEMLLAKEEVFGPVAPLLKFKTDEEAIQMANDTNAGLAAYIFSTNIKRAWRVTEALEYGIVGVNEGLVS
TEVAPFGGVKQSGLGREGSKYGMDEYLEMKYVCLGSMS

>StALDH6B1

MMQFSVHRVRKVRSLTPGIFALANHHFSVATESSWKHRTSLRVPNLIGGSFVDSQSSEFVDVINPATQEVVSQIPLTTDKEFKSAVSAAKEAFPS
WKNTPITTRQRVMLKFQELIRKNMDKLAFNVTTEQGKTLKDAQGDVFRGLEVVEHACGMATLQMGEYVSNVSNGIDTYSLREPLGVCAGICPFNF
PAMIPLWMFPVAATCGNTFILKPSEKDPGASMMLAELAMEAGLPDGVLNIVHGTHDVVNAICDDDDIRAISFVGSNTAGMHIYSRASAKGKRVQS
NMGAKNHGVVMPDANIDSTINALVAAGFGAAGQRCMALSTVVFVGDSKPWEEKLLERAKTLKVSAGTEPDADLGPVISKQAKERVCQLVQSGVDS
GAKLLLDGRDIVVPGYEKGNFVGPTILSGVTPDMECYKEEIFGPVLLCMQANSLDEAINIVNQNKYGNGAAIFTTSGVAARRFQTEIESGQIGIN
VPIPVPLPFFSFTGSKASFAGDLNFYGKAGVQFYTQIKTVTQQWKDLSGGSGVSLAMPTSQK

>StALDH6B2

MGTKNYAVVMPYTNVEATLNVLVAAGFGVAGERCTTISTGLCWRLKITVKERISKLIQAIVDSGAKLVLDGRQVAVPKLLSSLLNICG

>StALDH7A1

MTSFTKKEYEFLKELGIGPQNLGCYVNGTWKATGPVISTFNPANNQIIAEVVEASAQDYEEGMSACAEAAKIWVQVPAPKRGEIVRQIGDALRAN
LQEFGRLVSLEMGKILPEGIGEVQEVIDMCDFAVGLSRQLNGSVIPSERPNHMMLEMWNPLGIVGVITAFNFPCAVLGWNACIALVCGNCVVWKG
APTTPLVTIAMTKIVASVLEKNNLPGSIFTAFCGGADVGQAIAKDTRIPLVSFTGSSKVGLAVQQTVSQRFGKCLLELSGNNAIIIMDDADIKLA
VRSVLFAAVGTAGQRCTTCRRLLVHESIYEKVLEPLVDVYKQVKIGDPLEKGTLLGPLHTCTSRENFEKGIHNIKSQGGKILTGGSVVESEGNFV
HPTIVEISSKAEIVKEELFAPVLYVMKFKTFEEAVEINNSVPQGLSSSIFTRNPENIFKWIGPQGSDCGIVNVNIPTNGAEIGGAFGGEKGTGGG
REAGSDSWKQYMRRSTCTINYGSELPLAQGINFG

>StALDH10A1

MAIPNIRIPCRQLFIDGEWREPLKKNRLPIINPANEEIIGYIPAATEEDVDIAVKAARSALRRDDWGSTTGAQRAKYLRAIAAKVLEKKPELATL
ETIDNGKPWFEAASDIDDVVACFEYYADLAEALDSKKKTEVKLHLDSFKTHVLREPLGVVGLITPWNYPLLMTTWKVAPALAAGCAAILKPSELA
SITSLELGEICREVGLPPGALSILTGLGHEAGSPLVSHPDVDKIAFTGSGPTGVKIMTAAAQLVKPVTLELGGKSPIVVFDDIHDLDIAVEWTLF
GCFWTNGQICSATSRLIIQETIAPQFLARLLEWTKNIKISDPLEEDCKLGPVISRGQYEKVLKFISTAKDEGATILYGGDRPEHLKKGYYIQPTI
ITDVDTSMEIWNEEVFGPVLCVKTFKTEEEAIELANDTKYGLGAAILSKDLERCERFTKAFQSGVVWINCSQPCFWQPPWGGKKRSGFGRELGEW
SLENYLNIKQVTQYVTPDEPWAFYKSPSKL

>StALDH10A2

MANRNVPISRRQLYIGGEWREPVKKNRIPIINPATEEIIGDIPAATAEDVDIAVEAARKAIARDDWGSTTGAQRAKYLRAIAAKVLEKKSVLATL
ESLDSGKTLFESAADMDDVAGCFEYYADLAEALDSRRKTPVNLNSDSFKTYVLREPLGVVGLITPWNYPLLMAIWKVAPALAAGCAAILKPSELA
SVTCLELGEICREIGLPSGALNILTGLGPEAGGPLASHPHVDKISFTGSGPTGSKIMTAAAQLVKPVSLELGGKSPIVVFDDIDNLDIAAEWTLF
GIFANTGQVCSATSRLIVQESIASAFLDRLLKWTKNIKISDPLEEDCKLGPVVSAGQYEKVLKFISNAKSEGATILYGGKRPQHLKKGYYVQPTI
ITDVNTSMEIWKEEVFGPVLCVKTFKTEEEAIELANDTKYGLAAAVMSKDVKRCERFTKAFQTGIIWINCSQPTFNQLPWGGKKRSGFGRDLGEW
GLESFLNIKQVTEYTSAEPWAFYKSPSRN

>StALDH11A1

MAGNGVFAEIIDGEVYKYYCEGEWRKSASGKSVAIINPTTRKTQYKVQACTQEEVNKVMEIAKAAQKSWAKTPLWKRAELLHKAAAILKEHKAPI
AECLVKEIAKPAKDAVTEVVRSGDLVSYTAEEGVRILGEGKFLVSDSFPGNERTKYCLTSKIPLGVILAIPPFNYPVNLAVSKIAPALIAGNSLV
LKPPTQGAVAALHMVHCFHLAGFPKGLISCVTGKGSEIGDFLTMHPGVNCISFTGGDTGVAISKKAGMVPLQMELGGKDACIVLEDADLDLAAGN
IVKGGFSYSGQRCTAVKVVLVMESVADTLVEKVNAKVAKLTVGPPEDNCDITPVVSESSANFIEGLVMDAKEKDATFCQPYKREGNLIWPLLLDN
VRPDMRIAWEEPFGPVLPVIRINSVEEGIHHCNASNFGLQGCVFTKDINKAILISDAMETGTVQINSAPARGPDHFPFQGIKDSGIGSQGITNSI
NMMTKVKTTVINLPTPSYTMG

>StALDH12A1

MYRLSAYRQLKNRASSSHLNWITLFNSTRSNHTLSFATVKAEEVSGSQPAEVHNLVQGKWTKSSSWNTILDPLNGQPFIKVAEVNESELQPFVES
LSKCPKHGLHNPFKAPERYLMLGDVSTKAAHALGLPEVSDFFAKLIQRVSPKSYQQALIEVLVTQKFLENFCGDQVRFLARSFAVPGNHLGQQSH
GFRWPYGPVAVIAPFNFPLEIPLLQLMGALYMGNKPVLKVDSKVCIVMEQMLRLLHECGLPVDDVDFINSDGKTMNKLLVEAKPRMTLFTGSSRV
AEKLADDLSGRVKLEDAGFDWKILGPDVNEVDYVAWVCDQDAYACSGQKCSAESILFMHENWSKSSLIDKMTELAARRKLDDLTIGPVLTVTTET
MLDHAKKLLQIPGSRLLFGGEALQNHSIPKIYGAIKPTAIFVPLEEILKDEHYPLVTKEIFGPFQVVTEYKDNQLPLVLDALEKMHAHLTAAVVS
NDILFLQKVIGNSVNGTTYAGLRARTTGAPQNHWFGPAGDPRGAGIGTPEAIKLVWSCHREIIYDVGPMPLGWKVPAST

>StALDH18A1

MDSADPARAFVKDVKRIIIKVGTAVVTRGDGRLALGRMGSLCEQIRELTSQGFEVILVTSGAVGVGRQRLRYRKLINSSFADLQKPQGDLDGKAC
AAVGQNGLMALYDTLFSQLDVTSAQLMVTDNDFRDPDFRRQLNETVNSLLCLKVVPIFNENDAISTRKAPYEDSSGIFWDNDSLAALLAMELKAD
LLVLLSDVEGLYTGPPSDPQSELIHTYVKEKHEGLITFGDKSRVGRGGMTAKVKAAVYAAYAGIPVVITSGFANNNIIKALDGQRVGTLFHREAI
KWASIGDFDAREMAVSARECARRLQTLSSQERSKILLDIADALEAKEEEILAENEADVAAAQQSGYENSLISRLAMKPGKISSLANSVRVLANMD
EPVGRILKRTELADGIILEKTSSPLGVLLIIFESRPDALVQIASLAVRSGNGLLLKGGKEAKRSNAILHKVITSSIPPTVGERLIGLVTSREEIP
ELLKLDDVIDLVIPRGSNKLVSQIKAATKIPVLGHADGICHVFIDKSADLDMAKRIVLDAKTDYPAACNAMETLLVHEDLVQTGGLNDLILELQV
KGVSLFGGPKASSVLSIPEANSFHHEYGALACTVEIVEDVNTAIEHIHRHGSAHTDSIITEDKEVAELFLRQVDSAAVLHNASTRFSDGFRFGLG
AEVGISTSRIHARGPVGVEGLLTTRWLARGSGQVVDGDKEIVYTHRDLNLEA

>StALDH18A2

MDSADPARAFVKDVKRIIIKVGTAVVTRGDGRLALGRMGSLCEQIRELTSQGFEVILVTSGAVGVGRQRLRYRKLINSSFADLQKPQGDLDGKAC
AAVGQNGLMALYDTLFSQLDVTSAQLMVTDNDFRDPDFRRQLNETVNSLLCLKVVPIFNENDAISTRKAPYEDSSGIFWDNDSLAALLAMELKAD
LLVLLSDVEGLYTGPPSDPQSELIHTYVKEKHEGLITFGDKSRVGRGGMTAKVKAAVYAAYAGIPVVITSGFANNNIIKALDGQRVGTLFHREAI
KWASIGDFDAREMAVSARECARRLQTLSSQERSKILLDIADALEAKEEEILAENEADVAAAQQSGYENSLISRLAMKPGKISSLANSVRVLANMD

EPVGRILKRTELADGIILEKTSSPLGVLLIIFESRPDALVQIASLAVRSGNGLLLKGGKEAKRSNAILHKVITSSIPPTVGERLIGLVTSREEIP
ELLKLDDVIDLVIPRGSNKLVSQIKAATKIPVLGHADGICHVFIDKSADLDMAKRIVLDAKTDYPAACNAMETLLVHEDLVQTGGLNDLILELQV
KGVSLFGGPKASSVLSIPEANSFHHEYGALACTVEIVEDVNTAIEHIHRHGRQVTFLITIQF

>StALDH22A1

MAFWWPLIVIAIAFAICKLLLMLIPDNVPSIDVDTSDVLDDGNQAKDNSFIYIPSRRHTDKVQCYEPATMKYLGYFPALKPDEVKERVVQARKAQ
KIWAKSSFKQRRLFLRILLKYIIEHQDLICNISSRDTGKTMVDASLGEIMTTCEKIHWLLSEGEKWLKPEYRSCGRSMLHKVAKVEFSPFGVVGA
IVSWNYPFHNIFNPMLAAVFSGNSIVIKVSEHASWSGCFYLRIIQTALAAVGAPENLVEVITGFAETGEALVSSVDKIIFVGSPGVGKKIMRSAS
NTLIPVTLELGGKDAFIVCEDVDVPHVAQIAARGALQSSGQNCAGAERFYVHKDVYSSFVAEIVKIVKSVTAGPPLSGKYDMGAICMQEHSERLQ
YLVNDALDKGAEIVARGSVGNIGEGAVDQYFPPTVIVNVNHTMKLMQEEAFGPILPIMKFSSDEEVVQLANDSSYGLGCAVFSGSQRRARHIASQ
LHCGVAAINDFASNYMCQSLPFGGVKDSGFGRFAGIEGLRACCLVKSVVEDRWWPFIKTKIPKPIQYPIAENGFEFQESLVHTLYGLNIWDRLRA
LVNVLKILSQQPPAPTSNRRRND

Appendix 3. All the sequences of used for phylogenetic tree construction

>StALDH2B1
MNPTTKAETNVNRPKSSTFLYCNSNHIASNCRKPSNLVMFEEDGEIDSFLLGGQTHQEYSNYWKKGIRRFCSSAVVHEEPITPPVEVKYNQLLINGQ
FVDAASGKTFPTFDPRTGEAITTVAEANTEDVNRAVSAARKAFDEGPWPKMTAYVGRIKFMVLQLLLTVCTMFRPCMSQLVLPDR
>StALDH2B2
MAARRLSSLLSRSLHLPSASASLGRSHGVARHINRFSTAAAVEELITPPVQVNHTKLLIN
GQFVDSASGKTFPTLDPRTGEVIANVAEGDLEDVNRAVAAARKAFDEGPWPKMSAYERSRIMLKFADLVEKHNDEIAALETWDNGKPYLQAAQAEVP
SFVRLFRYYAGWADKIHGLTVPADGPYHVQTLHEPIGVAGQIIPWNFPLLMMAWKVGPALACGNTIVLKTAEQTPLTALYVANLFHEAGLPPGVLNI
VSGFGPTAGAALASHMDVDKLAFTGSTETGQTVLQLAAKSNLKPVTLELGGKSPFIICEDADVDHAVELAHFALFFNQGQCCCAGSRTYVHERVYDE
FVEKAKARAMRRVVGDPFKKGVEQGPQIDSEQFKKILRYIREGRDSSATLECGGDRIGSKGYFIQPTVFSNVKEDMSIAQDEIFGPVQCVFKFKDIG
EVIKRANNTRYGLAAGVFTKNIDTANTLTRGLRAGTVWINCYDIFDAGIPFGGYKMSGTGREKGIYSLNNYLQVKAVVTPLKNPAWI
>StALDH2B3
MTRSEQKNKCPSFSNTTAEMRFNGRKKYKEMLLAQKEALLLQRRTRDLESRRRLLQVPVMIREPYVTSTKGKAKACVMRPVVGDPFTKGVEQGPQIN
SGQFKKTLRCIREGRDSSAALECGDDTICSQSYFIQFKC
>StALDH2B4
MILVCFPSTTLSRKLLLSSPPSEIAPPKPHNNIVNDEDNNPKEKSNLKPVTLELGGKSPFIVCEDADIDTAVEQAHLALFFNQGQCCCAGSQTYVHE
KVYDEFLEKAKARTLKRVVGDPFKSGTEQGPQDDMLIAQDEIFGPVQSILKFKDLDEVVRRANSSRYGLAAGVFSQNIDTANTLARALRVGMVWINC
FDTFDATIPFGGYKMSGQGREKGEYDLRNYLQVKAVATALKNPTWL
>StALDH2B5
MLPYLWQRTETKRAKELSTTTNRLQQTRKRDLDEMVRRDNSSQYGLAAGVFSQNIDTANTLARASRVGMVWINCFDTFNSTIPFGGYKMSGQGREKG
EYGLRNYLQVKSVVTPLKSPAWL
>StALDH2B6
MAARVFLSRSVHLLSKGKRSHLGRIAAYKYSTAAALEEPIKPTVNVDHTKLFINGQFVDSASGKTFPTLDPRTGEVIAHIAEGDAEDINRAVAAARK
AFDEGPWPRMTAYERSKILLRLADLIEKHNDQIATLETWDTGKPYAQAAKIEVPMVVRLLRYYAGWADKIHGMTIPADGPYHVQTLHEPIGVAGQII
PWNFPLLMFSWKIGPALACGNTIVLKTAEQTPLSALYVANLLQEAGLPEGVLNIISGFGATAGASLCSHMDVDKLAFTGSTETGKTILELAAKSNLK
PVTLELGGKSPFIVYEDADIDTAVEQAHFALFFNQGQCCCAGSRTYVHEKVYDEFLEKAKARALKRVVGDPFKSGTEQGPQIDSKQFDKIMKYIRSG
VDSGATLETGGEQFGKKGYYIKPTVFSNVKDDMLIAQDEIFGPVQSILKFKDLDEVVRRANSSRYGLAAGVFSQNIDTANTLARALRVGTVWINCFD
TFDATIPFGGYKMSGQGREKGEYGLRNYLQVKAVVTPLKNPAWL
>StALDH2B7
MGKGTGSFGKRRNKTHTLCVRCGRRSFHIQKSRCSACAYPAARLRKYNWSVKALRRKTTGTGRMRYLRNVPRRFKTNFREGRNSRVAATAALRYTTA
APIAQDPIKPSVNVEYTKLFINGQFVDSTSGKTFPTLDPRTGEVIAHVAEGDVEDINRAVVAARNAFDEGPWPKMSAYERSKVLFRIADLIEKHNDE
IATLETWDSGKLYQQVATIEIPMIVRILRYYAGWADKIHGMTVPADGPYHVQTLHEPIGVVGQIIPWNFPLLMFAWKIGPALACGNTVVLKTAEQTP
LSALYVSKLLQEAGLPEGVVNVISGFGPTAGAALCSHMDVDKLAFTGSTDTGKTIMSLAANSNLKPVTLELGGKSPFIVCEDADVDQAVEFAHFALF
FNQGQCCCAGSRTYVHESIYDEFVEKAKARALKRTVGDPFESGNEQGPQISSEQFEKVLKYIRSGIESGATLETGGDRLGTRGYYIKPTVFSNVKDD
MLIATDEIFGPVQSILKFKDHDEVIRRANATKYGLAAGVFTKNIDTANTFMRALRVGTIWINCFDIFDAAIPFGGYKMSGQGREKGEYSLKQYLQVK
AVVTSLKNPAWL
>StALDH2C1
MAESNGNSETQFQIPKIKFTKLFINGEFVDSVSGNTFETIDPRNEEVIARISEGDKEDVDLAVKAAREAFDDGPWPRLSPSERRRIMLKFADLIVEN
AEEIAALDAMDAGKLFAPVKNMDIPAAAEVIRYYAGAADKIHGTTLKMSCELQGYTLLEPIGVVGHIIPWNFPTQMFVMKVGPALAAGCTMIVKPAE
QTPLSALYYAQLAKQAGVPDGVINVVTGFGSTAGAALCSHMDVDKISFTGSTEVGRLVMQAAALSNLKPVSLELGGKSPFIVFDDVDVDKVAPLALV
GILYNKGEICVAGSRLFIQEGIYDKFVKKLEEMAKTWVVGDPFDPNSHQGPQVDKKQYERVLSYIEHGKREGAKLLTGGNALDRKGYFIEPTIFIDV
EDDMTIAKEEIFGPVLAVMKFKTVEEVIKRANCTNYGLAAGVMTNDLNIANTVSRSIRAGVIWINCYFAFDPDCPYGGYKCSGFERDLGMEGLHKYL
QVKSVATPIYNSPWL
>StALDH3F1
MGAAAKHLTPVTLELGGKCPVIIDSLSSSWDKKIAIKRILSGKFGSCAGQACIGIDYILVDKTFVNELVKLIKLGIPKMFGENPKESHSISRIVNRN
HFLRLKNLLDEPMVKKSVIYGGSSDEDNLYIEPTVLLDPPMQSTIMTEEIFGPLLPIITLDKIEDSIEFINARPKPLTIYAFTKNEEFKRKITKGTS
SGSLVFNDTIIQYAADTLPFGGVGQSGFGRYHGKFSFDTFSHEKAIARRSFLTDIWFRYPPWSDHTLQLFKSAFIYDYLSIVLITLGLKKA
>StALDH3F2
MNGVEEDVLGELRTTFRSGRTRSVAWRKAQLQAILKLLDENEEEIFEALKQDLGKHPVESYRDEVGVVRKSATNALRCVEKWMAPQKAPIPLVLFPA
RGAVVSEPLGVVLLFVSWNFPISLTLDPAIGAISAGNTIVLKPSELAPKCSSVLANTIPRYLDPEAIKVVEGGQDVSEQLLQLKWDKIFFTGSPRVG
RLIMSAAAKHLTPVTLELGGKCPTILDTLSSSYDLQVAVKRIAGGKWGPCNGQACIGIDYVLVETQFAPVLIELLEKIIKTFYGENLKTLGNLARIV
NKHHFDRVHNLLKDPKVAASVVYGGSVDEENMVIEPTILLNPPLDADIMTEEIFGPLLPIITLNNIEESIQFINSRPKPLAIYAFTKNDSLKEKILQ
ETSSGSLTFNDAMIQFICDTLPFGGVGQSGYGRYHGKFSFDTFSHEKAVLHRSLLIELESRYPPWNNFKLEFVRLAYDYDYLGLILLLLGLRGIFRT
NRRQ
>StALDH3H1
MDAEAIVKELRGTYGTGKTKSYEWRVSQLKALFKIAENHEKEITDALYSDLSKPELEAFIHEISMMKTACKLALKELKRWMKPEKVKTSLTSFPSSA
EIVPEPLGVVLVISAWNYPFLLSLDPVIGAIAAGNAVVLKPSEIAPATSSVLAKLLGQYMDVSAIRVVEGAVPETTALLEQKWDKIFYTGNGKVGRI
VLAAAAKHLTPVVLELGGKSPVVVDSNIDYKIAVRRIIAGKWGCNNGQACISPDYIITTKENVPKLLDAMKQELEKFYGKDPLKSGDLSRIVNANHF
QRLSKLLDDKKVVDKVVHGGQRDEDNLKISPTILLDVPEDSLIMKEEIFGPLLPIITVNKVEDSIQFINAREKPLAAYLFTSNKKLEEEFVMNISAG
GLLINDTTLQVALSTLPFGGVGESGMGSCHGKFSFDSFSHKKAVLRRSFAGDVPARYPPYTTGKARFLKALLNGDILGLIRALIGW
>StALDH5F1
MALSACAMLHRSSISGPVRLMTTATQSIAAKLSSSGLLRSQALIGGKWVDAYDGKTIKVHNPATGEVITDVPCMGGRETNDAISSAYDAFSSWSKLT
AAERSKYLRKWYDLIMAHKEELGQLMTLEQGKPLKEAIGEVSYGAGFIEFSAEEGKRIYGDIIPSPLADRRLFVLKQPVGVVGAITPWNFPLAMITR
KVGPALACGCTVVIKPSELTPLTALAAAELSIQAGIPPGVVNVVMGNAPAIGDALLASPQVRKITFTGSTKVGKKLMEGAAATVKKVSLELGGNAPC

IIFDDADLEVALKGALATKFRNTGQTCVCANRILVQEGIYDKFANAFAKAVQNMKVGDGFTEGVEQGPLINEAAVQKVESFVEEATSKGAKVLVGGK
RHSLGMTFYEPTVVTGVNSEMLLAKEEVFGPVAPLLKFKTDEEAIQMANDTNAGLAAYIFSTNIKRAWRVTEALEYGIVGVNEGLVSTEVAPFGGVK
QSGLGREGSKYGMDEYLEMKYVCLGSMS
>StALDH6B1
MMQFSVHRVRKVRSLTPGIFALANHHFSVATESSWKHRTSLRVPNLIGGSFVDSQSSEFVDVINPATQEVVSQIPLTTDKEFKSAVSAAKEAFPSWK
NTPITTRQRVMLKFQELIRKNMDKLAFNVTTEQGKTLKDAQGDVFRGLEVVEHACGMATLQMGEYVSNVSNGIDTYSLREPLGVCAGICPFNFPAMI
PLWMFPVAATCGNTFILKPSEKDPGASMMLAELAMEAGLPDGVLNIVHGTHDVVNAICDDDDIRAISFVGSNTAGMHIYSRASAKGKRVQSNMGAKN
HGVVMPDANIDSTINALVAAGFGAAGQRCMALSTVVFVGDSKPWEEKLLERAKTLKVSAGTEPDADLGPVISKQAKERVCQLVQSGVDSGAKLLLDG
RDIVVPGYEKGNFVGPTILSGVTPDMECYKEEIFGPVLLCMQANSLDEAINIVNQNKYGNGAAIFTTSGVAARRFQTEIESGQIGINVPIPVPLPFF
SFTGSKASFAGDLNFYGKAGVQFYTQIKTVTQQWKDLSGGSGVSLAMPTSQK
>StALDH6B2
MGTKNYAVVMPYTNVEATLNVLVAAGFGVAGERCTTISTGLCWRLKITVKERISKLIQAIVDSGAKLVLDGRQVAVPKLLSSLLNICG
>StALDH7A1
MTSFTKKEYEFLKELGIGPQNLGCYVNGTWKATGPVISTFNPANNQIIAEVVEASAQDYEEGMSACAEAAKIWVQVPAPKRGEIVRQIGDALRANLQ
EFGRLVSLEMGKILPEGIGEVQEVIDMCDFAVGLSRQLNGSVIPSERPNHMMLEMWNPLGIVGVITAFNFPCAVLGWNACIALVCGNCVVWKGAPTT
PLVTIAMTKIVASVLEKNNLPGSIFTAFCGGADVGQAIAKDTRIPLVSFTGSSKVGLAVQQTVSQRFGKCLLELSGNNAIIIMDDADIKLAVRSVLF
AAVGTAGQRCTTCRRLLVHESIYEKVLEPLVDVYKQVKIGDPLEKGTLLGPLHTCTSRENFEKGIHNIKSQGGKILTGGSVVESEGNFVHPTIVEIS
SKAEIVKEELFAPVLYVMKFKTFEEAVEINNSVPQGLSSSIFTRNPENIFKWIGPQGSDCGIVNVNIPTNGAEIGGAFGGEKGTGGGREAGSDSWKQ
YMRRSTCTINYGSELPLAQGINFG
>StALDH10A1
MAIPNIRIPCRQLFIDGEWREPLKKNRLPIINPANEEIIGYIPAATEEDVDIAVKAARSALRRDDWGSTTGAQRAKYLRAIAAKVLEKKPELATLET
IDNGKPWFEAASDIDDVVACFEYYADLAEALDSKKKTEVKLHLDSFKTHVLREPLGVVGLITPWNYPLLMTTWKVAPALAAGCAAILKPSELASITS
LELGEICREVGLPPGALSILTGLGHEAGSPLVSHPDVDKIAFTGSGPTGVKIMTAAAQLVKPVTLELGGKSPIVVFDDIHDLDIAVEWTLFGCFWTN
GQICSATSRLIIQETIAPQFLARLLEWTKNIKISDPLEEDCKLGPVISRGQYEKVLKFISTAKDEGATILYGGDRPEHLKKGYYIQPTIITDVDTSM
EIWNEEVFGPVLCVKTFKTEEEAIELANDTKYGLGAAILSKDLERCERFTKAFQSGVVWINCSQPCFWQPPWGGKKRSGFGRELGEWSLENYLNIKQ
VTQYVTPDEPWAFYKSPSKL
>StALDH10A2
MANRNVPISRRQLYIGGEWREPVKKNRIPIINPATEEIIGDIPAATAEDVDIAVEAARKAIARDDWGSTTGAQRAKYLRAIAAKVLEKKSVLATLES
LDSGKTLFESAADMDDVAGCFEYYADLAEALDSRRKTPVNLNSDSFKTYVLREPLGVVGLITPWNYPLLMAIWKVAPALAAGCAAILKPSELASVTC
LELGEICREIGLPSGALNILTGLGPEAGGPLASHPHVDKISFTGSGPTGSKIMTAAAQLVKPVSLELGGKSPIVVFDDIDNLDIAAEWTLFGIFANT
GQVCSATSRLIVQESIASAFLDRLLKWTKNIKISDPLEEDCKLGPVVSAGQYEKVLKFISNAKSEGATILYGGKRPQHLKKGYYVQPTIITDVNTSM
EIWKEEVFGPVLCVKTFKTEEEAIELANDTKYGLAAAVMSKDVKRCERFTKAFQTGIIWINCSQPTFNQLPWGGKKRSGFGRDLGEWGLESFLNIKQ
VTEYTSAEPWAFYKSPSRN
>StALDH11A1
MAGNGVFAEIIDGEVYKYYCEGEWRKSASGKSVAIINPTTRKTQYKVQACTQEEVNKVMEIAKAAQKSWAKTPLWKRAELLHKAAAILKEHKAPIAE
CLVKEIAKPAKDAVTEVVRSGDLVSYTAEEGVRILGEGKFLVSDSFPGNERTKYCLTSKIPLGVILAIPPFNYPVNLAVSKIAPALIAGNSLVLKPP
TQGAVAALHMVHCFHLAGFPKGLISCVTGKGSEIGDFLTMHPGVNCISFTGGDTGVAISKKAGMVPLQMELGGKDACIVLEDADLDLAAGNIVKGGF
SYSGQRCTAVKVVLVMESVADTLVEKVNAKVAKLTVGPPEDNCDITPVVSESSANFIEGLVMDAKEKDATFCQPYKREGNLIWPLLLDNVRPDMRIA
WEEPFGPVLPVIRINSVEEGIHHCNASNFGLQGCVFTKDINKAILISDAMETGTVQINSAPARGPDHFPFQGIKDSGIGSQGITNSINMMTKVKTTV
INLPTPSYTMG
>StALDH12A1
MYRLSAYRQLKNRASSSHLNWITLFNSTRSNHTLSFATVKAEEVSGSQPAEVHNLVQGKWTKSSSWNTILDPLNGQPFIKVAEVNESELQPFVESLS
KCPKHGLHNPFKAPERYLMLGDVSTKAAHALGLPEVSDFFAKLIQRVSPKSYQQALIEVLVTQKFLENFCGDQVRFLARSFAVPGNHLGQQSHGFRW
PYGPVAVIAPFNFPLEIPLLQLMGALYMGNKPVLKVDSKVCIVMEQMLRLLHECGLPVDDVDFINSDGKTMNKLLVEAKPRMTLFTGSSRVAEKLAD
DLSGRVKLEDAGFDWKILGPDVNEVDYVAWVCDQDAYACSGQKCSAESILFMHENWSKSSLIDKMTELAARRKLDDLTIGPVLTVTTETMLDHAKKL
LQIPGSRLLFGGEALQNHSIPKIYGAIKPTAIFVPLEEILKDEHYPLVTKEIFGPFQVVTEYKDNQLPLVLDALEKMHAHLTAAVVSNDILFLQKVI
GNSVNGTTYAGLRARTTGAPQNHWFGPAGDPRGAGIGTPEAIKLVWSCHREIIYDVGPMPLGWKVPAST
>StALDH18A1
MDSADPARAFVKDVKRIIIKVGTAVVTRGDGRLALGRMGSLCEQIRELTSQGFEVILVTSGAVGVGRQRLRYRKLINSSFADLQKPQGDLDGKACAA
VGQNGLMALYDTLFSQLDVTSAQLMVTDNDFRDPDFRRQLNETVNSLLCLKVVPIFNENDAISTRKAPYEDSSGIFWDNDSLAALLAMELKADLLVL
LSDVEGLYTGPPSDPQSELIHTYVKEKHEGLITFGDKSRVGRGGMTAKVKAAVYAAYAGIPVVITSGFANNNIIKALDGQRVGTLFHREAIKWASIG
DFDAREMAVSARECARRLQTLSSQERSKILLDIADALEAKEEEILAENEADVAAAQQSGYENSLISRLAMKPGKISSLANSVRVLANMDEPVGRILK
RTELADGIILEKTSSPLGVLLIIFESRPDALVQIASLAVRSGNGLLLKGGKEAKRSNAILHKVITSSIPPTVGERLIGLVTSREEIPELLKLDDVID
LVIPRGSNKLVSQIKAATKIPVLGHADGICHVFIDKSADLDMAKRIVLDAKTDYPAACNAMETLLVHEDLVQTGGLNDLILELQVKGVSLFGGPKAS
SVLSIPEANSFHHEYGALACTVEIVEDVNTAIEHIHRHGSAHTDSIITEDKEVAELFLRQVDSAAVLHNASTRFSDGFRFGLGAEVGISTSRIHARG
PVGVEGLLTTRWLARGSGQVVDGDKEIVYTHRDLNLEA
>StALDH18A2
MDSADPARAFVKDVKRIIIKVGTAVVTRGDGRLALGRMGSLCEQIRELTSQGFEVILVTSGAVGVGRQRLRYRKLINSSFADLQKPQGDLDGKACAA
VGQNGLMALYDTLFSQLDVTSAQLMVTDNDFRDPDFRRQLNETVNSLLCLKVVPIFNENDAISTRKAPYEDSSGIFWDNDSLAALLAMELKADLLVL
LSDVEGLYTGPPSDPQSELIHTYVKEKHEGLITFGDKSRVGRGGMTAKVKAAVYAAYAGIPVVITSGFANNNIIKALDGQRVGTLFHREAIKWASIG
DFDAREMAVSARECARRLQTLSSQERSKILLDIADALEAKEEEILAENEADVAAAQQSGYENSLISRLAMKPGKISSLANSVRVLANMDEPVGRILK
RTELADGIILEKTSSPLGVLLIIFESRPDALVQIASLAVRSGNGLLLKGGKEAKRSNAILHKVITSSIPPTVGERLIGLVTSREEIPELLKLDDVID
LVIPRGSNKLVSQIKAATKIPVLGHADGICHVFIDKSADLDMAKRIVLDAKTDYPAACNAMETLLVHEDLVQTGGLNDLILELQVKGVSLFGGPKAS
SVLSIPEANSFHHEYGALACTVEIVEDVNTAIEHIHRHGRQVTFLITIQF
>StALDH22A1

MAFWWPLIVIAIAFAICKLLLMLIPDNVPSIDVDTSDVLDDGNQAKDNSFIYIPSRRHTDKVQCYEPATMKYLGYFPALKPDEVKERVVQARKAQKI
WAKSSFKQRRLFLRILLKYIIEHQDLICNISSRDTGKTMVDASLGEIMTTCEKIHWLLSEGEKWLKPEYRSCGRSMLHKVAKVEFSPFGVVGAIVSW
NYPFHNIFNPMLAAVFSGNSIVIKVSEHASWSGCFYLRIIQTALAAVGAPENLVEVITGFAETGEALVSSVDKIIFVGSPGVGKKIMRSASNTLIPV
TLELGGKDAFIVCEDVDVPHVAQIAARGALQSSGQNCAGAERFYVHKDVYSSFVAEIVKIVKSVTAGPPLSGKYDMGAICMQEHSERLQYLVNDALD
KGAEIVARGSVGNIGEGAVDQYFPPTVIVNVNHTMKLMQEEAFGPILPIMKFSSDEEVVQLANDSSYGLGCAVFSGSQRRARHIASQLHCGVAAIND
FASNYMCQSLPFGGVKDSGFGRFAGIEGLRACCLVKSVVEDRWWPFIKTKIPKPIQYPIAENGFEFQESLVHTLYGLNIWDRLRALVNVLKILSQQP
PAPTSNRRRND
>AtALDH2C4
MENGKCNGATTVKLPEIKFTKLFINGQFIDAASGKTFETIDPRNGEVIATIAEGDKEDVDLAVNAARYAFDHGPWPRMTGFERAKLINKFADLIEEN
IEELAKLDAVDGGKLFQLGKYADIPATAGHFRYNAGAADKIHGETLKMTRQSLFGYTLKEPIGVVGNIIPWNFPSIMFATKVAPAMAAGCTMVVKPA
EQTSLSALFYAHLSKEAGIPDGVLNIVTGFGSTAGAAIASHMDVDKVSFTGSTDVGRKIMQAAAASNLKKVSLELGGKSPLLIFNDADIDKAADLAL
LGCFYNKGEICVASSRVFVQEGIYDKVVEKLVEKAKDWTVGDPFDSTARQGPQVDKRQFEKILSYIEHGKNEGATLLTGGKAIGDKGYFIQPTIFAD
VTEDMKIYQDEIFGPVMSLMKFKTVEEGIKCANNTKYGLAAGILSQDIDLINTVSRSIKAGIIWVNCYFGFDLDCPYGGYKMSGNCRESGMDALDNY
LQTKSVVMPLHNSPWM
>AtALDH2B4
MAARRVSSLLSRSFSASSPLLFRSQGRNCYNGGILRRFGTSSAAAEEIINPSVQVSHTQLLINGNFVDSASGKTFPTLDPRTGEVIAHVAEGDAEDI
NRAVKAARTAFDEGPWPKMSAYERSRVLLRFADLVEKHSEELASLETWDNGKPYQQSLTAEIPMFARLFRYYAGWADKIHGLTIPADGNYQVHTLHE
PIGVAGQIIPWNFPLLMFAWKVGPALACGNTIVLKTAEQTPLTAFYAGKLFLEAGLPPGVLNIVSGFGATAGAALASHMDVDKLAFTGSTDTGKVIL
GLAANSNLKPVTLELGGKSPFIVFEDADIDKAVELAHFALFFNQGQCCCAGSRTFVHEKVYDEFVEKSKARALKRVVGDPFRKGIEQGPQIDLKQFE
KVMKYIKSGIESNATLECGGDQIGDKGYFIQPTVFSNVKDDMLIAQDEIFGPVQSILKFSDVDEVIKRANETKYGLAAGVFTKNLDTANRVSRALKA
GTVWVNCFDVFDAAIPFGGYKMSGNGREKGIYSLNNYLQIKAVVTALNKPAWI
>AtALDH2B7
MASRRVSSLLSRSFMSSSRSIFSLRGMNRGAQRYSNLAAAVENTITPPVKVEHTQLLIGGRFVDAVSGKTFPTLDPRNGEVIAQVSEGDAEDVNRAV
AAARKAFDEGPWPKMTAYERSKILFRFADLIEKHNDEIAALETWDNGKPYEQSAQIEVPMLARVFRYYAGWADKIHGMTMPGDGPHHVQTLHEPIGV
AGQIIPWNFPLLMLSWKLGPALACGNTVVLKTAEQTPLSALLVGKLLHEAGLPDGVVNIVSGFGATAGAAIASHMDVDKVAFTGSTDVGKIILELAS
KSNLKAVTLELEESHHSFVCEDADVDQAVELAHFALFFNQGQCCCAGSRTFVHERVYDEFVEKAKARALKRNVGDPFKSGIEQGPQVDSEQFNKILK
YIKHGVEAGATLQAGGDRLGSKGYYIQPTVFSDVKDDMLIATDEIFGPVQTILKFKDLDEVIARANNSRYGLAAGVFTQNLDTAHRLMRALRVGTVW
INCFDVLDASIPFGGYKMSGIGREKGIYSLNNYLQVKAVVTSLKNPAWL
>AtALDH3I1
MTKLLEINHIQTLCFAKGFSPARLNVATSPFLISRRGGGGYCSNACIPYRLKFTCYATLSAVVKEQASDFRGKEAALLVDELRSNFNSGRTKSYEWR
ISQLQNIARMIDEKEKCITEALYQDLSKPELEAFLAEISNTKSSCMLAIKELKNWMAPETVKTSVTTFPSSAQIVSEPLGVVLVISAWNFPFLLSVE
PVIGAIAAGNAVVLKPSEIAPAASSLLAKLFSEYLDNTTIRVIEGGVPETTALLDQKWDKIFFTGGARVARIIMAAAARNLTPVVLELGGKCPALVD
SDVNLQVAARRIIAGKWACNSGQACIGVDYVITTKDFASKLIDALKTELETFFGQNALESKDLSRIVNSFHFKRLESMLKENGVANKIVHGGRITED
KLKISPTILLDVPEASSMMQEEIFGPLLPIITVQKIEDGFQVIRSKSKPLAAYLFTNNKELEKQFVQDVSAGGITINDTVLHVTVKDLPFGGVGESG
IGAYHGKFSYETFSHKKGVLYRSFSGDADLRYPPYTPKKKMVLKALLSSNMFAAILAFFGFSKDS
>AtALDH3H1
MAAKKVFGSAEASNLVTELRRSFDDGVTRGYEWRVTQLKKLMIICDNHEPEIVAALRDDLGKPELESSVYEVSLLRNSIKLALKQLKNWMAPEKAKT
SLTTFPASAEIVSEPLGVVLVISAWNYPFLLSIDPVIGAISAGNAVVLKPSELAPASSALLTKLLEQYLDPSAVRVVEGAVTETSALLEQKWDKIFY
TGSSKIGRVIMAAAAKHLTPVVLELGGKSPVVVDSDTDLKVTVRRIIVGKWGCNNGQACVSPDYILTTKEYAPKLIDAMKLELEKFYGKNPIESKDM
SRIVNSNHFDRLSKLLDEKEVSDKIVYGGEKDRENLKIAPTILLDVPLDSLIMSEEIFGPLLPILTLNNLEESFDVIRSRPKPLAAYLFTHNKKLKE
RFAATVSAGGIVVNDIAVHLALHTLPFGGVGESGMGAYHGKFSFDAFSHKKAVLYRSLFGDSAVRYPPYSRGKLRLLKALVDSNIFDLFKVLLGLA
>AtALDH3F1
MEAMKETVEESLREMRETFASGRTRSLKWRKAQIGAIYEMVKDNEDKICNALFQDLGKLSTEAFRDELGVVLRTATVAINCLDKWAVPKHSKLPLLF
YPAKGKVISEPYGTVLVLSSWNFPISLSLDPLIGAIAAGNTVLLKSSELSPNASAFLAKTIPAYLDTKAIKVIEGGPDVATILLQHQWDKIFFTGSP
KIGRIIMAAAAQHLTPVTLELGGKCPTIVDHTISKNIKSVVKRIAGGKWGSCNGQACISVDYVLIEKSFAPTLIDMLKPTIKSFFGENPKESGCLSR
IANKHHVQRLSRLLSDPRVQASIVYGGSIDEDKLYVEPTILLDPPLDSEIMNEEIFGPILPIITVRDIQESIGIINTKPKPLAIYAFTNDENLKTRI
LSETSSGSVTFNDVMIQYMCDALPFGGVGESGIGRYHGKYSFDCFSHEKAIMEGSLGMDLEARYPPWNNFKLTFIRLAFREAYFKLILLMLGLKR
>AtALDH5F1
MVIGAAARVAIGGCRKLISSHTSLLLVSSQCRQMSMDAQSVSEKLRSSGLLRTQGLIGGKWLDSYDNKTIKVNNPATGEIIADVACMGTKETNDAIA
SSYEAFTSWSRLTAGERSKVLRRWYDLLIAHKEELGQLITLEQGKPLKEAIGEVAYGASFIEYYAEEAKRVYGDIIPPNLSDRRLLVLKQPVGVVGA
ITPWNFPLAMITRKVGPALASGCTVVVKPSELTPLTALAAAELALQAGVPPGALNVVMGNAPEIGDALLTSPQVRKITFTGSTAVGKKLMAAAAPTV
KKVSLELGGNAPSIVFDDADLDVAVKGTLAAKFRNSGQTCVCANRVLVQDGIYDKFAEAFSEAVQKLEVGDGFRDGTTQGPLINDAAVQKVETFVQD
AVSKGAKIIIGGKRHSLGMTFYEPTVIRDVSDNMIMSKEEIFGPVAPLIRFKTEEDAIRIANDTIAGLAAYIFTNSVQRSWRVFEALEYGLVGVNEG
LISTEVAPFGGVKQSGLGREGSKYGMDEYLEIKYVCLGDMNRH
>AtALDH6B2
MVRVKQKNLESYRSNGTYPPTWRNPTTSFAPDQHRVSIHSSLKSKTKRRRLYKEADDNTKLRSSSSTTTTTTTMLLRISGNNLRPLRPQFLALRSSW
LSTSPEQSTQPQMPPRVPNLIGGSFVESQSSSFIDVINPATQEVVSKVPLTTNEEFKAAVSAAKQAFPLWRNTPITTRQRVMLKFQELIRKNMDKLA
MNITTEQGKTLKDSHGDIFRGLEVVEHACGMATLQMGEYLPNVSNGVDTYSIREPLGVCAGICPFNFPAMIPLWMFPVAVTCGNTFILKPSEKDPGA
SVILAELAMEAGLPDGVLNIVHGTNDTVNAICDDEDIRAVSFVGSNTAGMHIYARAAAKGKRIQSNMGAKNHGLVLPDANIDATLNALLAAGFGAAG
QRCMALSTVVFVGDAKSWEDKLVERAKALKVTCGSEPDADLGPVISKQAKERICRLIQSGVDDGAKLLLDGRDIVVPGYEKGNFIGPTILSGVTPDM
ECYKEEIFGPVLVCMQANSFDEAISIINKNKYGNGAAIFTSSGAAARKFQMDIEAGQIGINVPIPVPLPFFSFTGNKASFAGDLNFYGKAGVDFFTQ
IKTVTQQWKDIPTSVSLAMPTSQKQ
>AtALDH7B4
MGSANNEYEFLSEIGLTSHNLGSYVAGKWQANGPLVSTLNPANNQPIAQVVEASLEDYEQGLKACEEAAKIWMQVTAPKRGDIVRQIGDALRSKLDY
LGRLLSLEMGKILAEGIGEVQEVIDMCDFAVGLSRQLNGSVIPSERPNHMMLEMWNPLGIVGVITAFNFPCAVLGWNACIALVCGNCVVWKGAPTTP
LITIAMTKLVAEVLEKNNLPGAIFTAMCGGAEIGEAIAKDTRIPLVSFTGSSRVGSMVQQTVNARSGKTLLELSGNNAIIVMDDADIQLAARSVLFA

AVGTAGQRCTTCRRLLLHESVYDKVLEQLLTSYKQVKIGNPLEKGTLLGPLHTPESKKNFEKGIEVIKSQGGKILTGGKAVEGEGNFVEPTIIEISA
DAAVVKEELFAPVLYVLKFKSFGEAVAINNSVPQGLSSSIFTRNPENIFRWIGPLGSDCGIVNVNIPTNGAEIGGAFGGEKATGGGREAGSDSWKQY
MRRSTCTINYGNELPLAQGINFG
>AtALDH10A8
MAIPMPTRQLFIDGEWREPILKKRIPIVNPATEEVIGDIPAATTEDVDVAVNAARRALSRNKGKDWAKAPGAVRAKYLRAIAAKVNERKTDLAKLEA
LDCGKPLDEAVWDMDDVAGCFEFYADLAEGLDAKQKAPVSLPMESFKSYVLKQPLGVVGLITPWNYPLLMAVWKVAPSLAAGCTAILKPSELASVTC
LELADICREVGLPPGVLNVLTGFGSEAGAPLASHPGVDKIAFTGSFATGSKVMTAAAQLVKPVSMELGGKSPLIVFDDVDLDKAAEWALFGCFWTNG
QICSATSRLLVHESIASEFIEKLVKWSKNIKISDPMEEGCRLGPVVSKGQYEKILKFISTAKSEGATILHGGSRPEHLEKGFFIEPTIITDVTTSMQ
IWREEVFGPVLCVKTFASEDEAIELANDSHYGLGAAVISNDTERCDRISEAFEAGIVWINCSQPCFTQAPWGGVKRSGFGRELGEWGLDNYLSVKQV
TLYTSNDPWGWYKSPN
>AtALDH10A9
MAITVPRRQLFIGGQWTEPVLRKTLPVVNPATEDIIGYIPAATSEDVELAVEAARKAFTRNNGKDWARATGAVRAKYLRAIAAKVIERKSELANLEA
IDCGKPLDEAAWDMDDVAGCFEYYADLAEGLDAKQKTPLSLPMDTFKGYILKEPIGVVGMITPWNYPLLMAVWKVAPSLAAGCTAILKPSELASLTC
LELADICREVGLPPGVLNILTGLGTEAGAPLASHPHVDKIVFTGSTTTGSSIMTSAAKLVKPVSLELGGKSPIIVFDDVDIDKAVEWTMFGCFWTNG
QICSATSRLLVHERIADEFLDKLVKWTKNIKISDPFEEGCRLGPVVSKGQYERVLKFVSNARNEGATVLCGGVRPEHLKKGYFVEPAIVSNVTTSME
IWREEVFGPALCVKTFSTEDEAIQLANDSQYGLAGAVLSNDLERCDRVSKAFQAGIVWVNCSQPCFCQAPWGGTKRSGFGRELGEWGLENYLSVKQV
TQYISDEPWGWYKPPSKL
>AtALDH11A3
MAGTGLFAEILDGEVYKYYADGEWKTSSSGKSVAIMNPATRKTQYKVQACTQEEVNAVMELAKSAQKSWAKTPLWKRAELLHKAAAILKDNKAPMAE
SLVKEIAKPAKDSVTEVVRSGDLISYCAEEGVRILGEGKFLLSDSFPGNDRTKYCLTSKIPLGVVLAIPPFNYPVNLAVSKIAPALIAGNSLVLKPP
TQGAVSCLHMVHCFHLAGFPKGLISCITGKGSEIGDFLTMHPAVNCISFTGGDTGISISKKAGMIPLQMELGGKDACIVLDDADLDLVASNIIKGGF
SYSGQRCTAVKVVLVMESVADELVEKVKAKVAKLTVGPPEENSDITAVVSESSANFIEGLVMDAKEKGATFCQEYKREGNLIWPLLLDNVRPDMRIA
WEEPFGPVVPVLRINSVEEGINHCNASNFGLQGCVFTKDINKAILISDAMETGTVQINSAPARGPDHFPFQGLKDSGIGSQGVTNSINLMTKVKTTV
INLPTPSYSMG
>AtALDH12A1
MYRVFASRALRAKSLCDKSSTSLASLTLSRLNHSIPFATVDAEELSGSHPAEVQSFVQGKWIGSSNHNTLLDPLNGEPFIKVAEVDESGTQPFVDSL
SQCPKHGLHNPFKSPERYLLYGDISTKAAHMLALPKVADFFARLIQRVAPKSYQQAAGEVFVTRKFLENFCGDQVRFLARSFAIPGNHLGQQSHGYR
WPYGPVTIVTPFNFPLEIPLLQLMGALYMGNKPLLKVDSKVSIVMEQMMRLLHYCGLPAEDVDFINSDGKTMNKILLEANPRMTLFTGSSRVAEKLA
LDLKGRIRLEDAGFDWKVLGPDVQEVDYVAWQCDQDAYACSGQKCSAQSMLFVHENWSKTPLVSKLKELAERRKLEDLTIGPVLTFTTEAMLEHMEN
LLQIPGSKLLFGGKELKNHSIPSIYGALEPTAVYVPIEEILKDNKTYELVTKEIFGPFQIVTEYKKDQLPLVLDALERMHAHLTAAVVSNDPIFLQE
VIGNSVNGTTYAGLRGRTTGAPQNHWFGPAGDPRGAGIGTPEAIKLVWSCHREVIYDYGPVPQGWELPPST
>AtALDH18B1
MEELDRSRAFARDVKRIVVKVGTAVVTGKGGRLALGRLGALCEQLAELNSDGFEVILVSSGAVGLGRQRLRYRQLVNSSFADLQKPQTELDGKACAG
VGQSSLMAYYETMFDQLDVTAAQLLVNDSSFRDKDFRKQLNETVKSMLDLRVIPIFNENDAISTRRAPYQDSSGIFWDNDSLAALLALELKADLLIL
LSDVEGLYTGPPSDPNSKLIHTFVKEKHQDEITFGDKSRLGRGGMTAKVKAAVNAAYAGIPVIITSGYSAENIDKVLRGLRVGTLFHQDARLWAPIT
DSNARDMAVAARESSRKLQALSSEDRKKILLDIADALEANVTTIKAENELDVASAQEAGLEESMVARLVMTPGKISSLAASVRKLADMEDPIGRVLK
KTEVADGLVLEKTSSPLGVLLIVFESRPDALVQIASLAIRSGNGLLLKGGKEARRSNAILHKVITDAIPETVGGKLIGLVTSREEIPDLLKLDDVID
LVIPRGSNKLVTQIKNTTKIPVLGHADGICHVYVDKACDTDMAKRIVSDAKLDYPAACNAMETLLVHKDLEQNAVLNELIFALQSNGVTLYGGPRAS
KILNIPEARSFNHEYCAKACTVEVVEDVYGAIDHIHRHGSAHTDCIVTEDHEVAELFLRQVDSAAVFHNASTRFSDGFRFGLGAEVGVSTGRIHARG
PVGVEGLLTTRWIMRGKGQVVDGDNGIVYTHQDIPIQA
>AtALDH18B2
MTEIDRSRAFAKDVKRIVVKVGTAVVTGKGGRLALGRLGAICEQLAELNSDGFEVILVSSGAVGLGRQRLRYRQLVNSSFADLQKPQMELDGKACAG
VGQSSLMAYYETMFDQLDVTVAQMLVTDSSFRDKDFRKQLSETVKAMLRMRVIPVFNENDAISTRRAPYKDSTGIFWDNDSLAALLSLELKADLLIL
LSDVEGLYTGPPSDSTSKLIHTFIKEKHQDEITFGEKSKLGRGGMTAKVKAAVNAAYGGVPVIITSGYAAENISKVLRGLRVGTLFHQDAHLWAPVV
DTTSRDMAVAARESSRKLQALSSEDRKQILHDIANALEVNEKTIKAENDLDVAAAQEAGYEESLVARLVMKPGKISSLAASVRQLAEMEDPIGRVLK
KTQVADDLILEKTSSPIGVLLIVFESRPDALVQIASLAIRSGNGLLLKGGKEARRSNAILHKVITDAIPETVGGKLIGLVTSREEIPDLLKLDDVID
LVIPRGSNKLVSQIKNSTKIPVLGHADGICHVYVDKSGKLDMAKRIVSDAKLDYPAACNAMETLLVHKDLEQNGFLDDLIYVLQTKGVTLYGGPRAS
AKLNIPETKSFHHEYSSKACTVEIVEDVYGAIDHIHQHGSAHTDCIVTEDSEVAEIFLRQVDSAAVFHNASTRFSDGFRFGLGAEVGISTSRIHARG
PVGVEGLLTTRWIMRGKGQVVDGDNGIVYTHKDLPVLQRTEAVENGI
>AtALDH22A1
MPFWWPLIVLAFAYAICKFLLMLIPPNVPSIDVDASDVLAHGKDTEENSFIYIPPRGRSQQSDKKVQCYEPATMKYLGYFPALSPTEVEERVTLSRK
AQKTWAQSSFKLRRQFLRILLKYIIEHQELICEVSSRDTGKTMVDASLGEIMTTCEKITWLLSEGERWLKPESRSSGRAMLHKVSRVEFHPLGVIGA
IVPWNYPFHNIFNPMLAAVFSGNGIVIKVSEHASWSGCFYFRIIQAALAAVGAPENLVDVITGFAETGEALVSSVDKMIFVGSTAVGKMIMRNAAET
LTPVTLELGGKDAFIICEDADVSHVAQVAVRGTLQSSGQNCAGAERFYVHKDIYTAFIGQVTKIVKSVSAGPPLTGRYDMGAICLQEHSEHLQSLVN
DALDKGAEIAVRGSFGHLGEDAVDQYFPPTVLINVNHNMKIMKEEAFGPIMPIMQFSTDEEVIKLANDSRYALGCAVFSGSKHRAKQIASQIQCGVA
AINDFASNYMCQSLPFGGVKDSGFGRFAGIEGLRACCLVKSVVEDRFWPLIKTKIPKPIQYPVAENAFEFQEALVETLYGLNIWDRLRSLIDVLKFL
TDQSSNVSRTRKSH
>OsALDH2-1
MAAANGGDSKGFEVPKLEIKFTKLFINGRFVDAVSGKTFETRDPRTGEVIAKIAEGDKADIDLAVKAAREAFDHGPWPRMSGFARGRILHKFADLVE
QHVEELAALDTVDAGKLFAMGKLVDIPGGANLLRYYAGAADKVHGETLKMARPCHGYTLKEPVGVVGHIVPWNYPTTMFFFKASPALAAGCTMVVKP
AEQTPLSALFYAHLAKLAGVPDGVLNVVPGFGPTAGAAISSHMDIDKVSFTGSTEVGRLVMEAAAKSNLKPVSLELGGKSPVIVFDDADLDTAVNLV
HMASYTNKGEICVAGSRIYVQEGIYDAFVKKATEMAKKSVVGDPFNPRVHQGPQIDKEQYEKILKYIDIGKREGATLVTGGKPCGENGYYIEPTIFT
DVKEEMSIAQEEIFGPVMALMKFKTVEEAIQKANSTRYGLAAGIVTKNIDVANTVSRSIRAGAIWINCYLGFDPDVPFGGYKMSGFGKDMGMDALEK
YLHTKAVVTPLYNTPWL
>OsALDH2-2

MGSTGDCGNGKAAAGGGGLVVPEIKFTKLFINGEFVDAASGKTFKTRDPRTGDVLAHIAEADKADVDLAVKAAREAFEHGKWPRMSGYERSRVMNKL
ADLVEQHADELAALDGADAGKLLTLGKIIDMPAAAQMMRYYAGAADKIHGESLRVAGKYQGYTLREPIGVVGVIIPWNFPTMMFFLKVSPALAAGCT
IVVKPAEQTPLSALYYAHLAKLAGVPDGVINVVPGFGPTAGAALSSHMDVDSVAFTGSAEIGRAIMESAARSNLKNVSLELGGKSPMIVFDDADVDM
AVSLSSLAVFFNKGEICVAGSRVYVQEGIYDEFVKKAVEAAKNWKVGDPFDAATNMGPQVDKVQFERVLKYIEIGKNEGATLLTGGKPTGDKGYYIE
PTIFVDVKEEMTIAQEEIFGPVMSLMKFKTVEEAIEKANCTKYGLAAGIVTKNLNIANMVSRSVRAGTVWVNCYFAFDPDAPFGGYKMSGFGRDQGM
VAMDKYLQVKTVITAVPDSPWY
>OsALDH2-3
MSGSERGRVMAKYAEVVERHADELAALESLDAGKPLAAARAVDVGECVGILRYFAGAADKIHGETLKMSRQLQGYTLREPLGVAGLIVPWNFPAIMF
FSKVSPALAAGCTVVVKPAEQTPLSALFLAHLSKQAGVPDGVINVVTGFGPTAGAAISSHMDVDVVAFTGSTEVGRLIMEASAKSNLKPVALELGGK
SPFIVFDDADLDKAVELAIGGNFFNKGEACVAGSRVFVQEGIYDRFEQKLADTMKSWVVGDPFDPRVNQGPQVDKAQYERVLGYIEQGKAEGATVLT
GGKPCGKKGYYIEPTIFTNVKDDMVIAREEIFGPVMCLMKFKTVEEAIERANGTRYGLAAGLVTRDIDVANRMARSIRAGVVWVNCYFAMDRSCPFG
GRKMSGFGKDDSMHALDKFLAVKSVVTPVHGSPWF
>OsALDH2-4
MAARRAASSLLSRGLIARPSAASSTGDSAILGAGSARGFLPGSLHRFSAAPAAAATAAATEEPIQPPVDVKYTKLLINGNFVDAASGKTFATVDPRT
GDVIARVAEGDAEDVNRAVAAARRAFDEGPWPRMTAYERCRVLLRFADLIEQHADEIAALETWDGGKTLEQTTGTEVPMVARYMRYYGGWADKIHGL
VVPADGPHHVQVLHEPIGVAGQIIPWNFPLLMFAWKVGPALACGNAVVLKTAEQTPLSALFVASLLHEAGLPDGVLNVVSGFGPTAGAALSSHMGVD
KLAFTGSTGTGKIVLELAARSNLKPVTLELGGKSPFIVMDDADVDQAVELAHRALFFNQGQCCCAGSRTFVHERVYDEFVEKARARALQRVVGDPFR
TGVEQGPQIDGEQFKKILQYVKSGVDSGATLVAGGDRAGSRGFYIQPTVFADVEDEMKIAQEEIFGPVQSILKFRFATLEFPRIPSDRDRLSTGIYT
NTLRAMCSTVEEVVRRANATPYGLAAGVFTQRLDAANTLARALRVGTVWVNTYDVFDAAVPFGGYKMSGVGREKGVYSLRNYLQTKAVVTPIKDAAW
L
>OsALDH2-5
MAAAAARRGSSLLSRCLLSRPAAAASPAVPSALRRADGTQGLLPGILQRFSTAAVAEEPISPPVQVNYTQLLIDGKFVDSASGKTFPTLDPRTGELI
AHVAEGDAEDINRAVHAARKAFDEGPWPKMTAYERSRILLRFADLIEKHNDEIAALETWDNGKPYAQAANIEVPMVARLMRYYAGWADKIHGLVVPA
DGPHHVQVLHEPIGVAGQIIPWNFPLLMFAWKVGPALACGNTVVLKTAEQTPLSALFASKLLHEAGLPDGVVNVVSGFGPTAGAALASHMDVDKIAF
TGSTDTGKVVLELAARSNLKSVTLELGGKSPFIIMDDADVDHAVELAHFALFFNQGQCCCAGSRTFVHERIYDEFVEKAKARALKRVVGDPFKNGVE
QGPQIDDEQFNKILRYIKYGVDSGANLVTGGDRLGDKGYYIQPTIFSDVQDNMRIAQEEIFGPVQSILKFNDLNEVIKRANASQYGLAAGVFTNNLN
TANTLTRALRVGTVWVNCFDVFDAAIPFGGYKQSGIGREKGIDSLKNYLQVKAVVTPIKNAAWL
>OsALDH3-1
MEEKPQHGSLGGLVAGVREEYESGRTKELEWRKAQLGGLIRMITEEEDAIFDALHDDLGKHRVESFRDEVGVLAKSVRNTLQNLKKWASPEKVDVPL
ISFPCNARVVPEPIGVVLIFSCWNLPIGLALEPLSGAIAAGNAVVLKPSEFAPSTAAFLAANIPKYLDANAVKVVQGGAEVGEELMEHRWDKVLFTG
NARVGRIIMTKAAKHLTPVALELGSKCPCIVDCLDSKRECQVAVNRIIGAKWSTCAGQACVAIDYILVEEQFAPFLIELLKSTLKRFFTEPEYMARI
LNEKHFHRLTNLLEDDQVKSSIVHGGNADPKTLWIEPTIVLNPPFDSDIMMEEIFGPLLPIITVKKTEDCIAFLKSKPKPLAIYAFTNNEKLKQRIV
AETSSGSVLFNDAIVQYGLDSVPFGGIGESGFGQYHGKYTFELFSHRKAVVRRSLLVEFMFRYPPWDEYKMGMLRRVFRFDYVSLVLALLAFWLLGI
RR
>OsALDH3-2
MAPAMVAAMGEKPKPAVVLGGMVSGLREVYESGRTKDLEWRQSQLKALIRLLTDKEEEIFAVLHDDLGKHRGESFRDELGILVKSIKYTLQNLKKWA
ASERAESPLVAFPATAMVVPEPLGVVLVFSCWNLPLGLALEPLSGAIAAGNAVVLKPSELAPSTAAFLAANIPRYLDSRAVKVVLGGPNVGEELMEH
RWDKVLFTGSARIGRIIMAKAVKHLTPVALELGSKCPCIVDWLDSKRDRQIAVNRIIGAKWSTCAGQACIAIDHVIVEERFAPILIELLKSTLKRFM
AKPGGMARILNAKHFERLSGYLEDNRVAASVVHGGYMDPKKLNIEPTLLLNPPADSDVMTEEVFGPILPIITVKKIEDCIAYLKSKPKPIAMYAFTN
NERLKRRIVEETSSGSVTFNDAVVQYALESVPFGGVGHSGFGQYHGKYSFELFSHKKAVFKRSFLIEFMFRYPPWDERKIGTLRHVFSYNYFLLFFN
LLGFRR
>OsALDH3-3
MGRVAPSVEEVGGEQPPPALGPGETVSGTVAELRAAYESGRTRSLEWRQSQLRGLLRLLAEEEAAAFRALREDLGKHQAEAYRDEIGVLVKSANAAL
REVGKWMAPEKVWVPLIAFPARAQLEPQPLGVILVFSCWNVPLGLSLEPLVGALAAGNAVALKPSELAPATAKFLGDNVGKYMDATAVKVIQGGPEV
GEQLMEHRWDKVLFTGSPRIARVVMAAAAKHLTPVALELGGKCPCIFDTIGGSARDLQTAVNRVVGGKWSSCAGQACLAIDYVLVEERFVPVLIKAL
KSTLKKFFADSDHMARIVNARHFQRLSDLLKDKSVAASVLHGGTLDAKNLCIEPTILLNPPLDSAIMTEEIFGPLLPIITVKKIEDSIAFVRARPRP
LAVYAFTKNAALRRRIVEETSSGSVTFNDAVVQYGIDSLPFGGVGESGFGQYHGKYSFEMFSHKKAVLTRGYLIELTARYPPWDDSKISMMRQLYRY
NYVGFVLTFLGLKK
>OsALDH3-4
MAEEEVAAVVGELRGSFRSGRTRAAEWRAAQLRGIVRMVEEREGDISDALHSDLAKPRMESYLHEISLAKAACTFALKGLKNWMKPEKVPAALTTFP
STAQIVSEPLGVVLVISAWNYPFLLSIDPVIGAIAAGNAVVLKPSEIAPATSALFAKLLPEYVDSSCIKVVEGGVPETTALLEQKWDKIFYTGSGNV
GRIVMAAAAKHLTPVALELGGKCPAIVDSNTDLHVTMKRLAVGKWGCNNGQACIAPDYVITTKSFAPELVDSLKRVLKRFYGEDPLQSEDLSRIVNS
NHFRRLTNLIEDKKVAQKIVYGGQTDEKQLKIAPTVLLDVPLDTTLMAEEIFGPLLPIVTVDKIEDSIQFINSRTKPLAAYLFTKDKKLQEEFVSNV
PAGGMLVNDVALHLANPHLPFGGVGDSGIGSYHGKFSFDCFTHKKAVLIRGFGGEATARYPPYTIEKQKILRGLINGSFFALILALLGFPKERR
>OsALDH3-5
MAAARSVGMEAEVAALRGRFAAGGTRGAEWRAAQLRGILRMAAEAEAEVCRALHADLAKPYTESYVHEIALVKSSCKFALKNLKKWMKPQKVTAPLM
TFPSTARVAAEPLGVVLVISAWNYPFLLSIDPIIGAIAAGNAVVLKPSEVAPATSSLLAELLPRYVDGSCIKVVEGGVAETTTLLEQKWDKIFYTGN
GKVGRIVMASAAKHLTPVVLELGGKCPVVVDSNVNLHVTAKRIAAGKWGCNNGQACISPDFIITTKSFAPKLLEALEKVLEKFYGRDPLRSSDLSRI
VNSNHFNRLKKLMDDENVSDKIVFGGQRDEHQLKIAPTIFMDVPLDSGIMKEEIFGPLLPIITVDKIHESFALINSMTKALAAYLFTKDSKLQEQYE
AAISAGGMLVNDTAVHLTNQYLPFGGVGESGMGAYHGRFSFEAFSHKKAVLVRRFAGEAAARYPPYSPAKLKILRGVLKGNLGAMIKAILGFPRGK
>OsALDH5
MAMAMAMRRAAALGARHILAASSTSSSGVLLRRHMSVDAGAAMEKVRAAGLLRTQGLIGGKWVDAYDGKTIEVQNPATGETLANVSCMGSKETSDAI
ASAHSTFYSWSKLTANERSKALRKWHDLIISHKEELALLMTLEQGKPMKEALVEVTYGASFIEYFAEEAKRIYGDIIPPTLSDRRLLVLKQPVGVVG
AVTPWNFPLAMITRKVGPALACGCTVVVKPSEFTPLTALAAADLALQAGIPAGAINVVMGNAPEIGDALLQSTQVRKITFTGSTAVGKKLMAGSANT
VKKVSLELGGNAPCIVFDDADIDVAIKGSLAAKFRNSGQTCVCANRILVQEGIYEKFASAFIKAVQSLKVGNGLEESTSQGPLINEAAVQKVEKFIN

DATSKGANIMLGGKRHSLGMSFYEPTVVGNVSNDMLLFREEVFGPVAPLVPFKTEEDAIRMANDTNAGLAAYIFTKSIPRSWRVSEALEYGLVGVNE
GIISTEVAPFGGVKQSGLGREGSKYGMDEYLELKYICMGNLN
>OsALDH6
MLRAALLRSGSGLRRPPMAAPLSTAAAASWLSDSASSPPRVRLLIGGEFVESRADEHVDVTNPATQEVVSRIPLTTADEFRAAVDAARTAFPGWRNT
PVTTRQRIMLKYQELIRANMDKLAENITTEQGKTLKDAWGDVFRGLEVVEHACGMGTLQMGEYVSNVSNGIDTFSIREPLGVCAGICPFNFPAMIPL
WMFPIAVTCGNTFVLKPSEKDPGAAMMLAELAMEAGLPKGVLNIVHGTHDVVNNICDDEDIKAVSFVGSNIAGMHIYSRASAKGKRVQSNMGAKNHA
IILPDADRDATLNALIAAGFGAAGQRCMALSTAVFVGGSEPWEDELVKRASSLVVNSGMASDADLGPVISKQAKERICKLIQSGADNGARVLLDGRD
IVVPNFENGNFVGPTLLADVKSEMECYKEEIFGPVLLLMKAESLDDAIQIVNRNKYGNGASIFTTSGVSARKFQTDIEAGQVGINVPIPVPLPFFSF
TGSKASFAGDLNFYGKAGVQFFTQIKTVTQQWKESPAQRVSLSMPTSQK
>OsALDH7
MGSFARKEHQFLAELGLAPRNPGSFACGAWGGSGPVVTSTNPTNNQVIAEVVEASAREYEEGMRACYDAAKTWMAIPAPKRGEIVRQIGDALRAKLH
HLGRLVSLEMGKILPEGIGEVQEIIDMCDYAVGLSRQLNGSIIPSERPNHMMMEVWNPLGVVGVITAFNFPCAVLGWNACIALVCGNCVVWKGAPTT
PLITIAMTKIVASVLERNNLPGSIFTAFCGGADIGQAISLDTRIPLVSFTGSTKVGLMVQQQVNARFGKCLLELSGNNAIIVMDDADIQLAVRSVLF
AAVGTAGQRCTTCRRLLLHESIYRTFLDQLVEVYKQVRIGDPLENGTLLGPLHTPASRDAFLKGIQTIRSQGGKILYGGSAIESEGNFVQPTIVEIS
PSAPVVREELFGPVLYVMKVQNLKEAVEINNSVPQGLSSSIFTKRPDIIFKWIGPHGSDCGIVNVNIPTNGAEIGGAFGGEKATGGGREAGSDSWKQ
YMRRATCTINYGSELPLAQGINFG
>OsALDH10-1
MAAPSAIPRRGLFIGGGWREPSLGRRLPVVNPATEATIGDIPAATAEDVELAVSAARDAFGRDGGRHWSRAPGAVRAKYLKAIAAKIKDKKSYLALL
ETLDSGKPLDEAAGDMEDVAACFEYYADLAEALDGKQRAPISLPMENFESYVLKEPIGVVGLITPWNYPLLMATWKVAPALAAGCTAVLKPSELASL
TCLELGGICAEIGLPPGVLNIITGLGTEAGAPLASHPHVDKIAFTGSTETGKRIMITASQMVKPVSLELGGKSPLIVFDDVDIDKAVEWAMFGCFAN
AGQVCSATSRLLLHEKIAKRFLDRLVAWAKSIKISDPLEEGCRLGSVVSEGQYQKIMKFISTARCEGATILYGGARPQHLKRGFFIEPTIITNVSTS
MQIWREEVFGPVICVKEFRTEREAVELANDTHYGLAGAVISNDLERCERISKAIQSGIVWINCSQPCFVQAPWGGNKRSGFGRELGQWGLDNYLSVK
QVTKYCSDEPYGWYRPPSKL
>OsALDH10-2
MATAIPQRQLFVAGEWRAPALGRRLPVVNPATESPIGEIPAGTAEDVDAAVAAAREALKRNRGRDWARAPGAVRAKYLRAIAAKIIERKSELARLET
LDCGKPLDEAAWDMDDVAGCFEYFADLAESLDKRQNAPVSLPMENFKCYLRKEPIGVVGLITPWNYPLLMATWKVAPALAAGCTAVLKPSELASVTC
LELADVCKEVGLPSGVLNIVTGLGSEAGAPLSSHPGVDKVAFTGSYETGKKIMASAAPMVKPVSLELGGKSPIVVFDDVDVEKAVEWTLFGCFWTNG
QICSATSRLILHKKIAKEFQERMVAWAKNIKVSDPLEEGCRLGPVVSEGQYEKIKQFVSTAKSQGATILTGGVRPKHLEKGFYIEPTIITDVDTSMQ
IWREEVFGPVLCVKEFSTEEEAIELANDTHYGLAGAVLSGDRERCQRLTEEIDAGIIWVNCSQPCFCQAPWGGNKRSGFGRELGEGGIDNYLSVKQV
TEYASDEPWGWYKSPSKL
>OsALDH11
MAAVAGTGVFAEILEGEVYRYYADGEWRVSASGKSVAIVNPTTRLTQYRVQACTQEEVNKVMETAKVAQKAWARTPLWKRAELLHKAAAILKEHKTP
IAECLVKEIAKPAKDAISEVVRSGDLVSYTAEEGVRILGEGKLLVSDSFPGNERNKYCLSSKVPLGVVLAIPPFNYPVNLAVSKIGPALIAGNALVL
KPPTQGAVAALHMVHCFHLAGFPKGLINCVTGKGSEIGDFLTMHPGVNCISFTGGDTGIAISKKAGMVPLQMELGGKDACVVLEDADLDLVAANIVK
GGFSYSGQRCTAVKVVLIMESVADIVVEKVKAKLAKLTVGPPEADSDITPVVTESSANFIEGLVMDAKEKGATFCQEYRREGNLIWPLLLDHVRPDM
RIAWEEPFGPVLPVIRINSVEEGIHHCNASNFGLQGCVFTKDINKAIMISDAMETGTVQINSAPARGPDHFPFQGLKDSGIGSQGITNSINMMTKVK
STVINLPSPSYTMG
>OsALDH12
MSLILSRRRLAAAVRRSGPAALASRWMHTPPFATVSPQEISGSSPAEVQNFVQGSWTTSGNWNWLVDPLNGEKFIKVAEVQEAEIKPFVESLSNCPK
HGLHNPLKAPERYLMYGDISAKAANMLGQPVVSDFFAKLIQRVSPKSYQQALAEVQVSQKFLENFCGDQVRFLARSFAVPGNHLGQSSNGYRWPYGP
VAIITPFNFPLEIPLLQLMGALYMGNKPVLKVDSKVSIVMDQMLRLLHACGMPAEDVDFINSDGITMNKLLLEANPKMTLFTGSSRIAEKLAADLKG
KIKLEDAGFDWKILGPDVQEVDYIAWVCDQDAYACSGQKCSAQSILFMHKNWSSSGLLDKMKSLSERRKLEDLTIGPVLTVTTSSMIEHMKNLLKIP
GSKVLFGGEPLENHSIPEIYGAFKPTAVFVPLSEILKSGNFELVTREIFGPFQVVTEYSDDELELVLEACERMNAHLTAAVVSNDPLFLQEVLGRSV
NGTTYAGIRARTTGAPQNHWFGPAGDPRGAGIGTPEAIKLVWSCHREIIYDIGPLPKNRALPSAT
>OsALDH18-1
MGRGGIGGAGLVAAVAKADVENTDSTRGFVKDVKRIIIKVGTAVVTGPNGRLAMGRLGALCEQVKQLNFEGYEVILVTSGAVGVGRQRLKYRKLVNS
SFADLQNPQMDMDGKACAAVGQSVLMAIYDTLFSQLDVTSSQLLVTDRDFMDPSFGNQLRETVNSLLDLKVIPVFNENDAISTRRQPYEDSSGIFWD
NDSLARLLAQELKADLLIMLSDVEGLYSGPPSDPQSKIIHTYVHEQHGKLISFGEKSRVGRGGMQAKVAAAFTASSKGIPVVIASGFAIDSIIKVMR
GEKIGTLFHREANQWGCSKEATAREMAVAARDCSRHLQKLSSEERKKILLDIADALEANEDLITSENQADLDLAQDIGYDKSLVARMTIKPGKIKSL
AGSIREIADMEDPISHTLKRTEVAKDLVFEKTYCPLGVLLIIFESRPDALVQIASLAIRSGNGLLLKGGKEAMRSNTILHKVITGAIPDVVGKKLIG
LVKNKDEIADLLKLDDVIDLVIPRGSNKLVSQIKAATKIPVLGHADGICHVYIDKSADMDMAKRIVLDAKVDYPAACNAMETLLVHKDLNRTEGLDD
LLVELEKEGVVIYGGPVAHDTLKLPKVDSFHHEYNSMACTLEFVDDVQSAIDHINRYGSAHTDCIITTDGKAAETFLQQVDSAAVFHNASTRFCDGA
RFGLGAEVGISTGRIHARGPVGVDGLLTTRCILRGSGQVVNGDKGVVYTHRELPLQ
>OsALDH18-2
MASVDPSRSFVRDVKRVIIKVGTAVVSRQDGRLALGRVGALCEQVKELNSLGYEVILVTSGAVGVGRQRLRYRKLVNSSFADLQKPQMELDGKACAA
VGQSGLMALYDMLFNQLDVSSSQLLVTDSDFENPKFREQLTETVESLLDLKVIPIFNENDAISTRKAPYEDSSGIFWDNDSLAGLLALELKADLLIL
LSDVDGLYSGPPSEPSSKIIHTYIKEKHQQEITFGDKSRVGRGGMTAKVKAAVLASNSGTPVVITSGFENRSILKVLHGEKIGTLFHKNANLWESSK
DVSTREMAVAARDCSRHLQNLSSEERKKILLDVADALEANEDLIRSENEADVAAAQVAGYEKPLVARLTIKPGKIASLAKSIRTLANMEDPINQILK
KTEVADDLVLEKTSCPLGVLLIVFESRPDALVQIASLAIRSGNGLLLKGGKEAIRSNTILHKVITDAIPRNVGEKLIGLVTTRDEIADLLKLDDVID
LVIPRGSNKLVSQIKASTKIPVLGHADGICHVYIDKSADMDMAKHIVMDAKIDYPAACNAMETLLVHKDLMKSPGLDDILVALKTEGVNIYGGPIAH
KALGFPKAVSFHHEYSSMACTVEFVDDVQSAIDHIHRYGSAHTDCIVTTDDKVAETFLRRVDSAAVFHNASTRFSDGARFGLGAEVGISTGRIHARG
PVGVEGLLTTRWILRGRGQVVNGDKDVVYTHKSLPLQ
>OsALDH22
MALWWPLLVLAAAYALCRILLFLIPPTVPSIDVDASDVLEDANQNKEDSYIYIPPRKGKGAQTDKVQCYEPATMKYLGYFPALTPDEVKEHVAQARK
AQKIWAKSSFKQRRQFLRILLKYILEHQDLICEISSRDTGKTMVDASLGEIMTTCEKITWLLDEGEKWLKPEYRSCGRSMLHKKAKVEFYPLGVIGA
IVSWNYPFHNVFNPMLAAIFSGNAAVIKVSEHASWSGCFYFRIIQAALAAVGAPDNLVHIITGFAETGQALVSSVDKIIFVGSPGVGRMIMNRASDT

LIPVTLELGGKDAFIVCEDVDLPSVVQVAVRAALQSSGQNCAGAERFYVHKDIYSTFVSQVVKIIKSISVGPPLSGRYDMGAICMIEHSEKLQNLVN
DAVDKGAEIAGRGSFGHLGEDAVDQFFPPTVLVNVNHTMKIMQEEAFGPILPIMKFNSDEEVVKLANDSKYGLGCAVFSGNQKRAIKIASQLHCGVA
AINDFASSYMCQSLPFGGVKDSGFGRFAGVEGLRACCLVKAVVEDRWWPYVKTMIPKPIQYPVSENGFEFQELLVETLYGLSVWDRLRSLVNLLKMI
SEQNNSPANTRKKSR
>GmALDH2B1
MASSMRISRLLSRSFLSASTTPLFSRGGSGALGAGLSKFSTAAAIEEPIKPPVKVEHTQLLIDGKFVDAATGKTFPTLDPRTGDVISHVAEGDHEDV
DRAVAAARKAFDHGPWPKMTAYERQRILLRAADLFEKHNDELAALETWDNGKPYEQSAQIEIPMLVRLFRYYAGWADKIHGLTVPADGPYHVQTLHE
PIGVAGQIIPWNFPLVMFAWKVGPALACGNTIVLKTAEQTPLSALYASKLLHEAGLPPGVLNVISGFGPTAGAAIASHMDIDKLAFTGSTETGKVVL
ELAARSNLKPVTLELGGKSPFIVCEDADVDEAVELAHFALFFNQGQCCCAGSRTFVHERVYDEFIEKAKARALKRAVGDPFKGGIEQGPQIDSEQFQ
KILKYIRSGVESGATLETGGDRFGNSGFYIQPTVFSNVKDDMLIAKEEIFGPVQTILKFKDLDDVIQRANNTHYGLAAGVFTKNINTANTLTRALRV
GTVWINCFDTFDAAIPFGGYKMSGQGREKGEYSLKNYLQVKAVVTSLKNPAWL
>GmALDH2B2
MASSLRISRLLSRSFLSASTTTPLFSRGGSGALGAGLSKFSTAAAIEEPIKPPLKVEHTQLLIDGKFVDAATGKTFPTLDPRTGDVISHVAEGDHED
VDRAVAAARKAFDRGPWPKMTAYERQRILLRAADLFEKHNDDLAALETWDNGKPYEQSAQIEIPMLVRLFRYYAGWADKIHGLTVPADGPYHVQTLH
EPIGVAGQIIPWNFPLVMFAWKVGPALACGNTIVLKTAEQTPLSALYASKLLHEAGLPPGVLNIISGFGPTAGAAIASHMDIDKLAFTGSTETGKIV
LELAARSNLKPVTLELGGKSPFIVCEDADVDEAVELAHFALFFNQGQCCCAGSRTFVHERVYDEFIEKAKARALKRAVGDPFKGGIEQGPQIDSEQF
QKILKYIRSGVESGATLETGGDRFGNSGFYIQPTVFSNVKDDMLIAKEEIFGPVQSILKFKDLDDVIQRANNTHYGLAAGVFTKNINTANTLTRALR
AGTVWVNCFDTFDAAIPFGGYKMSGQGREKGEYSLKNYLQVKAVVTSLKNPAWL
>GmALDH2B3
MATRRLSLLLSRSLSSTSFQAASLLHSLGRNSGKWGNFNRFSTAAAVEDLITPQVPITYTKHLINGQFVDAASGKTFPTYDPRTGEVIAQVAEGDAE
DINRAVSAARKAFDEGPWPKLTAYERCKIILRFADLVEKHGDELAALETWNNGKPYEQSATAELPTFVRLFRYYAGWADKIHGLTVPADGNYHVETL
HEPIGVAGQIIPWNFPLLMFAWKVGPALACGNTVILKTAEQTPLTALYVAKLFHEAGLPPGVLNVVSGYGPTAGAALASHMDVDKLAFTGSTETGKV
VLGLAAQSNLKPVTLELGGKSPFIVCEDADVDQAVELAHFALFFNQGQCCCAGSRTFVHEHIYDEFLEKAKARALKRVVGDPFKKGVEQGPQIDVEQ
FQKVLRYIKSGIESKATLECGGDQIGSKGFFVQPTVFSNVQDDMLIAKDEIFGPVQTILKFKDIDEVIRRSNATHYGLAAGVFTKNVHTANTLMRAL
RVGTVWINCFDVFDAAIPFGGYKMSGIGREKGIYSLNNYLQVKAVVSPVKKPAWL
>GmALDH2B4
MASSLRISRLISRSFSSTSFFSRGGNGFLGSRQSKFSTSAAIEEEPIKPSIQVEHTQLLIDGKFVDAASGKTFQTLDPRTGEVIAHVAEGHSEDVDR
AVSAARKAFDHGPWPKMTAYERQRILLRVADLIEKHNDELAALETWDNGKPYEQAAKIEVPMLVRLIRYYAGWADKIHGLTVPADGPYHVQTLHEPI
GVAGQIIPWNFPLLMFAWKVGPALACGNTIVLKTAEQTPLSALYAAKLFHEAGLPAGVLNVVSGFGPTAGAALASHMEVDKLAFTGSTDTGKVVLEL
AAKSNLKPVTLELGGKSPFIVCEDADVDQAVELAHFALFFNQGQCCCAGSRTFVHENVYEEFVQKAKARALRRVVGDPFKGGIEQGPQIDSDQFEKI
LRYIRSGVESGATLETGGDKLGNKGFYIQPTVFSNVKDGMLIAKDEIFGPVQSILKFKDLGEVVQRANNTRYGLAAGVFTKNMDTANTLTRALRVGT
VWINCFDTFDAAIPFGGYKMSGQGREKGEYSLKNYLQVKAVVNPLKNPAWL
>GmALDH2B5
MQVMASRILSTLHYVCSSSASATKRCLGLYSHWQRSISGIAASVVADVEPSIAPVQIDQSQLLIDGKFVDAASGKTFPTFDPRTGDVIANVAEGDAE
DVNRAVHAARKAFDEGPWPKMTAYERSRIILRFADLLEKHNDEVAAIETWDSGKTYEQAANVEIPMVVRLFRYYAGWADKIHGLTVPADGPYHVQTL
HEPIGVAGQIVPWNFPLLIFSWKVAPALACGNTVVMKTAEQTPLSALYVSKLFLEAGLPPGVLNVISGFGPTAGAALCSHMDVDKLAFTGSTSTGKR
VLELSAHSNLKPVTLELGGKSPFIVCKDADVDAAVEASHFALFFNQGQCCCAGSRTFVHESIYGEFVEKAKARALKRVVGDPFKNGVEQGPQIDSVQ
FEKIMKYIRSGVESGAQLESGGQRIGSKGYYIQPTVFSNVQDNMLIAKDEIFGPVQSILKFKDLEEVIRRANATSYGLAAGVFTKNMDTANTLMRAL
QAGTVWINCYDVFDAAIPFGGYKMSGQGRVRGIYSLRSYLQVKAVVTALKNPAWL
>GmALDH2B6
MLLKGMLKISTRAVSAARKAFDEGPWPKMTAYERSRILLRFADLVEKHSDELAALETWNNGKTYEQAAKTELPMFVRLFHYYAGWADKIHGLTVPAD
GDYHVQTLHEPIGVAGQIIPWNFPLVMFAWKVGPALACGNTIVLKTAEQTPLTALFVAKLFHEAGLPDGVLNVVSGYGPTAGAALASHMDVDKLAFT
GSTDTGKVVLELAARSNLKPVTLELGGKSPFIICEDADVDKAVELAHFALFFNQGQCCCAGSRTFVHERVYDEFLEKSKKRALRRVVGDPFKKGVEQ
GPQIDVEQFEKVLRYIRSGIESHATLECGGDRLGSKGFFVQPTVFSNVQDDMLIAQDEIFGPVQSILKFKDIDEVIRRANKTRYGLAAGVFTKNVST
ANTLMRALRAGTVWINCFDVFDAAIPFGGYKMSGIGREKGIYSLHNYLQVKAVVSPVKNPAWL
>GmALDH2B7
MASSLRISRLISRSFSSTSFFSRGGNGFLGSRHCKYSTSSAIEEEPVKPSVQVEHTQLLIDGKFVDAASGKTFPTLDPRTGEVIAHVAEGHSEDVDR
AVAAARKAFDHGPWPKMTAYERQRILLRAADLLEKHNDELAALETWDNGKPYEQAAKIEVPMLVRLIRYYAGWADKIHGLTVPADGPYHVQTLHEPI
GVAGQIIPWNFPLLMFAWKVGPALACGNTIVLKTAEQTPLSALYAAKLFHEAGLPAGVLNVVSGFGPTAGAALASHMEVDKLAFTGSTDTGKVVLEL
AAKSNLKPVTLELGGKSPFIVCEDADVDQAVELAHFALFFNQGQCCCAGSRTFVHESVYDEFVEKAKARALKRVVGDPFKGGIEQGPQIDSDQFEKI
LRYIRSGVESGATLETGGDKLGNKGFYIQPTVFSNVKDGMLIARDEIFGPVQSILKFKDLGEVVQRANNTRYGLAAGVFTTNMDTAYTLTRALRVGT
VWINCFDTFDAAIPFGGYKMSGQGREKGEYSLKNYLQVKAVVNPLKNPAWL
>GmALDH2B8
MTSIRQCESDESSLKSAFEEVSTFSLCSHWHRSISGIGASAAADVEPSIAPVQIDHSQLLIDGQFVDAASGKTFPTFDPRTGDVIANVAEGDTEDVN
RAVRAARKAFDEGPWPKMTAYERSRIILRFADLLEKHNDEVAAIETWDSGKTYEQAAKVEIPMVVRLFRYYAGWVDKIHGLTVPADGPYHVQTLHEP
IGVAGQIVPWNFPLLIFSWMAAPALACGNTVVIKTSEQAPLSALYVSKPFLEAGLPPGVLNVITGFGATAGASLCSHMDVDKLAFTGSTSTGKRQSE
VTLELGGKSPFIVCEDADVDAAVEAAHFALFFNQGQCCCAGSRTFVHESIYDEFVEKAKARALKRVVGDPFKNGVEQGPQIDSAQFEKIMKYIRSGV
ENGATLESGGQRIGSKGYYIQPTVFSNVQDNMLIAKDEIFGPVQSILKFKDLEEVIRRANATSYGLASGVFTQNMDTANTLMRALRVGTVWINCYDV
FDAAIPFGGYKMSGQGRVRGIYSLRSYLQVKAVVTALKNPAWL
>GmALDH2B9
MTFNNGDAAAASLNKVPTVNFTKLFIDGHFVHSVSGKTFETIDPRTGDVIARISEGDKEDIDIAVKAARHAFDNGPWPRLPGSERGRILLKWAELIE
ENAEELAALDAIDAGKLYHMCRNLEVPAAANTLRYYAGAADKIHGEVLKMSRDFHAYTLLEPLGVVGHITPWNFPNTMFYIKVAPSLAAGCTMVLKP
AEQTPLSALFNAHLAKLAGIPDGVINVVPGFGPTAGAALSSHMDVDKVSFTGSTQTGREIMQAAAKSNLKQVSLELGGKSPLIIFDDADIDKAAELA
LLGILYNKGEVCVASSRVLVQEGIYDEFEKKLVEKAKAWVVGDPFDPKVQQGPQVDKEQFEKVLSYIEHGKKEGATLLTGGKTVGNKGYFIEPTIFS
NIREDMLIAQDEIFGPVMALKKFKTIEEAIKSANNTKYGLAAGIVTKNLDTANTVSRSIRAGTIWINCYFAFGDDVPFGGYKMSGFGKDHGLEALHK
YLQVKSVVTPLYNSPWL

>GmALDH2C1
MFLSLHIVLINHIATFHLPTPSLRQPPFSLSLARMSALSNSSSSHGNSFLKMPAIKFTKLFINGDFVDSISGRTFETIDPRKEEVIARVSEGDKEDI
DIAVKAARQAFDSGPWPRLPGSERAKIMMKWADLVDENIEELAALDTIDAGKLYYINKVAEIPSATNALRYYAGAADKIHGDVLKMNGDFHAYTLLE
PIGVVGHIIPWNAPSLSFFIKVSPSLAAGCTMVLKPAEQTPLSALFYAHLAKLAGIPDGVLNIVPGFGPTAGAAISSHMDIDAVSFTGSIEVGREVL
QAAAWSNLKPVSLELGGKSPLIIFNDADIDKASELALFGIMSNKGEICVAGSRVFVQEEIYDEFEKKLVEKAKSWVVGDPFDPKSLQGPQADRNQLE
KILSYIEHGKREGATLLTGGNTVGNKGYYIEPTIFSNVKEDMLIARDEIFGPVLALMKFKTMEEAIKSANNTKYGLAAGIVTKNLDTANTMSRSIRA
GIVWINCYFTVGSDVPFGGYKMSGFGRDLGLQALHKYLQVKSVVTPIHNSPWL
>GmALDH2C2
MENLSNGHLESFVKIPTIKFTKLFINGEFLDSVSGKTFETVDPRTEEVIAEIAEANKEDVDIAVKAAREAFDCGPWPRMPGAERAKIMLKWSELIEQ
NAEEIAALDTIDGGKLFSWCKAVDVPEASNILRYYAGAADKIHGDVFKTSRDLHLYSLMEPVGVVGHIIPWNFPTVMFFAKVAPALAAGCTMVIKPA
EQTPLSSLFYAHLARLAGIPDGVLNVVPGFGSIAGAAISSHMDIDAVSFTGSTETGRKIMQAAALSNLKPVSLELGGKSPVLIFDDADVDKAVDLAL
FGILHNKGEICVAFSRVYVQEGIYDEFEKKVVEKAKTWVVGDPFDPKVQQGPQTSKAQYDKIISYIEHGKSEGATLLTGGKPAGNKGYYIEPTIFVN
VKEDMLIAQEEIFGPVMTLSKFKTIEDAIKKANNSKYGLAAGIVTKNLDIANTVSRSIRAGIIWINCFFAFDIDCPFGGYKMSGFGRDYGLEALHKF
LKVKSVATPIYDSPWL
>GmALDH2C3
MAALSNGHDASFFKMPSIKFTKLFINGEFVDSLSGKEFETIDPRTGEVITRIAEGAKEDIDVAVKAARDAFDYGPWPRMPGAERAKIMMKWADLIDQ
NIEEIAALDAIDAGKLYHWCKAVDIPAAANTIRYYAGAADKIHGEVLKASREFHAYTLLEPIGVVGHIIPWNFPSTMFVAKVSPSLAAGCTMVLKPA
EQTPLSALFYAHLAKLAGIPDGVLNVVPGFGQTAGAAISSHMDIDKVSFTGSTEVGREVMRAAANSNLKPVSLELGGKSPVIVFDDADVDKAAGLAL
MGILFNKGEICVAGSRVLVQEGIYDEFEKKLVEKANAWVVGDPFDPKVQQGPQVDKKQFEKILSYIEHGKKEGATLLTGGKRVGNKGYYIEPTIFSN
VKEDMLIVQDEIFGPVMALMKFKTIEDAIKIANNTRYGLASGIVTKSLDTANTVSRSIRAGIVWINCYFAFGDDIPYGGYKMSGFGRDFGMEALHKY
LQVKSVVTPIYNSPWL
>GmALDH2C4
MTSLTNGDAGSLNKVPTIKFTKLFINGDFVDSLSGKTFETIDPRTGDVIARISEGDKEDIDIAVKAARHAFDNGPWPRLPGSERARILLKWAEIIEE
NAEELAALDAIDAGKLYHMCRNVEVPAAANTLRYYAGAADKIHGEVLKMSREFHAYTLLEPLGVVGHITPWNFPNTMFYIKVAPSLAAGCTMVLKPA
EQTPLSALFSAHLAKLAGIPDGVINVVPGFGPTAGAALSSHMDVDKVSFTGSTQTGRVIMQAAAKSNLKQVSLELGGKSPLIIFDDADIDKATELAL
LGILYNKGEVCVASSRVFVQEGIYDEFEKKLVEKAKAWVVGDPFDPKVQQGPQVDKEQFEKVLSYIEHGKKEGATLLTGGKTVGNKGYFIEPTIFSN
IREDMLIAQDEIFGPVMALKKFKTTEEAIKSANNTKYGLAAGIVTKNLDTANTVSRSIRAGTIWINCYFAFGDDVPFGGYKMSGFGKDHGLEALHKY
LQVKSVVTPLYNSPWL
>GmALDH2C5
MSSLSNNSSSSHGNSFLQMPPIKFTKLFINGDFVDSLSGRTFETIDPRTEEVIARVSEGDKEDIDIAVKAARQAFDSGPWPRLPASERAKIMMKWAD
LIDENIEELAALDTVDAGKLNYINKVVEIPSATNALRYYAGAADKIHGEVLKMNGDFHAYTLLEPIGVVGHIIPWNAPSLSFFIKVSPSLAAGCTMV
LKPAEQTPLSALFYAHLAKLAGIPDGVLNIVPGFGPTAGAAISSHMDIDVVSFTGSIEVGREVMQAAARSNLKPVSLELGGKSPLIIFNDADIDKAA
QLALFGIMSNKGEICVASSRVFVQEEIYDEFEKKLVEKAKSWVVGDPFDPKSLQGPQADRNQLEKILSYIEHGKREGATLLTGGNTVGNKGYYIEPT
IFCNVKEDMLIARDEIFGPVLALMKFKTMEEAIKSANNTKYGLAAGIVTKNLDTANTMSRSIRAGIVWINCYLTVGSDVPFGGYKMSGFGRDLGLQA
LHKYLQVKSVVTPIHNSPWL
>GmALDH2C6
MNSNGYPASSFKIPTVKFTKLFINGHFVDSLSGGEFETIDPRTGEVIARIAEGTKEDIDLAVKASRLAFDHGPWPRMPAVERARIMMKWADLIDQHV
EEIAALDAIDAGKLYHMLKAIEIPATANTIRYYAGAADKIHGEVLKPAREFHAYTLLEPVGVVGHIIPWNFPSIMFVSKVSPCLAAGCTMVLKPAEQ
TPLSALFYAHLAKLAGIPDGVLNVVPGFGATAGAAICSDMDIDKVSFTGSTEVGREVMRAAANSNLKPVSLELGGKSPFIIFDDADLDKAVELALMA
VVYNKQQHIFISDNYLLLLSGFQGEVCAAGSRVFVQEGIYDEFEKRLVEKAKAWVVGDPFDPNVQQGPQVDKKQFEKILSYIEHGKREGATLLTGGK
RVGNKGYYIEPTIFSNVKEDMLIAQDEIFGPVIALMKFKTIEEAIKSANNSRYGLVAGVVTKSLDTANTMSRSIRAGVVWINCYFAFENDIPYGGCK
MSGFGKDSGLEALHKYLHVKSVVTPIYNSPWL
>GmALDH2C7
MAALSNGHGSSFFKMPPIKFTKLFINGEFVDSLSGREFETRDPRTGEVITRIAEGAKEDVDVAVKAARAAFDYGPWPRMPGAERAKIMMKWADLVDQ
NIEEIAALDAIDAGKLYHWCKAVDIPAAASTIRYYAGAADKIHGEVLKASREFHAYTLLEPIGVVGHIIPWNFPSTMFVAKVSPSLAAGCTMVLKPA
EQTPLSALFYAHLAKLAGIPDGVLNVVPGFGQTAGVAISLHMDIDKVSFTGSTEVGREVMRAAANSNLKPVSLELGGKSPVIVFDDADVDKAAELAL
LGILFNKGEICVAGSRVLVQEGIYDEFEKKLVEKAKAWVVGDPFDPKVQQGPQVDKKQFEKILSYIEQGKKEGATLLTGGKRVGNKGYYIEPTIFSN
VKEDMLIVQDEIFGPVMALMKFKTIEDAIKIANNTRYGLASGIVTKSLDTANTVSRSIRAGIVWINCYFAFGNDIPYGGYKMSGFGRDFGMEALHKY
LQVKSVVTPIYNSPWL
>GmALDH2C8
MANLSNSHSESFVKIPTVKFAKLFINGEFLDSVSGKTFETVDPRTEEVIAEIAEANKEDVDIAVKAAREAFDFGPWPRIPGAERAKIMLKWSQLIEQ
NAEEIAALDTIDGGKLFSWCKAVDVPEASNILRYYAGAADKIHGDVFKTSRNLHLYSLMEPVGVVGHIIPWNFPTVMFFAKVAPALAAGCTVVIKPS
EQTPLSSLFYAHLSKLAGIPDGVLNVVPGFGSIAGAAISSHMDIDAVSFTGSTETGRKIMQAAALSNLKPVSLELGGKSPLLIFDDADVDKAVDLAL
FGILHNKGEICVAFSRVYVQKGIYDEFEKKVVEKAKTWVVGDPFDPKVQQGPQTSKAQYDKILSYIEHGKSEGATLLTGGNPAGNKGYYIEPTIFAN
VKEDMLIAQEEIFGPVMTLSKFKTIEDGIKKANSSKYGLAAGIVTKNLDIANTVSRSIRAGIIWINCFFAFDIDCPFGGYKMSGFGRDYGLEALHKF
LKVKSVATPIYNSPWL
>GmALDH2C9
MAVFNSLHKTSTRFFVPTGRNSGKWGNVNRFSTAAAVEELIIPQVPITYTKHLINGQFVDADAASGKTFPTYDPRTGEVIARVAEGDAEDINRAVSA
ARKAFDEGPWPKMTAYERCQIILRFADLTWNNGKPYEQWATSELPTFVRLFRYYAADKIHGLTVPADGNYHVETLHEPIGVAGQIIPWNFPLLMFAW
KVGPALACGNTVILKTAEQTPLTALYVAKAGLPPGVLNVVSGYGPTAGAALASHMDVDKLAFTGSTETGKVVLELAARSNLKPVILNLEGNLLSLGQ
CCCAGSRTFVHERIYDEFLEKAKARALKRVVGDPFIKGVEQGPQVCFASTLRQNIIDCVLSYHFCSFYSYYKATLECGGDRIGSKGFFVQPTVFSNV
QRVGTVWINCFDVFDAAIPFGGYKMSGISREKGIYSLNNYLQVKAVVSPVKNPAWL
>GmALDH3F1
MDIGGGVEEPVRELRQYFKTGKTKSVTWRKNQLTSLIDLVHENEDAIFKALHKDLGKHPVEAYRDEVGGVEKSASKALSCVEKWMAPKKSDIPFLFF
PAKGEVLSEPLGVVLIISSWNFPIILALDPIIGAISAGNVVVIKPSEQAPACSSFLANTIPRYLDSNAIKVIEGGEDVCEQLLRQKWDKIFFTGSPR
VASVVMSAAAKNLTPVTLELGGKCPAILDSLPNPSEFELAVKRIVGGKWGPCSGQACIGIDYLLVEEKFSSAVIKLLKKFIRRFYGENPVESKVISR

IINKQHFERLCNLLKDPLVAASIVHGGSVDEENLFIEPTILLDPPLDSEIMAEEIFGPLLPIITLDKIQESIEFINAKPKPLAIYAFTKDETFKRKI
LSETSSGSVVFNDTMVQFLCDTLPFGGVGQSGLGRYHGKYSFDTFSHEKAVMHRKLFLEIEPRYPPWNKFKLEFIRLAYRLNYFGLVLHMLGLKR
YN
>GmALDH3F2
MSGEETQRNVFGAETASSLVKELRDNFGKGTTRSYEWRVSQVKALLKAVVENEDQIVGALCSDLAKPPLETVVYEIGMFQNSCEVILKELKHWMTPE
KVKTSIRTFPSSAEIVPEPLGVVLVISAWNYPILLSLDPVVGAIAAGNAVVLKPSEIAPATSSVLAKLIEKYMDNSFVRVVEGAVDETTALLQQKWN
KIFYTGNGRVGKIVMTAAAKHLTPVVLELGGKSPVVVDSNNNLLVAARRIIAGKWGLNNGQACISPDYVITTKDYAPKLVDTLKTELESFYGRNPLE
SEDLSRIVSSNHFARLSKLLNDDKVSGKIVYGGEKDEKKLRIAPTILLDVPQDSSIMGEEIFGPLLPIITVNKLEESIDVINSGAKPLAAYVFTTDN
KFKEQFVKNVSAGGLLVNDTALHLVVDTLPFGGVGESGMGAYHGKFSFDAFTHKKAVLYRSFAGDSAIRYPPYTDTKLRLMKALVGGRILGIIRALF
GWS
>GmALDH3H1
MSVEEMQSQKRNVFDAETASSLVKELRDNFGSGRTRSYEWRVSQVKALLKAVVDNEEQIVDALRSDLAKPPLETIVYEVGMFKNSCEVILKELKQWM
KPEKVKTSIRTFPSSAEIVPEPLGVVLVISAWNYPILLSLDPVVGAIAAGNAVVLKPSEIAPASSSLLLKLIEKYCDNSFIRVVEGAVDETTALLQQ
KWDKIFYTGNGKVGRIVMTAAAKHLTPVVLELGGKSPVVVDSNVDLQIAARRIISGKWGLNNGQACISPDYVITTKDCAPKLVDALKTELEKCYGKN
PLESEDLSRIVTSNHFARLSKLLDDDKVAGKIVYGGEKDEKKLRIAPTLLLDVPRDSLIMGEEIFGPLLPIITVNKVEESIDLINSGTKPLAAYIFT
TNKKLKEQFVMNVPAGGLLVNDTVLHLVVDTLPFGGVGESGMGAYHGKFSFDAFTHKKAVLYRSFAGDSSLRYPPYTDTKLRLMKALIGGRFLGIIR
ALFGWS
>GmALDH3H2
MSSTPQDSVKTTASAKNTAFDAEAASRLVNELRRNFASNKTRSYEWRLSQLNALEKLVVVHEQEIVDALRNDLGKPPLETVAYEIAMLKNSCRIALK
ELKHWMTPEKVKTSIATFPSSAEIVSEPLGVVLVISAWNYPFLLSLDPVVGAIAAGNAVVLKPSEIAPATSSLLAKLIGDYLDNSCIRVVEGAVDET
SALLQQKWDKIFYTGNGRVARIVMAAASKHLTPVVLELGGKSPVVVDSNINLKVATRRIIAGKWGSNNGQACISPDYIITTKDYAPKLVDALKTELE
KFYGKNPLESKDLSRVVNSNHFNRLTKLLDDDKVSGKIVYGGQKDENKLKISPTVLLDVPRDSLIMNEEIFGPLLPILTVDKLEESFDVINSGPKPL
AAYIFTNNKKLKEQFVMTISAGGLVVNDTTLHLAVHTLPFGGVGESGVGAYHGKFSFEAFSHKKAVLYRKFIGDAPVRYPPYTNTKMRLLKAIIGGG
IHGIVRALFGW
>GmALDH3H3
MSSSTPDSDKTTTSSKKSAFDALAASRLVTELRGNFASGKTRSYEWRLLQLNAIAKLVVDHEQEIVDALRNDLGKPPLETVAYEIAMLKNSCRIALK
ELKHWMTPEKVKTSIATFPSSAEIVSEPLGVVLVISAWNYPFLLSLDPVIGAIAAGNAVVLKPSEIAPATSSLLAKLLGDYLDNSCIKVVEGAVDET
SALLQQKWDKIFYTGNGRVARIVMAAASKHLTPVVLELGGKSPVVVDSNINLKVATRRIIAGKWGSNNGQACISPDYIITTKDYAPKLVDALKTELE
KFYGKNPLESKDLSRIVNSNHFNRLTKLLDDDKVSGKIVYGGEKDESKLKISPTVLLDVPRDSLIMNEEIFGPLLPILTVDKIEESFDVINSGSKPL
AAYIFTNTKKLKEQFVMTISAGGLVVNDTTLHLAVHTLPFGGVGESGVGAYHGKFTFEAFSHKKAVLYRRFIGDAPVRYPPYTNTKMRLLKALIGGG
ILGIIRALFGW
>GmALDH3H4
MKSLCLGPFLAASAPVGRRAYGGHLSRKCFQKQLHFHSRCVAFSSFICSATISVMPELEEKQVFDGEKANLLVKDLRKSFDSGMTKSYGWRVSQLEA
IAKMLEEKEKEITEALYKDLGKPRLEAFITEISQAKSSCSEALKELKEWMKPEKVNTSITTYPSSAEIVPEPLGVVLVISTWNFPFLLSMDPVIGAI
SAGNAVVLKPSEISPATSSLLANLIEQYLDNSTIRVVEGAIPETSALLDQKWDKILYTGSARVGRIVMAAAAKHLTPVILELGGKCPAVVESDVNLQ
VTARRIIAGKWACNSGQACISVDYIITRKEFAPKLVDALKEELEQFFGKDPMESKDMSRIVSPNQFARLVNLLDEDKVSDKIVLGGQRDEKKLKIAP
TIILGVPEDAMIMQEEIFGPIMPIVTVDNIEDCYSIIKSKPKPLAAYLFTNNEQLKKDYVDKISSGGMLINDAVIHVATRGLPFGGVEESGMGCYHG
KFSFDSFSHRKSVLYRSFDADSTIRYPPYTPQKEKLLKALISGNIVQIILSLLGWS
>GmALDH3I1
MEITMQTLERDLNDTRGYYESGKTKEESWRESQLKGLRRFLLEKQVDIMNALMHDLGKHQLEAFRDEIGTLIKTVNLALKSLKDWMSGKKAALPQLA
LLTSAEIVPEPLGLVLIISSWNFPIGISLEPLIGAVAAGNAAVLKPSELSPACSSLLASSLPTYLDDKAIKVIQGGPQETQQLLEQRWDKIFFTGSA
RVGRIVMSSAVKHLTPVTLELGGKCPAVVDSLSSSWDKEVTVKRIIVGKYGTCAGQACITIDYVLVEKGYCLKLVELMKVWIKKMFGQNPRKSKTIA
KIVNKHHFSRLKNLLADKQVKGSVVYGGSMDEQNLFIEPTILVDPPLEAAIMSEEIFGPLLPIITVEKIEDSIKFINARPKPLALYVFTKNHTLQRR
MISETSSGSVTINDAVLQYAADTIPFGGVGESGFGMYHGKFSFDTFSHQKAIVRRSFLTDFWYRYPPWTLNKLQLLEVSYNYDYLGLLLVLLGLKRP
SKRLIADHV
>GmALDH3J1
MEIIMPSLERDLNDTRGYYESGKTKEASWRESQLKGLRRFLIEKQEDIMNALMHDLGKHQLEAFRDEIGTLIKTLNLALKSLKHWMSGKKAALPQLA
LLTSAEIVPEPLGVVLIISSWNFPFGISLEPLIGAVAAGNAAVLKPSELSPACSSLLASNLSTYLDNKAIKVIQGGPKETQQLLEQRWDKIFFTGSA
HVGKIVMSAAVKHLTPVTLELGGKCPAVVDSLSSSWNIEVAVKRIIVGKYGACAGQACIAIDYVLVEKVYCFKLVELMKVWIKKMCGENPQQSKTIA
KIVNKHHFSRLKNLLADKKVKESVIYGGSMDEQNLFIEPTILVDPPLEAAIMSEEIFGPLLPIITVEKIEDSIKFINSRPKPLALYVFTKNQTLQRR
MISETSSGSVTINDAILQYAVDTVPFGGVGESGFGMYHGKFSFDTFSHQKAIVRRSFLTDFWYRYPPWTLNKLQLLEVSYNYDYLGLLLVLLGLKRP
SKRLISDHV
>GmALDH3J2
MKYTGEALGRDLENVRKYYGSGKTKEASWRESQLKGLHNFLVEKEEEILRALKHDLGKHYVEAFRDEVGTLMKTLNLASKSLKNWMAGKEAKLPRIA
LLSSAEIVPEPLGLVLIISSWNFPFGLSLEPLIGAIAAGNSVVLKPSELSPTCSSLLATFLPTYLDNNAIKVIQGGPEVGELLLQQRWDKIFFTGSA
RVGRIVMSAAAVHLTPVTLELGGKCPAIIDSLSSSWDKEVAVKRILVAKFGACGGQACIAIDYVLVEKSFSSTLVTLMKEWIKKLFGENPKVSNTIA
RIVNKNHFMRLKNLLTEPRVKESVVYGGSMDENDLFIEPTILLDPPLDSAIMAEEIFGPVLPIITVEKIEESVEFISSRPKALAIYAFTKNQTLQRR
LVSETSSGSLVFNDAILQYVADTLPFGGVGECGFGKYHGKFSFDAFSHHKAVARRSYLTDFWFRFPPWTLNKLQLLEVSYNLDYLGILLVLLGLKKS
KRSLFQACN
>GmALDH3J3
MEYSVETLERDLKNTRKYYGSGKTKEAPWRESQLKGLHNFLVEKEEEIVTALKHDLGKHYVEAFRDELGTLMKTLNLATKSLKNWMAGKEAKLPRIA
LLSSAEIVPEPLGLVLIISSWNFPFGLSLEPLIGAVAAGNSVVLKPSELSPTCSSLLATFLPTYLDNNAIKVIQGGPEVGKLLLQQRWDKIFFTGSA
RVGRIVMSAAAVHLTPVTLELGGKCPALIDSLSSSWDKEVAVKRILVAKFGSCAGQACIAIDYVLVEKSFSSTLVTLMKEWIKKMFGENPKASNSIA
RIVNKNHFMRLQNLLTEPRVKESVVYGGSMDENDLFIEPTILLDPPLDSAVMAEEIFGPVLPIITLEKIEDSVEFISSRPKALAIYAFTKNQTLQRR
MVSETSSGSLVFNDAILQYVADTLPFGGVGECGFGKYHGKFSFDAFSHHKAVARRSYLTDFWFRFPPWTLDKLQLLEVSYNLDYLGILLVLLGLKKS
KRSLFQACN

>GmALDH3J4
MRPPHYKYSCPHEWEGSVEENKKEKENNQYDNFAILVVDQGRSIAMDIGGEVEETVRELRQYFKTGKTKSVTWRKNQLTALLDLVHENEDAIFKALH
QDLGKHPVEAYRDEVGGVEKSASNALSCVEKWMAPKKSDIPFLFFPAKGEVLSEPLGVVLIFSSWNFPIILTLDPIIGAISAGNVVVIKPSEQSPAS
SSFLATTIPRYLDSNAIKVIEGGPDVCEQLLLQKWDKIFFTGSPRVASVVMSAAAKNLTPVTLELGGKCPAILDSLPNPLEFKLAVKRIVGGKWGPC
SGQACIAIDYLLVEKKFSYALIELLKKIIRRFYGENPVESKVISRILNKQHFERLCNLLKDPLVAASIVHGGSVDEENLFIEPTILLDPPLDSQIMS
EEIFGPLLPIITMDKIQESIEFINAKPKPLAIYAFTKDETFKRNILSETSSGSVVFNDTMVQFLCDTLPFGGVGQSGFGRYHGKYSFDTFSHEKAVM
HRKLFLEIEPRYPPWSKFKLEFIRLAYRLNYFGLLLHMLGLKRYK
>GmALDH5F1
MAALNLCRMALRSSKLLYRPYNLLSVQLQMQMQPSSPPLTRKMSTDAQSIASQLNSSGLLRTQGLIAGKWSDAYDGKTIKVYNPATGESVVDVACMG
GRETNDAISAAYDAYGSWSKTTAAERSKLLRKWYDLLMVHKEELAQLITLEQGKPLKESVGEIVYGAGFIEFAAEEAKRIYGDIVPAPFSDRRLFVL
KQPVGVVGAITPWNFPLAMITRKVGPALACGCTVVIKPSELTPLTALAAVELSIQAGIPPGVVNVVMGNAPDIGDALLASPQVRKITFTGSTAVGKK
LMAGSAETVKKVSLELGGNAPCIVFDDADLDVAVKGTLAAKFRNSGQTCVCANRIIVQEGIYEKFANALRDAVQNMKVGDGFSEGVSQGPLINEAAV
KKVESLIHDATSKGAKVILGGKRHSLGLTFYEPTVISDVNSDMHISREEAFGPVAPLLRFKTEEEAIRIANDTNAGLGSYVFTNSIQRSWRVAEALE
YGLVGVNEGVISTEVAPFGGFKQSGLGREGSKYGMDEYLEIKYVCFGNMNKE
>GmALDH5F2
MAALNLCRMALRSSKLLSRPYHRLSVQLQMQMQPSSPPLTRKMSMDAQSVASQLNSSGLLRTQGLIGGKWSDAYDGKTIKVYNPATGESIVDVACMG
GRETNDAISAAYDAYGSWSKTTAAERSKFLRKWYDLLMVHKEELAQLITLEQGKPLKESVGEINYGAGFIEFAAEEAKRIYGDIIPAPLSDRRLFVL
KQPVGVVGAITPWNFPLAMITRKVGPALACGCTVVIKPSELTPLTALAAAELSIQAGIPPGVVNVVMGNAPDIGDALLASPQVRKITFTGSTAVGKK
LMAGSAETVKKVSLELGGNAPCIVFDDADLDVAVKGTLAAKFRNSGQTCVCANRIIVQEGIYEKFANALRDTVQNMKVGDGFSEGVAQGPLINEAAV
KKVESLIHDATSKGAKVILGGKRHSLGFTFYEPTVISDVNSDMRISREEAFGPVAPLLRFKTEEDAIRIANDTNAGLGSYIFTNSIQRSWRVAEALE
YGLVGVNEGVISTEVAPFGGFKQSGLGREGSKYGMDEYLEIKYVCLGNMHKA
>GmALDH6B1
MLRLSIQRVRKLNFLSPQISALGRSHLSTAAEPSSSKSNPPRVPNLIGGSFVDSKASTVIDVINPATQEVVSQVPLSTDEEFKEAVSAAKKAFPSWR
NTPITTRQRVMLKLQELIRRDMDKLALNVTTEQGKTLKDAQGDVFRGLEVVEHACGMATLQMGEYVSNVSHGIDTYSIREPLGVCAGICPFNFPAMI
PLWMFPMAITCGNTFVLKPSEKDPGASVMLAELALEAGLPEGVLNIVHGTHDIVNAICDDDDIKAISFVGSNVAGMHIYSRAAAKGKRVQSNMGAKN
HAIVMADANVDATLNALVAAGFGAAGQRCMALSTVVFVGGSKPWEDKLLEHAKALKVNAGTEPDTDLGPVISKQAKERIHRLVQSGVESGARLLLDG
RNIVVPGYESGNFIGPTILSDINANMECYKEEIFGPVLLFMEADSLEEAINIINSNKYGNGASIFTTSGVAARKFQTEIEAGQVGINVPIPVPLPFF
SFTGNKASFAGDLNFYGKAGVNFYTQIKTITQQWKDSTGGSKINLAMPTSQK
>GmALDH6B2
METRMLRLSIQRVRKLNFLRPQISALGRSHLSTAAEPSSSKSNPPRVPNLIGGSFVDSKASTVIDVINPATQEVVSQVPLSTHEEFKAAVSAAKEAF
PSWRNTPITTRQRVMLKLQELIRRDMDKLALNVTTEQGKTLKDAQGDVFRGLEVVEHACGMATLQMGEYVSNVSHGIDTYSIREPLGVCAGICPFNF
PAMIPLWMFPMAVTCGNTFVLKPSEKDPGASVMLAELALEAGLPEGVLNIVHGTHDIVNAICDDENIKAISFVGSNVAGMHIYSRAAAKGKRVQSNM
GAKNHAIVMPDANVDATLNALVASGFGAAGQRCMALSTVVFVGGSKPWEDKLLERAKALKVNAGTEPDTDLGPVISKQAKERIHRLVQSGVESGARL
LLDGRNIVVPGYESGNFIGPTILSDINANMECYKEEIFGPVLLFMEADSLEEAINIINSNKYGNGASIFTTSGVAARKFQTEIEAGQVGINVPIPVP
LPFFSFTGNKASFAGDLNFYGKAGVNFYTQIKTITQQWKDSTGGSRINLAMPTSQK
>GmALDH6B3
MANSHLSTPSELFSRQHKPPRVPNLIGGSFLDSKSLTFIDVINPATQEVVSQVPCTTDEEFKAAVSAAKKAFPSWRKTPITKRQRVMLKFQELIRRD
MDKLALNVTTEQGKTLKDAQGDVFRGLEVVEHACGMATLQMGEYVSDVSSGIDTYSIREPLGVCAGICPFNFPAMIPLWMFPVAVTCGNTFILKPSE
KVPGASVMLAELAMEAGLPEGVLNIVHGTHDIVNAICDDDDIKAISFVGSNVAGMHIYARAAAKGKRVQANMGAKNHAVVMPDASVDATVNALVAAG
FGAAGQRCMALSTVVFVGDSKLWESKLVEHAKALKVNVGTEPDADLGPVISKQAKERIHRLIQSGVESGARLVLDGRNIVVPGYESGNFIGPTILSD
VTANMECYKEEIFGPVLLLTEADNLEEAINIINENKYGNGASIFTTSGVAARKFQTEIEAGQVGINVPIPVPLPFFSFTGNKASFAGDLNFYGKAGV
NFYTQIKTVTQQWKDSASESKINLAMPTSQKS
>GmALDH7B1
MGSDNHQNLEFLKEIGLGSSNIGSYINGQWKATGSSVTSVNPSNNQSIAQVTEATLQDFEEGLRACSEAAKTWMTIPAPKRGEIVRQIGEALRAKLD
PLGRLVSLEMGKILPEGIGEVQEIIDMCDYCVGLSRQLNGSIIPSERPDHMMFEVWNPLGIVGVISAFNFPCAVLGWNACIALVCGNCVVWKGAPTT
PLITIAVTKLVAEVLERNKLPGAIFTSFCGGADIGQAIAKDTRIPLVSFTGSSKVGLMVQQTVNERFGKCLLELSGNNAIIVMDDADIKLAVRSILF
AAVGTTGQRCTTCRRLFLHESIYTDVLDQLVEVYKQVKIGNPLEKGTLVGPLHTRTSVENFQKGISVIKSQGGKILTGGSVLESGGNFVQPTIVEIS
PDAPVVKEELFGPVLYVMKFQTLEEAIALNNSVPQGLSSSIFTQRPGTIFKWIGPRGSDCGIVNANIPTNGAEIGGAFGGEKATGGGREAGSDSWKQ
YMRRSTCTINYGSELPLAQGINFG
>GmALDH7B2
MGSDNTNLEFLKEIGLGSSNIGSYINGQWKATGSSVTSVNPSNNQSIAQVTEATLQDYEEGLQACSEAAKTWMTIPAPKRGEIVRQIGEALRAKLDP
LGRLVSLEMGKILPEGIGEVQEIIDMCDYCVGLSRQLNGSIIPSERPDHMMFEVWNPLGIVGVITAFNFPCAVLGWNACIALVCGNCVVWKGAPTTP
LITIAVTKLVAEVLERNKLPGAIFTSFCGGADIGQAIAKDTRIPLVSFTGSSKVGLMVQQTVNERFGKCLLELSGNNAIIVMDDADIKLAVRSILFA
AVGTAGQRCTTCRRLFLHESIYADVLDQLIGVYKQVKIGNPLEKGTLVGPLHTPTSVENFQKGISVIKSQGGKILTGGSVLESAGNFVQPTIVEISP
DAPVVKEELFGPVLYVMKFQTLEEAIALNNSVPQGLSSSIFTQRPGTIFKWIGPRGSDCGIVNANIPTNGAEIGGAFGGEKATGGGREAGSDSWKQY
MRRSTCTINYGSELPLAQGINFG
>GmALDH10A1
MSIPIPHRQLFIDGDWKVPVLKNRIPIINPSTQHIIGDIPAATKEDVDLAVAAAKAALSRNKGADWASASGSVRARYLRAIAAKITEKKPELAKLEA
IDCGKPLDEAAWDIDDVAGCFEFYADLAEKLDAQQKAHVSLPMDTFKSYVLKEPIGVVALITPWNYPLLMATWKVAPALAAGCAAILKPSELASVTC
LELAEICKEVGLPPGVLNILTGLGPEAGAPLAAHPDVDKIAFTGSSATGSKIMTAAAQLIKPVSLELGGKSPIIVFEDVDLDKAAEWTIFGCFWTNG
QICSATSRLIESIATEFLNRIVKWVKNIKISDPLEEGCRLGPIVSEGQYEKILKFISNAKSEGATILTGGSRPEHLKKGFFVDQLEEVFGPVLCVKT
FSTEEEAIDLANDTVYGLGSAVISNDLERCERITKAFKAGIVWINCSQPCFTQAPWGGIKRSGFGRELGEWGLDNYLSVKQVTQYISDEPWGWYQSP
SRL
>GmALDH10A2
MAISIPSRQLFIDGEWKVPLLNNRFPIINPATEDIIGHIPAATKEDVDLAVDAAKRAFSHNKGKDWSSAPGSVRARYLRAIASKITEKKDELGKLEA
IDCGKPLDEALADLDDVIGCFNYYAELAEGLDAKQNAPVSLPMETFKSYVLKEPIGVVALITPWNYPLLMATWKVAPALAAGCTAILKPSELASVTC

LELAEICREVGLPPGVLNIVTGLGNEAGAPLSSHPDVDKISFTGSSATGSRIMTAAAQLTKPVSLELGGKSPIIVFEDVDLDKTAEWTIFGCFFTNG
QICSATSRLIVHESIATEFVNRLVQWAKNIKISDPFEEGCRLGPIVSEGQYKKVLNCISTAKSEGATILIGGSRPEHLKKGYFVEPTIITDVTTSMQ
IWREEVFGPVLCVKTFSTEEEAIELANDTHYGLGSAVMSKDLERCERISKAIQAGIVWINCAQPSFIQAPWGGVKRSGFGRELGEWGLENYLSVKQV
TKYISDEPWGWYQSPSKL
>GmALDH11A1
MAAGTGLFAEILDGDAYKYYADGEWKKSASGKSVSIINPTTRKTQYKVQACSQEEVNKVMDLAKSAQKLWAKTPLWKRAELLHKAAAILKEHKTPIA
ECLVKEIAKPAKDAVMEVVRSGDLVSYTAEEGVRILGEGKFLVSDSFPGNERTKYCLTSKIPLGVILAIPPFNYPVNLAVSKIAPALIAGNSIVLKP
PTQGAVSALHMVHCFHLAGFPKGLINCVTGKGSEIGDFLTMHPGVNCISFTGGDTGISISKKAGMIPLQMELGGKDACIVLEDADLDLVAANIIKGG
FSYSGQRCTAVKVVLVMESVADALVEKVKAKVAKLTVGPPEDDCDITPVVSESSANFIEGLVLDAKEKGATFCQEYKREGNLIWPLLLDNVRPDMRI
AWEEPFGPVLPVIRINSVEEGIHHCNASNFGLQGCVFTKDVNKAIMISDAMETGTVQINSAPARGPDHFPFQGIKDSGIGSQGITNSINMMTKVKTT
VINLPSPSYTMG
>GmALDH11A2
MAAGTGLFAEILDGDVYKYYADGEWKKSASGKSVAIINPTTRKTQYKVQACSQEEVNKVMDLAKSAQKLWAKTPLWKRAELLHKAAAILKEHKAPIA
ECLVKEIAKPAKDAVTEVVRSGDLVSYTAEEGVRILGEGKFLVSDSFPGNERTKYCLTSKIPLGVILAIPPFNYPVNLAVSKIAPALIAGNSIVLKP
PTQGAVSALHMVHCFHLAGFPKGLINCVTGKGSEIGDFLTMHPGVNCISFTGGDTGIAISKKAGMIPLQMELGGKDACIVLEDADLDLVAANIIKGG
FSYSGQRCTAVKVVLVMESAADALVEKVKAKVAKLTVGPPEDDCDITPVVSESSANFIEGLVLDAKEKGATFCQEYKREGNLIWPLLLDNVRPDMRI
AWEEPFGPVLPVIRINSVEEGIHHCNASNFGLQGCVFTKDVNKAIMISDAMETGTVQINSAPARGPDHFPFQGIKDSGIGSQGITNSINMMTKVKTT
VINLPSPSYTMG
>GmALDH11A3
MAGSGTFAEIIDGDVFKYYAQGHWNKSSSGKFVPIINPTTRKTHFKVQACTQKEVNRVMESAKTAQKSWAKTPLWKRAELLHKAAAILKEHKAPIAE
CLVKEIAKPAKDAVTEVIRSGDLVSYCAEEGVRILGEGKFLVSDSFPGNERTKYCLTSKIPLGVVLAIPPFNYPVNLAVSKIAPALIAGNSIVLKPP
TQGAVAALHMVHCFHLAGFPEGLISCVTGKGSEIGDFLTMHPGVNCISFTGGDTGIAISKKAGMVPLQMELGGKDACIVLEDADLDLAAANIVKGGF
SYSGQRCTAVKVALVMESVANTLVKRINDKIAKLTVGPPEIDSDVTPVVTESSANFIEGLVMDAKEKGATFCQEYVREGNLIWPLLLDNVRPDMRIA
WEEPFGPVLPVIRINSVEEGIHHCNASNFGLQGCVFTRDINKAMLISDAMETGTVQINSAPARGPDHFPFQGLKDSGIGSQGITNSINMMTKVKTTI
INLPAPSYTMG
>GmALDH12A1
MCLLRVLSAEFIFTICRFAHSLPFATVQAEEISDSRPAEVLNLVQGKWAGSSNWNTVVDPLNGDSFIKVAEVDETGIQPFVESLSSCPKHGVHNPFK
APERYLMFGEISAKAAHMLSLPKVSDFFTRLIQRVSPKSYQQAFGEVYVTQKFLENFCGDQVRFLARSFGVPGNHLGQQSHGFRWPYGPVAIITPFN
FPLEIPVLQLMGALYMGNKPVLKVDSKVSIVMDQMLRLLHNCGLPLEDVDFINSDGKTMNKLLLEANPRMTLFTGSSRVAEKLAVDLKGRVKLEDAG
FDWKILGPDVLQEDYIAWVCDQDAYACSGQKCSAQSLLFMHENWSKTSLLSKLKDLADRRKLADLTVGPVLTVTTDSMLEHINKLLEIPGSKLLFGG
QPLEDHSIPPIYGAMKPTAVYVPLEEIMKAKNFELVTREIFGPFQIVTDYKSSQLSVVLDALERMHNHLTAAVVSNDPLFLQEVIGQSVNGTAYAGL
RARTTGAPQNHWFGPAGDARGAGIGTPEAIKLVWSCHREIIYDFGPVPKNWEVPPST
>GmALDH12A2
MFMFLVSRVTKDSISRNRNAFASFAFSSRCAHSLSFATVEAEEISGSRPAEVLNLVQGKWVGSSNWNTIADPLNGDSFIKVAEVDETGIQPFIKSLS
SCPKHGVHNPFKAPERYLMYGDISTKAAHMLSLPKVSDFFTKLIQRVSPKSYQQAFGEVYVTQKFLENFCGDQVRFLARSFGVPGNHLGQQSHGFRW
PYGPVAIITPFNFPLEIPVLQLMGALYMGNKPVLKVDSKVSIVMEQMLRLLHTCGLPAEDVDFINSDGKTMNRLLLEANPRMTLFTGSSRVADKLAV
DLKGRVKLEDAGFDWKILGPDVHQEDYIAWVCDQDAYACSGQKCSAQSLLFMHENWSKTSLLSKLKDLAERRKLEDLTIGPVLTCTTGMMLEHKNKL
LEIPGSKLLFGGSPLENHSIPPIYGAIKPTAVYVPLEEIMKDKNFDLVTKEIFGPFQVITDYKNSQLSVVLDAVERMHNHLTAAVVSNDPLFLQEVV
GNSVNGTTYAGLRARTTGAPQNHWFGPAGDARGAGIGTPEAIKLVWSCHREVIYDFGPVPKDWKTPQST
>GmALDH12A3
MFKLLVSRAARVSTPHNHNAFASFAFSRYAHSLPFATVEAEEISGSRAAEVLNLVQGKWVGSSNWNTVVDPLNGDSFIKVAEVDETGIQPFVESLSS
CPKHGAHNPFKAPESLLGVNGMVVLILFRYLMFGEISAKAAHMLSLPKVLDFFTRLIQRVSPKSYQQAFGEVYVTQKFLENFCGDQVRFLARSFAVP
GNHLGQQSHGFRWPYGPVAIITPFNFPLEIPVLQLMGALYMGNKPVLKVDSKVSIVMEQMLRLLHTCGLPLEDVDFINSDGKTMNKLLLEGNPRMTL
FTGSSRVAEKLAVDLKGRVKLEDAGFDWKILGPDVHQEDYVAWVCDQDAYACSGQKCSAQSLLFMHENWSKTSLLSKLKDLAERRKLADLTIGPVLT
VTTDSMLEHVNKLLEIPGSKLLFGGSPLENHSIPPIYGAIKPTAVYVPLEEIMKDKNFELVTKEIFGPFQVITDYQNSQLAVVLDALERMHNHLTAA
VVSNDPLFLQEVIGKSVNGTTYAGLRARTTGAPQNHWFGPAGDARGAGIGTPEAIKLVWSCHREIIYDFGPVPKNWEVPPST
>GmALDH18B1
MELLQNGHKNFVSIKPSELPLTNGAALTLLNSLSKTQYLGNIDPSRVFVTKVKRIIVKVGTAVVTRSDGRLALGRIGALCEQLKELSSQGYEVILVT
SGAVGLGRQRLRYRKLANSSFSDLQKPQEELDGKACAAVGQSSLMALYDTMFSQLDVTSSQLLVNDGFFRDSGFRKQLSDTVNSLLDLRVIPIFNEN
DAVSTRKAPYEDSSGIFWDNDSLAGLLALELKADLLVLLSDVEGLYSGPPSDPNSRLIHTYIKEKHQGEITFGDKSRLGRGGMTAKVNAAVCAAHAG
IPVIITSGYATNNIIRVLQGERIGTVFHKDAHLWTNIKEVSAREMAVAAREGSRRLQILKSEERRKILLAIADALETSESMIRHENEADVADAVATG
YEKSLMSRLILKQEKISSLAKSVRMLADMEEPIGQILKRTELVDKLILEKISCPLGVLLVIFESRPDALVQIAALAIRSGNGLLLKGGKEARRSNAI
LHKVITSVMPDTVGDKLIGLVTSRDEILDLLKLDDVIDLVVPRGSNKLVSQIKESTKIPVLGHADGICHVYVDKSANIDMAKQIVRDAKTDYPAACN
AMETLLVHKDLSNNGGLHELVLELQREGVKMFGGPRASGLLNIAETNTFHHEYSSLACTVEIVEDVFAAIDHINQHGSAHTECIVTEDSEVAETFLS
QVDSAAVFHNASTRFCDGARFGLGAEVGISTSRIHARGPVGVEGLLTNRWILRGSGHVVDGDQGINYTYKELPLKA
>GmALDH18B2
MADPSRSFMKDVKRVIIKVGTAVVTREEGRLAVGRLGALCEQIKQLNSLGYDIILVSSGAVGIGRQRLRYRKLINSSFADLQKPQHELDGKACAAVG
QNSLMALYDTLFTQLDVTSAQLLVTDNDFRDKDFRKQLTETVKSLLSLKVIPVFNENDAVSTRKAPYEDSSGIFWDNDSLSALLALELKADLLVLLS
DVEGLYSGPPSDPHSKLIHTYIKEKHQNEITFGDKSRVGRGGMTAKVKAAVHAADAGIPVVITSGFAAENIINVLQGQRIGTLFHKDAHEWVQVKEV
DAREMAVAARECSRRLQAISSEERNQILHKIADALEANEKIIRTENEADIAVAQEAGYEKSLVARLAIKPGKIASLANNMRIIANMEDPIGQVLKRT
ELSDGLILEKTSSPLGVLLIVFESRPDALVQIASLAIRSGNGLLLKGGKEARRSNAILHKVITEAIPDTVGGKLIGLVTSREEIPELLKLDDVIDLV
IPRGSNKLVSQIKSSTKIPVLGHADGVCHVYVDKSANVEMARRIVLDAKIDYPAACNAMETLLVHKDLIEKGWLNDIVVDLRTEGVKLYGGPRASSL
LNIPQAQTFHHEYSSLACTVEIVDDVYAAIDHINLYGSAHTDSIVAEDKEVANVFLRQVDSAAVFHNASTRFSDGARFGLGAEVGISTSRIHARGPV
GVEGLLTTRWILKGSGQVVDGDKGIVYTHKDIAT
>GmALDH18B3

MELLQNGHKNLVSIKPSELPLLNGAALTLLNSLSETHEYYGNIDPSRVFVTKVKRIIVKVGTAVVTRSDGRLALGRIGALCEQLKELSSQGYEVILV
TSGAVGLGRQRLRYRKLANSSFSDLQKPQGELDGKACAAVGQSSLMALYDTMFSQLDVTSSQLLVNDGFFRDSGFRKQLSDTVNSLLDLRVIPIFNE
NDAVSTRKAPYEDSSGIFWDNDSLAGLLALELKADLLVLLSDVEGLYSGPPSDPNSKLIHTYVKEKHQGEITFGDKSRLGRGGMTAKVNAAVCAAHA
GIPVIITSGYATNNIIRVLQGERIGTVFHKDAHLWTNIKEMSAREMAVAAREGSRQLQILKSEDRRKILLAIADALEKNESMIRHENEADVADAVVA
GYEKSLISRLTLKQEKISSLAKSVRLLADMEEPIGQILKRTELVDKLILEKTSCPLGVLLVIFESRPDALVQIAALAIRSGNGLLLKGGKEARRSNA
ILHKVITSVMPDTVGDKLIGLVTSRDEIPDLLKLDDVIDLVVPRGSNKLVSQIKESTKIPVLGHADGICHVYVDKSANFDMAKQIVRDAKTDYPAAC
NAMETLLIHKDLSNNGGLNELVLELQREGVKMFGGPRASGLLNIAETNTFHHEYSSLACTVEIVEDVFAAIDHINQHGSAHTECIVTEDSEVAETFL
SQVDSAAVFHNASTRFCDGARFGLGAEVGISTSRIHARGPVGVEGLLTNRWILRGSGHVVDGDQGIDYTYKELPLKA
>GmALDH18B4
MADRSRSFMKDVKRVVIKVGTAVVTREEGRLAVGRLGALCEQIKQLNSLGYDIILVSSGAVGIGRQRLRYRKLINSSFADLQKPQLELDGKACAAVG
QNSLMALYDILFTQLDVTSAQLLVTDNDFRDEDFRKQLTETVKSLLSLKVIPVFNENDAVSTRKAPYEDSSGIFWDNDSLSALLALELKADLLVLLS
DVEGLYSGPPSDPHSKLIHTYIKEKHQNEITFGDKSRVGRGGMTAKVKAAVHAADAGIPVVITSGFAAENIINVLQGQRIGTLFHKDAHEWVQVKEV
DAREMAVAARECSRRLQAISSEERKQILLKIADDLEANEKIIRTENEADVAVAQQAGYENSLVARLALKPGKIASLANNVRIIANMEDPIGQVLKRT
ELSDGLILEKTSSPLGVLLIVFESRPDALVQIASLAIRSGNGLLLKGGKEAKRSNAILHKVITEAIPDTVGGKLIGLVTSREEIPELLKLDDVIDLV
IPRGSNKLVSQIKSSTKIPVLGHADGVCHVYVDKSANVEMARGIVLDAKLDYPAACNAMETLLIHKDLIEKGWLNDIVVDLRTEGVKLYGGPRASSL
LNIPQAHSFHHEYSSLACTVEIVDDVYAAIEHINLYGSAHTDSIIAEDKEVANVFLRQVDSAAVFHNASTRFSDGARFGLGAEVGISTSRIHARGPV
GVEGLLTTRWILKGSGQVVDGDKGIVYTHKDLAA
>GmALDH18B5
MENTDPCRHFLKDVKRIIIKVGTAVVTRQDGRLAVGKLGALCEQIKELNSLGYEIILVSSGAVGLGRQRLRYRKLINSSFADLQKPQVELDGKACAA
VGQNSLMALYDVLFSQLDVTSAQLLVTDNDFRDKDFRMQLSETMKSLLALKVIPIFNENDAVSTRKAPYEDSSGIFWDNDSLSALLALELKADLLIL
LSDVEGLYSGPPSDPRSKLIHTYIKEKHQSEITFGDKSRVGRGGMTAKVKASIHAAEAGIPVIITSGYAAENIIKVLQGQRIGTLFHKDAHKWAPVK
EVDAREMAVAARDCSRRLQALSSEERKQILLKIADALEAHQNEIRIENEADVADAKEAGYEKSLVARLVLKNEKLASLANNIRIIANMEDPIGRVLK
RTELAEGLILEKTSSSLGVLLIVFESRPDALVQIASLAIRSGNGLLLKGGKEAKRSNAILHKVITEAIPDIVGSKLIGLVTSRAEIPELLKLDDVID
LVIPRGSNKLVTQIKSSTKIPVLGHADGICHVYVDKSADLEMARRIVLDAKIDYPAGCNAMETLLVHKDLVEKGWLNSIIIDLRTEGVTLYGGPKAS
PLLNIPMARMLHHEYNSLACTVEIVDDVYAAIDHINLYGSAHTDSVVAEDHEVANVFLRQVDSAAVFHNASTRFSDGARFGLGAEVGISTSRIHARG
PVGVDGLLTTRWILKGSGQIVDGDKAVNYTHRDLSI
>GmALDH22A1
MAFWWPLLVLAFAYGICRFLLMLIPPKVPSIDVDTSDVLDDGNQAQENSFIYVPPRGTSQQSGKIVQCYEPATMKYLGYVPALTHEEVKDRVSKVRK
AQKMWAKSSFKQRRLFLRILLKYIIKHQALICEISSRDTGKTMVDASLGEIMTTCEKINWLLSEGEQWLKPEYRSSGRSMLHKRAKVEFHPLGVIGA
IVSWNYPFHNIFNPMLAAIFSGNGIVIKISEHASWSGCFYFRIIQSALAAIGAPEDLVEVITGFAETGEALVSSVDKVIFVGSPGVGKMIMNNASNT
LIPVTLELGGKDAFIVCEDVDLDHVAQIAVRAVLQSSGQNCAGAERFYVHREIYSSFVSKVTKIVKSVTAGPPLVGKYDMGALCMHEHSEKLEGLVN
DALDKGAEIVARGNLGHIGEDAVDQYFPPTVIVNVNHTMRLMQEEAFGPIMPIMKFSSDEEVVRLANDSKYGLGCAVFSGNQSRAREIASQIHAGVA
AVNDFASTYMCQSLPFGGVKHSGFGRFGGVEGLRACCLVKAVVEDRWWPFVKTKIPKPIQYPVAENGFEFQESLVEALYGLGIWDRLRALVNVLKML
TEQNPGGSSNKRRND
>GmALDH22A2
MAFWWPLLVLALAFAICKFLLILIPPKVPSIDVDASDVLDDGSQAQENSFIYVPPRGTAQQSSGKVQCYEPATMKYLGYVPALTPDEAREQVEKVRK
AQKMWAKTSFKKRRQFLRILLKYIIKHQALICEISSRDTGKTMVDASLGEIMTTCEKINWLLSEGEQCLKPEYRSSGRAMLHKRAKVEFHPLGVIGA
IVSWNYPFHNIFNPMLAAVFSGNGVVIKISEHASWSGCFYFRIIQSALAAIGAPEDLVEVITGFAETGEALVSSADKVIFVGSPGVGKMIMSNAAET
LIPVTLELGGKDAFIVCEDVDVDLVAQIAVRAALQSSGQNCAGAERFYVHRKIYASFGPPLAGKYDMGALCMHAHSEMLEALINDALDKGAEIIARG
SFGPIGEDAVDQYFPPTVIVNVNHSMRLMQEEAFGPIMPIMKFSSDEEVVRLANDSKYGLGCNVFSGSQSRAREIASQIHCGLAAVNDFASTYMCQS
LPFGGVKNSGFGRFGGVEGLRACCLVKSVVEDRWWPFIKTVIPKPIQYPVAENGFEFQESLVEALYGLSVWDRLQALVNVLKMLTEQNSTSGSRKKK
ND
>GmALDH22A3
MAFWWPLLVLALAFAICKFLLILIPPKVPSIDVDASDVLDDGSLTQENSFIYVPPRGTAQQSSGKVQCYEPATMKYLGYVPALTPDEVKEQVEKVRK
AQKMWAKTSFKKRRHFLRILLKYIIKHQALICEISSRDTGKTMVDASLGEIMTTCEKINWLLSEGEQCLKPEYRSSGRAMLHKRSKVEFLPLGVIGA
IVSWNYPFHNIFNPMLAAVFSGNGIVIKISEHASWSGCFYFRIIQSALAAIGAPEELVEVITGFAETGEALVASADKVIFVGSPGVGKMIMSNAAET
LIPVTLELGGKDVFIVCEDADVDHVAQVAVRAALQSSGQNCAGAERFYVHRNIYASFVSKVTKIIKSVTAGPPLAGKYDMGALCMHAHSEKLEALIN
DALDKGAEIIARGSFGHIGEDAVDQYFPPTVIVNVNHSMRLMQEEAFGPIMPIMKFSSDEEVVRLANDSKYGLGCNVFSGSQSRAREIASQIHCGLA
AVNDFAATYMCQSLPFGGVKNSGFGRFGGVEGLRACCLVKSVVEDRWWPFIKTVIPKPIQYPVAENGFEFQESLVEALYGLSVWDRLQALVNVLKML
TEQNSTSGSRKKKND
>GmALDH22A4
MAFWWPLLVLAFAYGICRFLLMLIPPKVPSIDVDTSDVLDDGNQAQENSFIYVPPRGTSQQSGKIVQCYEPATMKYLGYVPALTRDEVKDRVAKVRK
AQKMWAKSSFKQRRLFLRILLKYIIKHQALICEISSRDTGKTMVDASLGEIMTTCEKINWLLSEGEQWLKPEYRSSGRSMLHKRAKVEFHPLGVIGA
IVSWNYPFHNIFNPMLAAIFSGNGIVIKISEHASWSGCFYFRIIQSALAAIGAPEDLVEVITGFAETGEALVSSVDKVIFVGSPGVGKMIMNNAANT
LTPVTLELGGKDAFIVCEDVDLDHVAQIAVRAVLQSSGQNCAGAERFYVHREIYSSFVSLVTKIVKSVTAGPPLVGKYDMGALCMHEHSEKLEGLVN
DALDKGAEIVARGSFGHIGEDAVDQYFPPTVIVNVNHTMRLMQEEAFGPIMPIMKFSSDEEVVRLANESKYGLGCAVFSGNQSRAREIASQIHAGVA
AVNDFASTYMCQSLPFGGVKHSGFGRFGGVEGLRACCLVKAVAEDRWWPFVKTKIPKPIQYPVAENGFEFQESLVEALYGIGIWDRLRALVNVLKML
TEQHPGGGGKRRND
>BrALDH2C1
MENGKCNGGATAKLPEIKFTKLFINGQFLDAASGKTFETIDPRNGEVIAKIAAGDKEDVDLAVNAARHAFDHGPWPRMTGFERARIINKYTDLIQQN
IEELAALDAVDGGKLFQVGKMNDIPAAAGHFRYYAGAADKIHGETLRMTRPSLFGYTLKEPIGVVGHIIPWNFPSIMFAMKVAPALAAGCTMVVKPA
EQTPLSALFYAHLSKEAGFPDGVINLVTGFGSTAGAAIASHMDIDKVSFTGSTDVGRKIMQAAATSNLKKVSLELGGKSPLLIFDDADVNKAAELAL
LGCFYNKGEICVASSRVFVQEGIYDKVVAKMVEKVKDWPVGDPFDSTSRQGPQVDKKQYEKVLSYIEHGKNEGATLLTGGNAIGDKGYYIEPTIFAD
VTDDMKIYKEEIFGPVMSLMKFKTMEEGIKCANNTKYGLAAGIVSQNVDVINTVSRSIKAGVIWVNCYFAFDLDSPYGGYKMSGNCRESGMDALDSY
LQVKSIAMPLHNSPWM
>BrALDH2B1

MAARSRVSSLLSRSFSASSPFLSRSQGRNLNNGSRIVRRFGTSSAAEEVISPSVQVSYTKLLIDGNFVDAASGKTFPTLDPRTGEVIAHVAEGDAED
INRAVKAARKAFDEGPWPKMTAYERSRVMLRFADLVEKHSEELAALESWDNGKTYEQALTAEIPMVARLFRYYAGWADKIHGLTVPADGNYHVQTLH
EPIGVAGQIIPWNFPLLMFAWKVGPALACGNTIVLKTAEQTPLTAFYVGKLFLEAGLPPGVLNIVSGFGATAGASLASHMDVDKLAFTGSTDTGKVI
LGLAANSNLKPVTLELGGKSPFIVFEDADIDKAVELAHFALFFNQGQCCCAGSRTYVHEKVYDEFVEKAKARALKRVVGDPFKKGIEQGPQIDSKQF
EKVMKYIRSGVESNATLECGGGQVGDRGYFIQPTVFSNVKDDMLIAQDEIFGPVQSILKFSDVDEVIKRANDTRYGLAAGVFTKSLDTANRVSRALK
AGTVWVNCFDVFDAAIPFGGYKMSGNGREKGIYSLNNYLQVKAVVTPLNNPAWI
>BrALDH2B2
MAARRVSSLLSRSFSTSSPFPFRSQGRNCYNGSRVVRRFGTSSAAEKIISPSVQVSCTQLLIDGNFVDATSGKTFQTLDPRTGEVIADVAEGDAEDI
DRAVKAARKAFDKGPWPRMTAYERSRIMLRFADLVEKHSEELAALETWDNGKTYQQAKTAEIPMLARLFRYYAGWADKIHGLTVPADGNYHVQTLHE
PIGVAGQIIPWNFPLLMFAWKVGPALACGNTIVLKTAEQTPLTAFYAGKLFLEAGLPPGVLNIVSGFGPTAGASLASHMDVDKLAFTGSTDTGKVIL
GLAANSNLKPVTLELGGKSPFIVFEDANINKAVELAHFALFFNQGQCCCAGSRTYVHEKVYDEFVEKAKARALKRVVGDPFKKGIEQGPQIDSKQFE
KVMRYIRSGVESNATLECGGDQIGNKGYFIQPTVFSNVKDDMLIAQDEIFGPVQSILKFRDVDEVIRRANETRYGLAAGSLDTANRVSRALKAGTVW
VNCFDVFDAAIPFGGYKMSGNGREKGIYSLNNYLQIKAVVTALNNPAWI
>BrALDH2B3
MASRRVSSLLSRSLISSSSLFSLRGKDPLLNRGARRYSNLAASLEDTITPPVKVEHTQLLINGKFVDSASGKTFPTLDPRTGEVIAQVAEGDVEDVN
RAVVAARKAFDQGPWPRMTAYERSKILFRFADLIEKHNDEIAALETWDNGKPYEQSANIEVPMLARVFRYYAGWADKIHGMTVPGDGSHHVQTLHEP
IGVAGQIIPWNFPLLMLSWKLGPALACGNTVVLKTAEQTPLSALLVGRLLHEAGLPEGVVNIVSGFGPTAGAAIASHMDIDKVAFTGSTDVGKIILE
LASKSNLKAVTLELGGKSPFIVCEDADVDQAVEMAHFALFFNQGQCCCAGSRPFVQERVYDEFVEKAKARAINRAVGDPFKSGIEQGPQVDSEQFEK
ILKYIRHGVDSGATLQAGGDRHGSKGYYIQPTVFSDVKDDMLIAKDEIFGPVQTILKFKNLDEVIARANNSRYGLAAGVFTQNLDTANRLMRALRVG
SVWINCFDVFDATIPFGGYKMSGIGREKGIYSLNNYLQVKAVVTSIKNPAWL
>BrALDH2B4
MASRRVSSMLSRSFMSSPSLFALRGKHHNMNRGVYGYSNVAAGEDTITPPVKVEHTQLLIGGKFVDAASGKTFPTLDPRTGEVIAQVAEGDVEDVNR
AVSAARKAFDEGPWPRMTAYERSKILLRFADLVDKHNDEIAAIETWDNGKPFEQSSKIEVPMLARVFRYYAGWADKIHGMTVPGDGSHHVQTLHEPI
GVAGQIIPWNFPLLMLSWKLGPALACGNTVVLKTAEQTPLSALLVGRLLHEAGLPEGVVNIVSGFGPTAGAAIASHMDIDKVAFTGSTDVGKIILQL
ASKSNLKAVTLELGGKSPFIVCEDADVDQAVELAHFALFFNQGQCCCAGSRTFVHERVYDEFVEKAKARAIKRAVGDPFKSGIEQGPQVDSEQFKKI
LKFIKHGVESGATLQAGGDRFGSKGYYIQPTVFSDVKDDMLIATDEIFGPVQTILKFKNLDEVIARANNSRYGLAAGVFTQNLDTANRLMRALRVGS
VWINCFDVFDATIPFGGYKMSGIGREKGIYSLNNYLQVKAVVTAIKNPAWL
>BrALDH3H1
MFKQPLTDLMLSIDAVIGAISAGNTVVLKASELAPASSSLLAKLLEQYLPCAVRVVEGAVTETTLLLEQKWNKIFYTCSSRIGRIIMKAAVKHLTPV
SLELGGKSLIVIDSDTNLDVKKYSPFPSGITITVRRIISGKWGCNNGQVCISPDYILTTKEYAPKVIDALKQELEAFYGNKSRESKDMSRIVNLNQF
DRLSKMLEEKEVSDKIIYGGQKNRDNLNISPTILLDVPLDSLIMSEEIFGPLLPILMMYGQDGALVVHKVRHELRHQTRIQGLHESVKEH
>BrALDH3H2
MAKVFEAADASNLMTELRMSFDAGVTRSYEWRVSQLKKLQVICDNHEPEIVSALHDDLGTPELESSVYEVALLRNSIKLALKQLKNWMAPDKAKTSL
TTFPASAEIVSEPLGVVLVISAWNYPFLLSIDPVIGAISAGNAVVLKPSELAPASSSLLAKLLEQYLDPSAVRVIEGAVTETTLLLEQKWDKIFYTG
SSKIGRIIMMAAAKHLTPVVLELGGKSPVVIDSDTNLKITVKRIIAGKWGCNNGQACISPDYILTTKEYAPKVIDAMKQELEAFYGKNPMESKDMSR
IVNSNHFDRLSKILEEKEVSDKIVYGGQKNRDNLKIAPTIFLDVPLDSLIMSEEIFGPLLPILTLNNLEECFDVIRSRPKPLAAYLFTQNQKLKERF
AMTVSAGGIVVNDIAVHLSLPTLPFGGVGESGMGSYHGKFSFDAFSHKKAVLYKSFIGDAAIRYPPYSRGKLRLLKALVNSNLVEVFKVLLGLS
>BrALDH3H3
MVKVFQAADATDLVTELRRSFDDGVTRGYEWRVTQLKKLLLICDNHEPEIVSALHDDLGKPELESSVYEVALLRNSINLAVKQLKDWMAPDKAKTSL
TTFPASAEIVYEPLGVVLVISAWNYPFLLSIDPVIGAISAGNAVVLKPSELAPASSSLLAKLLEQYLDSSAVRVVEGAVTETTLLLEQKWDKIFYTG
SSRIGRIIMMAAAKHLTPVVLELGGKSPVVIDSDTNLKITAKRIIAGKWGCNNGQACISPDYILTTKEFSPKVIDALKQELEAFYGKNPMESKDMSR
IVNSNHFDRLSKMLEEKEVSDKIVYGGQKNRDKLKIAPTILVDVPLDSQIMSEEIFGPLLPIITLNNLEECFDVIRSRPKPLAAYLFTQNQKLKERF
ALTVSAGGIVVNDIAVHLAVPTLPFGGVGESGMGSYHGKFSFDAFSHKKAVLYKSFIGDAAIRYPPYSTGKLRLLKALVNSNILEIFRVILGLS
>BrALDH3I1
MTKLLKINHHHTIPFAGGLYRTTTRSNVASLALATSPFQFSSGYCSKTCIPSRLKLVSSTCYATLSAVVKPQESAFDGKEAALLVDELRTNFNTGRT
RSYEWRISQLQNIAKMIDEKEKCITEALYQDLSKPELEAFLAELSNTKSSCMLAIKELKNWMAPETVKTSVTTFPSSAQIVSEPLGVVLVISAWNFP
FLLSVEPVIGAISAGNAVVLKPSEIAPATSSLLAKLFSEYLDETAIRVVEGGVPETTALLDQKWDKIFFTGGARVGRIVMAAAAKNLTPVVLELGGK
CPALVDSDVNLQVAARRIITGKWACNNGQACIGVDYVITTKDFAPKLIDALKTELKTFFGENPLKSKDVSRIVNSFHFKRLESMMKENGVANKIVHG
GQTMEDKLKISPTILVDVPEESSMMQEEIFGPLLPVITVSKIEDGFQVIRSKPKPLAAYLFTDNKVLQNRFVENVSAGGMGINETVLHVTLKDLPFG
GVGESGIGAYHGKFSYETFSHKKGVLYRSFDGDSDLRYPPYTPEKKRVLKALLSSDIFGAILAFFGFSKDS
>BrALDH3F1
MEEVVDQSLREMRDTFASGRTRSVKWRKTQLEAIIEMVKDNEDKMCDVLFQDLGKHSTEAFRDELGFVMRSATTALNCLDKWVVPKKSNLPLLFYPS
TGKVISEPYGTVLVLSSWNFPISLSLDPLIGAISAGNTVLLKASELSPNASAFLAKTIPSYLDNKAIKVIEGGPDVATILLKHQWDKIFFTGSPRIG
KIIMAAAAEHLTPVTLELGGKCPTIVDHHSVSKDMKSVVKRISGGKWGSCSGQACISVDYVLVEKSFASSLIEMLKPMIRSFFGENPKESGCLSRIV
NKKHFQRLARLLNDPGVQASIVYGGSMDEEKLYIEPTILLDPPLDSEIMNEEIFGPILPIITLRDIQESIGFIKSKPKPLAIYAFTKDENLKTRILS
ETSSGSVTFNDVMIQYMCDALPFGGVGESGIGRYHGKYSFECFSHEKAIMEGSLAMDLEARYPPWNSFKLTFLRLAFREAYFKLVLFMLGLKK
>BrALDH3F2
MEAMKETVDQSLREMRDTFASGRTRSVKWRKTQLEAIIEMVKDNEDKMCDVLFQDLGKHSIEAFRDELGFVMRSATTALNSLDKWVVPRKSNLPLLF
YPATGKVISEPYGTVLVLSSWNFPISLSLDPMIGAISAGNTVLLKASELSPNASALLAKTIPSYLDNKAIKVIEGGPDVATILLQHQWDKIFFTGSP
RIGKIIMAAAAENLTPVTLELGGKCPTIIDHHSVSKDMKSVVKRISGGKWGSCSGQACISVDYVLVEQSFASSLIEMFKPVIKSFFGENPKESGCVA
RIVTKKHFQRLSRLLNDPRVQASIVYGGSMDEEKLYVEPTILLNPPLDSEIMNEEIFGPVLPIITLRDIQESIGFIKSKPKPLAIYAFTKDEKLKTR
ILSETSSGSVTFNDVMIQYMCDALPFGGVGESGMGRYHGKYSFECFSHEKAIMEGSLAMDLEARYPPWNNFKLTFIRLAFREAYFKLVLLMLGLKR
>BrALDH3F3
MEAVKETVDQSLREMRDTFASGRTRSVKWRKAQLGAIIEMVKDNEEKMSDVLFQDLGKHSTEAFRDELGFVMRSATTALNCLDKWVVPRNSNLPLLF
YPATGKVISEPYGTVLVLSSWNFPISLSLDPMIGAIAAGNTVLLKASELSPNASALLAKLIPSYLDTKAIKVIEGGPDIATILLQHQWDKIFFTGSP
KIGKIIMAAAAEHLTPVTLELGGKCPTIIDHHSVSKDMKAYYVLVEQSFASTLIDMFKPVIRSFFGENPKESGCLAKIVTKKHFQRLSRLLNDPRVK

ASIVYGGSMDEEKLYVEPTILLDPPLDSEIVNEEIFGPILPIITLRDIQESIGFIKSKPKPLAIYAFTKDENLKTRILSETSSGSVTFNDLMIQYMC
DALPFGGVGQSGMGRYHGKYSFECFSHEKAIMEGSLAMDPEARYPPWNNFKLNFIRLAFREAYFKLVLLMLGLTKGVRK
>BrALDH5F1
MVLGAAARVAIVGCRRLVCSSSHASPLLVSSQCRQMSMDAQSVSEKLRSSGLLRTQGLIGGKWIDSYDKTTIKVNNPATGEIVADVACMGVKETNDA
IASSYEAFQSWSRRTAGERSRVLRRWFDLLVAHKEELGQLITLEQGKPLKEAIGEVAYGASFIEYYAEEAKRVYGDIIPPNASDRRLLVLKQPVGVV
GAITPWNFPLAMITRKVGPALASGCTVVVKPSELTPLTALAAAELALQAGVPPGALNVVMGNAPEIGDALLASPQVRKITFTGSTAVGKKLMAAAAP
TVKKVSLELGGNAPSIIFDDADLDVAVKGTLAAKFRNSGQTCVCANRVLVQDGIYDKFAEAFSEAVQKLEVGDGFKEGTTQGPLINDAAIQKVESFV
QDAVSKGAKILLGGKKHSLGMTFYEPTVIRDVTSNMIMSKEEIFGPVAPLIRFKTEEDAIRIANDTIAGLAAYIFTNSVQRSWRVSEALEYGLVGVN
EGIISTEVAPFGGVKQSGLGREGSKYGMDEYHEIKYICMGDMNRQ
>BrALDH6B1
MLLRTSLQQGKTNLRPRFLRCLLSTMSSTQPPRVPNLIGGSFVDSQASSHIDVINPATQEVVSQVPLTTNEEFKAAVSSAKKAFPSWRNTPITTRQR
VMLKFQELIRKNMDKLALSITTEQGKTLKDAHGDIFRGLEVVEHACGMATLQMGEYVPNVSNGVDTYSLREPLGVCAGICPFNFPAMIPLWMFPIAV
TCGNTFVLKPSEKDPGASVMLAELAMEAGLPDGVLNIVHGTNDTVNAICDDDDIRAVSFVGSNTAGMHIYARAAAKGKRIQSNMGAKNHGVVLPDAN
VDATLNALLAAGFGAAGQRCMALSTVVFVGNSKSWEDKLVERAKALKVSCGTEPDADLGPVISIQAKERICRLIQSGVDDGAKLLLDGRNIVVPGYE
KGNFIGPTILSGVTPDMECYKEEIFGPVLVCMEASSFDEAIDILNRNKYGNGAAIFTASGAAARKFQMEIEAGQIGINVPIPVPLPFFSFTGNKASF
AGDLNFYGKAGVDFFTQIKTVTQQWKDIPTSVSLAMPTSQKQ
>BrALDH7B1
MVQQVSGRSGKTLLELSGNNAIIVMDDADIHLLHVSVYDKVLEQLLTSYQVKIGDPLQKGTLLGPLHTPESRKNFKKGIEVIKSQASTSSPKSSLNI
FNFQLTLLHSQGGKVLTGGKAIEGEGNFVEPTILEISSDAVLVKEELFAPVLYALSHLKKQVAINNSIPQGLSSSIFTRKPKNIFKWIGPMGSDCGI
VNVNIPTNGAEIGGDFGGEKATGGGRETGSDSWKQ
>BrALDH7B2
MGSASKEYEFLSEIGLSSSHNLGNYVGGKWLGNGPLVSTLNPANNQPIAQVVEASLEDYEIGLKACEEAAKTWMQVPAPKRGDIVRQIGDALRSKLD
YLGRLLSLEMGKILAEGIGEVQEVIDMCDFAVGLSRQLNGSVIPSERPNHMMLEMWNPLGIVGVITAFNFPCAVLGWNACIALVCGNCVVWKGAPTT
PLITIAMTKLVAEVLEKNHLPGAIFTAMCGGAEIGEAIAKDTRIPLVSFTGSSKVGLTVQQTVSARSGKTLLELSGNNAIIVMDDADIQLAARSVLF
AAVGTAGQRCTTCRRLLLHESVYDKVLEQLLTSYKQVKIGDPLEKGTLLGPLHTPESKKNFEKGIEGGKVLTGGKAVEGEGNFVEPTIIEISSDAAV
VKEELFAPVLYALKFKTFEEAVAINNSVPQGLSSSIFTRSPENIFKWIGPMGSDCGIVNVNIPTNGAEIGGAFGGEKATGGGREAGSDSWKQYMRRS
TCTINYGNELPLAQGINFG
>BrALDH10A1
MAIRVPRRQLFIGGQWTEPIRRQTLPVVNPATEDIIGYIPAATSEDVELAVEAARKALTRNQGKDWSKASGAVRARYLRAIAAKVTERKSELANLEA
IDCGKPLDEAAWDMDDVAGCFEYYADLAQGLDAKQKAPLSLPLDTFKGYVLKEPIGVVGLITPWNYPLLMAVWKVAPALAAGCTAILKPSELASVTC
LELADICREVGLPPGVLNILTGLGTEAGAPLASHPHVDKIVFTGSTATGSNIMTSAAKLVKPVSLELGGKSPIIVFDDVEIDKAVEWTMFGCFWTNG
QICSATSRLLVHEKIADEFLDKLVKWTKNIKISDPFEEGCRLGPVVSKGQYERVVKFVSNARKEGATVLCGGARPGHLKKGYFVEPAIISNVTTSME
IWRDEVFGPVLCVKTFSTEDEAIQLANDSQYGLAGAVLSNDLERCDRVSKAFEAGIVWVNCSQPCFCQAPWGGTKRSGFGRELGEWGLENYLSVKQV
TQYISNEPWGWYKPPSKL
>BrALDH10A2
MAIPMPTRQLFIDGEWRAPILKNRIPIVNPATQDVIGDIPAATKEDVEVAVNAARRAFSRNKGKDWAKAPGALRAKYLRAIAAKVTERKSHLATLES
LDSGKPLDETVWDMEDVAGCFEFYADLAEGLDAKQKAPVSLPMETFKSYVLKQPIGVVGLITPWNYPLLMAVWKVAPSLAAGCTAVLKPSELASVTC
LELADICREVGLPPGVLNVLTGYGSEAGAPLASHPSVDKIAFTGSFATGSKVMTAAAQLVKPVSMELGGKSPLIVFDDVDLDKAAEWALFGCFWTNG
QICSATSRLLVHENIASEFIEKLVKWSKNIKVSDPLEEGCRLGPVVSEGQYEKILKFISTAKSEGATILHGGSRPEHLKKGFFIEPTIITDVTTSMQ
IWREEVFGPVLCVKTFSSEDEAIELANDSHYGLGAAVISNDAERCDRVSQDFEAGIVWINCSQPCFTQAPWGGVKRSGFGRELGEWGLDNYLSVKQV
TLYTSNDPWGWYKPPC
>BrALDH11A1
MAGTGIFTDILDGDVYKYYSDGEWKTSSSGKSVAIINPATRKTQYKVQACTQEEVNKVMEMAKSAQKSWAKTPLWKRAELLHKAAAILKDNKAPIAE
SLVKEIAKPAKDSVTEVVRSGDLISYCAEEGVRILGEGKFLLSDSFPGNERTKYCLTSKIPLGVVLAIPPFNYPVNLAVSKIAPALIAGNSLVLKPP
TQGAVSCLHMVHCFHLAGFPKGLISCITGKGSEIGDFLTMHPAVNCISFTGGDTGISISKKAGMIPLQMELGGKDACIVLEDADLDLVASNIIKGGF
SYSGQRCTAVKVVLVIKSVADELVEKVKAKVAKLTVGPPEENCDITAVVSESSANFIEGLVMDAKDKGATFCQEYKREGNLIWPLLLDNVRPDMRIA
WEEPFGPVLPVLRINSVEEGINHCNASNFGLQGCVFTKDINKAILISDAMETGTVQINSAPARGPDHFPFQGLKDSGIGSQGVTNSINLMTKLKTTV
INLPTPSYSMG
>BrALDH11A2
MAGTGIFTEILDGEVYKYYSDGEWRTSSSGKSVAIVNPATRKTQYKVQACTQEEVNKVMEMAKSAQKSWAKTPLWKRAELLHKAAAILKDNKAPIAE
SLVKEIAKPAKDSVTEVVRSGDLISYCAEEGVRILGEGKFLLSDSFPGNERTKYCLTSKIPLGVVLAIPPFNYPVNLAVSKIAPALIAGNSLVLKPP
TQGAVSCLHMVHCFHLAGFPKGLISCITGKGSEIGDFLTMHPAVNCISFTGGDTGISISKKAGMIPLQMELGGKDACIVLEDADLDLVASNIIKGGF
SYSGQRCTAVKVVLVMESVADELVEKVKAKVAKLTVGPPEENCDITAVVSESSANFIEGLVMDAKEKGATFCQEYKREGNLIWPLLLDNVRPDMRIA
WEEPFGPVLPVLRISSVEEGINHCNASNFGLQGCVFTKDINKAMLISDAMETGTVQINSAPARGPDHFPFQGLKDSGIGSQGVTNSINLMTKVKTTV
INLPTPSYSMG
>BrALDH12A1
MYRVLASRGLRAKSLCDNKASSFLASFTSSRLNHSIPFATVDAEEISGARPAEVQSFVQGKWIGSSNYNTLLDPLNGEPFIKVAEVEESGVQPFIES
LAQCPKHGLHNPFKSPERYLLYGDISTKAAHMLALPKVSDFFTRLIQRVAPKSYQQAAGEVFVTRKFLENFCGDQVRFLARSFAVPGNHLGQQSHGY
RWPYGPVTIVTPFNFPLEIPLLQLMGALYMGNKPLLKVDSKVSIVMEQMMRLLHYCGLPVEDVDFINSDGKTMNKILLEANPRMTLFTGSSRVAEKL
ALDLKGRIRLEDAGFDWKVLGPDVQEVDYVAWVCDQDAYACSGQKCSAQSMLFVHENWSKTPLLSKLKDLAGRRKLEDLTIGPVLTFTTEAMVEHME
NLLQIPGSKLLFGGKPLKNHSIPSIYGALEPTAVYVPIEEILKDSKTYELVTKEIFGPFQIVTEYKKDQLPLVLDALERMHAHLTAAEVIGNSVNGT
TYAGLRGRTTGAPQNHWFGPAGDPRGAGIGTPEAIKLVWSCHREVIYDYGPIPQGWELPPST
>BrALDH12A2
MSHDEKRLISSLLDLNNVQIFDEVISGAHPAEVQSFVQGKWIGSSNYNTLDPLNGEPFIKVSEVDESGVQPFVESLSQCPKHGLHIPFKSPERYFKS
PERYLLYCDISTKAAHMLALPKVSDFFTRLIQRVAPKSYQQAAGEVFVTRKFLDNFCGDQVMQCSKKSV
>BrALDH22A1

MAFWWPLIVLAFAYAICRFLLMLIPPNVPSIDVDASDVLAHGKETEENSFIYIPPRGRSQQSDKKVQCYEPATMKYLGYFPALSTSEVKERVALSRK
AQKTWAQSSFKVRRQFLRILLKYIIEHQELICEVSSRDTGKTMVDASLGEIMTTCEKITWLLSEGERWLKPEYRSSGRAMLHKVSRVEFHPLGVIGA
IVPWNYPFHNIFNPMLAAVFSGNGIVIKVSEHASWSGCFYFRIIQAALAAVGAPENLVDVITGFAETGEALVSSVDKMIFVGSTAVGKMIMRNAAET
LTPVTLELGGKDAFIICEDADVSHVAQVAVRGTLQSSGQNCAGAERFYVHKDIYTAFITQVTKIVKSVSAGPPLTGRYDMGAICLQEHSEHLQSLVN
DALDKGAEIAVRGSFGHLGEDAVDQYFPPTVLINVNHTMKIMKEEAFGPIMPIMQFSTDEEVIKLANDSRYALGCAVFSGSQRRAKQIASQIQCGVA
AINDFASNYMCQSLPFGGVKDSGFGRFAGIEGLRACCLVKSVVEDRFWPLIKTKIPKPIQAKNAFEFQEALVETLYGLNIWDRLRSLIDVLKFLTDQ
SSHVSRTRKSH
>VvALDH2B4
MAARRISSLLSRSLSVSSAFSLSLGKNFNRGKSIHRFSTAAAAAVEELITPTVQINYTQLLINGQFVDAASGKTFPTFDPRTGEVIANVAEGDAEDI
NRAVSAARKAFDEGPWPRMSPYERSRILLRFADLAEKHNDELAALETWNNGKPYEQAAKAELPLFVRLFRYYAGWADKIHGLTVQADGPHHVQILHE
PIGVAGQIIPWNFPLMMFAWKVGPALACGNTIVLKTAEQTPLTALFAAKLFHEAGLPPGILNIVSGYGPTAGAALASHMDVDKIAFTGSTDTGKIVQ
ELASKSNLKPVTLELGGKSPFIVCEDADIDQAVELAHFALFFNQGQCCCAGSRTFVHESVYDEFIEKAKARALSRTVGDPFKKGIEQGPQIDPEQFA
KVLRYIRSGIESNATLECGGGRIGSKGYFVQPTVFSNVQDDMLIAKDEIFGPVQSILKYKDLDEVIRRANSTRYGLAAGVFTKNINTANTLTRALRV
GTVWVNCFDVFDAAIPFGGYKMSGVGREKGIYSLNNYLQVKAVITPLKNPAWL
>VvALDH2B8
MTIPRISSLLSRSFTSSASSALLSSIGRNSSRRGGIFRYSTAAVVEEPINPSVNVNYTQLLINGQFVDAATGKTFETLDPRTGNVIASVAEGDAEDV
NRAVSAARKAFDEGPWPRMSPYERSKILLRFADLLEKHNDEIAALETWDNGKPFEQAAKAEVPLVIRLMRYYAGWADKIHGLTVPADGLHQVQTLHE
PIGVAGQIIPWNFPLLMYAWKIGPALACGNTIVLKTAEQTPLSALYASKLLHEAGLPPGVLNVVSGYGPTAGAALASHMDVDKLAFTGSTATGKIVL
QLAARSNLKPVTLELGGKSPFIVCEDANVDEAVELAHFALFFNQGQCCCAGSRTFVHESIYDEFVEKAKARALRRTVGDPFKAGIEQGPQIDSDQFE
KILRYIRSGVENGATLETGGERFGKEGFFIKPTVFSNVQDGMLIAQDEIFGPVQSILKFKDLGEVIRRANATSYGLAAGVFTQNLDTANTLTRALKV
GTVWINCFDVFDAAIPFGGYKMSGHGREKGIYSLQNYLQVKAVITPLKNPAWL
>VvALDH2B9
MAAPRIFSLLSASSTAASLRSFGRYSRWGRGSSRFSTAVATEEEPITPPVQIDYTQLLINGRFVDAASGKTFPTLDPRTGDVIAHVAEGEAEDINRA
VSAARKAFDEGPWPKMTPYERSCILFRFADLLEKHCSEIAALESWDNGKPYEQAANVEIPMVIRVFRYYAGWADKIHGLTVPADGLHHVQTLHEPIG
VAGQIIPWNFPLLLYGWKVGPALACGNTIVLKTAEQTPLSALYASKLLYEAGLPPGVLNVVSGFGPTAGAALSSHMDVDKLAFTGSTGTGKIVLGLA
AKSNLKPVTLELGGKSPFIVCEDADVDKAVELSHTALFYNQGQSCCSGSRTFVHESIYDEFIEKAKARALKRVVGDPFKKGVEQGPQIDSQQFNKIL
GYIKSGIEAGATLEAGGEKFSSKGYYIQPTVFSNVHDNMLIAKEEIFGPVQSILKFKDLDEVIRRANATHYGLAAGIFTQNLDTANTLTRALRVGTV
WINCFFVFDAAIPFGGRKMSGHGREKGIYGLSNYMQVKAVVTPLKNPAWL
>VvALDH3F1
MTGSGVNGMIGMEGGRVEESIGELRRTFRSGETRSAAWRKAQLKALLQLLRDNENKIFEALKQDLGKHPVESYRDELGVVEKSVKYSLSHVDEWMAP
KKSSLPLIFFPGKGQVLPEPLGLVLIFSSWNFPISLALDPVIGAISAGNSVVLKPSEQAPACSSFLANTIPLYLDSKAIKVIEGGAAISQQLLQQKW
DKIFFTGSPSVARIVMSAAVKHLTPVTIELGGKCPTIFDNLSSPSDTEVAVKRVVGGKWGPCNGQACIGVDYVLVEEKFASHLIEMLKKTIKKFYGE
NPKELKDISKIVNKHHFQRLHNLLKEPLVAASIVHGGLIDEEKLFIEPTILLDPPLDAEIMTEEIFGPLLPIITLKNIEESIEFINSRPKPLALYAF
TNDEAFKRRILSETSSGSVTFNDIIIQFVCDTLPFGGVGQSGFGRYHGKYSFDTFSHEKAVLRRSFFLELEPRFPPWNDFKLKFIRLVYSFDYLGLI
LLLLGLKR
>VvALDH3H1
MGVTPIKLNFCIFFVNIIIIIKYKILLVCLIFQREGKRLLIDLIVVFPRSSISKSCCGRKSSMAEDSETKKVFDAEAAASLMKELRGTYASGKTRSY
EWRVAQLKNLMKIVDDHEKDILDAIRADLSKPEQESYIAEISIIKSSCTRALGELNRWMKPEKVKTSITTFPSSAEIVSEPLGVVLIIGAWNFPLLL
ALDPAIGAIAAGNAVVLKPSELCPATSSLIAKLVGKYLDSSCIKVVEGAVAETSALLEQKWDKIFFTGSGRVGRIVMAAAAKHLTPVALELGGKCPV
VVDSDINLPVAIRRIAGGKWASNNGQACIAPDYIVTTKDFAPKLIDALKHELEAWYGKDPLESKDLAHIVNSNHFARLAKLLDDDKVSGKIIHGGQR
DKANLKFAPTILLDVPEDSLVMNEEIFGPLLPILTVDKLEDSFDMITSRGKPLAAYLFTNNKKLKEKFVKTVSAGGLVINDTVLHFAEKTLPFGGVG
ESGMGSYHGKFSYEAFSHRKSVLYRGFAGDASARYPPYSDRKLKLLKALLSGSVVGVILALIGWS
>VvALDH3H5
MRSLSPELFHVCSVDGGYTRSLRNQFPKQRNPPLPIPVLKKRKRLRFTHSSFICSATLAVMADEKKKVFDVESAASLVKELRGSFNAGKTKSYEWRI
AQLKGIEKMIDEREKDIIEALHEDLSKPELEAFVSEISMSKGACKLALKELGHWMKPEKAKTSMTTYPSSAEIVSEPLGVVLVISTWNYPLLLSIDP
VIGAIAAGNAVVLKPSEIAPATSTLLSKLLEEYLDNSSIRVVEGAVAETTALLEQKWDKIFYTGSPRVGRIVMAAAAKHLTPVTLELGGKCPVVVDS
NVNLQVAARRLIAGKWACNNGQACISPDYIITTKDFAPKLIDVLRHELEEFFGKNPIESEDMSRIVSVQHFKRLTRLLDEDEVSDKIIIGGQSDENQ
LKIAPTILVDVPEDTEIMKEEIFGPLLPILTVENLEESFDVINSKSKPLAAYLFSENKQLQKDFVNNISAGGMLINDTILHLTVSSLPFGGVGESGM
GSYHGKFSFDAFSHKKAVLYRGFTGESPARYPPYTPGKLKLLKTLTSGNIVSILLALLGFSKD
>VvALDH3J1
MAEIENLEPDLEELRESYRSGKTKEASWRKSQLKGLLTLLKEQEKDIFKALEQDLGKHYAESYRDEVGTLTKSVNLALSSLKDWMSSRKAKLPIATF
PSTAEVFPEPLGLVLIISSWNFPFGLSLEPVIGAIAAGNSVVLKPSELAPASSSLLAKTIPTYLDKKAVKVIEGGAAVGEHLLRCKWDKIFFTGNPR
VGRVVMTAAANHLTPVTLELGGKCPAIFDSFSSSWDKEMVIKRVLGGKFGACAGQACIAIDYILVQEGFAPTLLELLRNMTKKMFGENPRETKSMAR
IINKKHFLRLKNILDDPSVQSCIVHGGGVDEDNLFIEPTILMNPPLKASIMTDEIFGPLLPIITLKKIEDSIEFINSRPKALAIYVFTKNETLKRRI
ISETSSGSVTFNDAIIQYAADTIPFGGVGESGFGRYHGKFSFDTFTHEKAILRRSLLTEFWFRFPPWNDFKLALTKSAYRFDYFEFLLVLLGLKKNS
>VvALDH5F1
MGLLRSSCSALCHGPRTASVLRPSAVLTRQISMDTQNLVARLNSSGLLRSQCLIGGKWTEAYDGKTIPVHNPATGDVLVNVPCMGGQETNDAISVAY
EAFLSWSKLTAAERSKRLRKWYDLLIANKEELGQIITLEQGKPLKEAIGEVNYGAAFIEFSAEEAKRIYGDIIPSPLADRRLLVLKQPVGVVGAITP
WNFPLAMITRKVGPALACGCTVVIKPSELTPLTALAAAELALQAGIPPGAVNVVFGNAPEIGDALLASRQVRKITFTGSTAVGKKLMAGAAQTVKKV
SLELGGNAPCIIFDDADLEVAVKGALGTKFRNSGQTCVCANRILVQEGIYEKFAIAFSQAVQSMQVGEGFTEGVVQGPLINEAAVQKVESFVKDAVS
KGAKVLLGGKRHSLGMTFYEPTVIGDIKNDMLIARNEVFGPVAPLLRFKTEEEAIRIANDTNAGLAAYVFTENVQRMWRVTEALEYGLVGVNEGLVS
TEVAPFGGVKESGLGREGSKYGMDEFLEMKYVCFGNISSN
>VvALDH5F2
MEAQSVITRFQNSGVFRTQGLIGGKWTEAYDGKTIQVHNPATGEVIADVPCMGQPETNDAISSAYEMFNSWSKVTAIERSQCLWKWHDLLIAHKEEL
GQLITLEQGKPLNEAIIEVIIAAGYLEFFAEEAKHVYSDIIPSTVADCQLFVIKQPVGVVGAITPWNFPLAMLTSKVGPALACGCTVVLEPSELTPL
IAFAAAGLALEAGIPSGALNVVTGNAPDIEHALLASPKVRKITFTGLSAVEKKIMPGAGETLRKVSLEPGGNAPCIVFDDTDLGVAVKSILAVKFHN

SGQTCISANRILVQEGIYEKFATAFSKAVTSLQVGDGFCEGVTQGPLINEAAVQTVESLVQDAISKGAKLLLGGKRHNLGMTFYEPTVIGDVNNKML
ISRNKICGPIAALLRFKTEEEAICIANDTDEGLAAYIFTKNLQRSWRVSEVLEYGLVGVSEGLIPTVMAPVSGFKNTGLGQEGSKKGMLEYLELKYI
CLGNMNNI
>VvALDH5F3
MAKFLFDYYFQYNFQNRPTHLASWHYNSVMTYGSQLLFIVSEGFSRFGFHNLLLICHFYRQNFLIQISMDPQSAIAQVKRAGLFRTQGLIGGKWMDA
YDGKTFEVYNPATGEVLANVACMGKKEANDAIASAHAAFTSWSKLTTAERSKLMRKWYDLLIAHQTELGQLITLEQGKPVAEGYNEVLIGASCLEFF
LEEVKHCYGDIIPQTQADRRMFVLKQPVGVVGAITPWNLPLAMALRKVSPAMACGCTVVIKPSELTPLSALAVAELALQAGIPPGVFNMVMGFAPEI
GDAFLASPKVRAITFTGSTAVGKMLLAGAAQTVKKTSMELGGNAPSMIFDDADLEVTVKGLMIVKFFNCGQTCISPNRILVQEGIHDKFAAALTKAV
QTLRVGHGFDEGVTQGPLINQAALHKIEALVEDAVSQGAKVLVGGKRHSLGLTFYEPTVLVDVTSDMLISSTEIFGPVLALQRFKTEEEAIHLANDS
NAGLAGYIYTENLRRCWRVAEAIEFGIVGVNDGLIPAASAPFGGFKQSGLGREGSKYGLDDFLEIKYLCLGNMV
>VvALDH6B3
MLRFSLQRVRNFKSLRPELFALGNSRFSTATEPSSKQRNPPRVPNLIGGSFVDSQSSEFIDVINPATQQVVSQIPLTSNEEFKAAVSAAKQAFPAWR
NTPVTTRQRIMFKLQQLIRRDIDKLAMNITTEQGKTLKDAHGDVFRGLEVVEHACGMATLQMGEFVPNVSSGIDTYSIREPLGVCAGICPFNFPAMI
PLWMFPIAVTCGNTFVLKPSEKDPGASLMLAELAMEAGLPDGVLNIVHGTRNIVNAICDDEDIRAVSFVGSNTAGMHIYSRASAKGKRVQSNMGAKN
HAIVLPDASLDATLNALVAAGFGAAGQRCMALSTVVFVGDSKSWEDKLLERAKALKVNSGTEPDADLGPVISKEAKERICKLIQNGVDSGARLVLDG
RNIVVPGYEHGNFIGPTILSDVTADMECYKEEIFGPVLLCIQADSLDEAINIVNRNKYGNGASIFTSSGVDARKFQTEIEAGQVGINVPIPVPLPFF
SFTGSKASFAGDLNFYGKAGVQFYTQIKTVTQQWKGLASGSGVSLAMPTSQKSQ
>VvALDH6B5
MDIQDCTELNEIPQMLPPPPGSFIDREELIQHVGDFALSQGYVVTIKQSKKDKVVVLGCDRGGVYRNRRKLVDESSAEQVRKRKTGSRLTNCPFEVV
GKKEDGLWVLAIKNGEHNHDPIRDISEHPSSRRFTEREVLLIKDMTEAGLKPRQILKRLRQNNPELLSTPKHVYNVKAKLRQGNLTVRNFKSLRVKS
SVENSHISTANEPSWRQRNPPRVPNLIGGRFVDSQSFASIDVTNPATQKVVSQVPLTTNEEFRAAIFAAKRAFPSWRDTPVTTRQRIMFKFQELIRR
DIDKIAMNITTEHGKTLKDAYTDVHRGLEVVEHACGMATLQMGEFVSNVSNGIDTYSIREPLGVCAGICPFDFPAMIPLWMFPIAVTCGNTFILKPS
EKDPGATIILAELAMEAGLPNGVLNIVHGTVDIINAICDDDDIKAISFVGSNTDGMYIYARASAKGKRVQSNIGAKNHAIVMPDASKDATLNALVSA
GFGAAGQRCMVLSTVVFVGGSKSWEDKLVECAKALKVNAGIEPDADLGPVISKQVKERICRLIQAGVDSGARLVLDGRNIEACLTTYLSMIILLDFF
WSNQSFSYIP
>VvALDH6B7
MTDAGIKPRQVLKALKKNNPELQSTPRHLYNLKAKIRQGNISEKSFKSWRPNRSVPVNTTNPLESSSKHNIHPLKVPNLIGGKFVDSQACAIIDVIN
PATQEVVSEVPLTTYEEFKAAVSAAKQAYPSWRNTPVTTRQRIMFKLQELIRRDIDKLAMNITIEQGKTLKGAQGDVLRGLEVVEHACGMATLQMGE
FVPNASNGIDTYCLREPLGVCAGICPFNFPAMISLWMFPIAVTCGNTFILKPSEKNPGYCPYTFGKSSENFKRYIFFWTNKIMLLHESVNWDATHCG
AFIYLWNDLKRVKEDQGKENLWQSCLGFLSKHHLGEDRETFQRSLIATKKFLNKLLREIGENGSKPPERPQVENSKKGFLKQKDSILQKSKSSLNGH
LLGLFIGILVGSENCWERKKEIRSNLHVSCSVVMGDVKGELFENLYIFKLLIVFLPLLFEKVNKLESRKKRRDQTCFFIVFLLIFLHFLFSFPYDLS
LLFLRYKQGFRASMILAALAMEAGLPHGVLNIVHGTNDIVNYICDDDDIKAVSFVGSNTAGMNIYARAAARGKRVQSNMGAKNHAIIMPDASMEATL
NALVAAGFGAAGQRCMALSTAVFVGGSIPWEEELVACAKALKVNAGTEPGADLGPVISKEAKDRICRLVQNDVGSGARLVLDGRNIVWSDKERSTLV
LMMFPVSTLVNCPRSERYSLSECQVVNSHTLPKLRKASSKSHFWLGRLSESLCLRKHLGLWVIFIFLLFDSNVVAHCLILQACILSCFKVVGMDIYL
KRKMHVPGYEYGNFVGPTILCDVTTNMECYKEEIFGPVLLCMKADSLEEAITIVNRNKCSNGASIFTTSGVAARKFQNEVEAGLVGINVPVPVPLPF
SSFTGSKLSFAGDLNFCGKAGVQFYTQIKTVAQQWKDLPSRGHLRGCICLKNLIGMTLCSPHLKELKLLHQLLRGFMHLQRPLRGGRRKEEEERRMQ
CSNTCWASSVHPPGRFFTQSSHHNEDTSCPVVTLPLPSHVKSITSPSNPFVKHCFKLRHSSSYRYSHGSALVVGTTPIREIYKFQQSTQERTVEMDC
LLILDKAEIPEGLDDFSVRLVRVSSMVMKKLSGLQSTESVEAIALMRIPTSFFSVNDDTYEKDCRRWFQSPHRILVLDRIQDPGNLGTLLRSAMAFR
WGGVFLLSGCCDPFNGKALRASRGASFQLPIVSGSWIHLETLKNEFQMKMIAGHPDSNQKRKPVSPLSQGLADSLADVPLCLVLGSEGSGLSEKSWQ
LCELVSIPMAGEFESLNVSVAGGIFLYMLQPQNRRVGIGTEIGWL
>VvALDH7B5
MSFERKEYQFLAEIGVGPANPGCYINGEWKARGPLVSSVNPSNNQRIAVVTEASIEDYEEGLMACSEAAKTWMKIPAPKRGEIVRQIGEALRAKLGS
LGRLVSLEMGKILAEGIGEVQEIVDMCDYAVGLSRQINGSIIPSERPDHMMCEVWNPMGIVGVITAFNFPCAVLGWNACLALVCGNCVVWKGAPTTP
LVTIAVTKLVAEVLEKNNLPGAIFTSFCGGAEIGEAISKDARIPLVSFTGSSKVGLMVQQAVNQRFGKCLLELSGNNAIIIMEDADIGLAVRSVLFA
AVGTAGQRCTTCRRLLVHESIYETVLNQLIDVYKQVKMGDPLEKSTLVGPLHTRASKENFEKGIEIIKFQGGNILIGGSTVESEGNFVQPTIVEISP
NASVVKEELFGPVLYVMKFQTFEEAVEMNNSVPQGLSSSIFTRKPEVIFKWIGPHGSDCGIVNVNIPTNGAEIGGAFGGEKATGGGREAGSDSWKQY
MRRSTCTINYGNELPLAQGINFG
>VvALDH7D1
MIVECADMFARSEYMFLSEIDLSVVHPGYYVNGKWKGRSSSMVTSVNPVDNETIAAVTEGSIEDYEEGIQACSKAAKLWMKTPVSKRCEIVRQIGDA
LRAKLQLFGRLVSLEVGKILVAGIGEVQEVIDMCDYAAGLSEKLNLNASIRHERQNHVTLQLRNPFGVVGVITPFNFPCAVLGRNACMALVTGNCVV
WKGSRTTPLVTIAITKLVAGVLKNNNLPGAIFTSFCGGAPIGQAMAEDKRIPLVSFTGTSKVGLMVQQRVNDRFGKCLLELSGNNAITVMGDADIPL
VVQAVLLDAVGIAGQCRITCHRLFIQETIYELVIERLLLEYTLVTIGMGDPLKTGTLLGPLHTKALKRNFRTVMQKIKSQGGKVFIGDVVSTVGNFV
RPTIVEISPNADVVKEELFVPVLYVIKFTTFEEAMQINNSISPGSSNSIFTRKPHLVVPGIRSLGIDCGIVNVNLPTRGRGGAGSDSWEQYTRRTIW
>VvALDH10A9
METKIPCRQLFIDGKWVEPITKRRIPVINPATEQTIGLIPAATGEDVELAVDAARRAFARNKGADWAKAPGAVRAKYLRAIAAKITERKTELAKLEA
LDCGKPLDEAAWDIDDVASCFEYFADHAEALDAQQKAPLSLPMETFKCHILKEPVGVVGLITPWNYPLLMATWKVAPALAAGCTAILKPSELASVTC
LELADVCIEVGLPPGVLNILTGLGSEAGAPLSSHPHVDKIAFTGSTVTGSKIMTAAAQLVKPVSLELGGKSPILVFEDVDLDKAAEWTAFGCFWTNG
QICSATSRLLVHESIAAEFLDKLVKWTKNIKISDPFEEGCRLGPVVSREQYEKILKLVSTAKSEGATILCGGGRPQHLKMGFYIEPTIISDVTTSMQ
IWREEVFGPVLCVKTFSSEDEAIELANDTQYGLAGAVISNDLERCERVSKALNAGIVWINCSQPCFCQAPWGGNKRSGFGRELGPRGLDNYLSVKQV
TQYLSNEPWGWYQSPSKL
>VvALDH10B1
MMAVPIPSRQLFIDGEWREPLLKKRIPIINPATQEIIGDIPAATAEDVDIAVEAARRAFSKPDSWASTSGSFRAKFLRAIADKILERKIELAKLEVV
DCGKPIDEAISDMVSVAGCFKYYAELAEALDAKQRIPISIPMESFKTHVLKEPIGVVALITPWNYPLLMAAWKVAPALAAGCTAILKPSELASVTCL
ELAEVCRDVALPPGVLNILTGLGPEAGAPLASHPHVDKIAFTGSTATGIKIMTTAAQTIKPISLELGGKSPILVFEDVDLDNAIEWTLYGCFPNNGQ
ICSATSRLLVHENIAAKFVEKLIQWSKSIKISDPLEEGCRLGAIVSEGQYEKILNFISTAKSEGATILYGGVRPQHLKKGFFIEPTIITDVSTSMQI

WREEVFGPVLCVKTFATEEEATQLANDTHYGLGAAVVSNDLERCERLTKVLQAGVVWVNCSQPCFDQAPWGGIKHSGIGRELGEWGLENYLTVKQVT
QYTSDKQWGWYSPP
>VvALDH11A3
MAGSGVFAEILDGDVFKYYTDGAWKTSSSGKSVPIINPTTRSTQYKVQACTQEEVNKVMETAKNAQKLWAKTPLWKRAELLHKAAAILKEQKAPIAE
CLVKEIAKPAKDAVTEVVRSGDLVSYCAEEGVRILGEGKFLVSDSFPGNERSKYCLTSKIPLGVILAIPPFNYPVNLAVSKIGPALIAGNSLVLKPP
TQGAVAALHMVHCFHLAGFPKGLINCVTGRGSEIGDFLTMHPGVDCISFTGGDTGIAISKKAGMVPLQMELGGKDACIILEDADLDLAASSIVKGGF
SYSGQRCTAIKVVLVMESVADALVEKVNVKLAKLTVGAPEDDCDITPVVTESSANFIEGLITDAKQKEATFCQEYKREGNLIYPLLLDNVRPDMRIA
WEEPFGPVLPVIRINSVEEGIHHCNASNFGLQGCIFTRDINKAILIGDAMETGTVQINSAPARGPDHFPFQGFKDSGIGSQGITNSINMMTKTKSMV
INLPTPTYSMG
>VvALDH11B1
MAGTGVFAEIIDGDVYKYYSEGEWRKAVSGKSVAIINPTTRKTQYRVQACSQEEVNKAMEIAKSAQKIWAKTPLWKRAELLHKAAAILKEHKAPIAE
CLVKEIAKPAKDAVTEVVRSGDLVSYTAEEGVRILGEGKFLVSDSFPGNERSKYCLTSKIPLGVILAIPPFNYPVNLAVSKIAPALIAGNSIVLKPP
TQGAVSALHMVHCFHLAGFPKGVISCVTGKGSEIGDFLTMHPGVNCISFTGGDTGVAISKKAGMIPLQMELGGKDACIVLEDADLDLVAANIVKGGF
SYSGQRCTAVKVVLAMESIADTLVEKVNAKVAKLTVGPPEDDCDITPVVSESSANFIEGLVMDAKQKGATFCQEYRREGNLIWPLLLDNVRPDMRIA
WEEPFGPVLPVLRINSVEEGIHHCNASNFGLQGCVFTRDINKAILISDAMETGTVQINSAPARGPDHFPFQGLKDSGIGSQGITNSINMMTKIKSTV
INLPSPSYTMG
>VvALDH12A1
MRGRVPQIAALNWLGSLSLSRSIHNLPFATIEVEEISGSQPAEVQNLVQGKWTGSTSGETIVDPLNGEPFIQVAEVDETGIQPFVDSLSKCPKHGLH
NPFKAPERYLMLGDISNKAAHMLSLPKVSDFFTRLIQRVAPKSYQQALGEVYVTQKFLENFSGDQVRFLARSFAVPGNHLGQQSHGFRWPYGPVAIV
TPFNFPLEIPVLQLMGALYMGNKPILKVDSKVSIVMEQMIRLLHHCGLPMEDLDFINSDGKTMNKLLLEANPRMTLFTGSSRVADKLAVDLKGRIKL
EDAGFDWKILGPDVQEVDYVAWVCDQDAYACSGQKCSAQSIVFMHENWFKSSLISRMKDLAARRKLEDLTIGPVLSFTTEAMLEHMNKLLQIPGSEL
LFGGKALENHSIPPIYGALKPTAIYIPLEEMLKDGNYELVTREIFGPFQVVTDYKDNQLPRVLEALERMHAHLTAAVVSNDSLFLQEVIGKSVNGTT
YAGLRARTTGAPQNHWFGPAGDPRGAGIGTPEAIKLVWSCHREIIYDIGPLPSHWEIPPAT
>VvALDH18B1
MHMDAMDPTRAFVKDVKRLVIKFGTAVVTRSDGRLALGRLGALCEQIKELNSQGYQVIVVTSGAVGLGRQRLRYRSLLNSSFADLQKPQAELDGKAC
AAVGQNNLMALYDTLFSQLDVTSAQLLVTDNDFRDEAFRNQLTQTVDSLLALRVIPIFNENDAVSTRKAPYEDSSGIFWDNDSLAGLLALQLKADLL
VLLSDVDGLYSGPPSDPRSKLIHTYLEKGHQGQITFGDKSRVGRGGMTAKVKSAVYSSQAGIPVVITSGYATGSILKVLNGERIGTLFHRDAYKWVQ
VKEVGAREMAVAARESSRRLQAMSSQDRKKILLDIANALETNEELIKIENDADVEAAQLAGYEKSLVSRLVLKPGKISSLANSIRVLANMEEPIGHV
LKRTEVADGLILEKMSCPLGVLLIVFESRPDALVQIASLAIRSGNGLLLKGGKEAKRSNAILHKVITEAIPDSVGKKLIGLVTSREEIPNLLKLDDV
IDLVIPRGSNKLVSQIKDSTKIPVLGHADGICHVYVDKSANMDTAKHIVLDAKVDYPAACNAMETLLVHKDLVQTGGLNQLIVELRNEGVTLYGGPR
ASALLNLPEAHSFHHEYNSMACTVEIVDDVHSAIDHIHRHGSAHTDCIIAEDLEVAEVFLRQVDSAAVFHNASTRFCDGARFGLGAEVGISTSRIHA
RGPVGVEGLLTTRWILRGNGQVVNGDKGVTYTHKELTLQP
>VvALDH18B3
MDSSREFVKDVKRVIVKVGTAVVTRADGRLAVGRVGALCEQLKELNSQGYEVILVASGAVGVGIQRLRYRRLVNSSLDDLQKPQIELDSKACAAVGQ
SSLMALYDTLFSQLDVTASQLLVTNSDFRNPDFRMQLSETVGSLLDLRVIPIFNENDAISTRDHPNEDSSGIFCDNDSLATLLALELKADLLVLLSD
VEGLYNAPPNKPHAELIHTYVKEKHEGEITFGDKSKVGRGGMTAKVKAANQAADAGTPTVITSGYATDNIIKVLQGQRVGTLFHKDAHSWTLVKEIG
AREMAVAARECSRRLQALPSKDRRKILLDIADALEANESLIKVENEVDVAAAQEAGYDKSLLSRLTLKPGKISGLAKSIRMLADMKEPIGHVLSRME
LADGLILEKTSCPMGVLLVVFEARPDALVQIASLAIRSGNGLLLKGGKEANRSNAILHKVITEAIPDTVGEKLIGLVTTREQIPDLLKLDDVIDLVI
PRGSNKLVSQIKDSTKIPVLGHADGICHVYIDKSANMKMAKHIVLDAKTDYPAACNAMETLLVHKDFLSNGGFNKLVTELQRKGVTLHGGPRASALL
NISQVNSFHHEYNSMACTVEIVDDVHSAIDHIHRHGSSHTDCIITEDSKIAEIFLLQVDSAAVFHNASTRFCDGARFGLGAEVGISTSRIHARGPVG
VEGLLTSRWILKGNGHIVDGDEGVIYTHKSLALQS
>VvALDH22A1
MAFWWPLLVLGFAFALCRFLLMLIPPNVPSIDVDASDVLDDGKTKENSFIYIPSRGRTPEKVQCYEPATMKYLGFCPALRPDEVREHVAQARKAQKI
WARSSFKQRRQFLRILLKYIIEHQELICEISSRDTGKTMVDASLGEIMTTCEKITWLLSEGERWLKPEYRSTGRSMLHKTAKVEFHPLGVIGAIVSW
NYPFHNIFNPMLAAVFSGNGVVIKVSEHASWSGCFYLRIIQAALAAVGAPENLVDVITGFAETGEALVSSVDKIIFVGSPGVGKTIMRNASDTLIPV
TLELGGKDAFIVCEDVDVPHVAQIAVRAALQSSGQNCAGAERFYVHQDIYSKFVAEVVRIVKSVTAGPPLSGKYDMGAICMQEHSEKLQNLVNEALD
KGAEFAGRGSFGNLGEDAVDQFFPPTVLVNVNHSMQLMQEEAFGPILPIMKFSSDEEVVKLANDSRYGLGCAVFSGSQRRAKAIASQIHCGMAAIND
FASTYMCQSLPFGGVKDSGFGRFAGIEGLRACCLVKSVVEDRWWPFIKTKIPKPIQYPVADNGFEFQESLVEALYGLNVWDRLRALVHVLKMLTEQN
TPSNSTKRKND
>SlALDH2B1
MDSLLMQPQTFPTFDPRTGEAITTVAEADTEDVNRAVFAARKAFDEGPWPKMTCAERSCIMLQFADLLERHSDELAALETWDKGKPYEQAANEEIPM
LIRLFRYYAGWADKIHGLTAPADSLHHVQTLHEPIGVAGQIIPWNFPLLMFAWKVGPALACGNTVVLKPAEQTPLSALYVSKLFHEVGLPPGVLNVI
PGSGSAGADLASHMDVDKIAFTGSTETGKAVVGAAAKSNLKPVTLELGGKSPFIICEDADVDKAVELAHSAVFFNQGQCCCAGSRTFVHERVYDEFV
EKAKARALKRIVGDPFKKGVEQGPQIDTEQFEKILKYIKSGTESGATLESGGEKLGSKGFYVQPTVFSNVQDNMLIARDEIFGPVQSLLKFKDVEEV
IRRANSSHYGLAAGVFTQNIDAANTISRALRVGTVWVNCFNIFDAAIPFGGYKMSGHGREKGVYSLSNYLQVKAIVTPLKNPAWL
>SlALDH2B3
MAARVFLSRSVHLLSKGKRSHLGKIAAYKYSTAAAFEEPVKPTVNVDHTKLLINGQFVDSASGKTFPTLDPRTGEVIAHIAEGDAEDINRAVAAARK
AFDEGPWPRMTAYERSKILLRLADLIEKHNDQIATLETWDTGKPYAQAAKIEVPMVVRLLRYYAGWADKIHGMTIPADGPYHVQTLHEPIGVAGQII
PWNFPLLMFSWKIGPALACGNTIVLKTAEQTPLSALYVASLFQEAGLPEGVLNIISGYGATAGASLCSHMDVDKLAFTGSTETGKTILELAAKSNLK
PVTLELGGKSPFIVCEDADIDTAVEQAHFALFFNQGQCCCAGSRTYVHEKVYDEFLEKAKARALKRVVGDPFKSGTEQGPQIDSKQFDKIMKYIRSG
VDSGATLETGGEQFGKKGYYIRPTVFSNVKDDMLIAQDEIFGPVQSILKFKDLDEVVRRANSSRYGLAAGVFSQNIDTANTLARALRVGTVWINCFD
TFDATIPFGGYKMSGQGREKGEYGLKNYLQVKAVVTPLKNPAWL
>SlALDH2B4
MAFRLITSRLSHSSSSSLASLFQGRNSRVAATAALRYTTAAQDPIKPSVNVEYTKLFINGQFVDSASGKTFPTLDPRTGEVIAHVAEGDVEDINRAV
VAARNAFDEGPWPKMSAYERSKILFRIADLIEKHNDEIATLETWDSGKLYQQVATIEIPMIVRLLRYYAGWADKIHGMTVPADGPYHVQTLHEPIGV
VGQIIPWNFPLLMFAWKIGPALACGNTVVLKTAEQTPLSALYVSKLLQEAGLPEGVLNVISGFGPTAGAALSSHMDVDKLAFTGSTDTGKTIMSLAA

NSNLKPVTLELGGKSPFIVCEDADVDQAVEFAHFALFFNQGQCCCAGSRTYVHESIYDEFVEKAKARALKRTVGDPFDSSNEQGPQISSEQFEKVLK
YIRSGIESGATLETGGDRLGTQGYYIKPTVFSNVKDDMLIATDEIFGPVQSILKFKDHDEVIRRANATKYGLAAGVFTKNIDTANTFMRALRVGTIW
INCFDIFDAAIPFGGYKMSGQGREKGEYSLKQYLQVKAVVTSLKNPAWL
>SlALDH2B7a
MAARRISSLLSRSLNLPSVSASLGRSHGVARHINRFSTAAAVEEIITPPVQINHTKLLINGQFVDSASGKTFPTLDPRTGEVIANVAEGDLEDVNRA
VAAARKAFDEGPWPKMSAYERSRIMLKFADLVEKHNDEIAALETWDNGKPYLQAAQAEVPSFVRLFRYYAGWADKIHGLTVPADGPHHVQILHEPIG
VAGQIIPWNFPLLMMAWKVGPALACGNTIVLKTAEQTPLTALYVANLLHEAGLPPGVLNIVSGFGPTAGAALASHMDVDKLAFTGSTETGQTVLQLA
AKSNLKPVTLELGGKSPFIICEDADIDHAVELAHFALFFNQGQCCCAGSRTYVHERVYDEFVEKAKARAMRRVVGDPFKKGVEQGPQIDSEQFQKIL
RYIREGRDSSATLECGGDRIGSKGYFIQPTVFSNVKEDMSIAQDEIFGPVQCVFKFKDIGEVIKRANNTRYGLAAGVFTKNIDTANTLTRGLRAGTV
WVNCYDIFDAGIPFGGYKMSGMGREKGIYSLNNYLQVKAVVTPLKNPAWI
>SlALDH2B7b
MNTHTRRIISQLRTSIRYSNYWRKGIRRFCSSAVVHEEPITPPVEVKYNQLLINGQFVDAASGKTFPTFDPRTGEAITTVAEADTEDVNRAVFAARK
AFDEGPWPKMTCAERSCIMLQFADLLERHSDELAALETWDKGKPYEQAANEEIPMLIRLFRYYAGWADKIHGLTAPADSLHHVQTLHEPIGVAGQII
PWNFPLLMFAWKVGPALACGNTVVLKPAEQTPLSALYVSKLFHEVGLPPGVLNVIPGSGSAGADLASHMDVDKIAFTGSTETGKAVVGAAAKSNLKP
VTLELGGKSPFIICEDADVDKAVELAHSAVFFNQGQCCCAGSRTFVHERVYDEFVEKAKARALKRIVGDPFKKGVEQGPQIDTEQFEKILKYIKSGT
ESGATLESGGEKLGSKGFYVQPTVFSNVQDNMLIARDEIFGPVQSLLKFKDVEEVIRRANSSHYGLAAGVFTQNIDAANTISRALRVGTVWVNCFNI
FDAAIPFGGYKMSGHGREKGVYSLSNYLQVKAIVTPLKNPAWL
>SlALDH2B7c
MAFRLITSRLSHSSSSSLASLFQGRNSRVAATAALRYTTAAQDPIKPSVNVEYTKLFINGQFVDSASGKTFPTLDPRTGEVIAHVAEGDVEDINRAV
VAARNAFDEGPWPKMSAYERSKILFRIADLIEKHNDEIATLETWDSGKLYQQVATIEIPMIVRLLRYYAGWADKIHGMTVPADGPYHVQTLHEPIGV
VGQIIPWNFPLLMFAWKIGPALACGNTVVLKTAEQTPLSALYVSKLLQEAGLPEGVLNVISGFGPTAGAALSSHMDVDKLAFTGSTDTGKTIMSLAA
NSNLKPVTLELGGKSPFIVCEDADVDQAVEFAHFALFFNQGQCCCAGSRTYVHESIYDEFVEKAKARALKRTVGDPFDSSNEQGPQISSEQFEKVLK
YIRSGIESGATLETGGDRLGTQGYYIKPTVFSNVKDDMLIATDEIFGPVQSILKFKDHDEVIRRANATKYGLAAGVFTKNIDTANTFMRALRVGTIW
INCFDIFDAAIPFGGYKMSGQGREKGEYSLKQYLQVKAVVTSLKNPAWL
>SlALDH2B7d
MAARVFLSRSVHLLSKGKRSHLGKIAAYKYSTAAAFEEPVKPTVNVDHTKLLINGQFVDSASGKTFPTLDPRTGEVIAHIAEGDAEDINRAVAAARK
AFDEGPWPRMTAYERSKILLRLADLIEKHNDQIATLETWDTGKPYAQAAKIEVPMVVRLLRYYAGWADKIHGMTIPADGPYHVQTLHEPIGVAGQII
PWNFPLLMFSWKIGPALACGNTIVLKTAEQTPLSALYVASLFQEAGLPEGVLNIISGYGATAGASLCSHMDVDKLAFTGSTETGKTILELAAKSNLK
PVTLELGGKSPFIVCEDADIDTAVEQAHFALFFNQGQCCCAGSRTYVHEKVYDEFLEKAKARALKRVVGDPFKSGTEQGPQIDSKQFDKIMKYIRSG
VDSGATLETGGEQFGKKGYYIRPTVFSNVKDDMLIAQDEIFGPVQSILKFKDLDEVVRRANSSRYGLAAGVFSQNIDTANTLARALRVGTVWINCFD
TFDATIPFGGYKMSGQGREKGEYGLKNYLQVKAVVTPLKNPAWL
>SlALDH2C4
MAEMNGNSETQFQIPKIKFTKLFINGEFVDSVSGNTFETIDPRNEEVIARISEGDKEDIDLAVKAAREAFDNGPWPRLSAAERRRIMLKFADLIIEN
AEEIAALDAMDAGKLFVPVKNMDIPAAAEIIRYYAGAADKIHGTTLKMSREMQGYTLLEPIGVVGHIIPWNFPTQMFLMKVGPALAAGCTMIVKPAE
QTPLSALYYAQLAKQAGVPDGVINVVTGFGSTAGAALCSHMDVDKISFTGSTEVGRLVMQAAALSNLKPVSLELGGKSPFIVFDDVDVDKVAPLALV
GILFNKGEICVAGSRLFIQEGIYDKFVKKLEQMVKTWVVGDPFDPNSHQGPQVDKKQYERVLSYIEHGKREGAKLLTGGNALDRKGYFIEPTIFIDV
EDDMKIAKEEIFGPVLAVMKFKTVEEVIKRANCTNYGLAAGVMTNNLNIANTVSRSIRAGVIWINCYFAFDPDCPYGGYKCSGFERDLGMEGLHKYL
QVKSVATPIYNSPWL
>SlALDH3F1a
MSTTTMTKLCNSYPTTPQIMLECEKELEVLKETFKSGKTKEESWRRSQLKNLLKLLEEKENDIFKALKQDLGKHKVEAYRDEVGTLVKSVHYALDGL
KQWMSPKKAKLPIAAFPSSAELLPEPLGLVLIISSWNFPFSLSLEPLIGAIAAGNVVLLKPSDQAPASSSVLAKIIPNYLDNKAIKVIEGDYTVGDK
LLQQKWDKIFFTGSPKVAQIVMGAAAKHLTPVTLELGGKCPAIIDSLSSSWDKKIAMKRILSGKFGSCAGQACIGIDYILVDNTFVNELVKLIKLGI
PKMLGENPKESHSISRIVNKNQFLRLKNLLDEPMVKKSIIYGGSSDEDNLYIEPTVLLDPPLQSTIMTDEIFGPLLPIITLDKIEDSIEFINARPKP
LTIYAFTKNEEFKRKITKGTSSGSLVFNDTIIQYAADTLPFGGVGQSGFGRYHGKFSFDTFSHEKAIARRSFLTDIWFRYPPWSDHTLQLFRSAFIY
DYLSVVLITLGLKRA
>SlALDH3F1b
MTGLSLEPLIGAIAYENVALLKPSDQAPASSSVLAKIIPNYLDNKAIKVIEGDYTVGDKLLQQKWDKIFFTGSPKVAQIVMGAAAKHLNPVTLELGG
KCPAIIDSLSSSWDKKIAMRRILSGKFGSCAGQACSGIDYILVDNIFVNELVKLIKLGIPKMLGENPKESHSISRIVIKNQFLRLKNLLDEPMNQRY
IEPTVLLYPPVQSTIMIDEIFGPLSPIITLDKIEDNIEFINARPKPLTIYAFTKNEEFKGKITKGTCSGSLVFSDTIIQDLEQFLP>SlALDH3F1c
MRTEKANHLKDKMEELSLNSSNANLVESSGTVVKDRKVGHRAARCYQRKGQDSKKEGQSDVQANLVEGNEVVVVVVVEANLQANKIGRVLETGASRH
FSANKELLHDFEESTDRECIYIGDSTTDVVMGLSLEPLIGAIAAGNVALLKPLDQAPASSSVLAKIIPNYLDNKAIKVIEGDYTVKLIKLGIPIMLG
ENPNESHSISRIVNKNQFLRLKNLLDEPMVKKSIIYGGSSDEDNLDIEPTVLLDPPVQSTIMADEIFGPLSPIITLDKIEDSIEFINARPKPLTIYA
FTKNEEFKRKITKRTSSGSLVFNDTIIQYATDTLPFGGVGQSGFGRYHGKFSFDTFSHEKAIAKRSFLTDIWFRYSPWSDHTLQLFRSAFIYDYISV
VLITLGLKRA
>SlALDH3F1d
MDVVEEDVLGEVTTAFRSRRTRSVAWRKAQLQAILKLLDENEEEIFEALRQDLGKHPVESYRDEVGVVRKSATNALRCVEKWMAPQKAPIPLVLFPA
RGAVVSEPLGVVLIFVSWNFPISLALDPVIGAISAGNAIVLKPSELAPKCSSVLANTIPRYLDPEAIKVVEGGHDVSEQLLQLKWDKIFFTGSPRVG
RLIMSAAAKHLTPVTLELGGKCPTILDRLSNFSDLQVAVKRIVGGKWGPCNGQACIGIDYVLVETQFAPVLIELLEKSIKTFYGENLKTLANLARIV
NKHHFDRVHNLLKDPKVAASVVYGGSVDEENMAIEPTILLNPPLDADIMNEEIFGPLLPIITLKNIEESIPFINSRPKPLAIYAFTKNDSLKEKILQ
ETSSGSLTFNDAMIQFLCDTLPFGGVGQSGYGRYHGKFTFDTFSHEKAVLHRSFLVELESRYPPWNDFKMEFVRLAYNYDYLGMILLLLGLRGLFRT
NRRQ
>SlALDH3H1
MDAEAIVKELRGTYGSGKTKSYEWRVSQLKALLKIAENHEKEITDALYSDLSKPELEAFIHEVSMMKTACKLALKELKWWMKPEKVKTSLTSFPSSA
EIVPEPLGVVLVISAWNYPFLLSLDPVIGAIAAGNAVVLKPSEIAPATSSVLAKLLGQYMDVSAIRVVEGAVPETTALLEQKWDKIFYTGNGKVGRI
VLAAAAKHLTPVVLELGGKSPVVVDSNIDYKIAVRRIIAGKWGCNNGQACISPDYIITTKESVPKLLDAMKQELEKFYGKDPLKSGDLSRIVNANHF

QRLSKLLDDNKVVDKVVHGGQRDENNLKISPTILLDVPEDSLIMKEEIFGPLLPIITVNKVEDSIQFIKAREKPLAAYLFTSNKKLEEEFVMNISAG
GLLINDTTLQVALSTLPFGGVGESGMGSCHGKFSFDTFSHKKAVLRRSFAGDVPARYPPYTAGKARFLKALLNGDIIGLIRALIGW
>SlALDH5F1a
MQMIRVRTRMALSACAMLYRSSISGPVRLMTTDTQSVAAKLSSSGLLRSQALIGGKWVDAYDGKTIKVHNPATGEVITDVPCMGGRETNDAISSAYD
AFSSWSKLTAAERSRYLRKWYDLIMAHKEELGQLMTLEQGKPLKEAIGEVSYGAGFIEFSAEEGKRIYGDIIPSPLADRRLFVLKQPVGVVGAITPW
NFPLAMITRKVGPALACGCTVVIKPSELTPLTALAAAELSIQAGIPPGVVNVVMGNAPDIGDALLASPQVRKITFTGSTKVGKKLMEGAAATVKKVS
LELGGNAPCIIFDDADLEVALKGALATKFRNTGQTCVCANRILVQEGIYDKFANAFAKAVQNMKVGDGFTEGVEQGPLINEAAVQKVEYFVDEATSK
GAKVLVGGKRHSLGMTFYEPTVVTGVNSEMLLAKEEVFGPVAPLLKFKTDEEAIQMANDTNAGLAAYIFSTNIKRAWRVTEALEYGIVGVNEGLVST
EVAPFGGVKQSGLGREGSKYGMDEYLEMKYVCLGSMS
>SlALDH5F1b
MQMIRVRTRMALSACAMLYRSSISGPVRLMTTDTQSVAAKLSSSGLLRSQALIGGKWVDAYDGKTIKVHNPATGEVITDVPCMGGRETNDAISSAYD
AFSSWSKLTAAERSRYLRKWYDLIMAHKEELGQLMTLEQGKPLKEAIGEVSYGAGFIEFSAEEGKRIYGDIIPSPLADRRLFVLKQPVGVVGAITPW
NFPLAMITRKVGPALACGCTVVIKPSELTPLTALAAAELSIQAGIPPGVVNVVMGNAPDIGDALLASPQVRKITFTGSTKVGKKLMEGAAATVKKVS
LELGGNAPCIIFDDADLEVALKGALATKFRNTGQTCVCANRILVQEGIYDKFANAFAKAVQNMKVGDGFTEGVEQGPLINEAAVQKVEYFVDEATSK
GAKVLVGGKRHSLGMTFYEPTVVTGVNSEMLLAKEEVFGPVAPLLKFKTDEEAIQMANDTNAGLAAYIFSTNIKRAWRVTEALEYGIVGVNEGLVST
EVAPFGGVKQSGLGREGSKYGMDEYLEMKYVCLGSMS
>SlALDH6B2
MMQFSVHRVKKLRSLTPGIFAVANHHFSVATESSWKHRTSLRVPNLIGGSFVDSQSSEFVDVINPATQEVVSQIPLTTDKEFKSAVSAAKEAFPSWK
NTPITTRQRVMLKFQELIRKNMDKLAFNVTTEQGKTLKDAQGDVFRGLEVVEHACGMATLQMGEYGSNVSNGIDTYSLREPLGVCAGICPFNFPAMI
PLWMFPVAATCGNTFILKPSEKDPGASMMLAELAMEAGLPDGVLNIVHGTHDVVNAICDDDDIRAVSFVGSNQAGMHIYSRASAKGKRVQSNMGAKN
HGVVMPDANIDSTVNALVGAGFGAAGQRCMALSTVVFVGDSKPWEEKLLERAKTLKVNAGTEPDADLGPVISKQAKERVCRLVQSGVDSGAKLLLDG
RDIVVPGYEKGNFVGPTILCGVTPDMECYKEEIFGPVLLCMQANSLDEAINIVNQNMYGNGAAIFTTSGVAARKFQTEIESGQIGINVPIPVPLPFF
SFTGSKASFVGDLNFYGKAGVQFYTQIKTVTQQWKDLSSGSGNSLAMPTSQK
>SlALDH7B4a
MTNFTMEEYEFLKELGIGPQNLGCYVNGTWKATGPVISTVNPASNQIIAEVYEASARDYEEGMSACAEAAKIWVQVPAPKRGEIVRQIGDALRANLQ
QFGRLVSLEMGKILPEGIGEVQEVIDMCDFAVGLSRQLNGSIIPSERPNHMMLETWNPLGIVGVITAFNFPCAVLGWNACIALVCGNCVVWKGAPTT
PLVTIAMTKIVASVLEKNNLPGSIFTAFCGGAAVGQAIAMDTRIPLVSFTGSSKVGLAVQQTVSQRFGKCLLELSGNNAIIIMDDADIKLAVRSVLF
AAVGTAGQRCTTCRRLLVHESIYDKVLEPLVDVYKQVKIGDPLEKGTLLGPLHTRTSRENFEKGIHNIKSQGGKILTGGSVVESEGNFVRPTIVEIS
SKAEIVKEELFAPVLYVMKFKTFEEAVEINNSVPQGLSSSIFTRNPENIFKWIGPQGSDCGIVNVNIPTNGAEIGGAFGGEKGTGGGREAGSDSWKQ
YMRRSTCTINYGSELPLAQGINFG
>SlALDH7B4b
MTNFTMEEYEFLKELGIGPQNLGCYVNGTWKATGPVISTVNPASNQIIAEVYEASARDYEEGMSACAEAAKIWVQVPAPKRGEIVRQIGDALRANLQ
QFGRLVSLEMGKILPEGIGEVQEVIDMCDFAVGLSRQLNGSIIPSERPNHMMLETWNPLGIVGVITAFNFPCAVLGWNACIALVCGNCVVWKGAPTT
PLVTIAMTKIVASVLEKNNLPGSIFTAFCGGAAVGQAIAMDTRIPLVSFTGSSKVGLAVQQTVSQRFGKCLLELSGNNAIIIMDDADIKLAVRSVLF
AAVGTAGQRCTTCRRLLVHESIYDKVLEPLVDVYKQVKIGDPLEKGTLLGPLHTRTSRENFEKGIHNIKSQGGKILTGGSVVESEGNFVRPTIVEIS
SKAEIVKEELFAPVLYVMKFKTFEEAVEINNSVPQGLSSSIFTRNPENIFKWIGPQGSDCGIVNVNIPTNGAEIGGAFGGEKGTGGGREAGSDSWKQ
YMRRSTCTINYGSELPLAQGINFG
>SlALDH10A8
MAIPNIRIPCRQLFIDGEWREPLKKNRLPIINPANEEIIGYIPAATEEDVDMAVKAARSALRRDDWGSTTGAQRAKYLRAIAAKVLEKKPELATLET
IDNGKPWFEAASDIDDVVACFEYYADLAEALDSKKQTEVKLHLDSFKTHVLREPLGVVGLITPWNYPLLMTTWKVAPALAAGCAAILKPSELASITS
LELGEICREVGLPPGALSILTGLGHEAGSPLVSHPDVDKIAFTGSGPTGVKIMTAAAQLVKPVTLELGGKSPIVVFDDIHNLDTAVEWTLFGCFWTN
GQICSATSRLIIQETIAPQFLARLLEWTKNIKISDPLEEDCKLGPVISRGQYEKILKFISTAKDEGATILYGGDRPEHLKKGYYIQPTIITDVDTSM
EIWKEEVFGPVLCVKTFKIEEEAIELANDTKFGLGAAILSKDLERCERFTKAFQSGIVWINCSQPCFWQPPWGGKKRSGFGRELGEWSLENYLNIKQ
VTQYVTPDEPWAFYKSPSKL
>SlALDH10A9
MANRNVPIPRRQLYIGGEWREPVKKNRIPIINPATEEIIGDIPAATAEDVDIAVEAARKAIARDDWGSTTGAQRAKYLRAIAAKVLEKKSVLATLES
LDSGKTLYESAADMDDVAGCFEYYAGLAEALDSRRMTPVNLNSDSYKSYVLREPLGVVGLITPWNYPLLMAIWKVAPALAAGCAAILKPSELASITC
LELGEICREIGLPSGALNILTGLGPEAGGPLASHPHVDKISFTGSGPTGSKIMTAAAQLVKPVSLELGGKSPIVVFDDIDNLDIAAEWTLFGIFANT
GQVCSATSRLIVQENIASAFMDRLLKWTKNIKISDPLEEDCKLGPVVSAGQYEKVLKFISNAKSEGATILCGGERPQHLKKGYYVQPTIITDVNTSM
EIWKEEVFGPVLCVKTFKTEEQAIELANDTKYGLGAAVMSKDVKRCERFTKAFQTGIIWINCSQPTFNELPWGGKKRSGFGRDLGKWGLENFLNIKQ
VTEYTSAEPLAFYKSPSKN
>SlALDH11A3a
MAGNGVFAEIIDGEVYKYYCEGEWKKSASGKSVAIINPTTRKTQYKVQACTQEEVNKVMEIAKAAQKSWAKTPLWKRAELLHKAAAILKEHKAPIAE
CLVKEIAKPAKDAVTEVVRSGDLVSYTAEEGVRILGEGKFLVSDSFPGNERTKYCLTSKIPLGVILAIPPFNYPVNLAVSKIAPALIAGNSLVLKPP
TQGAVAALHMVHCFHLAGFPKGLISCVTGKGSEIGDFLTMHPGVNCISFTGGDTGVAISKKAGMVPLQMELGGKDACIVLEDADLDLAAGNIVKGGF
SYSGQRCTAVKVVLVMESVADILVEKVNAKVAKLTVGPPEDNCDITPVVSESSANFIEGLVMDAKEKDATFCQPYKREGNLIWPLLLDNVRPDMRIA
WEEPFGPVLPVIRINSVEEGIHHCNASNFGLQGCVFTKDINKAILISDAMETGTVQINSAPARGPDHFPFQGIKDSGIGSQGITNSINMMTKVKTTV
INLPTPSYTMGKL
>SlALDH11A3b
MAGNGVFAEIIDGEVYKYYCEGEWKKSASGKSVAIINPTTRKTQYKVQACTQEEVNKVMEIAKAAQKSWAKTPLWKRAELLHKAAAILKEHKAPIAE
CLVKEIAKPAKDAVTEVVRSGDLVSYTAEEGVRILGEGKFLVSDSFPGNERTKYCLTSKIPLGVILAIPPFNYPVNLAVSKIAPALIAGNSLVLKPP
TQGAVAALHMVHCFHLAGFPKGLISCVTGKGSEIGDFLTMHPGVNCISFTGGDTGVAISKKAGMVPLQMELGGKDACIVLEDADLDLAAGNIVKGGF
SYSGQRCTAVKVVLVMESVADILVEKVNAKVAKLTVGPPEDNCDITPVVSESSANFIEGLVMDAKEKDATFCQPYKREGNLIWPLLLDNVRPDMRIA
WEEPFGPVLPVIRINSVEEGIHHCNASNFGLQGCVFTKDINKAILISDAMETGTVQINSAPARGPDHFPFQGIKDSGIGSQGITNSINMMTKVKTTV
INLPTPSYTMG
>SlALDH11A4a

MALRMWASSTANALRVSSTVSRTNFSLSRCFSTVLEGLKYASSHEWVKHEGSVATIGITDHAQDHLGEVVFVDLPDSGTSVSHGSSFGAVESVKATS
DINSPISGEIVEVNTKLSETPGLINSSPYEDGWMIKVKPSNPSELESLMGAKEYTKLCDEEEIH
>SlALDH11A4b
MALRMWASSTANALRVSSTVSRTNFSLSRCFSTVLEGLKYASSHEWVKHEGSVATIGITDHAQDHLGEVVFVDLPDSGTSVSHGSSFGAVESVKATS
DINSPISGEIVEVNTKLSETPGLVKCLLTHISTRALMKTDG
>SlALDH12A1
MMYRLSAYRQLQKRASSSHLNWITLFDSRRSNHTLSFATVKAEEVSGSQPAEVHNLVQGKWTKSSSWNTILDPLNGQPFIKVAEVNESELQPFVESL
SKCPKHGLHNPFKAPERYLMLGDVSTKAAHALGLPEVSDFFAKLIQRVSPKSYQQALVEVLVTQKFLENFCGDQVRFLARSFAVPGNHLGQQSHGFR
WPYGPVAVIAPFNFPLEIPLLQLMGALYMGNKPVLKVDSKVCIVMEQMLRLLHECGLPVDDVDFINSDGKTMNKLLVEAKPRMTLFTGSSRVAEKLA
DDLSGRVKLEDAGFDWKILGPDVNEVDYVAWVCDQDAYACSGQKCSAESILFMHENWSKSSLLDKMTELAARRKLDDLTIGPVLTVTTETMLDHAKK
LLQIPGSRLLFGGEALQNHSIPKIYGAIKPTAIFVPLEEILKDEHYPLVTKEIFGPFQVVTEYKDNQLPLVLDALEKMHAHLTAAVVSNDILFLQKV
IGNSVNGTTYAGLRARTTGAPQNHWFGPAGDPRGAGIGTPEAIKLVWSCHREIIYDVGPMPLGWKVPAST
>SlALDH18B1
MDSADPARAFVKDVKRIIIKVGTAVVTRGDGRLALGRMGSLCEQIRELTSQGFEVILVTSGAVGVGRQRLRYRKLINSSFADLQKPQGDLDGKACAA
VGQNGLMALYDTLFSQLDVTSAQLMVTDNDFRDPDFRRQLNETVNSLLCLKVVPIFNENDAISTRKAPYEDSSGIFWDNDSLAALLALELKADLLVL
LSDVEGLYTGPPTDPQSELIHTYVKEKHEGLITFGDKSRVGRGGMTAKVKAAVYAAYAGIPVVITSGFANNNIIKALDGQRVGTLFHREAIKWASIG
DFDAREMAVSARECARRLQTLSSQERSKILLDIADALEAKEEEILAENEADVAAAQQAGYENALISRLAMKPGKISSLANSVRVLANMDEPVGRILK
RTELADGIILEKTSSPLGVLLIIFESRPDALVQIASLAVRSGNGLLLKGGKEAKRSNAILHKVITSSIPPIVGERLIGLVTSREEIPELLKLDDVID
LVIPRGSNKLVSQIKAATKIPVLGHADGICHVFIDKSADLDMAKRIVLDAKTDYPAACNAMETLLVHEDLVQTGGLNDLILELQEKGVSLFGGPKAS
SVLNIPEANSFHHEYGALACTVEIVEDVNTAIEHIHRHGSAHTDSIITEDKEVAELFLRQVDSAAVLHNASTRFSDGFRFGLGAEVGISTSRIHARG
PVGVEGLLTTRWLARGSGQVVDGDKEIVYTHKDLNLEA
>SlALDH18B2
MDSADPARAFVKDVKRIIIKVGTAVVTRGDGRLALGRMGSLCEQIRELTSQGFEVILVTSGAVGVGRQRLRYRKLINSSFADLQKPQGDLDGKACAA
VGQNGLMALYDTLFSQLDVTSAQLMVTDNDFRDPDFRRQLNETVNSLLCLKVVPIFNENDAISTRKAPYELSLQDSSGIFWDNDSLAALLALELKAD
LLVLLSDVEGLYTGPPTDPQSELIHTYVKEKHEGLITFGDKSRVGRGGMTAKVKAAVYAAYAGIPVVITSGFANNNIIKALDGQRVGTLFHREAIKW
ASIGDFDAREMAVSARECARRLQTLSSQERSKILLDIADALEAKEEEILAENEADVAAAQQAGYENALISRLAMKPGKISSLANSVRVLANMDEPVG
RILKRTELADGIILEKTSSPLGVLLIIFESRPDALVQIASLAVRSGNGLLLKGGKEAKRSNAILHKVITSSIPPIVGERLIGLVTSREEIPELLKLD
DVIDLVIPRGSNKLVSQIKAATKIPVLGHADGICHVFIDKSADLDMAKRIVLDAKTDYPAACNAMETLLVHEDLVQTGGLNDLILELQEKGVSLFGG
PKASSVLNIPEANSFHHEYGALACTVEIVEDVNTAIEHIHRHGSAHTDSIITEDKEVAELFLRQVDSAAVLHNASTRFSDGFRFGLGAEVGISTSRI
HARGPVGVEGLLTTRWLARGSGQVVDGDKEIVYTHKDLNLEA
>SlALDH19
MALSVQEMGQRAKKATAQVAGLSLATRNTLLKNMGAALLMRQDEIIAANQQDLVAYGASLSRPMQKRLTLDSDALTAIAESLAAVATLPDPLAGPYD
TWENHAGLKIVKKIVPLGVVAMIYEARPNVTVDAAALALKSGNAVILRGGKEAIHSNTVLATILRDVLIDQNLNPDIIQLITDTTHESVNTLLHMRE
AIDVLIPRGSAAFIDYVVANATVPVIETGAGNTHIFVDASADQAAALRIIHNAKTQKPAVCNAAEKLLIHEAIAQEFLPKIADRLIAARVALRGDQA
SLGIDGRLTPASDADWDTEYNDLVMGIKIVPDVTAAIDWINTHTTHHSETIISQDPDNIAAFMNQVDAAVVYQNASSRFTDGFEFGFGAEIGISTQK
LHARGPMGLPALTTIKYEALAMAIRA
>SlALDH22A1
MAFWWPLIVIAIAFAICKLLLMLIPDNVPSIDVDTSDVLDDGNQTKDNSFIYIPSRRHTDKVQCYEPATMKYLGYFPALKPDEVKERVVQARKAQKI
WAKSSFKQRRLFLRILLKYIIEHQDLICNISSRDTGKTMVDASLGEIMTTCEKIHWLLSEGEKWLKPEYRSCGRSMLHKVAKVEFSPLGVVGAIVSW
NYPFHNIFNPMLAAVFSGNSIVIKVSEHASWSGCFYLRIIQTALAAVGAPENLVEVITGFAETGEALVSSVDKIIFVGSPGVGKKIMRSASDTLIPV
TLELGGKDAFIVCEDVDVPHVAQIAARGALQSSGQNCAGAERFYVHKDVYSSFVAEVVKIVKSVTAGPPLSGKYDMGAICMQEHSERLQYLVNDALD
KGAEIVARGSVGNIGEGAVDQYFPPTVIVNVNHTMKLMQEEAFGPILPIMKFSSDEEVVQLANDSSYGLGCAVFSGSQRRARQIASQLHCGVAAVND
FASNYMCQSLPFGGVKDSGFGRFAGIEGLRACCLVKSVVEDRWWPFIKTKIPKPIQYPIAENGFEFQESLVHTLYGLNIWDRLRALVNVLKILSEQP
PAPTSNRRRND
>PtALDH2B3
MAARRISSLLSRSLSASASFLLSRGKNPSRGRSIYRFITAKALEEPITPPVQISYTQHFINGKFVDAASGKTFPAYDPRTGEVIAHVAEGDNEDVNR
AVAAARKAFDEGPWPKMSAYERSLIMLRFADLVDKHRDELAALESWNSGKPYEQSAKSELPSFARLFRYYAGWADKIHGLTVPADSNHYVQTLHEPI
GVAGQIIPWNFPLIMLAWKVGPALACGNTIVLKSAEQTPLTALHAAKLFQEAGLPPGVLNVVSGYGPSAGAALASHMNVDKLAFTGSTETGKIILEL
AAKSNLKSVTLELGGKSPFIVCEDADVDKAVELAHHALFFNQGQCCCAGSRTYVHERVYDEFIEKAKARALRRVVGDPFKKGVEQGPQIDSDQFEKV
LRYIRSGVESNATLECGGQRFGSKGYFIQPTVFSNVEDDMLIAQDEIFGPVQSILKFKNVDEVIRRSNSTRYGLAAGIFTKNVDTANTLSRALRVGT
VWVNCFDVFDAAIPFGGYKMSGIGREKGIYSLNNYLQVKAVVTPLKNPAWL
>PtALDH2B4
MAARRISSLLSRSLSAPSAPSASTPLLLSRGKNPGRGRGVCSYTQHLINGQFVDAASGKTFPTHDPRTGEVIAHVAEGDAEDVNRAVAAARKAFDEG
PWPKMSAYERSLIMLRFADLVDKHRGELAALESWNSGKPYEQSAKSELPSFARLFRYYAGWADKIHGLTVPADGNHHVQTLHEPIGVAGQIIPWNFP
LIMFAWKVGPALACGNTIVLKSAEQTPLTALYAAKLFQEAGLPPGVLNVVSGYGPSAGAALACHMDVDKIAFTGSTETGKIILELAAKSNLKAVTLE
LGGKSPFIVCEDADVDKAVELAHFALFFNQGQCCCAGSRTYVHERVYDEFVEKAKARALRRVVGDPFKKGVEQGPQIDSEQFEKILRYIKSGVESNA
TLECGGQRFGSKGYFIQPTVFSNVQDDMLIAKDEIFGPVQSILKFKNIDEVIQRANTTRYGLAAGIFTKNVDTANTLSRALRVGSVWVNCFDVFDAA
IPFGGYKMSGIGREKGIYSLHNYLQVKAVVTPLKNPAWL
>PtALDH2B7
MAAKKISTVLSRSFSSAAAPPCFFSRVRGGGGQSRLISRYNTTLAAVEDPITPPVSVKYNQLLINGQFVDAASGKTFPTLDPRTGEVIAHVAEGDVE
DVNRAVSAARKAFDEGPWPRMTAYERSRIIWRFADLLEKHTDEIAALETWDNGKPYEQSAKIEIPMTVRIFRYYAGWADKIHGLTVPADGPYHVQTL
HEPIGVAGQIIPWNFPMLMFSWKVGPALACGNTVVIKTAEQTPLSAVYAAKLFHEAGLPDGVLNVVSGFGPTAGAALASHMDVDKLAFTGSTDTGKI
VLELASKSNLKPVTLELGGKSPFIVCEDADVDQAVELSHSAVFFNQGQCCCAGSRTFVHERVYDEFVEKAKARANQRAVGDPFKEGIEQGPQVDSDQ
FEKILRIIRSGVESGANLKAGGDRFGTTGYYIQPTVFSDVQDDMLIAKEEIFGPVQSIFKFKDLDEVIQRSNNSRYGLAAGIFTHNLDTANTLSRAL
KVGTVWINCYDVFDAAIPFGGYKMSGNGREKGIYSLNNYLQVKAVVTSLKNPAWL
>PtALDH2C4

MMKYADLIDEHIEELAALDAIDAGKLFSGGKAVDIPNVARLLRYYAGAADKIHGEVLKMSRELHGYTLREPIGVSGHIIPWNFPSSMFFMMSAPALA
AGCTMIVKPAEQTPLSALFYGHLAKQAGMPDGVINVITGYGPTAGAAIASHMDVDKVCFTGSTEVGRKIMQAAATSNLKQVSLELGGKSPLLIFDDA
DVDKAADLALLGILYNKGEICVASSRVFVQEGIYDEFVKKLKEKAKDWVVGDPFDPRSRLGPQVDKQQFDKILSYIEHGKREGASLLTGGKPVGKKG
YFIEPTVFTDVKEDMMIATDEIFGPVMSLMKFKTIDEAIKKANNTKYGLAAGIVTKNLDVANTVSRSIRAGTIWINCYFAFDNDCSYGGYKMSGFGR
HLGMEALHKFLQVKSVVTPIYNSPWL
>PtALDH3F1
MEGLEGTLAELRDTFKSGRTRSVAWRKSQLRAMIEFVQDNEEEMFKVLDQDLGKHPVEAYRDEVGVVAKSAKLSLSCVEKWMAPKKGNLPLAFFPAS
AEVMPEPFGVVLIMGSWNFPISLTLDPLIGAISAGNVVVLKPSELSPACSSFLAEAIPKYLDPKSIKVIEGGIDVCEQLLQQNWDKIFFTGSQRVGR
IVMTAAAQHLTPVTLELGGKSPAILDSSSNPTNMKVIAKRIVAAKWGSCSGQACIAIDYMLVEEKFASYLIDLLEKTIKQFFGENPRESKSLCKILN
KNNFMRLLDLLKDPLIRASVVYGGSVDEETMYIEPTILLNPPLDSQIMTEEIFGPLLPIITLNNIHDSIEFISSRPKPLAIYAFTRDETFKKQILSK
TSSGSVTFNDTLLQFVCDSLPFGGVGQSGFGRYHGKYSFDTFSHEKAILQRRFFPELEPRYPPWNNLKFQFIKLLYAFNYIGLLLLLLGLKK
>PtALDH3F2
MSSKKAKLPRVALLSSAELVPEPLGFVLIISSWNFPFGLSLEPMIGAIAAGNTMVLKPSELAPASASLLANVLPTYLDNSAVKVIQGGPAVGERLLQ
QKWDKIFFTGSARVGRIIMSAAVKHLTPVALELGGKCPAVVDSVSSSWDTKVTVNRILVSKFGACAGQACIAIDYILVEKRFASILVELMKVMIKKM
FGENPRETNTVARIVNEQHFLRLKNLLSDSAVQNSIVYGGSMDEKNLFVEPTILVDPPLDAAIMTEEIFGPLLPIITLDKVEDSIAFINSKPKPLAI
YAFTNNEKFRRRMLSETSSGSLVFNDAVIQYAADALPFGGIGESGIGKYHGKFSFDTFSHYKAVTRRSFLTDFWFRFPPWNDYKLLLLEATYNYDYL
GMLLVILGLKRRR
>PtALDH3H1
MATEEEKQMVFDVEAANMLTKELRDVFASGKTRSYEWRISQLKSMIKMCDEHEEDIVDALHQDLSKPKLESIVYEITMLKNSCTLAIKELKQWMMPE
KAKTSLLTFPSSAEIVPEPLGVVLIISAWNYPFLLSLDPLVGAIAAGNAMVLKPSEFSPATSSLLAKLLPEYLDISSIKVVEGAVSETSALLEQKWD
KIFYTGNGIVGRIVMAAAAKHLTPVVLELGGKSPAVVDSAIDLQIATRRLIAGKWGCNNGQACVSPDYIITTKDCADKLVDSLKKELETFYGKNPLE
SKDLSRIVNSKHFSRLTKLLDEDKVSRKIVYGGERDEANLKISPTILVDVPCDSLIMKEEIFGPLLPILIVSKIEDSFDMINSGTKPLAAYLFTNNK
KLKEQFVMSVSAGGVVINDIAMHLAIHTLPFGGVGESGTGSYHGKFSFDAFSHKKAVLYRSFMGDAALRYPPYTRGKLRLMKAFMTSNFWTILRALF
GRS
>PtALDH3H2
MATKEEENTVFDVEAANVLTKELRDVFASGKTRSYEWRISQLKSIVKMCDEHEEDIVDALRQDLSKPQLESIVYELTMVKNSCTLAIKELKHWMMPE
KAKTSLTTFPSSAEIVSEPLGAVLIISAWNYPFLLSMDPLIGAIAAGNAMVLKPSEVAPATSSLLAKLLPEYLDCSSIKVVEGAVSETSALLEQKWD
KIFYTGNGRVGRIVMAAAAKHLTPVVLELGGKSPVVVDSGIDIQIATRRIIVGKWGCNNGQACISPDYIITTKDCAEKLVDSLKKELEAFYGKNPLE
SKDLSRIVNSNHFSRLTKLLDEDKVSGKIVYGGERDEANLRIAPTILLGVPQNSLIMKEEIFGPLLPILTVSKIEDSFDIIKSGTKPLAAYLFTNNK
KLKEQFLMSVSAGGVVINDTTLHLAVHSVPFGGVGESGMGSYHGKFSFDAFTHKKAVVYRSFVGDASVRYPPYTLGKLRLMKALITGNVWTILRTLL
GMS
>PtALDH3I1
MRSLCVEPFQNLSVVDTGARRAFTHCSPWKTNHKHEAVLSFPLSSPIRKSLCICLSSSANLPVMMEEKKQTFDANEAAWLVKELNESFRTGKTKSYE
WRVSQLKGIEKMVVEREKDICEALYKDLSKPEYEAFVSEIAMVKSSCEEALKELKQWMKPEKAKTSMATYPSSAEIVSEPLGAVLVISTWNYPFSLS
VKPVIGAITAGNAVVLKPSEIAPATSSLLSKLFEEYLDRSAVRVLEGGVLETTALLDQKWDKIFYTGSPRVGRIVMTAAAKHLTPVVLELGGKCPAV
IDSDVDLQVTVRRIIAGKWQLNNGQACISVDYIITTKEFAPKLIDALRKGIEEFFGTDPMESKDISCIVSSNHFSRLESLMDDYKVFNKIVVGGQRN
QKKLKIAPTIFLDVPGDSQLMQEEIFGPLLPIITVENVKDSIDLINSKPKPLTAYLFTNNEKLKNNFVQSVSSGGMVINDTVLHVTVSSLPFGGVGE
SGMGSYHGKFSFDAFSHKKAVLYRSFSGDASVRYPPYTPEKQKLIRAVMNGGIFDIILALMGF
>PtALDH3I2
MTEGKKQPFDANEAPSLVKELKESFRTGRTRSYEWRVSQLKGIEKMVEEREKDISEALYKDLSKPEFEAFVSEIAAVKSSCEEALKELKQWMKPEKA
KTSMTAYPSSAEIVSEPLGAVLVISTWNYPFLLSIDPVIGAIAAGNAVVLKPSEIAPVTSSLLSELFEEYLDSSAVRVVEGAVPETAALLEQKWDKI
FYTGSPRVGRIVMTAAAKHLTPVVLELGGKCPVVVDSDVDLQVTARRIIAGKWQLNNGQACISVDYIIATKDFAPKLIDALRNGIEEFFGADPMESK
YISRIVSSNHFSRLERLLDEYKVFNKIVVGGQRNQKKLKIAPTIFLDVPEDSQLMQEEIFGPLLPIITVENVKDSIDLINSKPEPLVAYLFTNNQKL
RNDFVQNVSCGGMVINDTVLHVTVSSLPFGGVGESGMGSYHGKFSFDAFSHKKAVLYRSFSGDSPVRYPPYTPENKKLMRAVMNGGIFDIILALMGW
SRD
>PtALDH5F1
MTLGRIALARIPATRCKIHSLFSAPHSSSSTNRTSPPLSRHMSMKSENLVSKLTSSGLLKTQGLIDGKWVDANDGDTIKVLNPATGEVVAIVPCMGQ
SETNNAISSAYDAFRSWSKLTASERSQRIRKWYDLLIAHKEELGQLITLEQGKPLKEAMGEVSYGASFIEFYAEEAKRVYGDIIPATLGDRRLFVLK
QPVGVVGAITPWNFPLAMITRKVGPALACGCTVVLKPSELTPLTALAAAELALQAGIPPGVLNVVMGKAPDIGDALLASHEVRKITFTGSTAVGKKL
MAGAAGTVKRLSLELGGNAPCIVFDDADLDVAVKGSLAAKFRNSGQTCVCANRIIVQEGIYDKFADSFSKAVQSMQVGDGFSEGVTQGPLINEAAVQ
KVESFVQDAIFKGAKVLLGGKRHSLGMNFYEPTIISNVTEAMLLSREEVFGPVAPLLRFKTEEEAILMANNTKAGLAAYIFTNNVQRSWRVTEALEY
GLVGVNEGLISTEVAPFGGVKQSGLGREGSKYGMDEYLEMKYVCLGDMNRK
>PtALDH6B1
MLLLRSSIQRARNLKALKPSIFALRSSYCFSTGAAEPSSSLPSPPRVPNLIGGKFVDSQSSSTIDVINPATQEAVSRVPFTTNEEFRAAVSAAKQAF
PAWRNTPITTRQRVMLKLQELIRRDIDKLAMNITTEQGKTLKDAHGDVFRGLEVVEHACGMATLQMGEYVPNVSNGIDTFSIREPLGVCAGICPFNF
PAMIPLWMFPVAVTCGNTFILKPSEKDPGASIILAELAMEAGLPDGVLNIVHGTNDVVNAICDDDDIRAISFVGSNTAGMHIYSRASAKGKRVQSNM
GAKNHAIVLPDANVDATLNALVAAGFGAAGQRCMALSTVVFVGDPESWENKLVERAKSLKVNSGMEPDADLGPVISKQAKERVCRLIQSGVESGARL
LLDGRNIVVPGFEHGNFIGPTILSGVTADMECYKEEIFGPVLLCMEAGSVEEAINILNRNKYGNGAAIFTASGAAARKFQTEIEAGQVGINVPIPVP
LPFFSFTGSKASFAGDLNFYGKAGVNFYTQIKTITQQWKDLPGGSGVSLAMPTSQKL
>PtALDH6B2
MLLLRSSIRRARNLKALKPSIFALRSSYCFSTGAAEPSSSLPSPPRVPNLIGGKFVDSQSSSTIDVINPATQEAVSPVPLTTNEEFRAAVSAAKQAF
PAWRNTPITTRQRVMLKLQELIRRDIDKLAMNITTEQGKTLKDAHGDVFRGLEVVEHACGMATLQMGEYVPNVSNGIDTFSIREPLGVCAGICPFNF
PAMIPLWMFPVAVTCGNTFILKPSEKDPGKDYLVYILCIIFGALSLQGTSVC
>PtALDH6B3
MLPLKISIQRARNLKALKPSIFALRSSYYFSTGVVEPSSSLRSPPRVPNLIGGKFVDSQSSSTIDVINPATQEVVSQIPLTTNEEFKAAVSAAKHAF
PAWRNTPITTRQRVMLKLQELIRRDTDKLAMNITTEQGKTLKDAHGDVFRGLEVVEHACGMATLQMGEYVPNVANGIDTFSIREPLGVCAGICPFNF

PAMIPLWMFPVAVTCGNTFVLKPSEKDPGASIILAELAMEAGLPNGVLNIVHGTNDIVNAICDDDDIRAISFVGSNTAGMHIYSRASAKGKRVQSNM
GAKNHAIVLPDANTDATLNALVAAGFGAAGQRCMALSTVVFVGDSQSWENKLVECAKSLKVNAGTEPDADLGPVISKQAKERVCKLIESGVESGARL
LLDGRNIVVPGYEDGNFIGPTILSGVTADMDCYKEEIFGPVLLCKEADSFEEAIHFVNRNKYGNGAAIFTTSGAAARKFQTEIEAGQVGINVPIPVP
LPFFSFTGSKASFAGDLNFYGKAGVNFYTQIKTITQQWKDLPGGSGVSLAMPTSQKL
>PtALDH6B4
MTDIQSSSGSGLDEAQMQMQPPPPGTFVDREELIQHVGDFAVSQGYVVTIKQSKRERVVVLGCDRGGVYRNRKKADEETSAERKRRKRSGSRLTNCP
FEAVGKKDDGLWVLTIKNGTHNHEPLKDITEHPSARRFSESEIVLIKEMTEAGLKPRQILKRLRQSNPELLSTPKHVYNVKAKLRQGNMTGRNFKSL
RPEKSAGRDKHLSIAEPSWRQRYPMRVPNFIGGRLVNSQSFASIDVINPATQQVVSQVPLTTNEEFRAAVFAAKRAFPQWRDTPITTRQRIMFKFQE
LIRRDIDKLAMSITTEHGKTLKDAHGDVLRGLEVVEHACGLASLQIGEFVSNISSGIDTYSIREPLGVCAGICPFEFPAMIPLWIFPIAVTCGNTFI
LKPSEKDPGASVMLAELAMEAGLPNGVLNIVHGTNEIINGICDDDDIKAISFVGPNAVGAYVYARASAKGKRTQSNIGAKNHAVVMPDASVGATINA
LVAAGFGGAGQKCMALNMAVFVGGLGPWEEKLVEHAKALKVTSGTEPDAELGPVISKQEKERIITLIQTGVESGAKLVLDGRNIVVAGYENGNFIGP
TILSDVTVNMECYKEDIFGPVLLCMQADSIEEAINIVNGNKYSNGASIFTTSGVAARKFQTEVEVGQVGINVPISVPLPFSSFISAKPSFAGDVSFD
GKAGIQFYTQVKTVTQQWRDLVSDDSSSHQLPSS
>PtALDH6B5
MWVLNIKNGEHNHEPLKDMSEHPYSRRFSEEEVRQIRMMTEAGVKPRQVLKALKQSNPELQSTPRHLYNLKAKIRQGGLSDRSLKSWRPNRSVLVNT
SASSTGESLKEDRQPMKVPNFIGGKFVVSQGCTIIDVLNPATQEVVSHLPLTTYEEFKDAVIAAKRAFPSWKNMPIATRQRVMFRFQELIRRDMDKL
ATSITSEQGKTLKGALGDVLCGLEAVEHACAMATLQMGEFVPNASNGIDTYCIREPLGVCAGICPFNFPAMIPLWMFPIAVTCGNTFVLKPCEKNPG
ASMILAALAVEAGFPDGVLNVIHGTNDIVNYICDDDDVKAISFIGSDLAGLHIYARAAARGKRVQSNIGGKNHAIILPDASIDDTLNALVAAGFGAA
GQRCMALSTAVFVGGSSAWEHELVEHAKALKVNAGTDPSADLGPVISKEVKDRICRLVQSGVDSGARLLLDGRNIVVPGYENGSFVGPTILCDVTIS
MECYKEEILGPVLLCMQADSLEEAITIVNRNRYGNGASIFTTSGVAARKFQNDIDAVLVGINVSVPVPLPCSSFHEAKVSFAGNLNFCGKTGVQFYT
QIKTVAQQWRELPSIGVSLSMHTSNEMEMTSRGVCSALPPSERDSPGKTVSPAMSLAPERDPQKHRELLCENLPKSGGSSVPSITDKDLHNQEASLV
LPPTAEKDLQAKIPPTIPHASEIKLSSQEISLTTCQTSEGMYIPVPSQWNETPTLTSQRTESISQISQRIYLPTSQRRNNAAPSLKRIDAAMDLTSE
CVYMATPRQNDNTGPALLKDSSPSPTSRPTDTAAHPASERLHDITTSHLSDSMVQSFQRNDHMFPTERKYTSAAAHRNDHIGLTSQRPDVASYPSSE
RVYSSATSQRTDNMIPASQRAEAMPPTTKTMYMPPIVQRNNGPQKTSERLFMYQSERMYSESTLISIDGFSSQGVSMTLATSQRM
>PtALDH7B3
MSFARKEYEFLSEIGLSSRNLGCYVDGTWKANGPVVTSVNPANNQAIAEVVEGSVEDYEEGMRACSEAAKIWMQVPSPKRGEIVRQIGDALRTKLQE
LGRLVSLEMGKILPEGIGEVQEIIDMCDFCVGLSRQLNGSVIPSERPNHAMLEMWNPLGIVGVITAFNFPCAVLGWNACIALVCGNCVVWKGAPTTP
LITIAMTRLVAGVLEKNNLPPAIFTSFCGGADIGQAIAKDTRISLVSFTGSSKVGLMLQQTVNQRFGKCLLELSGNNAIIIMDDADIQLAVHSVLFA
AVGTAGQRCTTCRRLLLHESIYQRVLDQLLDVYKQVKIGNPLEKGNLLGPLHTSESRKSFERGIEIIKSQACKILIGGSVIESEGNFVQPTIVEISP
NADVVKEELFAPVLYVMKFQTLQEAIEINNSVPQGLSSSIFTRKPEIIFKWIGPLGSDCGIVNVNIPTNGAEIGGAFGGEKATGGGREAGSDSWKQY
MRRSTCTINYGNELPLAQGINFG
>PtALDH7B4
MGFARKEYEFLSEIGLSSRNLGCYVDGTWKANGPVVTSVNPANNQAIAEVVEGSIEDYEEGMRACSEAAKIWMQVPSPKRGEIVRQIGDALRTKLQQ
LGRLVSLEMGKILPEGIGEVQEIIDMCDFSVGLSRQLNGSVIPSERPNHAMLEMWNPLGIVGVITAFNFPCAVLGWNACIALVCGNCVVWKGAPTTP
LITIAMTRLVAGVLEKNNLPPAIFTSFCGGADIGQAVAKDTRIPLVSFTGSSKVGLMVQQIVNQRFGKCLLELSGNNAIIVMDDANIQLAVRSVMFA
AVGTAGQRCTTCRRLLLHESIYQRVLDQLLDVYKQVKIGDPLEKGTLLGPLHTSESRKSFEKGIEIIKSQGGKIITGGSVIESEGNFVQPTIVEISP
NADVVKEELFAPVLYVMKFQTLQEAIEINNSVPQGLSSSIFTRQPGVIFKWIGPQGSDCGIVNVNIPTNGAEIGGAFGGEKATGGGREAGSDSWKQY
MRRSTCTINYGNELPLAQGINFG
>PtALDH10A8
MAIHLPNRQLFIDGEWRETVLKKRIPVINPATEQIIGDIPAATAEDVEIAVEAAKKAFSRNKGKDWSSASGAYRARYLRAIAAKITERKSELGKLEA
IDSGKPLDEALWDMDDVAGCFEYYADLAEGLDTKQKAPVSLPMETFKSFVLKEPLGVVALITPWNYPLLLATWKVAPALAAGCTAILKPSELASVTC
LELGEVCREVGLPPGVLNILTGLGTEAGAPLASHPHVDKVAFTGSTATGSRIMASAAQMVKPVSMELGGKSPIIVFEDVDLDKAAEWTLFGCFWTNG
QICSATSRLLVHESIASEFLDKLVKWTKKIKISDPFEEGCRLGPLVSGEQYDKILKFIATAKSEGATILSGGDRPKHLNKGFFVEPTIIIDVTTSMQ
IWREEVFGPVLCVKTFSTEDEAIDLANDTHYGLGAAVISNDPERCDRVAKAFRAGIVWINCSQPCFCQAPWGGIKRSGFGRELGEWGLENYLSVKQV
TRYISEEPWGWYQAPSKL
>PtALDH10A9
MAIHLPIRQLFIDGEWREPVLKKRIPVINPATEQIVGDIPAATAEDVEIAVEAARKAFSRNKGQDWPSTSGAYRAKYLRAIAAKITEKKSELGKLEV
IDCGKPLDEALWDMDDVAGCFEYYADLAEGLDAKQKAPVSLPMETFKSYVLKEPLGVVALITPWNYPLLMGAWKVAPALAAGCTAILKPSELASVTC
LELAEVCREVGLPPGVLNILTGLGTEAGAPLASHPHVDKVAFTGSSATGSKIMASAAQMVKPVSMELGGKSPIIVFEDVDLDKAVEWTLFGCFWTNG
QICSATSRLLVHESIASEFLDRLVKWTKKIKISDPFEEGCRLGPVVSGGQYEKVLEFIATARSEGATILSGGDRPKHFTKGFFVEPTIITDVTTSMQ
IWREEVFGPVLCVKTFSTEDEAIELANDTHYGLGAAVISNDLERCDRVTKVRHELTSNLVQITHQNLLIGIVYLLRQYFSFAPVLQAFRAGIVWINC
SQPCFCQAPWGGIKRSGFGRELGEWGLENYLSVKQVTQYISDEPWGWYQSPAKL
>PtALDH11A1
MAGTGMFSEILDGDLYKYYSDGEWKKSSSGKTVSIVNPTTRKTQYKVQACTQEEVNKVMESAKSAQKAWAKTPLWKRAELLHKAAAILKEHKAPIAE
CLIKEIAKPAKDSVTEVVRSGDLISYTAEEGVRILGEGKFLVSDSFPGNDRTKYCLTSKIPLGVVLAIPPFNYPVNLAVSKIGPALIAGNSLVLKPP
TQGAVSCLHMVHCFHLAGFPKGLISCVTGKGSEIGDFLTMHPGVNCISFTGGDTGIAISKKAGMIPLQMELGGKDACIVLEDADLDLVAANIIKGGF
SYSGQRCTAIKVVLVMESVADALVEKVKARVAKLRVGPPENDCDITPVVTESSANFIEGLVMDAKEKGATFCQQYKREGNLIWPLLLDNVRPDMRIA
WEEPFGPILPVVRINSVEEGIYHCNASNFGLQGCVFTKDINRAMLISDAMETGTVQINSAPARGPDHFPFQGLKDSGIGSQGITNSINMMTKVKTTV
INLPSPSYTMGYC
>PtALDH11A2
MAGTGVFSEILDGDAYKYYSDGEWKKSSSGKTVAIVNPTTRKTQYKVQACNQEEVNKVMELAKSAQKTWAKTPLWKRAELLHKAAAILKEHKAPIAE
CLIKEIAKPAKDSVTEVVRSGDLISYTAEEGVRILGEGKFLVSDSFPGNERTKYCLTSKIPLGVVLAIPPFNYPVNLAVSKIGPALIAGNSLVLKPP
TQGAVSCLHMVHCFHLAGFPKGLISCVTGKGSEIGDFLTMHPGVNCISFTGGDTGISISKKAGMIPLQMELGGKDACIVLEDADLDLVAANIIKGGF
SYSGQRCTAIKVVLVMESVADALVEKVKARVAKLRVGPPEDDCDITPVVTESSANFIEGLVTDAKEKGATFCQQYKREGNLIWPLLLDNVRPDMRIA
WEEPFGPILPVIRINSVEEGIHHCNASNFGLQGCVFTKDINKAVLISDAMETGTVQINSAPARGPDHFPFQGLKDSGIGSQGITNSIDMMTKVKTTV
INLPSPSYSMGSSGSSIRSRI

>PtALDH11A3
MAGTSVFAELVDEDTTVFKFYSDGEWKKSTSGKLVSIINPTTRKTQYKVQACTQEEVNKIIEAAKTAQKSWAKTPLWKRAELLHKAAAILKEHRAPI
AECLVKEIAKPAKDAVTEVVRSGDLVSYCAEEGVRILGEGKFLVSDSFPGNERTKYCLTSKIPLGVVLAIPPFNYPVNLAVSKIAPALIAGNSIVLK
PPTQGAVAALHMVHCFHLAGFPKGLVSCVTGKGSEIGDFLTMHPGVSCISFTGGDTGIAISKKAGMIPLQMELGGKDACIILEDGDLDLAAANIIKG
GFSYSGQRCTAVKVILIMESVADTLVEKVKAKVAKLTVGPPEDDCDITPVVTESSANFIEGLVMDAKQKGATFCQEYKREGNLIWPLLLDNVRPDMR
IAWEEPFGPVLPVIRINSIEEAIYHSNASNFGLQGCIFTRDINKAILISDAMETGTVQINSAPARGPDHFPFQGLKDSGIGSQGITNSINMMTKIKS
TVINLPAPSYAMG
>PtALDH12A1
MMYGFLVCRASQKATRNWLSSFNLSRSVHSLPFATVDAEGISGSQPAKVHNLVQGKWIGSSTWNTIVDPLNGEPFIKIAEVDETGTQPFVESLSKCP
KHGLHNPFKSPERYLLYGDITAKAAHMLAVPKVSDFFTRLIQRVAPKSYQQALGEVQVTQKFLENFSGDQVRFLARSFAVPGNHLGQQSHGFRWPYG
PVAIITPFNFPLEIPLLQLMGALYMGNKPILKVDSKVCIVMEQMIRLLHHCGMPLSDVDFINSDGKTMNKLLLEANPQMTLFTGSSKVAEKLAVDLK
GRIKLEDAGFDWKILGPDVNEVDYIAWVCDQDAYACSGQKCSAQSILFMHENWSATSLISKMKDLAERRKLEDLTIGPVLTLTTEAMLDHMNKLLQI
PGSKLLFGGKPLENHSIPSIYGALKPTAIYVPLEEILRAKNYELVTREIFGPFQVITEYKKDQLPMVLDALERMHAHLTAAVVSNDVLFLQARLENI
FASHPTPLPIEVIGKTVNGTTYAGLRARTTGAPQNHWFGPAGDPRGAGIGTPEAIKLVWSCHREVIYDFGPLPKLWEIPPST
>PtALDH18B1
MSDLDRSRAFFNDVKRLIIKVGTAVVTRADGRLALGRLGALCEQIKDLSSLGYEVIVVTSGAVGLGRQRLKYRRFVNSSFSDLQKPQVDLDGKACAA
VGQNSLMALYDTMFSQLDVTSAQLLVTDRDFKNKDFRKQLDETVQSLLALRVIPIFNENDAVSTRKAPYEDSSGIFWDNDSLAALLALELKADLLVL
LSDVEGLYSGPPSDPRSKLIHTYIKEIHQSEITFGDKSRVGRGGMTAKVKAAVNAAYAGIPVVITSGYAPENIIKVLQGERVGTLFHQDAHLWALDK
EVGGREMAVAARESSRRLQALSSQDRNKILLDVADALEANEKLINIENEADVVAAQEAGLEKSLISRLALKPGKIKSLANTIRVLANMDDPIGCILK
RNELADGLVLEKTSSPLGVLLIIFESRPDALVQIASLAIRSGNGLLLKGGKEAKRSNAILHKVITTAIPDTVGGKLIGLVTSIDEIPDLLKLDDVID
LVIPRGSSKLVSKIKSSTKIPVLGHADGICHVYVDKSANIEMAKRVVLDAKVDYPAACNAMETLLVHQDLVHSGGLNELIADLRTEGVTLFGGQRAC
KELNIPEAHTFHHEYNSMACTVEIVDDEHAAIDHIHQHGSAHTDCIVAEDHDVAEVFLRQVDSAAVFHNASTRFCDGARFGLGAEVGISTSRIHARG
PVGVEGLLTTRWILRGCGQVVNADQGVIYTHKDITM
>PtALDH18B2
MNGTDPSRGFFKDVKRLIIKVGTAVVTRTDGRLALGRLGALCEQIKDLNSLGYEVIVVTSGAVGLGRQRLKYRRLVNSSFADLQKPQVDFDGKACAA
VGQNNLMALYDTLFSQLDVTSAQLLVTDSDFRDKGFRKQLDQTVKSLLALRVIPIFNENDAVSTRRAPYEDSSGIFWDNDSLAALLALELKADLLVL
LSDVEGLYSGPPSDPQSKLIHTYIKEIHQSEITFGDKSRVGRGGMTAKVKAAVNAAYAGIPVVITSGYAPENIMKVLQGERVGTLFHQDAHLWVPVK
EVSGREMAVAARESSRRLQALSSQDRKKILLGVADALEANEKLIKIENEADVAAAQQAGLEKSLISRLALKPGKIESLANSIRVLANMEDPIGRVLK
RTELADGLLLEKTSSPLGVLLIVFESRPDALVQIASLAIRSGNGLLLKGGKEAKRSNAILHKVITTAIPDTVGGRLIGLVTSRDEIPDLLKLDDVID
LVIPRGSNKLVSQIKSSTKIPVLGHADGICHVYMDKSANMEMAKRVVLDAKIDYPAACNAMETLLVHQDLVQTAGLNELIVDLRTEGVTLFGGQRAC
KELNLPEAHSLHHEYNSMACTVEIVDDVHAAINHIHQHGSAHTDCIIAEDQDVAEVFLCQVDSAAVFHNASTRFCDGARFGLGAEVGISTSRIHARG
PVGVEGLLTTKWILRGSGQVVNGDKGVIYTHKDMTLQSVD
>PtALDH22A1
MAFWWPLIVAASAYAICRFLLMLIPFNVPSIDVDASDVTEGNQTQENSFIYIPPRGRAQQSDKKVQCYEPATMKYLGFFPALSPAEVHDRVAQARKA
QKIWAESSFKQRRQFLRILLKYIIEHQELICEVSSRDTGKTMVDASLGEIMTTCEKITWLLSEGEKWLKPEYRCSGRAMFYKKSRVEFHPLGVIGAI
VSWNYPFHNIFNPMLAAVFSGNSIVIKVSENASWSGLFYFRIIQAALAAVGAPENLVDVITGFAETGEALVSSVDKIIFVGSPGVGKMIMRNASDTL
IPVTLELGGKDPFIVCEDADVSHVAQIAVRAVLQSSGQNCAGAERFYVHRDIYSSFVSEVTKIVKSVSVGPPLAGRYDMGAICLQEHSDKLQILVND
ALEKGAEIVVRGSFGHLGEGAVDQFYPPTVLVNVDHTMKLMQEETFGPIMPIMKFSTDEEAVKLANDSRYGLGCAVFSGSQRRAREIASQIHCGVAA
VNDFASNYMCQSLPFGGVKHSGFGRFAGVEGLRACCLVKSVVEDRLWPYIKTKIPKPIQYPVGENSFEFQQSLVEALYGLNIRDKLRAGVNVLKIMS
EQNSSNSKSRNE
>ZmALDH2B2
MARRAASSLVSRCLLARAPAGAPPAAPSAPRRTVPADGMHRLLPGVLQRFSTAAAVEEPITPSVHVNYTKLLINGNFVDSASGKTFPTLDPRTGEVI
AHVAEGDAEDINRAVAARKAFDEGPWPKMTAYERSRILLRFADLIEKHNDELAALETWDNGKPYEQAAQIEVPMVARLMRYYAGWADKIHGLIVPA
DGPHHVQILHEPIGVAGQIIPWNFPLLMYAWKVGPALACGNTLVLKTAEQTPLSALYISKLLHEAGLPEGVVNVVSGFGPTAGAALASHMDVDKIAF
TGSTDTGKIILELAAKSNLKTVTLELGGKSPFIIMDDADVDHAVELAHFALFFNQGQCCCAGSRTFVHERVYDEFVEKAKARALKRVVGDPFRKGVE
QGPQIDDEQFNKILRYIRYGVDGGATLVTGGDRLGDKGFYIQPTIFSDVQDGMKIAQEEIFGPVQSILKFKDLNEVIKRANASQYGLAAGVFTNSLD
TANTLTRALRAGTVWVNCFDVFDAAIPFGGYKMSGIGREKGVDSLKNYLQVKAVVTPIKNAAWL
>ZmALDH2B5
MAATVRRAASSVLSRFLLTKPSPSPASAAGNKSALLGAGAAALHRFSTAPASAAAAAEEPIQPAVEVKHTQLLINGNFVDAASGKTFPTLDPRTGEV
IARVAEGDSEDIDRAVAAARRAFDEGPWPRMTAYERCRVLLRFADLIERHAEEVAALETWDNGKTLAQAAGAEVPMVARCVRYYAGWADKIHGLVAP
ADGAHHVQVLHEPVGVAGQIIPWNFPLLMFAWKVGPALACGNTVVLKTAEQTPLSALYVANLLHEAGLPEGVLNVVSGFGPTAGAALCSHMGVDKLA
FTGSTGTGQIVLELAARSNLKPVTLELGGKSPFIVMDDADVDQAVELAHQAVFFNQGQCCCAGSRTFVHERVYDEFVEKSKARALKRVVGDPFRDGV
EQGPQIDGEQFNKILRYVQSGVDSGATLVAGGDRVGDRGFYIQPTVFADAKDEMKIAREEIFGPVQTILKFSGVEEVIRRANATPYGLAAGVFTRSL
DAANTLSRALRAGTVWVNCYDVFDATIPFGGYKMSGVGREKGIYALRNYLQTKAVVTPIKNPAWL
>ZmALDH2C1
MATANGSSKGPFEVPKVEVRFTKLFIDGKFVDAVSGKTFETRDPRTGEVIASIAEGGKADVDLAVKAAREAFDNGPWPRMTGYERGRILHRFADLID
EHVEELAALDTVDAGKLFAVGKARDIPGAAHLLRYYAGAADKVHGATLKMAQRMHGYTLKEPVGVVGHIVPWNYPTTMFFFKVGPALAAGCAVVVKP
AEQTPLSALFYAHLAREAGVPAGVLNVVPGFGPTAGAAVAAHMDVDKVSFTGSTEVGRLVMRAAAESNLKPVSLELGGKSPVIVFDDADLDMAVNLV
NFATYTNKGEICVAGTRIYVQEGIYDEFVKKAAELASKSVVGDPFNPSVSQGPQVDKDQYEKVLRYIDIGKREGATLVTGGKPCGDKGYYIEPTIFT
DVKDDMTIAQDEIFGPVMALMKFKTVEEVIQKANNTRYGLAAGIVTKNIDVANTVSRSIRAGAIWINCYFAFDPDAPFGGYKMSGFGKDMGMDALDK
YLQTKTVVTPLYNTPWL
>ZmALDH2C2
MASNGCNGNGNGNGNGKAAPAGVVVPEIKFTKLFINGEFVDAASGKTFDTRDPRTGDVLAHVAEADKADVDLAVKSARDAFEHGKWPRMSGYERGRI
MSKLADLVEQHTEELAALDGADAGKLLLLGKIIDIPAATQMLRYYAGAADKIHGDVLRVSGRYQGYTLKEPIGVVGVIIPWNFPTMMFFLKVSPALA
AGCTVVVKPAEQTPLSALYYAHLAKMAGVPDGVINVVPGFGPTAGAALASHMDVDSVAFTGSTEVGRLIMESAARSNLKTVSLELGGKSPLIIFDDA
DVDMAVNLSRLAVFFNKGEVCVAGSRVYVQEGIYDEFVKKAVEAARSWKVGDPFDVTSNMGPQVDKDQFERVLKYIEHGKSEGATLLTGGKPAADKG

YYIEPTIFVDVTEDMKIAQEEIFGPVMSLMKFKTVDEVIEKANCTRYGLAAGIVTKSLDVANRVSRSVRAGTVWVNCYFAFDPDAPFGGYKMSGFGR
DQGLAAMDKYLQVKSVITALPDSPWY
>ZmALDH2C4
MASNGNGDGTARVVVPEIKFTKLFINGEFVDAASGKTFETRDPRTGDVLAHVAEADQADVDLAVKSARDAFDHGKWPRMSGYERGRVMSKLADLVEQ
HTEELAALDGADAGKLLLLGKMIDIPAATQMLRYYAGAADKIHGDVLRVSGKYQGYTLKEPIGVRRYLSS
>ZmALDH2C5
MVSESNRGGADRTTAAGEERGQLLFDVPEIRFTKLFINGSFVDAVSGRTFETRDPRTGGVIASVAEADKEDVDLAVRAARAAFDHGEWPRMSGSERG
RIMARLADLVEERADELAALESLDAGKHPAVTRAVDVGNAAGSLRYFAGAADKIHGETLKMPGQFQGHTLREPLGVAGVIIPWNFPSTMFAVKVAPA
LAAGCALVVKPAEQTPLSALYLAQLAKQAGVPDGVINVVPGFGPTAGAALASHMDVDMVSFTGSTEVGRLIMKASAESNLKPVYLELGGKSPLIVFD
DADLDMAVELAVGASFFNKGEACVAASRVYVQERVYDRFEERLAERMRSWVVGDPFSDPSADQGPQVDKAQYERVLSYIDHGKREGATLLTGGRPCG
PEGKGYYIEPTVFTNVKEDMIIAKEEIFGPVMCLMKFKTVEEAIARANDTRYGLGAGVVTRDLDVANRVVRSVRAGVVWVNCYFAMGSDCPFGGRKM
SGFGKDEGMHALDKYLAVKSVVTPLRASPWI
>ZmALDH3E1
MGSVPEEKAKLGFGGLVGDLREVYESGRTQGLEWRQSQLRGLVRLLEEKEEEIFDVLHEDLGKHRGEAFRDEVGVLKKSVVDKLQNLKNWAAPEKAH
TPLVAFPATALVVPEPLGVVLVFSCWNLPIGLALEPLSGALAAGNAVVVKPSELAPATSAFLAANIPKYLDSKAVKVVEGGPEVGEKLMEHRWDKVL
FTGSSRVGRLIMAQAAKHLTPVALELGSKCPCIVDWLDSDRDSQVAVNRIIGAKWSTCSGQACIAIDYLLVEEEFAPILIEMLKSTLERFFTKPEYM
ARILNEKQFQRLSGFLADRRVASSVVHGGHFNPKTLSMEPTLLLNPPLDSDIMTEEIFGPLLPIITVKKIEDSIKFLRSKPKPLAIYAFTRNEKLKQ
RIIDETSSGSITFNDAIVQYGLDSIPFGGVGHSGFGQYHGKYSFDMFSHKKAVLKRSFLVEFMFRYPPWDETKIGMLRRVYRFDYVSLFLALIGLRR
>ZmALDH3E2
MGRTEAADDGAESGGLGLGVGVGVGETVRELREAYESGRTRSLAWRQAQLRGLLRLLEEKEVEAFQALHKDLGKHHAEAYRDEVGVLIKSANGALQQ
LGKWMAPEKVRVPLIAWPATAQVVPEPLGVVLVFSCWNVPLGLSLEPLIGAIAAGNAVALKPSELSPCTARFLGDNIGRYMDSSAVKVVQGGPDVGV
QLMEHRWDKVLFTGSPRIARAVMAAASRHLTPVALELGGKCPCIFDAMGSARDLQISVNRMIAGKWSSCAGQACIAIDYVLVEERFAPILIKVLKST
LKRFFPEADHMARIVNERHFERLSNLLKDRSVAPSVLHGGSMDSKNLYIEPTILLNPPLDSAIMTEEIFGPLLPIITVKNIEDSIAFVKAMPKPLAI
YAFTRDAALRRRIVDETSSGSVTFNDAVVQYAIDGLPFGGVGQSGFGQYHGKYSFEMFSHKKAVMKRGYLVELTLRYPPWDESKVTLMRYLYRFNYF
AFVLSFLGLRR
>ZmALDH3H1
MDAEAAAAAAAATAVEERERLRRSFASGRTRPAAWREAQLRGLLRMATEREDDICAALHADLAKPLTECYVHEISLVISSCKFALKNLKKWMKPRKVP
GGLLTFPSAASVVAAEPLGVVLVISAWNYPFLLAIDPVVGAFAAGNAVALKPSEVAPATSLLLADLLPRYVDPSCVRVVQGGIAETTALLELQWDKIF
YTGNSRVGRIVMSYAAKHLTPVVLELGGKCPVVVDSDVNLHVAAKRIAAGKWGCNSGQACVSPDYVVTTKSFAPKLLESLKRVLFEFYGEEPLRSPD
LSRVVNSNHFNRLMALMDDYSVSGNVAFGGQIDERRLRIAPTLLLDVPLDSAMMKEEIFGPLLPIITVDKIGESFAVINSMPKPLAAYLFSNDGQLK
QQFERTVSAGGIMFNDTGIHLTNPNLPFGGVGESGMGAYHGAFSFDAFSHRKAVLDRSFLGEARARYPPYTPAKLAILRGVLNGSPLATVQAAAGCT
GGASAD
>ZmALDH3H2
MAEETVRELRASFAAGQTRPAEWRAAQLKGLIRMIDEKEAEISAALHEDLAKPHMESFLHEISLTKSSCKFALKGLKNWMKPEKVPAAITTFPSSAQ
IVPEPLGVVLIISAWNYPFILSIDPVIGAIAAGNAVVLKPSEIAPATSSLLAKLLPEYVDNSCIKVVEGSVPETTALLEQRWDKIFYTGNGTVGRIV
MAAAAKHLTPVALELGGKSPVIVDSNVDLHVAAKRIVVGKWGCNNGQACIAPDYIITTKSFAPELVASFKRVLERFYGEDPLESADLSRIVNSKQFK
RLTNLIEEKRVADKIVYGGKADEKQLKISPTLLLDVPEDSEIMTGEIFGPLLPIVTVEKIEESFDLINAKPKPLAAYLFTKNRKLQEEFVASVPAGG
MLVNDTALHLTNPYMPFGGVGDSGMGCYHGKFGFDCFSHKKGVLIRGFGGEANARYPPYTTEKQKILRGLINGSFIALILALLGFPREKR
>ZmALDH3H3
MAEETVQELRASFASGRTRRAEWRAEQLKGLIRMIDEKEAEISAALHEDLAKPHMESYLHEISITRSSCKFALDGLKSWMKPEKIPAALTTFPSSAQ
IVPEPLGVVLIISAWNYPFILSIDPVIGAIAAGNAVVLKPSEIAPATSSLLAKLLPEYVDNSCIKVVEGGVAETTSLLEQRWDKIFYTGNGTVGRIV
MAAAAKHLTPVALELGGKSPVVVDSNVDLHVAVKRIVVGKWGCNNGQACIAPDYIITTKSFAPELVASLKRVLERFYGEDPLQSADLSRIVNSKHFR
RLTELIEEKSVADKIVYGGEVDEKQLKIAPTLLLDVPQDSAIMTGEIFGPLLPIVTVEKIEESFDLINARPKPLAAYLFTKNKKLQEEFVADVPAGG
MLVNDTVLHLANPYMPFGGVGDSGMGCYHGKFGFDCFSHKKGVLVRGFGGEANARYPPYTTEKQKILRGLINGSFIALILALLGFPREKR
>ZmALDH5F1
MATAMMTMRRAAALGARHIPPAAAFSRHMSADASAAMEKIRAAGLLKTQGLIAGQWVDAYDGKTIEVQNPATGEVLANVSFMGSRETSDAIASAHST
FYSWSKLTASERGKALRKWYDLIISHKEELALLMTLEQGKPMKEALGEVNYGASFIEYFAEEAKRIYGDIIPPTLSDRRLLVLKQPVGVVGAITPWN
FPLAMITRKVGPALACGCTVVVKPSEFTPLTSLAAADLALQAGIPAGALNVVMGNAPEIGDALLQSTQVRKITFTGSTAVGKKLMAESANTVKKVSL
ELGGNAPCIVFDDADIDVAVKGSLAAKFRNSGQTCVCANRILVQEGIYEKFASAFIKAVQSLKVGNGLEESTSQGPLINEAAVQKVEKFINDATSKG
ANVMLGGKRHSLGMSFYEPTVVGNVSNDMLLFREEVFGPVAPLIPFKTEEEAVHMANDTNAGLAAYIFTKSIPRSWRVSESLEYGLVGVNEGIISTE
VAPFGGVKQSGLGREGSKYGVDEYLELKYICMGNLG
>ZmALDH5F2
MAMAMMAMRRAVALGARHIPAAAASSFRVVSLRHMSADAGAAMEKIRAAGLLRTQGLIAGQWVDAYDGKTIEVQNPATGEVLANVSCMGSRETSDAI
ASAHSTFYSWSKLTASERSKALRKWYDLIISHKEELALLMTLEQGKPMKEALGEVNYGASFIEYFAEEAKRIYGDIIPPTLSDRRLLVLKQPVGVVG
AITPWNFPLAMITRKVGPALACGCTVVVKPSEFTPLTALAAADLALQAGIPAGALNVVMGNAAEIGDALLQSTQVRKITFTGSTAVGKKLMAGSANT
VKKVSLELGGNAPCIVFDDADIDVAVKGSLAAKFRNSGQTCVCANRILVQEGIYEKFAKAFIQAVQSLKVGNGLEESTSQGPLINEAAVQKVEKFIN
DATSKGANVMLGGKRHSLGMSFYEPTVVGNVSNDMLLFREEVFGPVAPLIPFKTEEEAVHMANDTNAGLAAYIFTKSIPRSWRVSESLEYGLVGVNE
GIISTEVAPFGGVKQSGLGREGSKYGIDEYLELKYICMGNLG
>ZmALDH6B1
MLRSALFRSAPGLRRSPATAHLSTAAAAAAAWLSNGPASAPSRVRLLIGGEFVESRADEHVDVTNPATQEVVSRIPLTTADEFKAAVDAARTAFPGW
RNTPVTTRQRVMFKFQELIRANMDKLAENITTEQGKTLKDAWGDVFRGLEVVEHACGMGTLQMGEYVSNVSNGIDTFSIREPLGVCAGICPFNFPAM
IPLWMFPIAVTCGNTFVLKPSEKDPGAAMMLAELAMEAGLPKGVLNIVHGTNDVVNNICDDEDIKAVSFVGSNTAGMHIYSRASAAGKRVQCNMGAK
NHAIILPDADRDATLNALIAAGFGAAGQRCMALSTAVFVGGSESWEDELVKRASGLVVSSGMVNDADLGPVISRQAKDRICKLVQSGVDLCARILLD
GRKIVVPYPVVY
>ZmALDH7B6
MGAFAKEEHQFLAELGLAQRNPGAFACGAWGGSGPTVTSTSPTNNQVIAEVVEASVHDYEEGMRACFDAAKTWMAIPAPKRGEIVRQIGDALRAKLH

HLGRLVSLEMGKILPEGIGEVQEIIDMCDYAVGLSRQLNGSIIPSERPNHMMMEVWNPLGVVGVITAFNFPCAVLGWNACIALVCGNCVVWKGAPTT
PLITIAMTKIVASVLEKNNLPGAIFTSFCGGTEIGQAIALDIRIPLVSFTGSTRAGLMVQQQVSARFGKCLLELSGNNAIIVMDDADIQLAVRSVLF
AAVGTAGQRCTTCRRLILHENIYQTFLDQLVEVYKQVRIGDPLEKGTLLGPLHTPASKENFLKGIQTIKSQGGKILFGGSAIESEGNFVQPTIVEIT
PSAPVVKEELFGPVLYVMKFQSLKEAIEINNSVPQGLSSSIFTKRPDIIFKWLGPHGSDCGIVNVNIPTNGAEIGGAFGGEKATGGGREAGSDSWKQ
YMRRATCTINYGSELPLAQGINFG
>ZmALDH10A5
MAPPQTIPRRGLFIGGAWREPCLGRRLPVVNPATEATIGDIPAGTAEDVEIAVAAARDAFSRDGGRHWSRAPGAVRANFLRAIAAKIKDRKSELALL
ETLDSGKPLDEASGDMDDVAACFEYYADLAEALDGKQQSPISLPMENFKSYVLKEPIGVVGLITPWNYPLLMATWKVAPALAAGCTTILKPSELASV
SCLELGAICMEIGLPPGVLNIITGLGPEAGAPLSSHSHVDKVAFTGSTETGKRIMISAAQMVKPVSLELGGKSPLIVFDDIGDIDKAVEWTMFGIFA
NAGQVCSATSRLLLHEKIAKKFLDRLVAWAKNIKVSDPLEEGCRLGSVISEGQYEKIKKFISTARSEGATILYGGGRPQHLRRGFFLEPTIITDVST
SMQIWQEEVFGPVICVKEFRTESEAVELANDTHYGLAGAVISNDQERCERISKALHSGIIWINCSQPCFVQAPWGGNKRSGFGRELGEWGLDNYLTV
KQVTKYCSDEPWGWYQPPSKL
>ZmALDH10A8
MASPAMVPLRQLFVDGEWRPPAQGRRLPVVNPTTEAHIGEIPAGTAEDVDAAVAAARAALKRNRGRDWARAPGAVRAKYLRAIAAKVIERKPELAKL
EALDCGKPYDEAAWDMDDVAGCFEYFADQAEALDKRQNSPVSLPMETFKCHLRREPIGVVGLITPWNYPLLMATWKIAPALAAGCTAVLKPSELASV
TCLELADICKEVGLPSGVLNIVTGLGPDAGAPLSAHPDVDKVAFTGSFETGKKIMASAAPMVKPVTLELGGKSPIVVFDDVDIDKAVEWTLFGCFWT
NGQICSATSRLLIHTKIAKKFNERMVAWAKNIKVSDPLEEGCRLGPVVSEGQYEKIKKFISNAKSQGATILTGGVRPAHLEKGFFIEPTIITDITTS
MEIWREEVFGPVLCVKEFSTEDEAIELANDTQYGLAGAVISGDRERCQRLSEEIDAGCIWVNCSQPCFCQAPWGGNKRSGFGRELGEGGIDNYLSVK
QVTEYISDEPWGWYQSPSKL
>ZmALDH10A9
MMASQAMVPLRQLFVDGEWRPPAQGRRLPVVNPTTEAHIGEIPAGTAEDVDAAVAAARAALKRNRGRDWARAPGAVRAKYLRAIAAKVIERKQELAK
LEALDCGKPYDEAAWDMDDVAGCFEYFADQAEALDKRQNSPVSLPMETFKCHLRREPIGVVGLITPWNYPLLMATWKVAPALAAGCAAVLKPSELAS
VTCLELADICKEVGLPPGVLNIVTGLGPDAGAPLSAHPDVDKVAFTGSFETGKKIMAAAAPMVKPVTLELGGKSPIVVFDDVDIDKAVEWTLFGCFW
TNGQICSATSRLLVHTKIAKEFNEKMVAWAKNIKVSDPLEEGCRLGPVVSEGQYEKIKKFILNAKSEGATILTGGVRPAHLEKGFFIEPTIITDITT
SMEIWREEVFGPVLCVKEFSTEDEAIELANDTQYGLAGAVISGDRERCQRLSEEIDAGIIWVNCSQPCFCQAPWGGNKRSGFGRELGEGGIDNYLSV
KQVTEYISDEPWGWYRSPSKL
>ZmALDH11A3
MALAGTGVFAEILDSEVYRYYADGEWRSSASGKSVAIVNPTTRKTQYRVQACTQEEVNKAMDAAKVAQKAWARTPLWKRAELLHKAAAILKEHKAPI
AECLVKEIAKPAKDAVSEVVRSGDLVSYTAEEGVRILGEGKLLVSDSFPGNERNKYCLSSKIPLGVVLAIPPFNYPVNLAVSKIGPALIAGNALVLK
PPTQGAVAALHMVHCFHLAGFPKGLISCVTGKGSEIGDFLTMHPGVNCISFTGGDTGIAISKKAGMVPLQMELGGKDACIVLEDADLDLVSANIVKG
GFSYSGQRCTAVKVVLIMESIADAVVQKVNAKLAKLKVGPPEDDSDITPVVTESSANFIEGLVMDAKEKGATFCQEYRREGNLIWPLLLDHVRPDMR
IAWEEPFGPVLPVIRINSVEEGIHHCNASNFGLQGCIFTRDINKAILISDAMETGTVQINSAPARGPDHFPFQGLKDSGIGSQGITNSINMMTKVKS
TVINLPSPSYTMG
>ZmALDH12A1
MSRLLSRQHLAAVRRSAPFACVSRWLHTPSFATVSPQEVSGSSPAEVQNFVQGSWTASANWNWIVDPLNGDKFIKVAEVQGTEIKPFVESLSKCPKH
GLHNPLKAPERYLMYGDISAKAAHMLGQPAVLDFFAKLIQRVSPKSYQQALAEVQVSQKFLENFCGDQVRFLARSFAVPGNHLGQRSNGYRWPYGPV
AIITPFNFPLEIPLLQLMGALYMGNKPVLKVDSKVSIVMEQMIRLLHDCGLPAEDMDFINSDGAVMNKLLLEANPKMTLFTGSSRVAEKLAADLKGR
VKLEDAGFDWKILGPDVQEVDYVAWVCDQDAYACSGQKCSAQSVLFMHKNWSSSGLLEKMKKLSERRKLEDLTIGPVLTVTTEAMIEHMNNLLKIRG
SKVLFGGEPLANHSIPKIYGAMKPTAVFVPLEEILKSGNFELVTKEIFGPFQVVTEYSEDQLELVLEACERMNAHLTAAVVSNDPLFLQDVLGRSVN
GTTYAGIRARTTGAPQNHWFGPAGDPRGAGIGTPEAIKLVWSCHREVIYDVGPVPESWALPSAT
>ZmALDH18B2
MGRGGIGGAAAMAMAMETADPARAFVKDVKRIIIKVGTAVVTGMNGRLAMGRLGSLCEQVKQLNFQGYEVILVTSGAVGVGRQRLQYRKLIHSSFAD
LQNPQMNFDGKACAAVGQSVLMAIYDTLFSQLDVTSSQLLVTDRDFKDPSFGDQLRETVFSLLDLKVVPLFNENDAISTRRQPYEDSSGIFWDNDSL
AALLAAELNADLLIMLSDVEGLYSGPPSDPQSKIIHTYVNEKHGKLISFGEKSSVGRGGMQAKVSAAANAASKGVPVVIASGFATDSIITVLKGEKI
GTLFHNEANLWACSKEATAREMAVAARDCSRRLQKLSSEERKQILLDIADALEANEDAIRSENDADVEAAQVAGYEKSLVARMTLKPGKITNLARSI
RKTADMEDPISHTLKRTEVAKDLVFEKAYCPLGVLLIIFESRPDALVQIASLAIRSGNGLLLKGGKEVMRSNAILHKVITGVIPDTVGKKLIGLVTS
KEEIADLLALDDVIDLVIPRGSKSLVSQIKATTKIPVLGHADGICHVYIDKSADMDMAKRIVLDAKIDYPAACNAMETLLVHKDLNKSEGLDDLLVE
LEKEGVVIYGGPVAHDKLKVPKVDSFRHEYSSMACTVEFVDDVQSAIDHINRYGSAHTDCIITTDRSAAEAFLQQVDSAAVFHNASTRFCDGTRFGL
GAEVGISTERIHARGPVGVDGLLTTRCILRGSGQVVNGDKGVVYTHKDLPLQ
>ZmALDH18B1
MATADRTRTFMKDVKRVIIKVGTAVVTRGDDGRLAVGRLGCLCEQVKELNVLGYEVILVTSGAVGVGKQRLKYRKLVNSSFADLQKPQMELDGKACA
AVGQSGLMALYDMLFTQLDVSSSQLLVTDSDFENPNFRERLCETVESLLDLKVVPIFNENDAISTRKAPYEDSSGIFWDNDSLAGLLAIELKADLLV
LLSDVDGLYSGPPSEPGSKIIHTYIKDKHYSGITFGDKSRVGRGGMTAKVKAAFVASNSGTPVVITSGFASQSIVRVLQGEKIGTLFHKDASLWEPS
KDVSAREMAVAARECSRRLQNLSSDERKKILLDIADALEQNEDLIRTENEADVSAAQDAGYQKSLVDRLTLKPEKIASLAKSIRTLANMEDPINQIL
KRTEVAEDLVLEKTSCPLGVLLIVFESRPDALVQIASLAVRSGNGLLLKGGKEAMRSNTVLHKVITGAIPDNVGQKLIGLVTSRDEIADLLKLDDVI
DLVIPRGSNKLVSQIKASTKIPVLGHADGICHVYIDKSADMNMAKRIVMDAKTDYPAACNAMETLLVHKDLIKAPGLDDILLSLKTEGVAIYGGPVA
HEVLCIPKADSFHHEYSSMACTIEFVDDVQSAINHIHRYGSAHTDCIITTDDKVAETFLRQVDSAAVFHNASTRFSDGARFGLGAEVGISTGRIHAR
GPVGVEGLLTTRWIMRGSGQVVNGDKNVAYTHKNLPLQ
>ZmALDH22A1
MAFWWPLLVLAAAYALCRLLLFLIPPTVPSIDVDASDVLAKEDSFIYIPRRGKSTQTDKVQCYEPATMKYLGYFPVVTPDEVKEHVAQSRKAQRIWA
KSSFKQRRQFLRILLKYILEHQDLICEVSSRDTGKTMVDASLGEIMTTCEKITWLLDEGEKWLKPEYRSTGRSMLHKRAKVEFYPLGVIGAIVSWNY
PFHNVFNPVLAAVFSGNAAVIKVSEHATWSGCFYFRIIQAALSAVGAPENLVHIITGFAETGQALVSSVDKIIFVGSPGVGKMIMKRASETLIPVTL
ELGGKDSFIVCEDVDLPSVVQVATRAALQSSGQNCAGAERFYVHDDIYSAFVSQIVKTVKSISVGPPLSGRYDMGAICMIEHSEKLQNLVNDALDKG
AEIAVRGSFGNLGEDAVDQFFPPTVLVNVDHTMKIMQEETFGPIIPIMKFSSDEEAIKLANDSKYGLGCAVFSGNQKRAIRIASQLHCGVAAINDFA
SSYMCQSLPFGGVKDSGFGRFAGVEGLRACCLVKSVVEDRLWPYIRTVIPKPIQYPVSEHGFEFQQLLVETLYGYSVWDRLRSLVNLIKMVTEQNFA
PTSNATTKKRR

```
>HsALDH1A1
MSSSGTPDLPVLLTDLKIQYTKIFINNEWHDSVSGKKFPVFNPATEEELCQVEEGDKEDVDKAVKAARQAFQIGSPWRTMDASERGRLLYKLADLIE
RDRLLLATMESMNGGKLYSNAYLNDLAGCIKTLRYCAGWADKIQGRTIPIDGNFFTYTRHEPIGVCGQIIPWNFPLVMLIWKIGPALSCGNTVVVKP
AEQTPLTALHVASLIKEAGFPPGVVNIVPGYGPTAGAAISSHMDIDKVAFTGSTEVGKLIKEAAGKSNLKRVTLELGGKSPCIVLADADLDNAVEFA
HHGVFYHQGQCCIAASRIFVEESIYDEFVRRSVERAKKYILGNPLTPGVTQGPQIDKEQYDKILDLIESGKKEGAKLECGGGPWGNKGYFVQPTVFS
NVTDEMRIAKEEIFGPVQQIMKFKSLDDVIKRANNTFYGLSAGVFTKDIDKAITISSALQAGTVWVNCYGVVSAQCPFGGFKMSGNGRELGEYGFHE
YTEVKTVTVKISQKNS
>HsALDH1A2
MTSSKIEMPGEVKADPAALMASLHLLPSPTPNLEIKYTKIFINNEWQNSESGRVFPVYNPATGEQVCEVQEADKADIDKAVQAARLAFSLGSVWRRM
DASERGRLLDKLADLVERDRAVLATMESLNGGKPFLQAFYVDLQGVIKTFRYYAGWADKIHGMTIPVDGDYFTFTRHEPIGVCGQIIPWNFPLLMFA
WKIAPALCCGNTVVIKPAEQTPLSALYMGALIKEAGFPPGVINILPGYGPTAGAAIASHIGIDKIAFTGSTEVGKLIQEAAGRSNLKRVTLELGGKS
PNIIFADADLDYAVEQAHQGVFFNQGQCCTAGSRIFVEESIYEEFVRRSVERAKRRVVGSPFDPTTEQGPQIDKKQYNKILELIQSGVAEGAKLECG
GKGLGRKGFFIEPTVFSNVTDDMRIAKEEIFGPVQEILRFKTMDEVIERANNSDFGLVAAVFTNDINKALTVSSAMQAGTVWINCYNALNAQSPFGG
FKMSGNGREMGEFGLREYSEVKTVTVKIPQKNS
>HsALDH1A3
MATANGAVENGQPDRKPPALPRPIRNLEVKFTKIFINNEWHESKSGKKFATCNPSTREQICEVEEGDKPDVDKAVEAAQVAFQRGSPWRRLDALSRG
RLLHQLADLVERDRATLAALETMDTGKPFLHAFFIDLEGCIRTLRYFAGWADKIQGKTIPTDDNVVCFTRHEPIGVCGAITPWNFPLLMLVWKLAPA
LCCGNTMVLKPAEQTPLTALYLGSLIKEAGFPPGVVNIVPGFGPTVGAAISSHPQINKIAFTGSTEVGKLVKEAASRSNLKRVTLELGGKNPCIVCA
DADLDLAVECAHQGVFFNQGQCCTAASRVFVEEQVYSEFVRRSVEYAKKRPVGDPFDVKTEQGPQIDQKQFDKILELIESGKKEGAKLECGGSAMED
KGLFIKPTVFSEVTDNMRIAKEEIFGPVQPILKFKSIEEVIKRANSTDYGLTAAVFTKNLDKALKLASALESGTVWINCYNALYAQAPFGGFKMSGN
GRELGEYALAEYTEVKTVTIKLGDKNP
>HsALDH1B1
MLRFLAPRLLSLQGRTARYSSAAALPSPILNPDIPYNQLFINNEWQDAVSKKTFPTVNPTTGEVIGHVAEGDRADVDRAVKAAREAFRLGSPWRRMD
ASERGRLLNRLADLVERDRVYLASLETLDNGKPFQESYALDLDEVIKVYRYFAGWADKWHGKTIPMDGQHFCFTRHEPVGVCGQIIPWNFPLVMQGW
KLAPALATGNTVVMKVAEQTPLSALYLASLIKEAGFPPGVVNIITGYGPTAGAAIAQHVDVDKVAFTGSTEVGHLIQKAAGDSNLKRVTLELGGKSP
SIVLADADMEHAVEQCHEALFFNMGQCCCAGSRTFVEESIYNEFLERTVEKAKQRKVGNPFELDTQQGPQVDKEQFERVLGYIQLGQKEGAKLLCGG
ERFGERGFFIKPTVFGGVQDDMRIAKEEIFGPVQPLFKFKKIEEVVERANNTRYGLAAAVFTRDLDKAMYFTQALQAGTVWVNTYNIVTCHTPFGGF
KESGNGRELGEDGLKAYTEVKTVTIKVPQKNS
>HsALDH1L1
MKIAVIGQSLFGQEVYCHLRKEGHEVVGVFTVPDKDGKADPLGLEAEKDGVPVFKYSRWRAKGQALPDVVAKYQALGAELNVLPFCSQFIPMEIISA
PRHGSIIYHPSLLPRHRGASAINWTLIHGDKKGGFSIFWADDGLDTGDLLLQKECEVLPDDTVSTLYNRFLFPEGIKGMVQAVRLIAEGKAPRLPQP
EEGATYEGIQKKETAKINWDQPAEAIHNWIRGNDKVPGAWTEACEQKLTFFNSTLNTSGLVPEGDALPIPGAHRPGVVTKAGLILFGNDDKMLLVKN
IQLEDGKMILASNFFKGAASSVLELTEAELVTAEAVRSVWQRILPKVLEVEDSTDFFKSGAASVDVVRLVEEVKELCDGLELENEDVYMASTFGDFI
QLLVRKLRGDDEEGECSIDYVEMAVNKRTVRMPHQLFIGGEFVDAEGAKTSETINPTDGSVICQVSLAQVTDVDKAVAAAKDAFENGRWGKISARDR
GRLMYRLADLMEQHQEELATIEALDAGAVYTLALKTHVGMSIQTFRYFAGWCDKIQGSTIPINQARPNRNLTLTRKEPVGVCGIIIPWNYPLMMLSW
KTAACLAAGNTVVIKPAQVTPLTALKFAELTLKAGIPKGVVNVLPGSGSLVGQRLSDHPDVRKIGFTGSTEVGKHIMKSCAISNVKKVSLELGGKSP
LIIFADCDLNKAVQMGMSSVFFNKGENCIAAGRLFVEDSIHDEFVRRVVEEVRKMKVGNPLDRDTDHGPQNHHAHLVKLMEYCQHGVKEGATLVCGG
NQVPRPGFFFEPTVFTDVEDHMFIAKEESFGPVMIISRFADGDLDAVLSRANATEFGLASGVFTRDINKALYVSDKLQAGTVFVNTYNKTDVAAPFG
GFKQSGFGKDLGEAALNEYLRVKTVTFEY
>HsALDH1L2
MLRRGSQALRRFSTGRVYFKNKLKLALIGQSLFGQEVYSHLRKEGHRVVGVFTVPDKDGKADPLALAAEKDGTPVFKLPKWRVKGKTIKEVAEAYRS
VGAELNVLPFCTQFIPMDIIDSPKHGSIIYHPSILPRHRGASAINWTLIMGDKKAGFSVFWADDGLDTGPILLQRSCDVEPNDTVDALYNRFLFPEG
IKAMVEAVQLIADGKAPRIPQPEEGATYEGIQKKENAEISWDQSAEVLHNWIRGHDKVPGAWTEINGQMVTFYGSTLLNSSVPPGEPLEIKGAKKPG
LVTKNGLVLFGNDGKALTVRNLQFEDGKMIPASQYFSTGETSVVELTAEEVKVAETIKVIWAGILSNVPIIEDSTDFFKSGASSMDVARLVEEIRQK
CGGLQLQNEDVYMATKFEGFIQKVVRKLRGEDQEVELVVDYISKEVNEIMVKMPYQCFINGQFTDADDGKTYDTINPTDGSTICKVSYASLADVDKA
VAAAKDAFENGEWGRMNARERGRLMYRLADLLEENQEELATIEALDSGAVYTLALKTHIGMSVQTFRYFAGWCDKIQGSTIPINQARPNRNLTFTKK
EPLGVCAIIIPWNYPLMMLAWKSAACLAAGNTLVLKPAQVTPLTALKFAELSVKAGFPKGVINIIPGSGGIAGQRLSEHPDIRKLGFTGSTPIGKQI
MKSCAVSNLKKVSLELGGKSPLIIFNDCELDKAVRMGMGAVFFNKGENCIAAGRLFVEESIHDEFVTRVVEEIKKMKIGDPLDRSTDHGPQNHKAHL
EKLLQYCETGVKEGATLVYGGRQVQRPGFFMEPTVFTDVEDYMYLAKEESFGPIMVISKFQNGDIDGVLQRANSTEYGLASGVFTRDINKAMYVSEK
LEAGTVFINTYNKTDVAAPFGGVKQSGFGKDLGEEALNEYLKTKTVTLEY
>HsALDH2
MLRAAARFGPRLGRRLLSAAATQAVPAPNQQPEVFCNQIFINNEWHDAVSRKTFPTVNPSTGEVICQVAEGDKEDVDKAVKAARAAFQLGSPWRRMD
ASHRGRLLNRLADLIERDRTYLAALETLDNGKPYVISYLVDLDMVLKCLRYYAGWADKYHGKTIPIDGDFFSYTRHEPVGVCGQIIPWNFPLLMQAW
KLGPALATGNVVVMKVAEQTPLTALYVANLIKEAGFPPGVVNIVPGFGPTAGAAIASHEDVDKVAFTGSTEIGRVIQVAAGSSNLKRVTLELGGKSP
NIIMSDADMDWAVEQAHFALFFNQGQCCCAGSRTFVQEDIYDEFVERSVARAKSRVVGNPFDSKTEQGPQVDETQFKKILGYINTGKQEGAKLLCGG
GIAADRGYFIQPTVFGDVQDGMTIAKEEIFGPVMQILKFKTIEEVVGRANNSTYGLAAAVFTKDLDKANYLSQALQAGTVWVNCYDVFGAQSPFGGY
KMSGSGRELGEYGLQAYTEVKTVTVKVPQKNS
>HsALDH3A
MSKISEAVKRARAAFSSGRTRPLQFRIQQLEALQRLIQEQEQELVGALAADLHKNEWNAYYEEVVYVLEEIEYMIQKLPEWAADEPVEKTPQTQQDE
LYIHSEPLGVVLVIGTWNYPFNLTIQPMVGAIAAGNSVVLKPSELSENMASLLATIIPQYLDKDLYPVINGGVPETTELLKERFDHILYTGSTGVGK
IIMTAAAKHLTPVTLELGGKSPCYVDKNCDLDVACRRIAWGKFMNSGQTCVAPDYILCDPSIQNQIVEKLKKSLKEFYGEDAKKSRDYGRIISARHF
QRVMGLIEGQKVAYGGTGDAATRYIAPTILTDVDPQSPVMQEEIFGPVLPIVCVRSLEEAIQFINQREKPLALYMFSSNDKVIKKMIAETSSGGVAA
NDVIVHITLHSLPFGGVGNSGMGSYHGKKSFETFSHRRSCLVRPLMNDEGLKVRYPPSPAKMTQH
>HsALDH3A2
MELEVRRVRQAFLSGRSRPLRFRLQQLEALRRMVQEREKDILTAIAADLCKSEFNVYSQEVITVLGEIDFMLENLPEWVTAKPVKKNVLTMLDEAYI
QPQPLGVVLIIGAWNYPFVLTIQPLIGAIAAGNAVIIKPSELSENTAKILAKLLPQYLDQDLYIVINGGVEETTELLKQRFDHIFYTGNTAVGKIVM
```

EAAAKHLTPVTLELGGKSPCYIDKDCDLDIVCRRITWGKYMNCGQTCIAPDYILCEASLQNQIVWKIKETVKEFYGENIKESPDYERIINLRHFKRI
LSLLEGQKIAFGGETDEATRYIAPTVLTDVDPKTKVMQEEIFGPILPIVPVKNVDEAINFINEREKPLALYVFSHNHKLIKRMIDETSSGGVTGNDV
IMHFTLNSFPFGGVGSSGMGAYHGKHSFDTFSHQRPCLLKSLKREGANKLRYPPNSQSKVDWGKFFLLKRFNKEKLGLLLLTFLGIVAAVLVKAEYY
>HsALDH3B1
MDPLGDTLRRLREAFHAGRTRPAEFRAAQLQGLGRFLQENKQLLHDALAQDLHKSAFESEVSEVAISQGEVTLALRNLRAWMKDERVPKNLATQLDS
AFIRKEPFGLVLIIAPWNYPLNLTLVPLVGALAAGNCVVLKPSEISKNVEKILAEVLPQYVDQSCFAVVLGGPQETGQLLEHRFDYIFFTGSPRVGK
IVMTAAAKHLTPVTLELGGKNPCYVDDNCDPQTVANRVAWFRYFNAGQTCVAPDYVLCSPEMQERLLPALQSTITRFYGDDPQSSPNLGRIINQKQF
QRLRALLGCGRVAIGGQSDESDRYIAPTVLVDVQEMEPVMQEEIFGPILPIVNVQSLDEAIEFINRREKPLALYAFSNSSQVVKRVLTQTSSGGFCG
NDGFMHMTLASLPFGGVGASGMGRYHGKFSFDTFSHHRACLLRSPGMEKLNALRYPPQSPRRLRMLLVAMEAQGCSCTLL
>HsALDH3B2
MKDEPRSTNLFMKLDSVFIWKEPFGLVLIIAPWNYPLNLTLVLLVGALAAGSCVVLKPSEISQGTEKVLAEVLPQYLDQSCFAVVLGGPQETGQLLE
HKLDYIFFTGSPRVGKIVMTAATKHLTPVTLELGGKNPCYVDDNCDPQTVANRVAWFCYFNAGQTCVAPDYVLCSPEMQERLLPALQSTITRFYGDD
PQSSPNLGRIINQKQFQRLRALLGCGRVAIGGQSNESDRYIAPTVLVDVQETEPVMQEEIFGPILPIVNVQSVDEAIKFINRQEKPLALYAFSNSSQ
VVNQMLERTSSGSFGGNEGFTYISLLSVPFGGVGHSGMGRYHGKFTFDTFSHHRTCLLAPSGLEKLKEIHYPPYTDWNQQLLRWGMGSQSCTLL
>HsALDH4A1
MLLPAPALRRALLSRPWTGAGLRWKHTSSLKVANEPVLAFTQGSPERDALQKALKDLKGRMEAIPCVVGDEEVWTSDVQYQVSPFNHGHKVAKFCYA
DKSLLNKAIEAALAARKEWDLKPIADRAQIFLKAADMLSGPRRAEILAKTMVGQGKTVIQAEIDAAAELIDFFRFNAKYAVELEGQQPISVPPSTNS
TVYRGLEGFVAAISPFNFTAIGGNLAGAPALMGNVVLWKPSDTAMLASYAVYRILREAGLPPNIIQFVPADGPLFGDTVTSSEHLCGINFTGSVPTF
KHLWKQVAQNLDRFHTFPRLAGECGGKNFHFVHRSADVESVVSGTLRSAFEYGGQKCSACSRLYVPHSLWPQIKGRLLEEHSRIKVGDPAEDFGTFF
SAVIDAKSFARIKKWLEHARSSPSLTILAGGKCDDSVGYFVEPCIVESKDPQEPIMKEEIFGPVLSVYVYPDDKYKETLQLVDSTTSYGLTGAVFSQ
DKDVVQEATKVLRNAAGNFYINDKSTGSIVGQQPFGGARASGTNDKPGGPHYILRWTSPQVIKETHKPLGDWSYAYMQ
>HsALDH5A1
MATCIWLRSCGARRLGSTFPGCRLRPRAGGLVPASGPAPGPAQLRCYAGRLAGLSAALLRTDSFVGGRWLPAAATFPVQDPASGAALGMVADCGVRE
ARAAVRAAYEAFCRWREVSAKERSSLLRKWYNLMIQNKDDLARIITAESGKPLKEAHGEILYSAFFLEWFSEEARRVYGDIIHTPAKDRRALVLKQP
IGVAAVITPWNFPSAMITRKVGAALAAGCTVVVKPAEDTPFSALALAELASQAGIPSGVYNVIPCSRKNAKEVGEAICTDPLVSKISFTGSTTTGKI
LLHHAANSVKRVSMELGGLAPFIVFDSANVDQAVAGAMASKFRNTGQTCVCSNQFLVQRGIHDAFVKAFAEAMKKNLRVGNGFEEGTTQGPLINEKA
VEKVEKQVNDAVSKGATVVTGGKRHQLGKNFFEPTLLCNVTQDMLCTHEETFGPLAPVIKFDTEEEAIAIANAADVGLAGYFYSQDPAQIWRVAEQL
EVGMVGVNEGLISSVECPFGGVKQSGLGREGSKYGIDEYLELKYVCYGGL
>HsALDH6A1
MAALLAAAAVRARILQVSSKVKSSPTWYSASSFSSSVPTVKLFIGGKFVESKSDKWIDIHNPATNEVIGRVPQATKAEMDAAIASCKRAFPAWADTS
VLSRQQVLLRYQQLIKENLKEIAKLITLEQGKTLADAEGDVFRGLQVVEHACSVTSLMMGETMPSITKDMDLYSYRLPLGVCAGIAPFNFPAMIPLW
MFPMAMVCGNTFLMKPSERVPGATMLLAKLLQDSGAPDGTLNIIHGQHEAVNFICDHPDIKAISFVGSNKAGEYIFERGSRHGKRVQANMGAKNHGV
VMPDANKENTLNQLVGAAFGAAGQRCMALSTAVLVGEAKKWLPELVEHAKNLRVNAGDQPGADLGPLITPQAKERVCNLIDSGTKEGASILLDGRKI
KVKGYENGNFVGPTIISNVKPNMTCYKEEIFGPVLVVLETETLDEAIQIVNNNPYGNGTAIFTTNGATARKYAHLVDVGQVGVNVPIPVPLPMFSFT
GSRSSFRGDTNFYGKQGIQFYTQLKTITSQWKEEDATLSSPAVVMPTMGR
>HsALDH7A1
MWRLPRALCVHAAKTSKLSGPWSRPAAFMSTLLINQPQYAWLKELGLREENEGVYNGSWGGRGEVITTYCPANNEPIARVRQASVADYEETVKKARE
AWKIWADIPAPKRGEIVRQIGDALREKIQVLGSLVSLEMGKILVEGVGEVQEYVDICDYAVGLSRMIGGPILPSERSGHALIEQWNPVGLVGIITAF
NFPVAVYGWNNAIAMICGNVCLWKGAPTTSLISVAVTKIIAKVLEDNKLPGAICSLTCGGADIGTAMAKDERVNLLSFTGSTQVGKQVGLMVQERFG
RSLLELGGNNAIIAFEDADLSLVVPSALFAAVGTAGQRCTTARRLFIHESIHDEVVNRLKKAYAQIRVGNPWDPNVLYGPLHTKQAVSMFLGAVEEA
KKEGGTVVYGGKVMDRPGNYVEPTIVTGLGHDASIAHTETFAPILYVFKFKNEEEVFAWNNEVKQGLSSSIFTKDLGRIFRWLGPKGSDCGIVNVNI
PTSGAEIGGAFGGEKHTGGGRESGSDAWKQYMRRSTCTINYSKDLPLAQGIKFQ
>HsALDH8A1
MAGTNALLMLENFIDGKFLPCSSYIDSYDPSTGEVYCRVPNSGKDEIEAAVKAAREAFPSWSSRSPQERSRVLNQVADLLEQSLEEFAQAESKDQGK
TLALARTMDIPRSVQNFRFFASSSLHHTSECTQMDHLGCMHYTVRAPVGVAGLISPWNLPLYLLTWKIAPAMAAGNTVIAKPSELTSVTAWMLCKLL
DKAGVPPGVVNIVFGTGPRVGEALVSHPEVPLISFTGSQPTAERITQLSAPHCKKLSLELGGKNPAIIFEDANLDECIPATVRSSFANQGEICLCTS
RIFVQKSIYSEFLKRFVEATRKWKVGIPSDPLVSIGALISKAHLEKVRSYVKRALAEGAQIWCGEGVDKLSLPARNQAGYFMLPTVITDIKDESCCM
TEEIFGPVTCVVPFDSEEEVIERANNVKYGLAATVWSSNVGRVHRVAKKLQSGLVWTNCWLIRELNLPFGGMKSSGIGREGAKDSYDFFTEIKTITV
KH
>HsALDH9A1
MFLRAGLAALSPLLRSLRPSPVAAMSTGTFVVSQPLNYRGGARVEPADASGTEKAFEPATGRVIATFTCSGEKEVNLAVQNAKAAFKIWSQKSGMER
CRILLEAARIIREREDEIATMECINNGKSIFEARLDIDISWQCLEYYAGLAASMAGEHIQLPGGSFGYTRREPLGVCVGIGAWNYPFQIASWKSAPA
LACGNAMVFKPSPFTPVSALLLAEIYSEAGVPPGLFNVVQGGAATGQFLCQHPDVAKVSFTGSVPTGMKIMEMSAKGIKPVTLELGGKSPLIIFSDC
DMNNAVKGALMANFLTQGQVCCNGTRVFVQKEILDKFTEEVVKQTQRIKIGDPLLEDTRMGPLINRPHLERVLGFVKVAKEQGAKVLCGGDIYVPED
PKLKDGYYMRPCVLTNCRDDMTCVKEEIFGPVMSILSFDTEAEVLERANDTTFGLAAGVFTRDIQRAHRVVAELQAGTCFINNYNVSPVELPFGGYK
KSGFGRENGRVTIEYYSQLKTVCVEMGDVESAF
>HsALDH16A1
MAATRAGPRAREIFTSLEYGPVPESHACALAWLDTQDRCLGHYVNGKWLKPEHRNSVPCQDPITGENLASCLQAQAEDVAAAVEAARMAFKGWSAHP
GVVRAQHLTRLAEVIQKHQRLLWTLESLVTGRAVREVRDGDVQLAQQLLHYHAIQASTQEEALAGWEPMGVIGLILPPTFSFLEMMWRICPALAVGC
TVVALVPPASPAPLLLAQLAGELGPFPGILNVLSGPASLVPILASQPGIRKVAFCGAPEEGRALRRSLAGECAELGLALGTESLLLLTDTADVDSAV
EGVVDAAWSDRGPGGLRLLIQESVWDEAMRRLQERMGRLRSGRGLDGAVDMGARGAAACDLVQRFVREAQSQGAQVFQAGDVPSERPFYPPTLVSNL
PPASPCAQVEVPWPVVVASPFRTAKEALLVANGTPRGGSASVWSERLGQALELGYGLQVGTVWINAHGLRDPSVPTGGCKESGCSWHGGPDGLYEYL
RPSGTPARLSCLSKNLNYDTFGLAVPSTLPAGPEIGPSPAPPYGLFVGGRFQAPGARSSRPIRDSSGNLHGYVAEGGAKDIRGAVEAAHQAFPGWAG
QSPGARAALLWALAAALERRKSTLASRLERQGAELKAAEAEVELSARRLRAWGARVQAQGHTLQVAGLRGPVLRLREPLGVLAVVCPDEWPLLAFVS
LLAPALAYGNTVVMVPSAACPLLALEVCQDMATVFPAGLANVVTGDRDHLTRCLALHQDVQAMWYFGSAQGSQFVEWASAGNLKPVWASRGCPRAWD
QEAEGAGPELGLRVARTKALWLPMGD

>HsALDH18A1
MLSQVYRCGFQPFNQHLLPWVKCTTVFRSHCIQPSVIRHVRSWSNIPFITVPLSRTHGKSFAHRSELKHAKRIVVKLGSAVVTRGDECGLALGRLAS
IVEQVSVLQNQGREMMLVTSGAVAFGKQRLRHEILLSQSVRQALHSGQNQLKEMAIPVLEARACAAAGQSGLMALYEAMFTQYSICAAQILVTNLDF
HDEQKRRNLNGTLHELLRMNIVPIVNTNDAVVPPAEPNSDLQGVNVISVKDNDSLAARLAVEMKTDLLIVLSDVEGLFDSPPGSDDAKLIDIFYPGD
QQSVTFGTKSRVGMGGMEAKVKAALWALQGGTSVVIANGTHPKVSGHVITDIVEGKKVGTFFSEVKPAGPTVEQQGEMARSGGRMLATLEPEQRAEI
IHHLADLLTDQRDEILLANKKDLEEAEGRLAAPLLKRLSLSTSKLNSLAIGLRQIAASSQDSVGRVLRRTRIAKNLELEQVTVPIGVLLVIFESRPD
CLPQVAALAIASGNGLLLKGGKEAAHSNRILHLLTQEALSIHGVKEAVQLVNTREEVEDLCRLDKMIDLIIPRGSSQLVRDIQKAAKGIPVMGHSEG
ICHMYVDSEASVDKVTRLVRDSKCEYPAACNALETLLIHRDLLRTPLFDQIIDMLRVEQVKIHAGPKFASYLTFSPSEVKSLRTEYGDLELCIEVVD
NVQDAIDHIHKYGSSHTDVIVTEDENTAEFFLQHVDSACVFWNASTRFSDGYRFGLGAEVGISTSRIHARGPVGLEGLLTTKWLLRGKDHVVSDFSE
HGSLKYLHENLPIPQRNTN
>CrALDH2E1
MAMSTLRAALGHAARVAHEGSLVGLLSRGMASAAQPAVDHAHHEKSRSALSYAQKLPNQLFIDGKWVDALSRKTMPVVDPRTEEVVVEVAEGDAADV
DRAVEAARRAFDTGPWPRMTAKERGRLLYRLADAMEAHVDELAQLETLDNGKPFFYSRHVDVPFAIDHLRYYAGWADKIHGKTIPVDGPYLAYTFHE
PLGVVGQIIPWNFPILMAAWKLGPALAAGNTVVLKPAEQTPMTALKVAQLAKEVGLPDGVLNVVTGYGPTAGNRVASHPGVDKTAFTGSTEVGRLVA
KAAAEQLKPCTLELGGKSPIIVCPDVDVDKAVADAHMALFFNHGQCCAAGSRVYVHEAVYDEFVRKSTEAAATRKVGDPFSSVEQGPQVDDDQFKKI
LSYIDSGKRQGAKLMTGGGRKGDRGYYVEPTVFADVKDDMKIAREEIFGPVQSIMKWKSLDDVIARANNSPYGLAAGVFSNNIDTVNTLTRALKSGT
VWVNCYNLYDNAVPFGGYKESGIGREKGEYALSNYTQVKAVYQPLSNPAWR
>CrALDH5G1
MPPRITCARTQVLNPATGAVIATLPRMRADETRAAIAAAHSVLPQWRATPARERAAILRRWHDLILQHQSDIAALMTAECGKPTAEALAEIAGGAAS
VDWFAGEAVRVAGDVLEPPSRDRRMVVLKQPVGVVGAITPWNFPMSMITRKVAPALAAGCTALVRPATTTPHIRLPPPAHTPKRAGLPDGVLNLVLG
DAAAIGHELVHSDTVRKIGFTGSTAVGKMLAAGAGAGVKRVSLELGGNAPVLVFEDADLELAARGIVASALRNAGQTCICANRVFVHTAVYDKLAEA
VVGRVRKLKVGDGAEPGVHVGPLITPAALDKAIVTAHVHDAVAKGGKLLLAGGGGGGGGGAAAAAAGNFYLPTVIGEATIDMRCFKEETFGPLIPLF
RFTSDEEAVLLANTTEYGLAAYFYTRDLGRAWRVAEELEFGMVGASMGWVGGEVAPFGGVKQSGLGREQSKYGIAEFMDIKYVCMGLG
>CrALDH6B1
MLRGQRSSPVLRAGHRFFASAAAPSPVAAAAAPPKVKLLIDGQFVDSTTENWLDVVNPANQDVLGKLPLTTKSEFNAAVKAASDAFPKWRATPVPTR
VRVMFKFQELIRANMEELARSVTMEQGKTLADARGDVFRGLEVVETACGIAPYMTGEMVENVAGGIDCYSIRQPLGVVAGICPFNFPAMVPLWMFPL
AITAGNTFVLKPSERDPGAAVMLADLAQQAGLPKGVLNIVQGSRDVVNWICDDPAIRAISFVGSDSAGKYIYARGCAAGKRVQANLGAKNHAVVMPD
ADVDSTVKALAGAAFGAAGQRCMAISAAVFVGGFTDKWREPLLEAARGLKLNAGWEKDADVGPMISPEAKARAERLIASGAAAGAQVLLDGRGVSVP
GYERGNFLGPTLLAGVTPDMEAYREEIFGPVLSCMDAATLDDALAIVNGNEHGNGTAIFTRSGAAARRFQNEVDVGMVGINVPIPVPLPFFSFTGWR
GSFAGDLHMYGRAGVQFYTQTKTVTAKWPAEDIRGISAPAATTSSSSGSKSSCGPKERLPGLDRVGAS
>CrALDH10A1
MASPVPPRLLYIGGEWVAPVKGGSLPVINPATEKEFARIPNATSEDVDAAVAAATAAFKSGHWSKTTGAYRAKYLKAIATKLREHKAVLAKAETMDC
GKPIDEASWDMDDVATCFDYYAGQAEALDGRNGAAPAIDVGMSEFDVRVRREALGVVGLITPWNYPLLMAAWKVAPALAAGCTAVLKPSELASLTCL
ELAAIAAEVGLPPGVLNVITGTGQDAGAPLSAHKGLAKVAFTGSAATGRLVAQAAAANIRPASMELGGKSALIVFEDADIEKAVEWAMFGCFWTNGQ
ICSSTSRLLVQEAVAPAFLQQLKKRAEAINVCDPLTEGCRLGPLVSEGQYRKVLSYVEAGKAEGAQLLTGGGRPAGAPGTAGYWLAPTVFAGVKPHM
RIWREEIFGPVLSVGTFSTEAEAVAAANDSEYGLAGAVISADPDRCKRVAEALECGIVWINCSQPCFCYAPWGGIKNSGHGRELGEWGLDNFLSVKQ
ITKYVSPDIWGWYNPPSKL
>CrALDH11A1
MSAQEFYAPILASTGVYKFYIDGQWKESVSGKSVAISNPSTRQTAYQVQACTQDEVNKMFESAKVAQKAWARTPLYKRAEVLHKVASLMRQYAQPIA
DCLVKEVAKPSKDSLTEVVRSADLIDYTAEEGVRYLGEGQLLNSDSFPGNARNKLCLVSKVPLGVVLAIPPFNYPVNLAVSKLAPALMAGNAVRGSA
RVSAAVCPLVLLSSCFHAAGLPAGLLSVATGRGAEIGDFLTTHPDVNCISFTGGDTGISIAKKAGMVPLQMELGGKDVCIVCEDADLDLAAKHIIKG
GFSYSGQRCTAVKLVLVAAPVADRLVAAVAAGVAKLSVGRPEDDCDITPVVSESSANFIEGLAMDAKAKGATFVTGEWRREGNLIWPVLLDHVTADM
RLAWEEPFGPVLPVMRVSSVEAAVEHCNKSKYGLQGCVFTRDINAAIRISDAMETGTVQVNSAPARGPDHFPFQGFRDSGIGSQGIRNSLAMMIKTK
STVINLDKESYTLG
>CrALDH12A1
MLGLAPRGAALGSAAPVLPSLLHLARQFTEWATVDPKKLSGAAPASCQNLVAGRWTGSRESRQLPDPLNGEPFISVPHTQVDEITPFVESLRAVPKS
GLHNPLKNPQRYLLYGDVSFRVAAEMRKPAVEDFFARLIQRVAPKSYDQALGEVRVTRKFFENFTGDQVRFLARGFTNPGDHAGQTSSGTRWPYGPV
ALITPFNFPLEIPALQLMGALYMGNKPLLHVDQRVSVVAEQLVRLLAHCGMPASDLDLLHGPGATVGEVIKRAEPRSTLFTGSQRVAERLAVETHGK
VFLEDAGFDWKIMGPDVSNVDYVAWQCDQDAYACSGQKCSAQSILFAHSNWVQAGLLNKMAAAAAQRQLSDLTIGPVLTWTTEAILAHTNKLLQIPG
AKVLFGGKPLTGHSIPAVYGAVQPTAVFVPLVEALKPEHFGTVTTEVFGPFQVVTEYGDGQLPLVLEACERMTHHLTAAIVSNDINFIQHVLAHTVN
GTTYAGIRARTTGAPQNHWFGPAGDPRGAGIGTPEAIRMVWSCHREIITDFGPVPPASGLKQS
>CrALDH18D1
MQRLVLKPAKIAQLAEGIRAIAAQEEPLGRLLRKVEVAEGLILDKVTVPIGVLLVIFEARPDALPQIASLAIRSGNGLLLKGGKEATHSNAALHKVI
VEALGPMGSDLIALVTSREEIESLLALDDVVDLVIPRGSNALVSHIKRNTRIPVLGHADGICHVYVDAAADLDSAIKIVLDAKTDYPAACNAVEKVL
IHKDWVGKGGVKAIYEALHQAGVEVHAGDAVKPLLPELPPPAPRHEYSALAVTLELVDNMEVAIDHIHKYGSAHTDCIVTTDGGRAEAFLRGVDSA
CVFHNASTRFADGFRFGLGAEVGISTSRIHARGPVGVEGLLTTKWVLRGEGHVVAKDQGVRFTHKVLAGGSEAGGSEAGGAGAGAGGRQGSTGRRRG
CVVM
>CrALDH22B1
GQPVDKDVVPCYDPSTMQLLGHLPAMSASEVRSRIARCKAAQKEWRTSSFAQRRKLLRILLKFIIENVETICRVSARDSGKPMLDAILGEVVVTCEK
IHWLSREGEAVLRPERRSAGILSFYKSARVEFHPVGVVGAIVPWNYPFHNVLNPLTAALFAGDGLVIKVSEHASWSTGYYGRMISAALAAAGAPADL
VQIVTGYGEAGSALVTGGVDKVIFVGSTQVGKMVMRAAADTLTPVVLELGGKDAVIVTEDADLDNLVQVVLKAAFLNCGQNCAGGERFFVHEKIYDK
FLERLTPLVAGLRQGNPLGDAPVDCGAMCMPGLAEKVHGLVTEAVSRGARLLAGGVLVPSGERGGQFYPPTLLADVRPGMKIWEEEVFGPVMSVIKW
STDDEVVALANDCDFGLGSNVFAGSQARARSIASRLEAGMSSINDFATTYMCQSLPFGGVKHSGFDRFAGVEGLRGLCVPKAVAEDRFPLLMRSSIP
PAWQLPLPPHAVAFGVSLVTMFYG
>CrALDH24A1

MCGAMCQLLFLTKVLKAYGGCGLQLPPPSPAAAGLLEMLAGRLTNTAQPPPRVAASDNAEQLCGSTGSGGSACLRVDDAASVGRAAAEAQRAQRTWC
AASSPAARLAVMRGFASRLESGDGAVIAELLCSETGKPLRQARAEVRLAAARVRQACGLLQQLPFTLAVGDRVTTGLGPGEGLASSFASKPQRQRTE
FVEWEPAGVVASITAWNFPLVLACDVAVPALCLGNAVLAKPSEHAVLTGLALQEVWHRAGLPQASGNARGYTLVLFVSTHAHLLVEVIGGPVNKAAA
DAFARLAAARVARLRAGDPAEASTELGPLTLGRAAAAGLQALVREAVEGGAKDWVAEQVADPAAATAATATWAVAEVESGGSFLAPAVLTGVKPGMR
VLEDETFGPVLCVVAVSSPEEAAALMSRSRYGLTAACYSRDEGVARRLLRAADVGTVFWNGCGEMPLALPWSGRRRSGLGFQLGGPEGYRAFLRPKS
HVFTRMFA
>PpALDH2B1
MHCRAVLATEQVEAPIKSPFPVQYTKLFINNSFVDSVSGKTFPSIDPRSEEVAVEVAQAAAEDVDRAVKAARKAFEEGPWPRMPGCERAGIMNRIAD
LLDEHKDELSALDTLNMGKVYDMARLGEAPLAIGLFRYYAGRWCDKAQGMTLPTNGPFHAYTLHEPIGVVGSILPWNAPFYLLAMKVAPALACGNTI
VLKPAQQSPLSALLIAKLAAEAGLPDGVLNVVTGYGDTGMHIASHMDVDKVAFTGSTQVGRQIMQAAAQSNLKPVNLELGGKSPFIIFGDADMDAAV
ESAHQAIFYNQGQMCVAGSRTFVHESVYDEYLERAKARAEKRVVGDPFKPGVEQGPQQADEAQFNKVMSYIRAGKDEGARLITGGERVGSKGYYIQP
TIFSDVQDDMKICREEIFGPVMSVIKFKTVEEVIQRSNQSEYGLGATVMSKNVDIINTVTRSLKAGIVWVNTYGILTPSAPFGGYKSSGFGRENGAY
ALANYQQVKSVIMPICNPPYL
>PpALDH2B2
MARGKARTLLSTALRATSAGPARLHRNFCAAAAAEELENPTIAPVEVKLTKLLIDGEFVDAASGKTFPTIDPRSEQVIAHVAEGDVEDVNRAVRAAR
KAFDHGPWPKMPPFQRQRILLKYADLLDQHADELAALETMDSGKPYEQARYAELPLMSRQFRYFAGWADKIFGTTGPSDGIHAVQTLHEPIGVVGQI
IPWNFPLVMYCWKVAPALAAGNTIVLKTAEQTPLSAILAGKLALEAGIPPGVLNIVSGYGPTAGASIAEHMDIDKVAFTGSTEVGKLVMAAAARSNL
KPVTLELGGKSPMIICEDANVDEAVELAHFALFFNMGQCCCAGSRTFVHESIYDEFVEKSKARALKRVVGDPFRKGVEQGPQVDKDQFHKVLGYVES
GMEQGANLITGGGRLGSKGYYIKPTIFTDVKEGMKIFDEEIFGPVQSIAKFKTLDEVVQRANNTVYGLAAGIFSNNINTVNTLSRALRAGTIWVNCF
DVFDATIPFGGYKQSGIGREKGKYVLESYTQVKAVVTPLHNPAWL
>PpALDH3H1
MEEDLEVGGRGAVRGAAELPRLVTEVREAYRNGRTRPAAWRVQQLNGIIRMISERESEIVQTLYTDLGKPSHESYVTEVSLVRSACKLAIKELKKWM
APLKVSGSITTFPSWGAIVAEPLGVALVISAWNFPFLLSVEPLVGAISAGCAMVLKPSEVAPATAALLSKLVPLYLDSSVIRVVEGGVDETTVLLDQ
QWDKIFYTGSPRVGRIVMAAASKHLTPVTLELGGKCPVYIDRTADLKVGLRRIALGKWGCNNGQACIAPDYLLIDEIIASEVVDTLIDVIETFYGKD
PKTSQDLSRIVNTKHYSRLAGFLDDPKISSKIVHGGARDDNKLYISPTLVCDVPMDSTLMSEEIFGPILPIIKVKGVQEAIDIISDRPKPLVAYVFT
KNKEVEKRIVASISSGGMVVNDTIVHFLNPGLPFGGVGESGMSSYHGKFSFDAFSHKKAVLYRNNLGDVPARFPPFTTMKQNFRRAIMDGHYLSAVI
SLTGLKK
>PpALDH3H2
MKVGGRSADHGDERVLQLVAEVREAYMTMRTKPAEWRVQQLKGLLRMVIESESEIVEALYADLGKPAHESYMSEISLVKSSCKLAIKELKKWMAPQR
LCIMHVMFGALMVSGSMITFPSSASIVAEPLGVTLVISAWNFPFLLSVDPLIGAISAGCAVVLKPSEVVYATPALLAKLIPLYMDNSVIRVVEGGVA
ETTLLLEQKWDKIFYTGNPKVGRIVMAAASKHLTPVTLELGGKCPVYFDRSANLKVCLRRIAQGKWGNNNGQACISPDYILVDESIASELVDNLKEI
IETFYGKNPISSTNLSRIVNTKHYLRLISFLEDPQICSKIVHGGERDEKKLYIAPTLVCDALMDSFLMSEEIFGPILPIIKVQGEQEAIDIINARPK
PLAAYVFTTNKAVEERMVKNVSSGGMVVNDTVMHFVNPGLPFGGVGESGMGSYHGKFSFDAFSHKKAVLYRTSLGDFPARYPPFTTKKQNFLRCVLD
GDYIGAILSLTGLKK
>PpALDH3K1
MTTIDAPQIVSELRATLRTARTRPAQWRLDQIRAVLKLVNENEDDIYAALHSDLHKSNYESFLTEVNVLVSACKSTMKNLHKWMAPEKKPIPLAVWP
ANASVISEPLGVALVISPWNFPLLLALDPVVGAIAAGCTVCLKTSEIAPATSALLARLLPEYVDTEAIKVVEGSIPEVTALLEQKWDKIFYTGNAKV
GRIIMGAAAKHLTPVTLELGGKCPLFIDDSVDLKVASKRIMVGKYGSNAGQACISPDYVLVEEHFAPTLIKQLQKTLLEFYGPDPSASVDLARIINK
NHFQRLSSMLDDPSIADKIVHGGERDEKSLYIAPTLIDNPPLDSPVMVEEIFGPMLPIITVRNVDHALNIINDKPKPLEVYVFSNNKDLFNRFRDET
SSGGIVMNDCVLQFIIPELPFGGVGESGTGAYHGKATFDAFSHRKAVLVKNMGGDVFARYPPFTVRKQSLIKALLTGTIIDIILAALGWRK
>PpALDH3K2
MTIIKPHSVLDAAKVASCLRETFRTGRTRPSNWRLEQLHAIVKLIEENEDEICRALFADLHKPRHEALTMESLVVTASAKHAIKNLNKWRAPVKKET
PYIVWPASAFILPEPLGVAFVIAPWNFPFLLAVDPVIGAIAAGCTVCLKTSEVTPATSELLSTLVPKYLDNDAIKVVEGGVPEVTALLEQKWDKIFY
TGNPKVGRIVMGAAAKHLTPVTLELGGKCPVFIDDTVDLQLASRRIMAGKYGSNSGQACISPDYILVEEHLAPKLIKQFRSTLVEFFGEDPRTSKDL
SRIVNKNHFQRLSRLLDDPATADRIVHGGERDEDSLYIAPTLIEDPALDSPIMADEIFGPLLPIITVRNVNAAIDLINDKPKALVVHLFSTNKDYVK
MFTEETSSGGLVMNDCIMQFIVPELPFGGVGESGTGAYHGKASFDTFSHFKSIFNKSQSGDAPIRYPPFTLWKQAMLRAFLEGRFFKLIQLLLGLQK
>PpALDH3K3
MSTMNPVPRLDTSQVASSLRAAFRTGRTRSVKWRLEQLHAIVKLLEENEEDIYWALDADLRKPRHEAFLSEIYTITASARYSIKNLHKWMAPAKKGV
PLLAWPASASIVPEPLGVVFIMSPWNFPFMLAVDPLIGAISAGCAVCLKASEITPTTSALLARLIPKLSARGYGVAGTEMGQDLLHWITMRAAARHL
IPVTLELGGKCPLFIDDTVDLQVASRRIMSGKFSSNNGQACIAPDYILVEEHLAPKLIKQLQSTLVQFYGEDPRSTKDLARIVNKNHFQRLSRLLDH
PSTAENIIHGGERDEESLYIAPTLIKDPPLSSPIMEEEIFGPLLPIITDHVDAAVDLINDKPKALEIYLFSTRKDYAMKFAEETSSGGLVMNDCIVQ
FAVSELPFGGVGESGTGAYHGVASFNTFSHYKSIFNKSLGVDVAFRYPPFTTQKQGMMRAFLEGRFVNFILLALGLQK
>PpALDH5F1
MVQLNEAGLFKSQGLIGDKWVDAENGHTLPVNNPATGEILTSVPFMGKREAEKAIAAASQAFTSWSKRTANDRSKILRQWFNLLIKNKDDLGKLIVL
EQGKPLAEAVGEVIVYGAAFVEYYAEEAKRVYGDIIPSPFPEKRMLVMKQPVGVVAAIAPWNFPLAMITRKVPPALAAGCTVVIKPSELTPLTALAA
AELALQAGIPPGVVNVVMGDAKGIGDAMLDSTEVRKITFTGSTGVGKMLLAGAGKTVKKVSLELGGNAPCIVFDDANLDVAVKGVLAGKYRNSGQTC
VCINKIFVQDGIYDKFAEAFAKAVSGLRAGNGLEPGITQGPLINETALEKVERHVQDAVSKGAKVLVGGKRHSLGRTFYEPTILGNASDEMLIFREE
VFGPVAPLVRFNTDEEAIKLANNSEFGLAAYAFTENITRGWRVAESLEFGMVGLNEGLISTEVAPFGGMKQSGLGREGSKYGLDEYLEMKYVCLGNM
AQPVG
>PpALDH5F2
MGFAQVGAMVQLKNMGLFKTQGLINGEWANALDNRTLAVNNPATGDVLANVPFMGKIDAEKAIAAASVAFLPWSKRTAFDRCKLLRKWFDLILENKD
DLAKLITLENGKPLTEANGEVVYGGGFVEYYAEEGKRVFGDIIPSPFPTKRMLVMKQAVGVVGAITPWNFPLAMITRKVAPALAAGCTIVLKPAELT
PLTALAAAELAVQAGIPSGVLNVVMGDAVEIGAAMMDSNEVRKITFTGSTHVGKLLMAAASKTVKKISLELGGNAPLIIFNDADIEIAVQGALYGKY
RNAGQTCVCVNRILVQDGIYDKFSEAFTKAVQKLRVGHGLDPGVTQGPLINEASLKKVEAHVQDAVSKGAKVLVGGKRHSLGGTFYQPTVLCGCNDE
MLIFREEVFGPVAPLMRFQTDEEAIKMANDTEYGLAAYAYTENIEHGWRIAEALDYGMVGLNETLISSEVAPFGGTKQSGLGREGSNYGIDEYLELK
YLCLGNIKQPFMC

>PpALDH6B1
MSGIVRQALRASKCRGYQHIGSQLLNGSKVCSRNAALFRSAVVGAARPDAVIASSRVPWDYSSFSTSAVKAVKETPALKAKNYVGGKFVESQSTEHI
DVLNPATQEVVSRVPLTTHEEFETAVAVAKEAYKTWRKTPVTARQRVMLKLQELIRRDMDKLAMSVTLEQGKTLADARGDVFRGLEVVEQACGMANQ
QMGEFVENVSSGIDTYSIRQPLGVCAGICPFNFPAMIPLWMFPMAVTTGNTFVLKPSEKDPGAAMLLAELATEAGLPPGVLNIVHGTYDVVNQICDH
PDIKAVSFVGSDVAGMHIYSRASATGKRVQCNMGAKNHAVIMPDADPEATLNALVGAAFGAAGQRCMAISTAVFVGDSKRWEEGLRERGVKLKVTGG
TEPGADLGPVISKQSKERICRLVESGAKAGARIVLDGRGIKVPGYEQGNFVGPTILADVTEDMECYKEEIFGPVLLCMEAASLQDAIEIVNRNKYGN
GTAIFTKSGAAARAFQHEVDAGQVGINVPIPVPLPFFSFTGSRGSFAGDLNFYGKAGVHFFTQIKTVTSQWKEKDLHGVAMAFPTSGKV
>PpALDH7B4
MGVEFEREDYLFLNELGISPENLGCYGGGVWRANGPTVTSVNPSDNKPIASVREASLEDYEDSMRACAEARRMWMLTPAPKRGEIVRQIGDGLRDKL
PLLGKLVSLEMGKILAEGIGEVQEFIDMCDYAVGLSRQLSGSIIPSERPNHAMMEVWNPLGIVGVITAFNFPCAVLGWNACIALVCGNCVVWKGAPT
TPLVTLATTKVIAEVLERNKLPGGIFTCVCGGAEIGSAIAYDTRIPLVSFTGSTKVGLLVQSIVHARHGKTLLELSGNNAIIVMDDAVLPLAVRAVL
FAAVGTAGQRCTTCRRLIVHEKVYDDMLAGLLKAYKQVKTGNAVDETTLCGPLHSKHSKACFEEGIKKIKAQGGKILTGGSVIDRDGNFVEPTVVEI
SHDAEIVREELFGPVLYVFKIKSLEEAIELNNSVPQGLSSSIFTRNPETIFTWIGPTGSDCGIVNVNIPTNGAEIGGAFGGEKATGGGREAGSDSWK
QYMHRATCTINYGNDLPLAQGINFGG
>PpALDH10A1
MGLHAGVDVPRRGLFIDGEWVDPVLGKRIPIVNPTTEETVGDIPAATSEDVDAAVKAAKEAFYRNKGKDWAKAGGKHRATFLRAIAKRVAERKSELA
KLESIDCGKPIDEAEWDMDDVSGCFEYYADLAEKLDERQYAPLELPMEQFKCNILREAIGVVGLITPWNYPMLMATWKVAAALAAGCTAILKPSELA
SVTCLELAGIAKDVGLPRGVLNVVTGYGHEAGAPLASHSGVDKIAFTGSTATGRSVMSAASQLIKPVTLELGGKSPIIVFDDADVEKAVEWAMFGAF
WTNGQICSATSRLLLQEGIADEFLKKLGIWASSIKVSDPLEKDCRLGPLVSEGQYEKVQKFVKVALDEGATLVCGGKRPDHLTTGYFLAPTVLSNVK
PHMQIWTDEVFGPVLAVSTFKTEEEALALANDTQYGLAGAVISKDDDRCKRVSEALEVGIVWINCSQPCFCQAPWGGNKRSGFGRELGEWGLENYLS
VKQLTRYISTDDWGWYPKPSKL
>PpALDH11A5
MFFSSAVGSETFKELLDGDTYKFYSNGEWQVSTSGKSIGVLNPTTLKLQFKVQACTPEEVNSCVEKAKVAQKGWAKTPLWKRAEALHRFASILKEQK
APIAECLVAEVAKCLKDAITEVVRSGDLLAYAAEEGVRILGKGDFLVSDSFPGNGRNKYCLASKIPLGVVLAIPPFNYPVNLAVSKLGPALVAANAV
ILKPPTQGGVSCLHMIQCLHMAGFPKGLVSAITGKGSIIEDLLTTHPSISCIRHIPQLPQPFYICHFFTGGETGMAICRKAGMIPLQMELGGKDTCL
VLEDADLELAANNIIKGGYSYSGQRCTAVKVICVMESVADDLVKRVVEKMKKLTVGPPDKDCDITPVISESSANFIQGLVDDARQQGAKFHQEWRRE
GNLIWPMLVDNVKPDMRIAWEEPFGPVIPVLRIKSPEEGIHHCNANSFALQGCVFTRDIDKAILMSNAMEGPDHFPFQGLKDSGIGSQGITNSINMM
TKTKSTVINLPVESYTIG
>PpALDH11A1MAGKGVFKEILDGDVFKYYVDGEWRRSCSGQFINVQNPSTRKPMFRVQACTQDEVNRCIDSAKAAQKIWARTPLWKRAEALHRCAA
IMKEQKAPIADCLVKEVAKALKDAVVEVVRSGDLLSYTAEEGIRILSEGKFLVSDPFPGNERNKYCLSSKIPLGVILAIPPFNYPVNLAVSKMGPAL
IAGNAVVLKPPTQGAVSCLHMVHTFHLAGFPKGLISAVTGKGADIGDFLTMHPGINCIRPVSRPLFTGGCETGIAISKKAGMIPLQMELGGKDSCII
LEDADLELAATNIIKGGFSYSGQRCTAVKVVLAMECIADQLVCKVNAKLAKLTIGMPEDDCDITPVISETSANFIEGLVHDARHKGARLHQEWRREG
NLIWPILIDHVRPDMRIAWEEPFGPILPVIRIKTVEEGIHHCNANNFALQGCIFTRSFDKAIMISDAMESGTIQINSAPGRGPDHFPFQGLRDSGVG
SQGITNSINMMCKIKSTVMNFPAATYTMG
>PpALDH11A2
MAGQGFFQDIFDGEAFKYYADGEWKVSSSRASVSITNPSTLKTQFKVQACTQDEVNKAIESAESAQKLWAKTPLWKRAEALHKFAGILKDQKNAIAD
VLVKEIAKPLKDAVTEVVRSGDLVSYSAEEGIRLLAEGKFLVSDSFPGNGRNKYCLASKIPIGVVLAIPPFNYPINLAVSKIAPALIAGNAVVLKPP
TQGAVAALHIVHCIHLAGFPKGLVAAITGKGSEIGDLLTMHPGINCISFTGGDTGIAISRKAGMIPLQMELGGKDCCIVLEDADLELAANNVIKGGY
SYSGQRCTAIKVICVMESVAEELVQKIVQRISKLKVGMPEDNCDITPVVSQSSANFIQGLVEDAQKKGAKFHQEWKREGNLIWPILIDHVTPDMRIA
WEEPFGPVIPVIRIKTVEEGIHHCNANNFALQGCIFTKDINKAILISNAMESGTIQINAAPARGPDHFPFQGLRDSGIGSQGITNSIIMMTKTKSTV
INLPVESYTMG
>PpALDH11A3
MAGTGFYESILDNDVFKYYADGEWKVSSSGKSVGITNPSTLKVQYKVQACTQDEVNKAVESAQAAQKIWAKTPLWKRAEALHRFAAILKDNKNEIAE
ALVKEIAKPHKDALTEVVRSGDLISYSAEEGIRILAEGKFLVSDSFPGNGRNKYCLASKIPLGVVLAIPPFNYPVNLAVSKIAPALIAGNAVVLKPP
TQGAVSALHMVHCLHMAGFPKGLVSAITGKGSEIGDLMTMHPGINCISFTGGDTGIAISRKAGMVPLQMELGGKDCCLVLEDADLELAANNVIKGGY
SYSGQRCTAVKVICVMESVAEELVSKIVQKMTKLTVGMPEDNCDITPVVSQSSANFIQGLVEDAQAKGAKFHQEWKREGNLIWPLLIDNVTPDMRIA
WEEPFGPVIPVIRIKTVEEGIHHCNANNFALQGCVFTKDINKAILVSDAMESGTIQINAAPARGPDHFPFQGLRDSGIGSQGITNSIQMMTKTKSTV
INLPTESYTMG
>PpALDH11A4
MAGQGFFKDILDGDVFKYYADGEWKVSSSGRSVGITNPSTLKVQYKVQACTQEEVNKSVESAHAAQKIWAKTPLWKRAEALHRFAGILKDQKNPIAE
ALVKEIAKPQKDAVTEVVRSGDLISYSAEEGIRILAEGKFLVSDSFPGNGRNKYCLASKIPLGVVLAIPPFNYPVNLAVSKIAPALIAGNAVILKPP
TQGAVSALHMVHCLHMAGFPKGLVSAITGKGSEIGDLMTMHPGINCISFTGGDTGIAISRKAGMVPLQMELGGKDCCLVLEDADLELAANNVIKGGY
SYSGQRCTAVKVICVMESVAEELVTKIVEKMTKLKVGMPEDNCDITPVVSQSSANFIQGLVEDAKAKKAKFHQEWKREGNLIWPLLIDNVTADMRIA
WEEPFGPVIPVIRIKTVEEGIHHCNANNFGLQGCVFTKDINKAILISDAMESGTIQINAAPARGPDHFPFQGLRDSGIGSQGITNSIQMMTKTKSTV
INLPTESYTMG
>PpALDH12A1
MQRCVVKRVGAVYGRSRSVIGKASSECLHPSFTHRSSSTLLSDHRPQSSLSFASCDADKLSEAHQYQMHNLVQGKWEQTSKSIELLDPLNGEKFISV
PDTSIDEISPFVQSLRACSKSGLHNPLKNPERYLLYGDIMAKAAHLLKQPQVETFFARLIQRVAPKSFAQAVGEVTVTQKFLENFSGDQVRFLARSF
VVPGNYQGQQSNGMRWPYGPVAIITPFNFPLEIPALQALGALFMGNKPILKVDSKVSIVMEQFIRLLHKCGMPPTDMDFINSDGPVMNKLLLEAEPK
TTLFTGSSKVAEKLALDLKGRVKLEDAGFDWKILGPDVQNEDYVAWVCDQDAYACSGQKCSAQSILFMHENWANQNFLERLKKLASKRKLEDLTVGP
VLTVTTERMLDHVKNLLAIPGARVEFGGKPLTNHTIPDVYGALEPTAVFVPLKEILRNEENFALATTEIFGPFQILTEYKHEDLPLVFEACERMHAH
LTAAVVSNDVHFLQEVLSNTVNGTTYAGIRARTTGAPQNHWFGPAGDPRGAGIGTPEAIKLVWSCHREIIQDVGPIPNGWSTPQCT
>PpALDH18B1
MDRSRIFIRDAKRVVIKIGTAVVTRHDGRLALGRLGAICEQVKELITDGIEVIFVTSGAVGVGRQKLRHQRMMNSRRVIFVDLQKPQVELDGKPCAA
VGQSGLMALYDSLFSQLDVASSQLLVTDNDFKDPEFRQQLSETVNSLLALRVVPIFNENDAISTRKSPYFDSTGIFWDNDSLAALLALELQADLLIL
LSDVEGLYTGPPSEPKSQLIHTYLKEKHDDMVTFGEKSRVGRGGMTAKVYAAWQAASAGIPVVISSGCVADGLQRVMRGDHVGTLFHRDAHQWVDLK

ETGARNMAVAAREGSRRLQGLTSEERKSILHAVADALLANEAIIKAENDADVELAQMTGVSKALVGRLTIKPGKIAALASSLRTLADMKEPIGEVLK
RTEVAEGLTLEKTSCPLGVVLVVFESRPDALVQIASLAIRSGNGLLLKGGKEAARSNAILHKVITEALPKSVGPNLIGLVTSRDEIPDLLKLDDVID
LVIPRGSNKLVAQIKATTKIPVLGHADGVCHVYVDTAADLEKAKNIVIDSKVDYPAACNALETLLVHEDLVATGGLEMLAFALQSAGVTLYGGARAS
GILKLPRASSYHIEYSALSCTVEVVKDVQEAIDHIHEHGSAHTDCIVTENHITAETFLHHVDSAAVFHNASTRFSDGARFGLGAEVGISTGRIHARG
PVGVEGLLTTRWLLRGSGQLVNGDKGVQYTHKKLPIGEDEGLAALATANQSLENGNSAVKTTSNGAVPS
>PpALDH21A1
MTLGHMVQKAKESSGDVTPKKYNIFLASKPVDGDRKWLDVTNKYTNDVAAKVPQATHKDIDDAIDAAVAAAPAMAAMGAYERKAVLEKVVAELKNRF
EEIAQTLTMESGKPIKDARGEVTRTIDTFQVAAEESVRIYGEHIPLDISARNKGLQGIVKKFPIGPVSMVSPWNFPLNLVAHKVAPAIAVGCPFVLK
PASRTPLSALILGEILHKIEELPLGAFSILPVSREDADMFTVDERFKLLTFTGSGPIGWDMKARAGKKKVVMELGGNAPCIVDDYVPDLDYTIQRLI
NGGFYQGGQSCIHMQRLYVHERLYDEVKEGFVAAVKKLKMGNPFEEDTYLGPMISESAAKGIEDWVKEAVAKGGKLLTGGNRKGAFIEPTVIEDVPI
EANARKEEIFGPVVLLYKYSDFKEAVKECNNTHYGLQSGIFTKDLNKAFYAFEHMEVGGVILNDSPALRVDSQPYGGLKDSGIQREGVKYAMDDMLE
TKVLVMRNVGTL
>PpALDH23A1
MFDVVNPATGKVIGELLIESKDEVVSKFEALAAGQKKWRSVPLVERRAMLERFNELLRLNMPVLAKTLSTEMGKPIAQAKNEVRATVDRVRFYLENY
EKVLKESCVLETSILKEKVVYEPLGVVANISAWNYPYFVSTNVFAAALLTGNAVLYKPSEHATLTGMEITNLLYEAGVPKNVFAMTTGKGETGAAVA
SLKGLGGLFFTGSNKTGLEIAKQAAPNLVKLQLELGGKDPVYVRADVADVGAAAASIADGAFYNCGQSCCSVERIYVDKRIYNEFLSAFIKNVMAFK
VGDPLKPDTYIGPVARQPHLPYLAAQVQDAISKGARASSHTHLESNQGGFYFPPTVLSDVNHTMDVMKEESFGPLIGIQAVENDAEALALMNDTTYG
LTASVYCKHNQDAENILRELDVGTGYWNCCDRVSPRLPWSGRRGSGLGVTLGMDGLRSFVKPKGIVFQSPSNKD
>MmALDH1A1
MSSPAQPAVPAPLADLKIQHTKIFINNEWHNSVSGKKFPVLNPATEEVICHVEEGDKADVDKAVKAARQAFQIGSPWRTMDASERGRLLNKLADLME
RDRLLLATMEALNGGKVFANAYLSDLGGCIKALKYCAGWADKIHGQTIPSDGDIFTYTRREPIGVCGQIIPWNFPMLMFIWKIGPALSCGNTVVVKP
AEQTPLTALHLASLIKEAGFPPGVVNIVPGYGPTAGAAISSHMDVDKVAFTGSTQVGKLIKEAAGKSNLKRVTLELGGKSPCIVFADADLDIAVEFA
HHGVFYHQGQCCVAASRIFVEESVYDEFVKRSVERAKKYVLGNPLTPGINQGPQIDKEQHDKILDLIESGKKEGAKLECGGGRWGNKGFFVQPTVFS
NVTDEMRIAKEEIFGPVQQIMKFKSVDDVIKRANNTTYGLAAGLFTKDLDKAITVSSALQAGVVWVNCYMMLSAQCPFGGFKMSGNGRELGEHGLYE
YTELKTVAMKISQKNS
>MmALDH1A2
MTSSEIAMPGEVKADPAALMASLQLLPSPTPNLEIKYTKIFINNEWQNSESGRVFPVCNPATGEQVCEVQEADKVDIDKAVQAARLAFSLGSVWRRM
DASERGRLLDKLADLVERDRATLATMESLNGGKPFLQAFYIDLQGVIKTLRYYAGWADKIHGMTIPVDGDYFTFTRHEPIGVCGQIIPWNFPLLMFT
WKIAPALCCGNTVVIKPAEQTPLSALYMGALIKEAGFPPGVVNILPGYGPTAGAAIASHIGIDKIAFTGSTEVGKLIQEAAGRSNLKRVTLELGGKS
PNIIFADADLDYAVEQAHQGVFFNQGQCCTAGSRIFVEESIYEEFVKRSVERAKRRIVGSPFDPTTEQGPQIDKKQYNKVLELIQSGVAEGAKLECG
GKGLGRKGFFIEPTVFSNVTDDMRIAKEEIFGPVQEILRFKTMDEVIERANNSDFGLVAAVFTNDINKALMVSSAMQAGTVWINCYNALNAQSPFGG
FKMSGNGREMGEFGLREYSEVKTVTVKIPQKNS
>MmALDH1A3
MATTNGAVENGQPDGKPPALPRPIRNLEVKFTKIFINNDWHESKSGRKFATYNPSTLEKICEVEEGDKPDVDKAVEAAQAAFQRGSPWRRLDALSRG
QLLHQLADLVERDRAILATLETMDTGKPFLHAFFVDLEGCIKTFRYFAGWADKIQGRTIPTDDNVVCFTRHEPIGVCGAITPWNFPLLMLAWKLAPA
LCCGNTVVLKPAEQTPLTALYLASLIKEVGFPPGVVNIVPGFGPTVGAAISSHPQINKIAFTGSTEVGKLVREAASRSNLKRVTLELGGKNPCIVCA
DADLDLAVECAHQGVFFNQGQCCTAASRVFVEEQVYGEFVRRSVEFAKKRPVGDPFDAKTEQGPQIDQKQFDKILELIESGKKEGAKLECGGSAMED
RGLFIKPTVFSDVTDNMRIAKEEIFGPVQPILKFKNLEEVIKRANSTDYGLTAAVFTKNLDKALKLAAALESGTVWINCYNAFYAQAPFGGFKMSGN
GRELGEYALAEYTEVKTVTIKLEEKNP
>MmALDH1A7
MSSPAQPAVPAPLANLKIQHTKIFINNEWHDSVSSKKFPVLNPATEEVICHVEEGDKADVDKAVKAARQAFQIGSPWRTMDASERGRLLNKLADLME
RDRLLLATMESMNAGKVFAHAYLLDVEISIKALQYFAGWADKIHGQTIPSDGNIFTYTRREPIGVCGQIIPWNGPLIIFTWKLGPALSCGNTVVVKP
AEQTPLTALHMASLIKEAGFPPGVVNIVPGYGPTAGGAISSHMDIDKVSFTGSTEVGKLIKEAAGKSNLKRVTLELGGKSPCIVFADADLDSAVEFA
HQGVFFHQGQICVAASRLFVEESIYDEFVRRSVERAKKYILGNPLNSGINQGPQIDKEQHNKILGLIESGKKEGAKLECGGGRWGNKGFFVQPTVFS
NVTDEMRIAKEEIFGPVQQIMKFKSMDDVIKRANNTTYGLAAGVFTKDLDKAITVSSALQAGMVWVNCYLAVPVQCPFGGFKMSGNGRELGEHGLYE
YTELKTVAMQISQKNS
>MmALDH1B1
MLTARLLLPRLLCLQGRTTSYSTAAALPNPIPNPEICYNKLFINNEWHDAVSKKTFPTVNPTTGEVIGHVAEGDRADVDLAVKAAREAFRLGSPWRR
MDASERGRLLNRLADLVERDRVYLASLETLDNGKPFQESYVLDLDEVIKVYRYFAGWADKWHGKTIPMDGEHFCFTRHEPVGVCGQIIPWNFPLVMQ
GWKLAPALATGNTVVMKVAEQTPLSALYLASLIKEAGFPPGVVNIITGYGPTAGAAIAQHMDVDKVAFTGSTEVGHLIQKAAGESNLKRVTLELGGK
SPSIVLADADMEHAVDQCHEALFFNMGQCCCAGSRTFVEESIYREFLERTVEKAKQRKVGNPFELDTQQGPQVDKEQFERILGYIRLGQKEGAKLLC
GGERLGERGFFIKPTVFGDVQDGMRIAKEEIFGPVQPLFKFKKIEEVIQRANNTRYGLAAAVFTRDLDKAIYFTQALQAGTVWVNTYNIVTCHTPFG
GFKESGNGRELGEDGLRAYTEVKTVTIKVPEKNS
>MmALDH1L1
MKIAVIGQSLFGQEVYCQLRKEGHEVVGVFTIPDKDGKADPLGLEAEKDGVPVFKFPRWRARGQALPEVVAKYQALGAELNVLPFCSQFIPMEVINA
PRHGSIIYHPSLLPRHRGASAINWTLIHGDKKGGFTIFWADDGLDTGDLLLQKECDVLPDDTVSTLYNRFLFPEGIKGMVQAVRLIAEGTAPRRPQP
EEGATYEGIQKKETAMINWDQPAEAIHNWIRGNDKVPGAWTEACGQKLTFFNSTLNTSGLVAQGEALPIPGAHRPGLVTKAGLILFGNDDRMLLVKN
IQLEDGKMMPASQFFKGSASSALELTEEELATAEAVRSSWMRILPNVPEVEDSTDFFKSGAASVDVVRLVEEVKELCDGLELENEDVYMATTFGDFI
QLLVRKLRGEDGESECVINYVEKAVKKLTLQMPYQLFIGGEFVDAEGAKTYSTINPTDGSVICQVSLAQVSDVDKAVAAAKEAFENGLWGKINARDR
GRLLYRLADLMEQHQEELATIEALDAGAVYTLALKTHVGMSIQTFRYFAGWCDKIQGATIPINQARPNRNLTLTKKEPVGVCGIVIPWNYPLMMLSW
KTAACLAAGNTVVIKPAQVTPLTALKFAELTLKAGIPKGVVNILPGSGSLVGQRLSDHPDVRKIGFTGSTEVGKHIMKSCALSNVKKVSLELGGKSP
LIIFADCDLNKAVQMGMSSVFFNKGENCIAAGRLFVEDSIHDQFVQKVVEEVGKMKIGNPLDRDTNHGPQNHEAHLRKLVEYCQRGVKEGATLVCGG
NQVPRPGFFFQPTVFTDVEDHMYIAKEESFGPIMIISRFADGDVDAVLSRANATEFGLASGVFTRDINKALYVSDKLQAGTVFVNTYNKTDVAAPFG
GFKQSGFGKDLGEAALNEYLRIKTVTFEY
>MmALDH1L2

MLWRGSQALRHFSTSRVYFKNKLKLALIGQSLFGQEVYSQLLKEGHRVVGVFTVPDKDGKADPLALAAEKDGTPVFKFPRWRLKGKTIKEVAEAYQS
VGAELNVLPFCTQFIPMDVIDSPKHGSIIYHPSLLPRHRGASAINWTLIMGDKKAGFSVFWADDGLDTGPILLQRSCDVKPNDTVDSLYNRFLFPEG
IKAMVEAVQLIADGKAPRTPQPEEGATYEGIQKKENAEVSWDQPAEGLHNWIRGHDKVPGAWAEINGQMVTFYGSSLLTSSVPSGEPLDIRGAKKPG
LVTKNGLVLFGNDGKALMVRNLQFEDGKMIPASQYFSAGETSVVELTAEELKVAETIKVIWARILSNTPVIEDSTDFFKSGASSMDVVRLVEEIRQS
CGGLQLQNEDVYMATKFGDFIQKVVRRLRGEDEEAEMVVDYVSKEVNGMTVKIPYQCFINGQFVDAEDGETYATVNPTDGTTICRVSYASLADVDRA
VAAAKDAFENGEWGRMNARDRGRLMYRLADLMEENQEELATIEALDSGAVYTLALKTHIGMSVQTFRYFAGWCDKIQGSTIPINQARPNYNLTFTKK
EPLGACAIIIPWNYPLMMLAWKSAACLAAGNTLVLKPAQVTPLTALKFAELTVKAGFPKGVINIIPGSGGVAGQRLSQHPDIRKLGFTGSTSVGKQI
MKSCAVSNLKKVSLELGGKSPLIIFSDCDLEKAVRMGMGAVFFNKGENCIAAGRLFVEEAIHDEFVTRVVEEIKKMKIGDPLDRSTDHGPQNHRAHL
EKLLQYCETGVQEGATLVYGGRQVQRPGFFMEPTVFTGVEDHMYLAKEESFGPIMVISKFQNGDIDGVLQRANNTEYGLASGVFTRDINKAMYVSDK
LEAGTVFINTYNKTDVAAPFGGMKQSGFGKDLGEEALNEYLKIKTVTLEY
>MmALDH2
MLRAALTTVRRGPRLSRLLSAAATSAVPAPNHQPEVFCNQIFINNEWHDAVSRKTFPTVNPSTGEVICQVAEGNKEDVDKAVKAARAAFQLGSPWRR
MDASDRGRLLYRLADLIERDRTYLAALETLDNGKPYVISYLVDLDMVLKCLRYYAGWADKYHGKTIPIDGDFFSYTRHEPVGVCGQIIPWNFPLLMQ
AWKLGPALATGNVVVMKVAEQTPLTALYVANLIKEAGFPPGVVNIVPGFGPTAGAAIASHEGVDKVAFTGSTEVGHLIQVAAGSSNLKRVTLELGGK
SPNIIMSDADMDWAVEQAHFALFFNQGQCCCAGSRTFVQENVYDEFVERSVARAKSRVVGNPFDSRTEQGPQVDETQFKKILGYIKSGQQEGAKLLC
GGGAAADRGYFIQPTVFGDVKDGMTIAKEEIFGPVMQILKFKTIEEVVGRANDSKYGLAAAVFTKDLDKANYLSQALQAGTVWINCYDVFGAQSPFG
GYKMSGSGRELGEYGLQAYTEVKTVTVKVPQKNS
>MmALDH3A1
MSNISSIVNRARDAFNSGKTRPLQFRVEQLEALQRMINENLKGISKALASNLRKNEWTSYYEEVAHVLDEIDFTIKGLSDWAEDEPVAKTRQTQEDD
LYIHSEPLGVVLVIGAWNYPFNLTIQPMVGAIAAGNAVVLKPSEVSDHMADLLSTLIPQYMDKDLYPVIKGGVPETTELLKEKFDHIMYTGSTAVGK
IVMAAAAKHLTPVTLELGGKSPCYVDKDCDLDVACRRIAWGKFMNSGQTCVAPDYILCDPSIQNEIVEKLKKSLKDFYGEDAKQSHDYGRIINDRHF
QRVINLIDSKKVAHGGTWDQPSRYIAPTILVDVDPQSPVMQEEIFGPVMPIVCVRSLDEAIKFINQREKPLALYVFSNNDKVIKKMIAETSSGGVTA
NDVIVHITVPTLPFGGVGNSGMGAYHGKKSFETFSHRRSCLVRSLRNEEANKARYPPSPAKMPRH
>MmALDH3A2
MERQVLRLRQAFRSGRSRPLRFRLQQLEALRRMVQEREKEILAAIAADLSKSELNAYSHEVITILGEIDFMLGNLPELASARPAKKNLLTMMDEAYV
QPEPLGVVLIIGAWNYPFVLTMQPLVGAIAAGNAAIVKPSELSENTAKILAELLPQYLDQDLYAIVNGGIPETTELLKQRFDHILYTGNTAVGKIVM
EAAAKHLTPVTLELGGKSPCYIDRDCDLDVACRRIAWGKYMNCGQTCIAPDYILCEASLQNQIVQKIKETVKDFYGENIKASPDYERIINLRHFKRL
QSLLKGQKIAFGGEMDEATRYLAPTILTDVDPNSKVMQEEIFGPILPIVSVKNVDEAINFINDREKPLALYVFSRNNKLIKRVIDETSSGGVTGNDV
IMHFTVNSLPFGGVGASGMGAYHGKYSFDTFSHQRPCLLKGLKGESVNKLRYPPNSESKVSWAKFFLLKQFNKGRLGMLLFVCLVAVAAVIVKDQL
>MmALDH3B1
MDSFEDKLQQLREAFKEGRTRSAEFRAAQLQGLSHFLRDNKQQLQEALAQDLHKSAFEAEVSEIAISQAEVDLALRNLRSWMKDEKVSKNLATQLDS
AFIRKEPFGLVLIIVPWNYPINLTLVPLVGAIAAGNCVVLKPSEISKATEKILAEVLPRYLDQSCFTVVLGGRQETGQLLEHKFDYIFFTGNAYVGK
IVMAAAAKHLTPITLELGGKNPCYVDDNCDPQIVANRVAWFRYFNAGQTCVAPDYILCSQEMQERLVPALQNAITRFYGDNPQTSPNLGRIINQKHF
KRLQGLLGCGRVAIGGQSDEGERYIAPTVLVDVQETEPVMQEEIFGPILPLVTVRSLDEAIEFMNRREKPLALYAFSKRSQVIKQVLARTSSGGFCG
NDGFMHMTLSSLPFGGVGTSGMGRYHGKFSFDTFSNQRACLLRSPGMEKINDLRYPPYSSRNLRVLLVAMEERCCSCTLL
>MmALDH3B2
MSAAETGSEPSQGAGPSEATLHSLREAFNAGRTRPTEFRTAQLRSLGRFLQENKELLQDALAKDVGKSGFESDMSEIILCENEVDLALKNLQTWMKD
EPVSTNLLTKLSSAFIRKEPFGLVLIIAPWNYPVNLMIIPLVGAIAAGNCVVLKPSEISKNTEKVLAELLPQYLDQSCFAVMLGGPEETRQLLEHKF
DYIFFTGSPRVGKIVMTAAAKHLTPITLELGGKNPCYVDDNCDPQTVANRVAWFRYFNAGQTCVAPDYILCSQEMQERLVPALQNSITRFYGDNPQT
SPNLGRIINQKHFKRLQGLLGCGRVAIGGQSDEGERYIAPTVLVDVQETEPVMQEEIFGPILPLVTVRSLDEAIEFINRREKPLALYAFSNNNQVVN
QMLERTSSGGFGGNDGFLYLTLPALPLGGVGNSGMGRYHGKFSFDTFSHHRACLLRSPGMEKLNDLRYPPYGPWNQQLISWAIGSRSCTLL
>MmALDH4A1
MLPLPSLRRSLLSHAWRGAGLRWKHTSSLKVTNEPILAFSQGSPERDALQKALKDLKGQMEAIPCVVGDEEVWTSDIQYQLSPFNHAHKVAKFCYAD
KALLNRAIDAALAARKEWDLKPMADRAQVFLKAADMLSGPRRAEVLAKTMVGQGKTVIQAEIDAAAELIDFFRFNAKFAVELEGEQPISVPPSTNHT
VYRGLEGFVAAISPFNFTAIGGNLAGAPALMGNVVLWKPSDTAMLASYAVYRILREAGLPPNIIQFVPADGPTFGDTVTSSEHLCGINFTGSVPTFK
HLWRQVAQNLDRFRTFPRLAGECGGKNFHFVHSSADVDSVVSGTLRSAFEYGGQKCSACSRLYVPKSLWPQIKGRLLEEHSRIKVGDPAEDFGTFFS
AVIDAKAFARIKKWLEHARSSPSLSILAGGQCNESVGYYVEPCIIESKDPQEPIMKEEIFGPVLTVYVYPDDKYRETLQLVDSTTSYGLTGAVFAQD
KAIVQEATRMLRNAAGNFYINDKSTGSVVGQQPFGGARASGTNDKPGGPHYILRWTSPQVIKETHKPLGDWRYSYMQ
>MmALDH5A1
MATCFLLRSFWAARPALPPPGRFRPEPAGTPRRSYASGPGGLHADLLRGDSFVGGRWLPAPATFPVYDPASGAKLGTVADCGVPEARAAVRAAYDAF
NSWKGVSVKERSLLLRKWYDLMIQNKDDLAKIITAESGKPLKEAQGEILYSALFLEWFSEEARRIYGDIIYTSAKDKRGLVLKQPVGVAAIITPWNF
PSAMITRKVGAALAAGCTVVVKPAEDTPYSALALAQLANQAGIPAGVYNVIPCSRNKAKEVGEVLCTDPLVSKISFTGSTATGKILLHHAANSVKRV
SMELGGLAPFIVFDSANVDQAVAGAMASKFRNAGQTCVCSNRFLVQRGIHDSFVTKFAEAMKKSLRVGNGFEEGTTQGPLINEKAVEKVEKQVNDAV
AKGATVVTGGKRHQSGGNFFEPTLLSNVTRDMLCITEETFGPLAPVIKFDKEEEAVAIANAAEVGLAGYFYSQDPAQIWRVAEQLEVGMVGVNEGLI
SSVECPFGGVKQSGLGREGSKYGIDEYLEVKYVCYGGL
>MmALDH6A1
MAAAVAAAAAMRSRILQVSSKVNATWYPASSFSSSSVPTVKLFIDGKFVESKSDKWIDIHNPATNEVVGRVPQSTKAEMDAAVESCKRAFPAWADTS
ILSRQQVLLRYQQLIKENLKEIARLITLEQGKTLADAEGDVFRGLQVVEHACSVTSLMLGETMPSITKDMDLYSYRLPLGVCAGIAPFNFPAMIPLW
MFPMAMVCGNTFLMKPSERVPGATMLLAKLLQDSGAPDGTLNIIHGQHDAVNFICDHPDIKAISFVGSNQAGEYIFERGSRNGKRVQANMGAKNHGV
VMPDANKENTLNQLVGAAFGAAGQRCMALSTAILVGEAKKWLPELVDRAKNLRVNAGDQPGADLGPLITPQAKERVCNLIDSGTKEGASILLDGRRI
KVKGYENGNFVGPTIISNVKPSMTCYKEEIFGPVLVVLETETLDEAIKIVNDNPYGNGTAIFTTNGATARKYAHMVDVGQVGVNVPIPVPLPMFSFT
GSRSSFRGDTNFYGKQGIQFYTQLKTITSQWKEEDATLSSPAVVMPTMGR
>MmALDH7A1
MWRVPRRLCVQSVKTSKLSGPWSRPAAHMSTLLIHHPQYAWLQDLGLREDNEGVYNGSWGGRGEVITTYCPANNEPIARVRQASLKDYEETIGKAKK
AWNIWADIPAPKRGEIVRKIGDAFREKIQLLGRLVSLEMGKILVEGIGEVQEYVDVCDYAAGLSRMIGGPTLPSERPGHALIEMWNPLGLVGIITAF
NFPVAVFGWNNAIALITGNVCLWKGAPTTSLVSVAVTKIIAQVLEDNLLPGAICSLVCGGADIGTTMARDERVNLLSFTGSTQVGKEVALMVQERFG

KSLLELGGNNAIIAFEDADLSLVVPSVLFAAVGTAGQRCTTVRRLFLHESIHNEVVDRLRSAYSQIRVGNPWDPNILYGPLHTKQAVSMFVRAVEEA
KKQGGTVVYGGKVMDHPGNYVEPTIVTGLAHDAPIVHQETFAPILYVFKFQDEEEVFEWNNEVKQGLSSSIFTKDLGRIFRWLGPKGSDCGIVNVNI
PTSGAEIGGAFGGEKHTGGGRESGSDAWKQYMRRSTCTINYSTSLPLAQGIKFQ
>MmALDH8A1
MAGKRELLMLENFIGGKFLPCNSYIDSYDPSTGEVYCKVPNSGKEEIEAAVEAAREAFPAWSSRSPQERSLVLNRLADVLEQSLEELAQAESKDQGK
TLTLARTMDIPRSVLNFRFFASSNLHHVSECTQMSHLGCMHYTVRTPVGIAGLISPWNLPLYLLTWKIAPAIAAGNTVIAKPSEMTSVTAWMFCKLL
DKAGVPPGVINIVFGTGPRVGEALVSHPEVPLISFTGSQPTAERITQLSAPHCKKLSLELGGKNPAIIFEDANLEECIPATVRSSFANQGEICLCTS
RIFVQRSIYSEFLKRFVEATRKWKVGVPSDPSANMGALISKAHLEKVRSYVLKAQTEGARILCGEGVDQLSLPLRNQAGYFMLPTVITDIKDESRCM
TEEIFGPVTCVVPFDSEEEVITRANSVRYGLAATVWSKDVGRIHRVAKKLQSGLVWTNCWLIRELNLPFGGMKSSGIGREGAKDSYDFFTEIKTITI
KY
>MmALDH9A1
MSTGTFVVSQPLNYRGGARVEPVDASGTEKAFEPATGRVIATFACSGEKEVNLAVENAKAAFKLWSKKSGLERCQVLLEAARIIKERKDEIATVETI
NNGKSIFEARLDVDTCWQCLEYYAGLAASMAGEHIQLPGGSFGYTRREPLGVCVGIGAWNYPFQIACWKSAPALACGNAMIFKPSPFTPVSALLLAE
IYTKAGAPPGLFNVVQGGAATGQFLCHHREVAKISFTGSVPTGVKIMEMSAKGVKPITLELGGKSPLIIFSDCNMENAVKGALMANFLTQGQVCCNG
TRVFVQKEIADKFINEVVKQTQKIKLGDPLLEDTRMGPLINAPHLERVLGFVKLAKEQGATVLCGGEVYVPEDPKLKHGYYMTPCILTNCRDDMTCV
KEEIFGPVMSILTFGTEAEVLERANDTTFGLAAGVFTRDIQRAHRVAAELQAGTCYINNYNVSPVELPFGGYKKSGFGRENGRVTIEYYSQLKTVCV
EMGDVESAF
>MmALDH16A1
MAATRVQPSTREIFTTLEYGPVPESHACALAWLDTHNRLLGHHVNGMWLKPEHRNPAPCQDPITGENLASCLQAEAEDIAAAVEAAKIAFKAWSQLP
GAARGQHLTRLAKVVQKHQRLLWTLESLVTGRAVREVRDGDVPLAQQLLQYHAVQAHAQGDALADWQPVGVIGLILPTPFSFLDMMWRVCPALAMGC
TVVALVPPAFPTPLLLAQLAGELGSFPGILNVVCGPASLGPVLASQPGVQKVAFCGAVEEGRVLRRTLAGRGAELGLALGTESLLLLTDSADVDSAV
EGVVDAVWSDRSLGGLRLLIQESVWDEAMRRLQARMAQIRSGRGLDGAVDMGARGAAARDLAQSFVDEAQSQGGQVFQAGDVPSSSPFFSPALVSGL
PPAAPCAQAEVPWPVVMASPFRTVKEALALANGTPRGGSASVWSERLGQALELGYGLQVGTVWINAHGLRDPAVPTGGCKESGSSWHGGPDGLYEYL
QPLGTPSQESFLCENINYDTFGLAASSILPSGPETGPSPAPPYGLFVGGRFQSPGTQSSRPIQDSSGKVSSYVAEGGAKDIRGAVEAAHQAAPGWGA
QSPRARAGLLWALAAALERRKPVLTSQLERHGAAPTVAKTEVELSVRRLQTWGTRVQDQGQTLQVTGLRGPVLRLREPLGVLAVVCPDEWPLLAFVS
LLAPALAHGNAVVLVPSGACPLLALEVCQDIAPLFPAGLVSVVTGDRDHLTRCLALHQDVQALWYFGSAQGSQFVEWASAGNLKSVWVNRGFPRAWD
VEVQGAGQELSLHAARTKALWLPMGD
>MmALDH18A1
MLRHMHRSGVQPFRQRLLPWVQSIAVPRSNRVQPSAIRHVRSWSNIPFITVPLSRAHGKPFAHRSELKHAKRIVVKLGSAVVTRGDECGLALGRLAS
IVEQVSVLQNQGREMMLVTSGAVAFGKQRLRHEILLSQSVRQALHSGQNHLKEMAIPVLEARACAAAGQSGLMALYEAMFTQYSICAAQILVTNLDF
HDEQKRRNLNGTLHELLRMNIVPIVNTNDAVVPPAEPNSDLQGVNVISVKDNDSLAARLAVEMKTDLLIVLSDVEGLFDSPPGSDDAKLIDIFYPGD
QQSVTFGTKSRVGLGGMEAKVKAALWALQGGTSVVIANGTHPKVSGHVITDIVEGKKVGTFFSEVKPAGPTVEQQGEMARSGGRMLATLEPEQRAEI
INHLADLLTDQREEILLANKKDLEEAEGRLASPLLKRLSLSTSKLNSLAIGLRQIAASSQESVGRVLRRTRIAKNLELEQVTVPIGVLLVIFESRPD
CLPQVAALAIASGNGLLLKGGKEAAHSNRILHLLTQEALSIHGVKEAIQLVNTREEVEDLCRLDKIIDLIIPRGSSQLVRDIQKAAKGIPVMGHSEG
ICHMYVDSEASVDKVTRLVRDSKCEYPAACNALETLLIHRDLLRTPLFDQIIDMLRVEQVKIHAGPKFASYLTFSPSEVKSLRTEYGDLEVCIEVVD
SVQEAIDHIHKYGSSHTDVIVTENEKTAEFFLQHVDSACVFWNASTRFSDGYRFGLGAEVGISTSRIHARGPVGLEGLLTTKWLLRGQDHVVSDFSE
HGSLKYLHENLPVPQRNFS
>SmALDH2D1
MGSDAKDLVKHTKLFIDGRFVDAVSGKTFPTFNPSNSECIAQVAEGDAADVDLAVRAAREAFDHGPWPRLAAAERGRILYKFADVIEEHLDELATLE
TLNNGMLIDLSKGIIAGSVASLRYNAGWADKLNGKTLRTDSTRMCYTLLEPIGVVGAIVPWNFPAHMFLNKVGSALTCGNTIVVKVAEQTPLTGLLL
ASLSQEAGIPAGVLNVIPGYGPTAGAAISKHMSVDKVTFTGSTEVGRMIMESAARSNLKPVTLELGGKSPFIICEDADLDSAVAVSQNAIFMHQGQV
CVAASRVFVHESIHDEFIKRSVKLASERVIGDPFQSGVQNGPQINQEQLDKVLSYIESGKKEGASLLVGGKRIGDKGFYIQPTIFGDVKQSMKIANE
EIFGPVLSVLKFKTLDEAVELANSTHYGLAAAVFSKNIDTVNLLTRSIKSGVVYVNSYLRAGPTVPFGGYKMSGIGRENGYEGLLPYLQHKSILMPL
ENSPWN
>SmALDH2B2
MAPSTISDLPIGPAPVSVKYTKLFIDGQFVDAVSGRTFETLDPRNGEVISKVAEADKQDVDVAVKAARKAFDHGPWPRLSGYARGRILLKFADLLEH
HFDELAALETLDNGKPLDLVKYVDLPMALRLLRSFAGFADKICGKTVKIDGPYHAYTLLEPIGVVGQIIPWNFPLIMFFLKISPALAAGNTIVLKTA
EQTPLSALFCASLLKEAGLPPGVLNILSGFGPTAGAAISSHNDVDKIAFTGSTDVGKLVMEAAAKSNLKAVSLELGGKSPMIVLDDADVDVAVELAH
LALFFNVGQCCVAGSRVFVQEGIYDEFLRKAADRAKRRVTGDSFQSGVDHGPVVDQQQFDRVLGYVEIGKREGARLVTGGCRIGSRGFYIEPTIFAD
VEDYMRIAREEIFGPVMSVLKFRTIDEVIQRANDTAYGLAAGIVTKDLNSANRLTRSLRAGTVWINCYHVFDPALPFGGYKMSGIGRENGKQVLYQY
SQVKSVVTPVESPWL
>SmALDH2B4
MEFGTILAYWWLNARIWILHQLAWCKTFPTIDPRSEEIIAQVAEGDEEDVNRAVKAARNAFEKGPWPRMTAYERSKILFRYADLLEQHSDELVALDV
LDNGKTIDQATFAEMPNVIRWFRYYAGWADKIHGMTLQADSPHHVHTLHEPIGVVGQIVPWNFPIIMFSWKVAPALACGNTVVLKSAELTPLSAILA
GTLALEAGVPPGVLNIISGFGHTAGAAIASHMDIDKVAFTGSTEVGRSVMEAAARSNLKPVTLELGGKSPFIVCGDADIDKALELSHLALFFNQGQT
CCAGSRTFVHESVYDEFVEKAKKKAENRVLGDPFQSGVEHGPQVDISQFNKVMKYIGYGKEQGATLLTGGERHGDKGFYIQPTVFADVGDSMAISRD
EIFGPVQCISKFKTLEEVVERANNTQYGLAAGVFTQSLDTANFLSRALKVGTVWVNTYYAFDAAIPFGGYKMSGFGREKGEYVLKNYLQVKAVVTPL
KNPAWL
>SmALDH2B1
MDFLSRDKLKETIEHTQLFIDGQFVDSASGKKFAAFDPSTGETIADVAEGDERDVDLAVQAARKAFEEGPWPRLAGAKRGKILAKLADLMEAKIMDL
STLETLNNGMPLQATMFMTNAAIDVLRYYGGWADKIAGKTLKGDGDVHAYTLYEPIGVVGAIVPWNFPVYLLVCKIAPALVCGNTMVVKPSEQAPLT
ALWIAKLALEAGVPAGVLNIVPGFGPTAGAAIARHMDIDKLTFTGSTNVGRLVMNDAASSNLKQVTLELGGKSPFIICEDANLEVAAFFSHLAIFFH
QGQVCLAGSRVFVHESVYDAFVEKAVAMAKRRVIGDPLKIEVEHGPQINQAQADKILSYIESAHAEGARLVTGGKRIGDKGFYIEPTIFADVTQSMT
IAKEEIFGPVLSVLKFKTLDEAVKLANSTSYGLAAAIFAKDIDTVNFLSRSIKSGIVFVNSYFSAGPGIPFGGYKMSGIGRENGYEGLLPYLQTKSV
VMPLANSPWL
>SmALDH2B3

MKSSILNSLSMGSLWTLPLVSETFPTVDPRTEEVLADVAKADVEDINRAVKAARKAFDHGPWPRMTAYERSKILLKYADLLEKHNDELATLDSLDSG
KLYSQSQGVEIPHFVTRLFRYYAGWADKIHGKTLPADGPHQVLTFHEPIGVVGQIIPWNFPMVMFAWKVAPALACGNTIVLKTAEQTPLSACLAAKL
AVEAGLPPGVLNVVSGFGETAGAAISSHMDIDKVAFTGSTETGKLVMQAAARSNLKPVTLELGGKSPFIIMPDADIDQAVELSHFALFFNQGQCCCA
GSRTFVHESIYDEYIEKAKARALKRVVGDPFKSGVEQGPQVDKAQFEKILSYIDVGRHEGANLVTGGARIGNKGYYIQPTIFSDVKDDMAISRDEIF
GPVQAVTKFRTVQEAIERANNSPYGLAAGVFTKDIDTANTFSRALRVGSVWINCYDVFDAAIPFGGYKMSGQGREKGEYVLHNYTQVKAVVTPLKNP
AWL
>SmALDH2D2
MGSDAKDLVKHTKLFIDGRFVDAVSGKTFSTFNPSNSECIAQVAEGDAADVDLAVRAAREAFDHGPWPRLAAAERGRILYKFADVIEEHLDELATLE
TLNNGMLIDLSKGIIAGSVASLRYNAGWADKLNGKTLRTDSTRMCYTLLEPIGVVGAIVPWNFPAYLFLNKVGSALTCGNTIVVKVAEQTPLTGLLL
ASLSQEAGIPPGVLNVIPGYGPTAGAAISKHMRVDKVTFTGSTEVGRMIMESAARSNLKPVTLELGGKSPFIICEDADLDSAVAVSQDAIFMHQGQV
CVAASRVFVHESIHDEFIKRSVKLASERVIGDPFQSGVQNGPQINQEQLDRVLSYIESGKKEGASLLVGGKRIGEKGFYIQPTIFGDVKQSMKIASE
EIFGPVLSVLKFKTLDEVVELANSTHYGLAAAVFSKNIDTVNLLTRSIKSGVVYVNSYLGDGPAVPFGGYKMSGIGRENGYEGLLPYLQHKSILMPM
ENSPWN
>SmALDH3H1
MESAVIAAVCGEVRGDFKSGRTRSLDWRLAQLKSIVDLIKKHEEDITEAVAVDMGKPSYECFASEIFPVKSACQLAIKNLKNWTAAVNVPPLLATLP
ASASMKPEPFGVVLIISAWNFPFLLSLDPMVGAIAAGNAVVLKPSEMAPATSALIARLLPLYLDKSAIRVVEGGVPETTALLQQKWDKIFYTGSPRV
GRIVMAEAAKNLTPVTLELGGKSPVIVDSSSDLKVATRRIAVGKWGNNNGQACVSPDYVLVDSSCSTKFIEAMKDTLKSFYGENPRESMDISRVVNI
NHFNRLVGLLDDPNIASKIAHGGEKDETKLYIAPTLLEDVPLDSKVMSEEIFGPILPIISVRSIDEAIDIVNSRPKPLALYLFTKKDKVKEKVIAET
SAGGMVVNDCCLHFLTTTLPFGGVGESGMGSYHGKFSFDAFSHHKAILTRPFWMDIMARYPPYSAHKKTFIRCLLEADFVGVILCLLGLKG
>SmALDH3H2
MDPLQTDRLVAELRDEFRSGRTRPMDWRAAQLRAMLRMIDEREGEIIEALDRDIGKPAYETYVAELSTIANSCTNALKHLRSWMAPEKVSTSMISFP
SSGEIVPEPLGVALVISAWNFPFLLSLDPVIGAICAGNAVVLKPSELAPATAALLAKLVPLYLDKKAIRVVEGGVPETTALLDQQWDKIFYTGSTRV
GKIVMAAAAKNLTPVVLELGGKSPVLVDSNVDVKVTARRIALGKWGNNNAQACISPDYILADESVVPKLITAIKECLLEFYGDDPSRSKDIARVVNG
SHFERLTGLLDEDGVKDKIVFGGARDSNKLFIAPTVILDPPADSAVMTEEIFGPLLPVIPVDSMESAMSFVNTRPKPLALYLFTRDKALEKKVVSET
SAGGMVVNDTVLHFVTETMPFGGVGHSGMGAYHGKFSFDAFSHRKAVLYRGFWADMASRYPPYTIAKQNFVRNFLQGNYLEAIKALLQNLRS
>SmALDH5F1
MATIASAGASQAVLDKINNAGLLRAKGLISGEWVSAEDGRTLPVYNPATGEFLTEVPLMGERETLSAIDSAHNAFKSWSKKTCSERSKLLRRWYELI
LEKKEEIAQLMTLEQGKPLKEALGEVSYGAGFIELFAEEAKRTYGDIIPSPYPDRRLLVVKQPVGVVGTITPWNFPLAMITRKVAPALAAGCTVVIK
PAELTPLTALAAAELSIQAGIPPGVINVVMGDAPQIGAALLESTKVRKISFTGSTQVGKKLMAGAASTVKRLSLELGGNAPCIILDDADIEVAVKGA
LASKYRNSGQTCVCANRVLVQDGIYDKFAEAFIQAVSGLKVGNGLEEGVTQGPLINEAAVKKVEQHVEDALSKGAMLLAGGHRHRLGAAFYEPTVLG
DATEEMLIMKEEVFGPVAPLIRFKTDEDAIRIANSTEAGLAAYLFSGSISRAWRVAEALEYGMVGWNEGVISTEVAPFGGVKQSGLGREGSKYGIDE
YLEMKYICIGNMLP
>SmALDH6B1
MIRRVFCGVLARSSAAKGIPGDNRALVAGLNAVFSPRNFSSLPEKELNDSAIAFGQPKVKLLVGGEFIDSKTSDWVDVVNPATQEIVSRLPLTTPSE
FDAAVDSAKAAFPKWRDTPVTTRQRVMLKLQELIRRDMDKLALNVTTEQGKTLGDARGDVFRGLEVVEFACGAATLQMGEFVENVSTGIDTYSIRQP
LGICAGICPFNFPAMIPLWMFPVAVTCGNTFVLKPSEKDPGASLMLAELAAEAGLPPGVLNIVHGTNDVVNRICDHPDIKAVSFVGSDKAGMHIYSR
AAATGKRVQCNMGAKNHAVIMPDASPEATMNALTGAAFGAAGQRCMAISTAVFVGDSKPWEEGLKQRAMKLKIGCGTEPGADLGPVISKQILAVQAK
ERICSLVESGLKDGARVVLDGRNVEVPGFTSGNFVGPTILADVRPDMDCYKEEIFGPVLLCLKAETLEEAIEIVNSNKYGNGTAIFTTSGPAARKFQ
HEIDVGQVGINVPIPVPLPFFSFTGSRGSFAGDLNFYGKAGVHFFTHMKTVTTQWKDSPGGVTMAFPTSQKV
>SmALDH7B4
MDFGSRPELRFLEDLGLQATNQGCYGGGEWRAGGKSVSSLSPASNQPIATVIEGSLEDYERSLKACESARESWMLTPAPKRGEIVRQIGDAFRKNLE
NLGRLISLEMGKILVEGIGEVQEVIDMCDFAVGLSRQLSGLILPSERPNHMMMEVWNPLGIVGVITAFNFPCAVLGWNACIALVCGNCVVWKGAPTT
PLVSIATTKIIAGVLERNGLPGAIFTCICGGAEIGEAIAKDSRIPLVSFTGSSKVGQIVQCHVNSRFGKCLLELSGNNAIVVMDDADLSLAVRSVLF
AAVGTAGQRCTTCRRLFVHQAIYKDFIEKLVTAYGQLKVGDPLKHDSLVGPLHSSQSKQAFEQGLEAIASQGGKFLTGGVGTTTSPGENYVTPSIVE
ISHDADVVKEELFGPLLYVFQFKTLDEAIAMNNSVPQGLSSSIFTKSHETIFKWIGPTGSDCGIVNVNIPTNGAEIGGAFGGEKATGGGREAGSDSW
KQYMHRATCTINYGKTLPLAQGINFN
>SmALDH10A1
MAPAIPSRLLYIGGQWRAPDLGGQIAVINPATEDTIGYIPAATADDVDLAVKAAREAFTKDNGKYWARTTGKYRAKFLRAIAAEVTARKSQLAELEA
MDCGKPLDEAAWDMDDVAGCFDYYAGLAEGLDAKGHLSVELPMDTFKTNVLKEPIGVVGLITPWNYPLLMATWKVAPALAAGCTAILKPSELASVTC
LELASIAAKVGLPAGVLNVVTGLGKDAGAPLSKHPGVDKVAFTGSTATGKSIMGAAAEIIKPVTLELGGKSAIIVFDDVDIEKAVEWTMFGVFWTNG
QICSATSRLLLQENIASVFLERLAEWTKTIKISNPLEPGCRLGPVVSDGQYKKVMKYISTAQEEGATLLCGGKRPEHLSKGYFVEPTVFANVRPSSQ
IWKEEVFGPVLAVRTFRTEEEAIKLANDSEYGLAGAVISTDEDRCQRVAELLQAGIIWINCAQPTFTQAPWGGTKRSGFGRELGEWGLENYLSVKQV
TKYISEEQWGWYPRPSKM
>SmALDH11A1
MAGTGTFKEIVEGDVYKFYADGQWRLSSSGNISYVYNPTVAGKQAYKITACSKDEVNQAFQSAKAAQKIWAKTPLYKRAEMLHKVAEIMRKNKGPIA
ECLVKEVAKPAKDSVTEVVRSADLLSYTAEEGIRILSEGKFLVSDSFPGNPRNKLCLASNAPLGVILAIPPFNYPVNLTCSKIGPALIAGNAVVVKP
PTQGSVSTLHMMHCFHLAGFPKGLLSCVPIKASELGDYVTTHPLVNCISFTGGCDTGMSISKKAGMVPLQMELGGKDAMIVLEDGDIELAATNIIKG
GFSFSGQRCTAVKVVLVMESVADKLVSSVNAKVAQLKVGPPENDCDIVPVVTEASANFIEGLVTDAKAKNAKLCQAKFSRQEWKRQGNLIWPLLIDN
VTTDMRIAWEEPFGPVIPVMRIKTPEEGVKHCNSSKYGLQGCVFTSDINKAMLLSDAMESGTIQINSAPARGPDHFPFQGIRESGVGSQGVTNSINL
MTKIKTTVLNLPTASYTMGTNKLPFSSLLSFLLSLVLGSGRRRVFADREKRTSVW
>SmALDH11A2
MSIGGDCRCCVDEEIDAFGEIVGGNGVFKYYADGAWKISSSEESLPVINPTSREPFVAITACTRREVNKAFHSAKEAQKSWAKTPLFKRAEMLHKVA
GVMKEKKNMEAIAECLIKEVAKPDKDAYTEVVRSADLLSYTAEEGIRILSQGKFLVSDSFPGNERNKVCISSKVPLGIILAIPPFNYPVNLTVSKIG
PALIAGNAVVIKPPTQGAVSTLHMIHCFHMAGFPKGLISCVTIKASELGDYMTTHPLVNCISFTGGCETGISISQKAGMIPLQMELGGKDAMIVLED
ADLDLAATNIIKGGFSYSGQRCTAVKIVLAMEGIADALVSKVNAQIAKLKIGPPERIDTDIVDVVSEASADFIETLVADAIDSGAKLCQGWKRRGNK

IWPLLIDNVTTEMRIAWEEPFGPVIPVMRIKSVEEGIQHCNSSKYGLQGCVFTKDINKAILVSDAMESGTIQINAAPARGPDHFPFQGIRDSGIGSQ
GVTNSINFMTKIKTTVINLPSASYSLGKILFLELKGLAMERAFPCFQLWVIMSTRNRKGSWMPEILKL
>SmALDH11A3
MAGTGVFKACLDGDVYKYYADGDWKMSSSGKTVSVTNPTTGKPIYKVTACTQDEINKVFQSAKAAQKLWAKTPLYKRAEMLHKVSVIMKEHKSPLAD
CLVKEVAKPAKDAVTEVVRSGDLIDYTAEEGIRIMSEGKFLVSDPFPGNERNKLCLSSKVPLGVILAIPPFNYPINLSVSKIGPALITGNSVVIKPP
TQGAVSTIHMMHCFHLAGFPKGLISCVTIKVSELGDFLTTHPAVNCISFTGGCDTGIAISKKANMIPLQMELGGKDTMLVLDDADVESAAVNIMKGG
FAFSGQRCTAVKVVLAMESIADALVSKVNAKVAKLSVGPPENDCDIVPVVSESSANFIEGLVMDAKQKGAKLCQEWKREGNLIWPLLIDHVKPDMRI
AWEEPFGPVIPVLRIKTAEEGINHCNSSRYGLQGCVFTKDVNKAMLVSDAMETGTVQINAAPARGPDHFPFQGIRDSGIGSQGVTNSINMMTKIKTT
VINLPQPSYTMA
>SmALDH11A4
LAGQGPFKSVLDGDVFCYYADGEWKTSSSGKSLAVLNPSNGKTQYKVTACTRDEVNKAFQSAKKAQKEWAKVPLCKRAEYLYKAAAILKAHKTPIAE
CLVREVAKPAKDSVTEVVRSADLIVYTAEEGIRILSQGNFLVSDSFPGNDRNKLCFTSKVPLGVVLCIPPFNYPVNLSVSKLGPALIAGNAVVLKPP
TQGAVSAIHMMHCFHLAGFPKGLLSCVTIKVSELGDYLTTHPMVNCISFTGGCETGISISRKANMVPLQMELGGKDAFIVLDDADLEAAATNVIKGG
FAFSGQRCTAVKVVLAQESIADTLVSKINAKVGKLTVGPPEQDCDIVPVVSDASANFIEGLVSDAKQKGAKLCQDWKRQGNLIWPILVDKVKPEMRI
AWEEPFGPIIPVIRIKTQEEGIKHCNASRYGLQGCVFTQSLDRAMQVSDAMETGTVQINAAPARGPDHFPFQGVRDSGIGSQGITNSINMMVKIKTT
VMNLPSNSYTMA
>SmALDH11A5
GPFQDIVEEDGSFKYYADGEWQTSSSRRSVTISNPATREAQYKVQACTQEEVNRAIDSAKAAQRVWAKTPLWKRAEKLHKAASLLKELKNPIAECLI
KEIAKPAKDAMSEVVRSGDLISYTAEEGIRVLAEGKFLVSDSFTGNDRNKLCLASKIPLGVILAIPPFNYPVNLAVSKIAPALIAGNAVVVKPPTQG
AVAGLHAIHCFHLAGFPKGLISCITGKGSEIGDFLTMHSGVNCISFTGGDTGIAISKKAGMIPLQMELGGKDACIVLEDADIDLAATNIVKGGFSYR
QVLDGQRCTAVKLVLVMDSVADELVAKVNARISKLTVGSPEDNSDITAVVSEASANFIEDLVEDAREKGASFCQDYKRKGNLIWPLLVDRVTPDMRL
AWEEPFGPVIPVIRIKSVEEAIHHCNSSNLGLQGCVFTRDINKAMIMSDAMETGTVQINSAPARGPDHFPFQGLRDSGIGSQGVTNTINMMTKTKTT
VINLPGPSYSMG
>SmALDH11A6
MAAGTGPFEEIIDGGVYKYYADGEWRTSSSGKSVTIYNPSTREAQYKVQACTQEEVNKAIDSAKAAQKLWAKVPLWKRAEALHKAAALLKELKDPIA
ECLVKEIAKPAKDAVSEVVRSGDLISYTAEEGVRILAEGKFLVSDSFPGNDRTKLCLTSKIPLGVVLAIPPFNYPVNLAVSKIAPALIAGNAIVLKP
PTQGAVAGLHTVQCFHRAGFPKGLISCITGKGSEIGDFLTMHPGVHCISFTGGDTGIAISKKAGMIPLQMELGGKDACIVLDDADIDLAAANVVKGG
FSYSGQRCTAVKVVLVMESVADDLVSKVNAKIAKLKVGAPEDDCDITAVVTESSANFIEGLVKDAKEKGATFCQEYKREGNLIWPLLLDHVRPDMRI
AWEEPFGPIVPVIRISSVEEGIHHCNASNFALQGCVFTRDVNKAMMISDAMETGTVQINSAPARGPDHFPFQGLRDSGIGSQGVTNSIAMMTKIKTT
VINLPAPSYSMG
>SmALDH12A1
MDSSSALAFATVYPDDLSTAHPYQVENLVRGRWGKSLKSSKLPDPLNGGEFITVPEATGHELPEYIESLQSCPKSGLHNPLKNPERYLLYGDISARA
ASSLKHREVGHFFARLIQRVSPKSYQQAQAEVTVTQKFLENFSGDQVRFLAKSFAAPGNHLGQQSIGYRWPYGPVAIITPFNFPLEIPVLQLMGALY
MGNKALVKVDSKVSVVMEQMLRLLHACGLPPNDTDFINCDGPVMNKLLVEAKPSMTLFTGSSRVAEKLALDLKGRIKLEDAGFDWKILGPDVNEIDY
VAWVCDQDAYACSGQKCSAQSILFMHENWSSRNFVEKLKQLAAKRKLDDLTIGPVLTVTTKTMLDHMEKLLSIPGSSVAFGGKPLENHSIPDVYGAI
EPTAIFVPLKEILKDEHFDLVTKEIFGPFQVITEFKQCELPLVLRACERMHAHLTAAVVSNDVEFLQEVLGETVNGTTYAGIRARTTGAPQNHWFGP
AGDPLGAGIGTPEAIKLVWSCHREIIQDFGPVPKNWTSTTS
>SmALDH18B1
MRQQIDPSREFTRDVRRVIVKVGTSVVTRPDGKLAVGRLGALCEQVKDIMDDHIEVIVVTSGAVAVGRQKLRQQRMMNSRQVRRVIHHFCFLLVCLD
LQKPQAELSGKACAAIGQAGLMALYDSIFSQLDVASSQLLVTDREFIDPEFRKQLCETVDTLLQLRVVPIFNENDAMSTRRAPYKDSSGIFWDNDSL
AALLALELKVDLLILLSDVEGLYTGPPSDPSSKLISTYIPAQHETITFGEKSRYGRGGMTTKVTAAREVASAGIPVVITSGYTPDGFHRVLRGDSIG
TLFHKDAHIWTLTINNDARLMAVAARDSSRRLQSLTSGERQNILYDVADALERNEETIRSENQADVRLAEELGIAKPLLSRLTLKPGKIADLAKAVR
ALADMREPIGSILQRTEVAENLTLDKTSCPLGVILVIFESRPDALVQIASLAIKSGNGLLLKGGKEAARSNAILHKVITEALPKSVGKELIGLVTSR
DEIPDLLKLDDVIDLVIPRGSNKLVSQIKESTKIPVLGHADGICHVYVDKAARLDVATKIAIDSKVDYPAACNAMETLLVHEDLVDTGGLETLASAL
KSAGVTLNGGERASKLLNIPKMTKFHHEYSGLACTVEVVKDVHAAINHIHEHGSAHTDCIVTEDKEVAELFLQQLDSAAVFHNASTRFSDGTRFGLG
AEVGISTSRIHARGPVGVEGLLTTRWLLRGNGQVVQGDRGVVYTHKDVEVDSMEGWTSMGNGLPASVNGGSLKKSASPFLGSVLAPSVDAAT
>SmALDH21A1
MKFPMYIASCARDQSANYLDVIDKHTGQAAAKVPLASPEDIEEAIQECVKAAPAMAALPSYERKAVLRKIVFELEKRSEEIAQLITTESGKPIKDAR
GEVQRSIDTFEVAAEEATRIYGEVLPLDISARNKGIEGIVKKFPIGPVSMVSPWNFPLNLVAHKVAPAIAVGCPFVLKPSSRTPLSALFLGEILASC
DSLPKGAFSILPTTRNEADAFTTDDRFKLLTFTGSGMAGWNMKARAGKKKVVMELGGNAPCIVEDLVPDLEGTIARLVHGGFYQSGQSCIHMQRLYV
RGGLYKEVKDALIAAVKKLKGGDPRQDDTSIGPMISESSAATVEKSVNEAVKAGAKLLVGGKRRGAFMEPTVLEDAPFDTDARKEEIFGPVILLYSY
NDFKEAVKEANNTHYGLQAGVFTRDLNKAFYAFEHIEAGGVCLNDSPSMRVDSQPYGGIKDSGIQREGVKYAMDDMLETKVLVMRNVGNASYF
>SmALDH22A1
MDVLWAVAILAILFYLCRLLMLLIPPVVPEIVVDTSDVMLGKGGDDDSYIYVARRRSGDEDTVHCYDPATMKYLGQLPALNFDEVSDHVARAREAQK
KWARSSFEKRRQLLRILLKYTIDHQELICEVSARDSGKTLVDAALGEILTTCEKITWLVGQGEQWLQPEYRLVKLFISAGRMMLHKTARVEYSPIGV
IGAIVPWNYPFHNILNPMVSAVFAGNAIVIKVSEHASWSAFFYSRIIKAALRAAGAPADLVHVITGYGETGKALVSLVDKLIFVGSTAVGKMVMEQA
AKTLTPVVLELGGKDPFIVCEDADVAQIAARAALQSSGQNCAGAERFYIHAQIYQQFVDEVVRIVRTVRMGPPLEGLFDMGAVCIQEHTDRLQALVN
DAVTKGAEIAVRGDLVLPDFGNSVVGQFYPPTVLLNVNHSMRLMQEEIFGPIIPIMKFHSDDEAITLANDSNFGLGCSVFSANKERAVAIASKIYCG
MAAINDFAVTYMCQSLPFGGVKNSGFGKFAGVEGLRGCCLVKSIAEDRFSFFKTYPVAENAFQFEEALVRMFYGLTVVEKFQGLVNLVKIFTEQKDV
KKTL
>SmALDH23B1
MASFCKIVKRFYRANKPIKPNTSWPGYRNVLDITNPATLKKVGELQEDTIPDIERKLEYLHTGLKRWKLTHVDQRKAALEKFADALVTRKTTLAKIL
TSETGKPISQARSEIAATVDRIHYFVENCEKVIGTQTVLESSRLKEKVQYEPLGVIANISAWNYPYFLSANVFAPALLTGNCVLYKPSENASLTGQE
ITDMLHNAGIPEDVFVISSGGADTGSRIAGNKDIGGLFFTGSYDTGLEIAKKASPNLVKLQLELGGKDAAYIRHDVPNILATATTVADGAFYNSGQS
CCSIRRIYVNKRVYVPFMEALKKISHSYRIGDPTLEDTYIGPLCTKKQVEKIGKLLAEAINRGAQVDVGGDTTNSTAHKVGYFVPPTILTDVDHQMS

IMRKETFGPVVGVMCVEDDDEATTLMSDTEYGLTASVFSKHLEDAEAILNELKVGTGYWNCCDRVSPRVPWSGRKRSGVGSTLGIDGLRAFVQPKGF
FCHEPMAAI
>SmALDH23B2
MASFCKIVKRFYRANKPIKPNTSWPGYRNVLDITNPATLKKVGELQEDTIPDIERKLEYLHTGLKRWKLTHVDQRKAALEKFADALVTRKTTLAKIL
TSETGKPISQARSEIAATVDRIHYFVENCEKVIGTQTVLESSRLKEKVQYEPLGVIANISAWNYPYFLSANVFAPALLTGNCVLYKPSENASLTGQE
ITDMLHNAGIPEDVFVISSGGADTGSRIAGNKDIGGLFFTGSYDTGLEIAKKASPNLLELGGKDAAYIRHDVPNILATATTVADGAFYNSGQSCCSI
RRIYVNKRVYVPFMEALKKISHSYRIGDPTLEDTYIGPLCTKKQVEKIGKLVDVGGDTTNSTAHKVGYFVPPTILTDVDHQMSIMRKETFGPVVGVM
CVEDDDEATTLMSDTEYGLTASVFSKHLEDAEAILNELKVGTGYWNCCDRVSPRVPWSGRKRSGVGSTLGIDGLRAFVQPKGFFCHEPMAAI
>OtALDH3
MRASFRSGHTLPMRKRKEQLGQLLKMLKEREDEILDALREDLSREHVEAFYYDFALPRAEIRAMLRNIRSWTGRSLVKAFNVITWPSKQWMERQPLG
CALVCSSWNFPFLLSLVPVAGAIAAGNAVVLKPSNDSKASTALLVKLVREYCDPRVVQCVGSEVPGNGVDVMQTVLKEKFDVIFFTGSSKVGKIVAR
AAAENLTPCILELGGKNPVVVTDCADVDLAAKQCVWGRVINCGQQCISPEYVLCHESRCDEFERMCSKWAAKFVPDVTLNGAMARIGGPDPESRMKA
IAKLIDDAKAGVAGDTVVYGGTYDVKRRLVEPTVIKCGEKSPFMEAELFAPILCVHSYKTLGQAVDTIQAQMKPLTMYVFSRSAKKTRFLLDNTHAG
GVTVNGTLTHCAHDRLPFGGVGDSGYGRYHGRYSVECFQREKPVLQKTRWGRCLGLGLLSDPSFLYSPQAEWKTKCVRAVASLM
>OtALDH11
MTTSSSSDGFYAELTNAEGGALRYAVNGAWRASSSDATVESVNPSRANARANAFQACTRAEVDEAFAGARAAQGPWARTPLHERASLLHRAATLMRE
NAGGMVSALMIEVAKGAKESATEVERXXXXXXXGNDRNKICMASNVPVGVVLCIPPFNYPVNLCVSKVAPALIAGNAVVVKPPTQGCTATLHMIHCF
IKAGFPPGLIQAVTGRGGEIGDYLTTHPLVNAISFTGGETGIRVAQKAGMVALQTELGGKDACIVLPDADLELAAKSIVKGGFSYSGQRCTAVKIVA
VFEEVADELIGKVNERIAKLSVGLPEDDATITAVVSKSSADFIQSLVVDAESKGATLCQEWKREDNLIWPLLIDNVTHDMKICWEEPFGPVLPVVRV
KNENEALELVNKSRFGLQGCVFTRDIDRAIRLSDAMQTGTVQINGPPARGPDHFPFQGVKDSGIGSQGITNSIKVMTKVKSTVINLAKPSYTIA
>OtALDH5
MSVRARASSSNASAASEVARALGLRRDDLLLDVVREREASTSGTMSVEVTNPATGTTLARCRATSANDVEHILRRSKESQERWANEYTAHARAKIVR
RWFELVEANAEDLARVCTAESGKPLAESRAEVAYAASFLEWFADEGRRVYGDVVPSSSTGSRIMAIKQPVGVTAAITPWNFPLAMITRKAGAALAAG
CSMVVKPSEETPLSAFALGALAKEAGCPDGVLQFVVGDPVVIGELLCASPIVRKITFTGSTRVGKLLMKQSSDTVKRVSMELGGNAPFIVCADADVD
AAVRGAMASKFRNSGQTCVCAQRFIVHESVEDDFVRKLTAAANALKLGNGLEDESVTQGPLINAAQVERVDAHVRDALSKGAVCHSGGKRADGTFYE
PTVLSKCTDDMLVMQEETFGPVAAITTFVSDDDALRMANSTNAGLASYVFTSDLKRSYTFSEKLEFGIVGVNTGVISTAQAPFGGVKESGVGREGGK
YGMEEYVETKYVCVGGLD
>OtALDH22
MSLVRVLTGHVRVALDATNAPEIAREIARVAVTTIEQRHAWTAVIAIAIVLRVLLVERTPKIVVALTEEEASCEGDALTFDPSGAWPTKTVPCYDPG
TMRRLGPDVEAMSAEEVRARIRRASEAQKEWAKSSFATRRKLLRVIQRFILEEQDSICRVSARDSGKPLVDAAFGEVLVTLEKIRWLCNEGEQWLKP
ESRSSGAMMFYKKARVEYHPVGVMGAIVPWNYPFHNVFNPLVANLFAGNALVVKVSEYATWSSQYYGRVIDAALDAVGAPRDLVQIITGYGEAGNAL
VTGGCQKVVFVGSTGIGRKVMEAAAKTLTPVVLELGGKDPFIVCADADLKQCVPMALRGAFQSCGQNCAGAERFYVHEKIHDKFLGKVVDSAKKLRQ
GPSVDKRAIMCMPKQAQYVQSLIDDAVARGAIVHLGAQGGQFYPPTVISGITHDMRIAREEVFGPVLAIVKTKSDEESIALANDCDFGLGSNVFTRS
TKRAEFLGKQLEAGMTSINDFCSTYMAQSLPFGGVKESGFDRFAGIEGLRGCCVPKSVVVDRFPLLMKTNIPPPLCYPVADNAFAFCKALARMFFGL
SLAQRFGGLLALAKCFLLPSKTYTKYD
>OtALDH12
MSRAAGAARGANLIAGAWREITDESRATKIVDPMNKSETDAFIVLPSTDTKQEIDEVARSLASCPKSGLHNAFKAPERYVMWGDVSMRLAQEFRKPE
VEEYFARLIQRVAPKSHAQALAEVVVTRKFLENFAGDNVRFMARGFSVSGDHLGQQSHGLRWPYGPVAVITPFNFPLEIPVLQLMGALFMGNKALVK
SDSKVSVVLEQFIRLMIECGAPATDLDFIHSDGVTMNSILSAAKPKMTLFTGSQKVAHHLARELEGNVKLEDAGFDWKILGPDVGDVDYVAHVCDQD
AYACSGQKCSAQSILFMHKNWVDVGIESKLAALAGERNLEDLTVGPVLTLTTKTMLDHVDRLAALPGARVAFGGKELKDGNHSIPSQYGAIEPTAVF
VPLKTIMASEENFKLVTTEFFGPMQVLTSYDDEELPLVLDACERMDAHLTAAVVSSDEMFSQRVLGSTVNGTTYAGRRARTTGAPQNHWFGPAGTPM
AGGIGTIEAIRLVWSCHREIIFDRGPVESDWKTPPRA
>OtALDH10
MDVRGGALGRAGALIDNAWTTTRRSLPVVNPHDGAVVGAIARGSVADVDDAVRSARKGFVTWSTRNGRERAKTLRAVADGLRRRRETLARLETTDCG
KPLDESAWDVDDAIGCFEYYADRCERVFGERAYAEEVVELPDEDFAGRVRREPLGVIGLITPWNYPLLMATWKVAPALASGCAVVLKPSEEASLTCQ
VLGDVCVEAGLPPGALCVVTGRGDEAGAALCAHRGVDKISFTGSFRTGQTIMRACAQDVKPVSLELGGKSALVIFDDCDLEKAVEWAMFGCFWTNGQ
ICSATSRVLVHENIRERFLARLKEASEAIPVGDPLAEGCRLGPLASAAQYKKVTSMVNRIKRTKIHLLTGGNRPRARGCEKGFYIEPTVFVDPPLDS
EAWREEIFGPVMCVRSFRTEEEVIAITNDSEYALAAAVITDDVARRERMASAFDVGIVWIQCSQPAFTQLPWGGRRRSGFGRDLGVNGMDKYMHQKQ
IVEYTSGAQFEWYPMFKKSKL
